# Discovery of Novel Synthetic Cyclohexene-Based Small Molecules Targeting Senescence against Age-Related Pulmonary Fibrosis

**DOI:** 10.1021/acs.jmedchem.6c00412

**Published:** 2026-07-10

**Authors:** Iván Arribas-Álvarez, Sergio Algar, Pilar Picallos-Rabina, Anabel Sánchez-Merino, Beatriz Marcos-Ramiro, Manuel Collado, Henar Vázquez-Villa, María L. López-Rodríguez, Bellinda Benhamú

**Affiliations:** † Department of Organic Chemistry, Faculty of Chemistry, 16734 Universidad Complutense de Madrid, E-28040 Madrid, Spain; ‡ Laboratory of Cell Senescence, Cancer and Aging, Center for Research in Molecular Medicine and Chronic Diseases (CiMUS), University of Santiago de Compostela, Health Research Institute of Santiago de Compostela (IDIS), E-15706 Santiago de Compostela, Spain; § Department of Immunology and Oncology, National Centre for Biotechnology (CNB-CSIC), E-28049 Madrid, Spain

## Abstract

Targeting cellular senescence has emerged as a therapeutic strategy for the increasingly prevalent age-related diseases, yet no drugs have reached clinical approval specifically as senotherapeutics. Through senescence-phenotype screening of our recently generated human microbiota-inspired library of small molecules, we identified a tetrasubstituted cyclohexene as a novel bioactive chemotype. Following the synthesis of related analogues and their evaluation in relevant models of senescence, we discovered compound **25** (UCM-17017) that decreases β-galactosidase activity in senescent human fibroblasts and selectively reduces the viability of senescent human lung adenocarcinoma cells over proliferative cells. The new senolytic compound exhibits a favorable pharmacokinetic profile *in vivo* and induces a beneficial effect in a mouse model of pulmonary fibrosis, a senescence-related disease with significant unmet medical needs. Our results are valuable for senotherapeutic drug discovery and support the interest of targeting cellular senescence as a promising approach against age-related pulmonary fibrosis.

## Graphical abstract

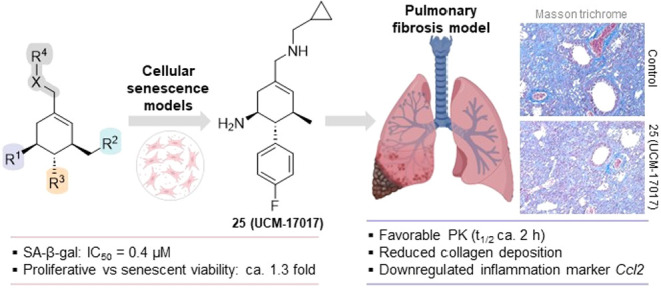

## Introduction

In recent decades, the considerable increase in life expectancy worldwide has led to a higher prevalence of chronic diseases associated with aging. According to the World Health Organization, these so-called age-related diseases, are among the leading causes of disability and morbidity in the elderly population.^1,2^ Indeed, the “geroscience hypothesis” considers aging to be the main risk factor for most serious chronic pathologies—including cancer, neurodegenerative processes, chronic pulmonary diseases, cardiovascular diseases, atherosclerosis, diabetes, osteoporosis, osteoarthritis, hepatic dysfunction, renal failure, and blindness.^3–7^ Thus, addressing an intervention that can slow down the aging process could reduce or postpone the incidence of debilitating age-related diseases, which should significantly reduce the enormous social and economic burden caused by these chronic diseases.^7,8^ Among the accepted hallmarks of aging,^9^ cellular senescence plays a key role;^10–12^ it is characterized by a stable cell-cycle arrest, deregulated metabolism, macromolecular damage, resistance to cell death and secretion of proinflammatory substances (cytokines, chemokines, extracellular matrix remodeling enzymes and growth factors, among others), collectively known as senescence-associated secretory phenotype (SASP).^13^

Senescence is an essential physiological program that occurs in normal cells in response to cellular stress, having beneficial and protective effects for the organism. These effects include tumor suppression by limiting the proliferation of damaged or unwanted cells, as well as limiting the occurrence of fibrosis by favoring tissue repair.^4,14^ However, the accumulation of senescent cells throughout life, mostly due to a weakened immune system, causes numerous adverse effects and contributes to the development of age-related pathologies.^10,15^ Therefore, targeting cellular senescence has emerged as a therapeutic strategy to prevent and mitigate age-related diseases and to increase lifespan and healthspan—defined as total years of life lived in good health and without disability.^3,7,16,17^ In this regard, compounds with antisenescence potential—globally known as senotherapeutics—have been identified as molecules capable of eliminating senescent cells—senolytics—or suppressing the senescence phenotype responsible for inflammation and impaired tissue regeneration—senomorphics.^18^ The anticancer drug dasatinib (D, Figure 1) and the natural flavonoid quercetin (Q, Figure 1) were the first senotherapeutics described in 2015, exhibiting senolytic efficacy in mice.^19^ The combination treatment D+Q has demonstrated efficacy in mouse models of atherosclerosis,^20^ pulmonary fibrosis,^21^ hepatic steatosis,^22^ Alzheimer’s disease^23^ and obesity,^24^ among others. Since then, a number of senolytics have been reported,^25^ mainly based on natural products, such as the flavonoid fisetin^26^ (Figure 1), or from the repurposing of anticancer drugs, such as navitoclax (ABT-263, Figure 1), inhibitor of the BCL-2 family of antiapoptotic proteins,^27^ and cardiac glycosides.^28,29^ The senolytic combination D+Q has entered several clinical trials for the treatment of Alzheimer’s disease,^30^ idiopathic pulmonary fibrosis,^31^ chronic kidney disease,^32^ frailty,^33^ and age-related bone loss.^34^ The human efficacy of the flavonoid fisetin in age-related diseases is also currently being evaluated in several clinical trials for frail elderly syndrome^35^ and knee osteoarthritis therapies.^36^ Among senomorphic compounds, rapamycin (Figure 1), indicated for the prophylaxis of organ transplant rejection, and metformin (Figure 1), a synthetic drug approved for the treatment of type 2 diabetes, have been shown to reduce the accumulation of β-amyloid and tau proteins and improve cognition in animal models.^37–39^ Clinical trials of senomorphic rapamycin for early Alzheimer’s disease^40^ and amyotrophic lateral sclerosis^41^ are presently underway.

**Figure 1:**
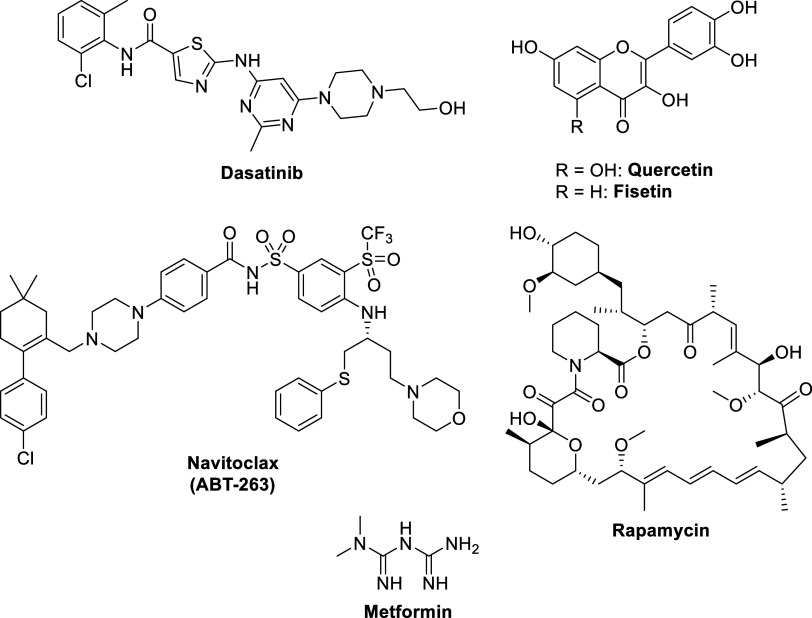
Structures of the most studied senotherapeutic compounds.

Hence, targeting cellular senescence has clearly emerged as a novel strategy to address different age-related pathologies and numerous preclinical studies support the potential of senotherapeutics. However, trials aimed at confirming their clinical indication and long-term safety are progressing slowly, and there are currently no drugs approved specifically as senotherapeutics.^3,42^

In the present work, we contribute to the desired search for novel compounds with senotherapeutic activity using an in-house chemical library that includes our recently generated focused library of small molecules inspired on human microbiota metabolites.^43^ Following the identification of tetrasubstituted cyclohexene scaffold as a new bioactive chemotype, the synthesis of related analogues and their evaluation in relevant cellular senescence phenotypes, we discovered compound **25** (UCM-17017) that leads to a decrease in the activity of senescence-associated β-galactosidase (SA-β-gal), reduces viability of senescent human lung adenocarcinoma cells with specificity over proliferative cells, and exhibits a favorable pharmacokinetic profile *in vivo*. The new senolytic agent induces a beneficial effect in a mouse model of pulmonary fibrosis, one of the most common diseases directly related to the senescence process and with unmet medical needs. Our results are valuable for senotherapeutic drug discovery that could provide new therapies against age-related pulmonary fibrosis.

## Results and Discussion

### Identification of New Senotherapeutic Compounds

The lack of a single and well-established senescence marker makes it difficult to identify a potent, nontoxic and selective senotherapeutic drug. For our primary screening, we have used the enzyme β-galactosidase that is overexpressed in senescent cells and is one of the most widely used biomarkers for senescence detection.^44^ The senescence phenotype was generated using human fibroblasts from lung tissue (IMR-90) that were treated with H_2_O_2_ as oxidative stress. The activity of β-galactosidase in senescent cells was determined by measuring fluorescein emission at 520 nm from the hydrolysis of fluorescein-di-β-galactopyranoside (FDG), and this fluorimetric SA-β-gal assay was used as screening for the identification of compounds affecting cellular senescence.^45^

An in-house chemical library of 256 compounds was first tested at 10 μM and the measured fluorescence intensity indicated β-galactosidase activity related to the senescence phenotype of the cells. Compounds that reduced SA-β-gal activity to less than 55% were subsequently tested at 1 μM. Upon analysis of the screening results, we identified compounds **1** and **S1**–**S9** able to reduce SA-β-gal activity to a value below 55% also at 1 μM (see Table S1). Among them, analogue **1** (Figure 2) exhibited the lowest SA-β-gal activity both at 1 and 10 μM (44% and 16%, respectively). Next, compound **1** was evaluated in nonsenescent IMR-90 cells using the colorimetric MTT assay, resulting in a cell viability of ≈100% that indicates it is not cytotoxic. Considering the results obtained in the FDG SA-β-gal and MTT assays, compound **1** was selected as a new chemotype in the search for synthetic senotherapeutic compounds. Interestingly, **1** was identified within our recently generated set of small molecules inspired on human microbiota metabolites. Indeed, compound **1** contains a central tetrasubstituted cyclohexene core related to shikimic acid, a metabolite of the human microbiota whose biological relevance has been highlighted in a recent study.^46^ Shikimic acid was shown to inhibit the proliferation and migration of vascular smooth muscle cells, suggesting a potential protective role in vascular wall diseases such as atherosclerosis and hypertension. This result provides further evidence for the influence of microbiota metabolites on human health and disease, particularly in age-related pathologies, an area of intense current research.^47–50^

**Figure 2:**
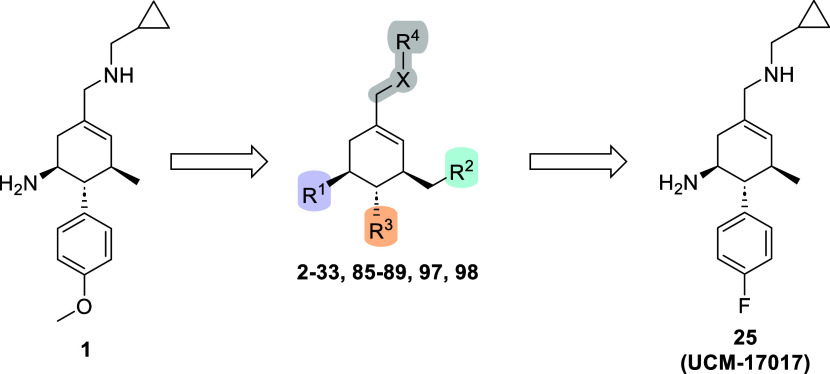
Search of new senotherapeutic compounds related to identified tetrasubstituted cyclohexene derivative **1** and discovery of the new senolytic compound UCM-17017.

For the synthesis of compound **1**, the tetrasubstituted cyclohexene scaffold was built using an asymmetric aminocatalytic cascade (Scheme 1).^51^ This multicomponent reaction affords with high enantio- and diastereoselectivity a chemotype characterized by the presence of three chiral carbon centers, also addressing the demand for compounds with an increased number of sp^3^ carbon atoms, a parameter that has achieved great significance in guiding drug research in recent years.^52,53^ Altogether, the identification of **1** as a bioactive molecule provides rationale to our valuable methodology based on the design of new chemotypes containing privileged scaffolds present in microbiota metabolites and their obtention following synthetic routes based on asymmetric organocatalytic reactions.^43^

**Scheme 1:**
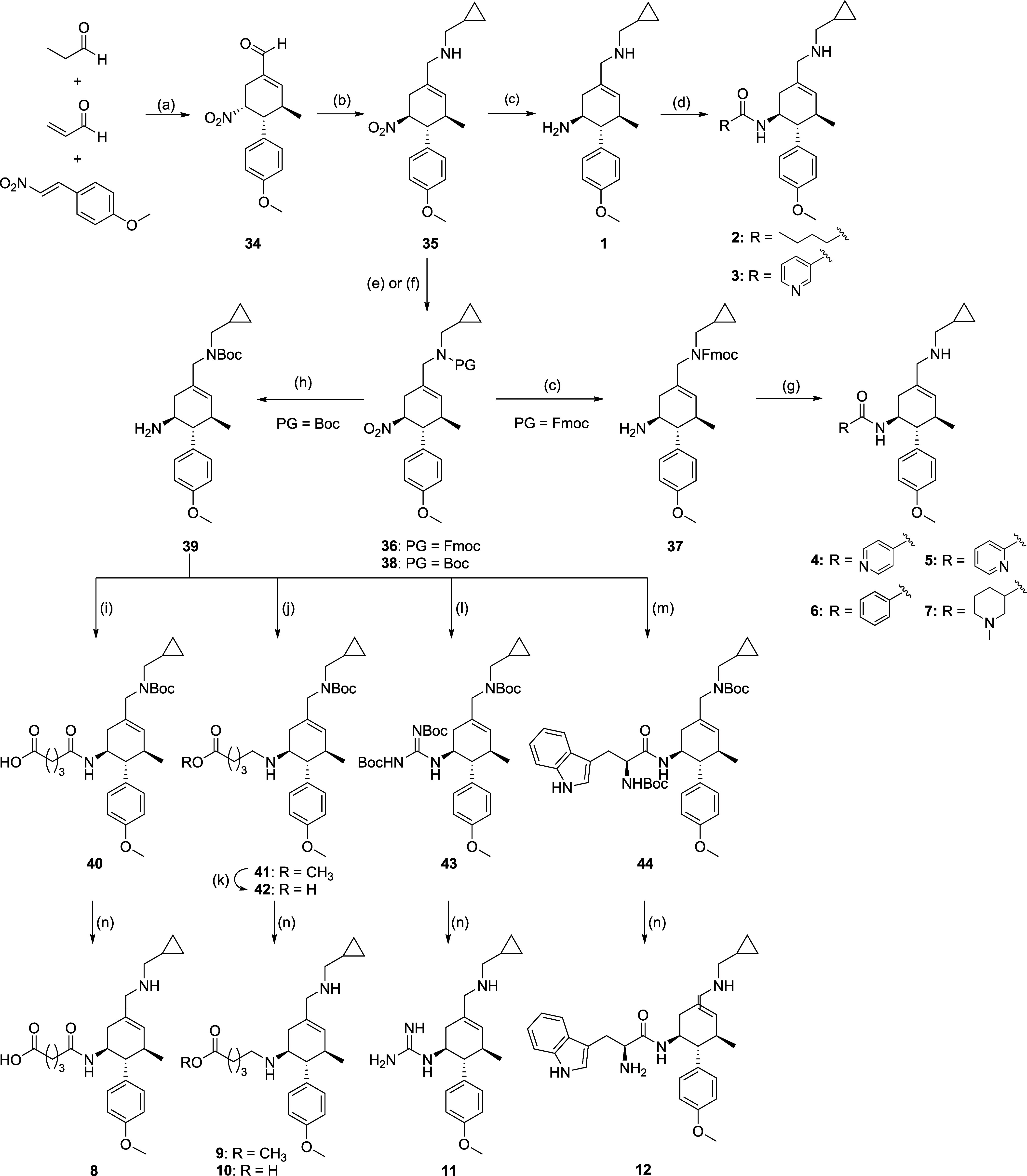
Synthesis of Compounds **1**–**12** ^a^

a Reagents and conditions: (a) 20 mol % (*R*)-2-{diphenyl­[(trimethylsilyl)­oxy]­methyl}­pyrrolidine), toluene, 0 °C, 1 h, rt, on, 50%; (b) i. (cyclopropylmethyl)­amine, MeOH, rt, 3 h; ii. NaBH_4_, rt, 3 h, 57%; (c) Zn, AcOH/MeOH, rt, 1–3 h, 80–90%; (d) valeric or nicotinic acid, EDC, HOBt, DIPEA, DCM, 0 °C to rt, 3 h, 18–36%; (e) Fmoc-OSu, DCM, rt, 3 h, 64%; (f) Boc_2_O, Et_3_N, DCM, rt, 3 h, 75%; (g) i. R-COOH, EDC, HOBt, DIPEA, DCM, 0 °C to rt, 3 h; ii. piperidine, DCM, rt, 8–16 h, 14–22% (for two steps); (h) HSiCl_3_, DIPEA, DCM, rt, on, 74%; (i) glutaric anhydride, DMF, rt, on, 35%; (j) methyl 5-bromovalerate, K_2_CO_3_, KI, ACN, rt, 4 h, 35%; (k) LiOH·H_2_O, THF:H_2_O, rt, 48 h, 62%; (l) *N*,*N*′-di-Boc-1*H*-pyrazole-1-carboxamidine, Et_3_N, ACN, rt, on, 30%; (m) *N*_α_-Boc-L-tryptophan, EDC, HOBt, DIPEA, DCM, rt, 3 h, 34%; (n) HCl (4 M in dioxane), rt, on, 49–75%.

Building upon the tetrasubstituted cyclohexene scaffold of compound **1**, new analogues **2**–**33** were synthesized in the search for novel senotherapeutic agents, incorporating structural modifications at the primary amino group (R^1^), the methyl substituent (R^2^) and the *p*-methoxyphenyl moiety (R^3^) (Figure 2 and Schemes 1–3).

Modifications around the primary amino group of **1** (R^1^, Figure 2) were explored in compounds **2**–**12** (Scheme 1), by introduction of a selection of structural fragments present in numerous microbiota metabolites (short-chain fatty acids; benzoic, nicotinic, isonicotinic, and pipecolic acyl moieties; guanidine group; and tryptophan system). This set of compounds was synthesized following the synthetic route employed for compound **1**, featuring an asymmetric three-component organocatalytic cascade as the key step (Scheme 1).^51^ Hence, the reaction between propionaldehyde, acrolein and *trans*-*p*-methoxy-β-nitrostyrene catalyzed by the *R* enantiomer of the Jørgensen–Hayashi catalyst [(*R*)-2-{diphenyl­[(trimethylsilyl)­oxy]­methyl}­pyrrolidine)] provided the tetrasubstituted cyclohexene scaffold **34** as the major diastereoisomer (diastereoisomeric ratio, dr = 9:1, determined by ^1^H-nuclear magnetic resonance (NMR) analysis of the reaction crude), which was isolated after chromatographic purification. The relative configuration of this intermediate was determined by ^1^H NMR nuclear Overhauser effect (NOE) experiments and agrees with that reported for the asymmetric cascade. The absolute configuration was assigned according to the stereochemical outcome of the reported reaction. Cyclohexenecarbaldehyde **34** was then transformed into amine **35** by reductive amination reaction with (cyclopropylmethyl)­amine (Scheme 1), observing the complete inversion at the chiral carbon bearing the nitro group, which results in a favorable relative *trans* configuration of this group and the aryl ring. Next, reduction of nitro group yielded compound **1** (Scheme 1).

For analogues **2** and **3**, incorporating a valeric acid aliphatic chain and a nicotinic acyl moiety, respectively, a direct coupling reaction of **1** with the corresponding carboxylic acid was performed under standard EDC (1-ethyl-3-(3-(dimethylamino)­propyl)­carbodiimide) conditions, yielding target amides **2** and **3**. However, both compounds were obtained in very low yield due to competing reactivity between the two amino groups present in **1** (Scheme 1). To prevent undesired acylation in the synthesis of final compounds **4**–**7**, the secondary amine of intermediate **35** was protected using an *N*-fluorenylmethoxycarbonyl (Fmoc) group. The resulting *N*-Fmoc derivative **36** was then reduced to amine **37**, which was coupled with the corresponding carboxylic acid followed by deprotection to afford target compounds **4**–**7** (Scheme 1). The yield of the amidation reaction under these conditions remained low, primarily due to partial *in situ* deprotection of **37**, which hindered regioselective acylation. To address this limitation for the synthesis of the remaining analogues, the *tert*-butoxycarbonyl (Boc) group was explored as an alternative protecting group for intermediate **35** (Scheme 1). In this approach, the nitro group of *N*-Boc protected derivative **38** was reduced using trichlorosilane and *N*,*N*-diisopropylethylamine (DIPEA), affording amine **39** that was then used as a common intermediate for the synthesis of final compounds **8**–**12**. Ring-opening reaction of glutaric anhydride with **39** yielded derivative **40**, which, upon treatment with hydrochloric acid, led to final compound **8**. For analogues **9** and **10**, the valeric acid moiety was introduced via alkylation of **39** with methyl 5-bromovalerate, followed by *N*-Boc deprotection of resulting intermediate **41**, to afford final compound **9**. Alternatively, hydrolysis of the ester group in **41** and subsequent acid treatment yielded compound **10**. Finally, derivatives **11** and **12** were synthesized by reaction of **39** with a guanidine precursor and *N*-Boc protected tryptophan, respectively, followed by Boc deprotection.

In compounds **13**–**17** we explored the introduction of microbiota privileged scaffolds such as indole, thiazole, benzothiazole, pyridine, or morpholine, as R^2^ modifications at the methyl group in **1** (Figure 2 and Scheme 2). Cyclohexenecarbaldehyde precursors **45**–**49** were synthesized by applying a modified version of the cascade reaction previously used for compound **34**, in which a nucleophilic component bearing the corresponding R^2^ group is added.^54,55^ Specifically, a multicomponent cascade between acrolein, *trans*-*p*-methoxy-β-nitrostyrene, and indole or an appropriate R^2^-functionalized alcohol acting as nucleophile [(1,3-thiazol-2-yl)­methanol, (1,3-benzothiazol-2-yl)­methanol, (pyridin-2-yl)­methanol, and 2-(morpholin-4-yl)­ethan-1-ol] was carried out, using the Jørgensen–Hayashi catalyst, to yield compounds **45**–**49** (Scheme 2). The experimental conditions of the cascade reaction were optimized to ensure the successful formation of the target intermediates, requiring the slow addition of acrolein to minimize polymerization side reactions. Under these conditions, cyclohexenecarbaldehydes **45**–**49** were obtained with good diastereoselectivity (dr = 8:2–9:1, determined by ^1^H NMR analysis of the reaction crude), and the major diastereoisomer was isolated after chromatographic purification. Subsequent reductive amination to introduce the cyclopropylmethyl fragment, followed by nitro group reduction in intermediates **50**–**54**, afforded the final compounds **13**–**17** (Scheme 2). In all cases, the reductive amination proceeded with complete inversion at the chiral center adjacent to the nitro group, leading to a favorable relative *trans* configuration of the *p*-methoxyphenyl group with respect to the other two substituents of the cyclohexene ring in derivatives **50**–**54**.

**Scheme 2:**
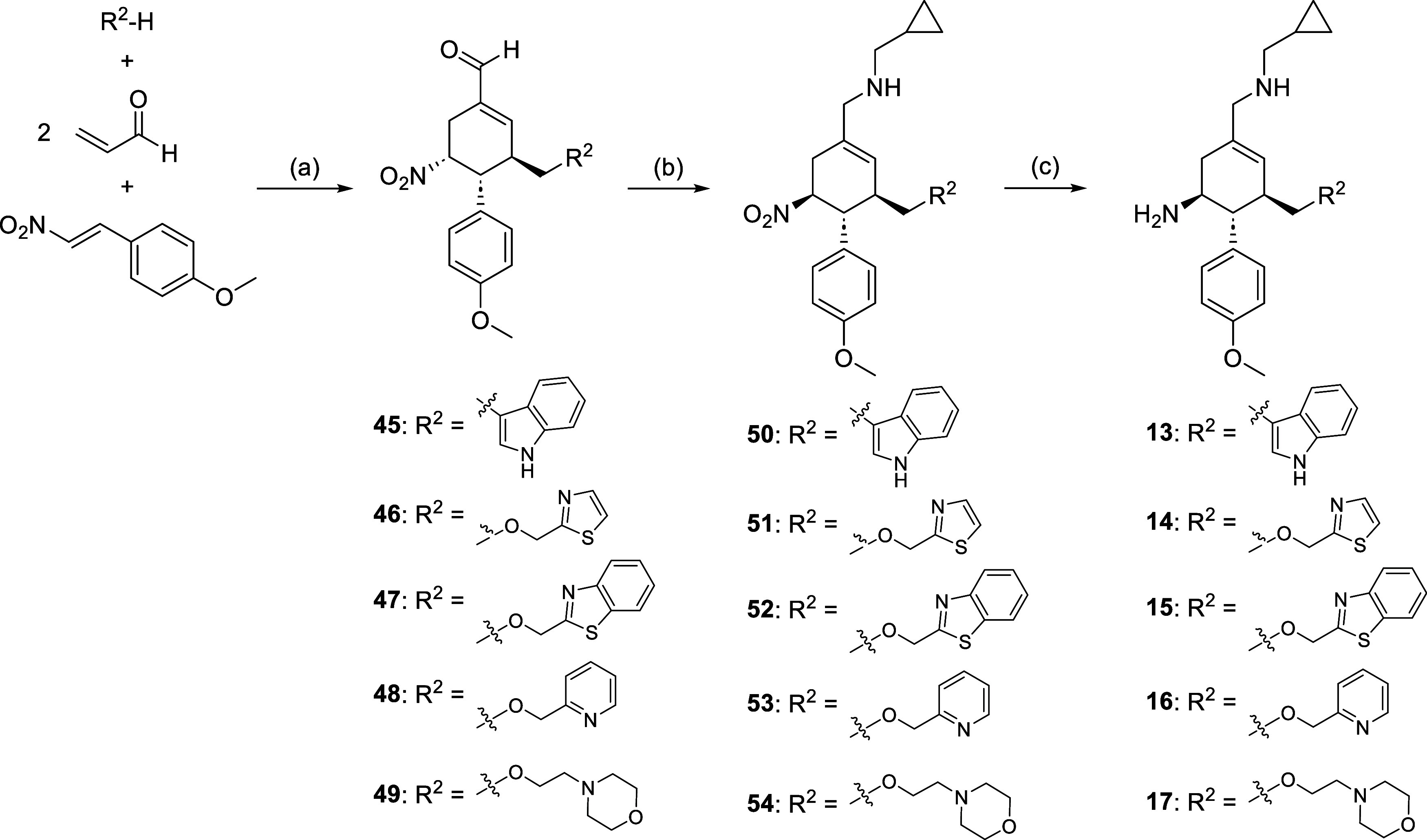
Synthesis of Compounds **13**–**17** ^a^

a Reagents and conditions: (a) 20 mol % (*R*)-2-{diphenyl­[(trimethylsilyl)­oxy]­methyl}­pyrrolidine), 25 mol % benzoic acid, CHCl_3_, rt, on, 28–54%; (b) i. (cyclopropylmethyl)­amine, MeOH, rt, 3 h; ii. NaBH_4_, rt, 3 h, 30–87%; (c) Zn, AcOH/MeOH, rt, 2 h, 37–95%.

For the structural exploration around the aryl group of **1** (R^3^, Figure 2), we considered phenyl rings differently substituted in *ortho*, *meta* and *para* positions (compounds **18**–**29**, **33**, Scheme 3), as well as the replacement with aromatic heterocycles such as pyridine, thiophene, and 1,3-oxazole (compounds **30**–**32**, Scheme 3). The synthesis of these new analogues was approached from cyclohexenecarbaldehyde intermediates **55**–**69**, obtained via an organocatalytic cascade involving acrolein, propionaldehyde and an appropriate nitroalkene containing the desired R^3^ groups (Scheme 3). As expected, the cascade reactions took place with high diastereoselectivity (dr = 9:1), except for intermediate **67**, which exhibited a lower dr (6:4), attributed to epimerization promoted by the basicity of the pyridine. Next, reductive amination followed by nitro group reduction, according to the methodology previously applied, afforded final compounds **18**–**32** (Scheme 3). Analogue **33**, featuring a hydroxy-substituted phenyl ring, was readily obtained by demethylation of compound **1**, using boron tribromide at low temperature (Scheme 3).

**Scheme 3:**
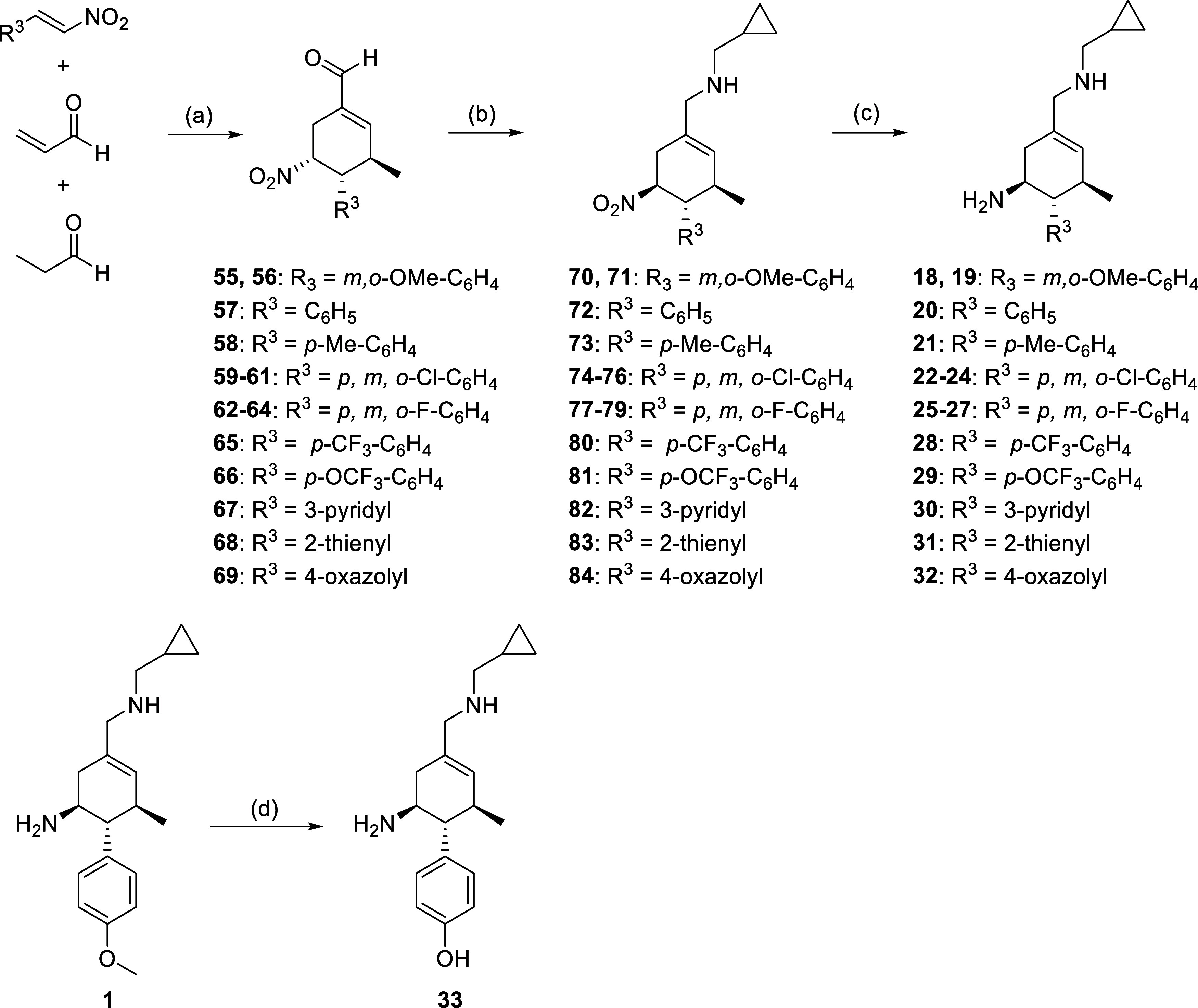
Synthesis of Compounds **18**–**33** ^a^

a Reagents and conditions: (a) 20 mol % (*R*)-2-{diphenyl­[(trimethylsilyl)­oxy]­methyl}­pyrrolidine), toluene, 0 °C, 1 h, rt, on, 19–75%; (b) i. (cyclopropylmethyl)­amine, MeOH, rt, 3 h; ii. NaBH_4_, rt, 3 h, 20–94%; (c) Zn, AcOH/MeOH, 2 h, 16–81%; (d) BBr_3_, DCM, 0 °C, 2 h, 65%.

From the data obtained in the FDG SA-β-gal assay (Table 1), compounds **3**–**6** and **12**, containing aromatic systems in R^1^, were able to reduce SA-β-gal activity to less than 55% and were subsequently tested at 1 μM. At low concentration, only **5** displayed similar senotherapeutic activity to that of parent compound **1**. In addition, compound **5** displayed no cytotoxicity in nonsenescent IMR-90 cells, as determined using MTT assay (viability = 97%).

**Table 1:**
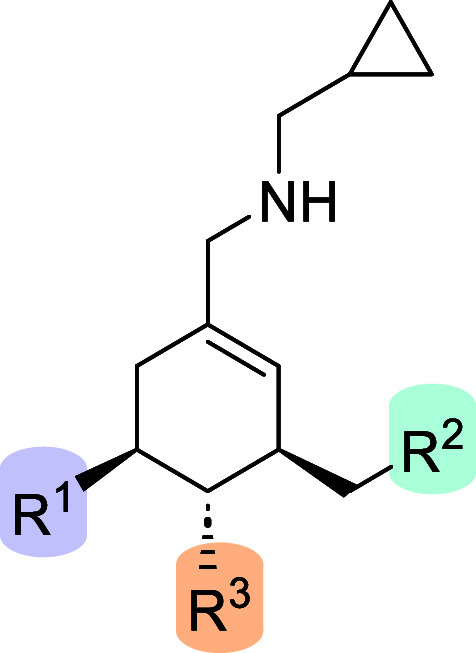

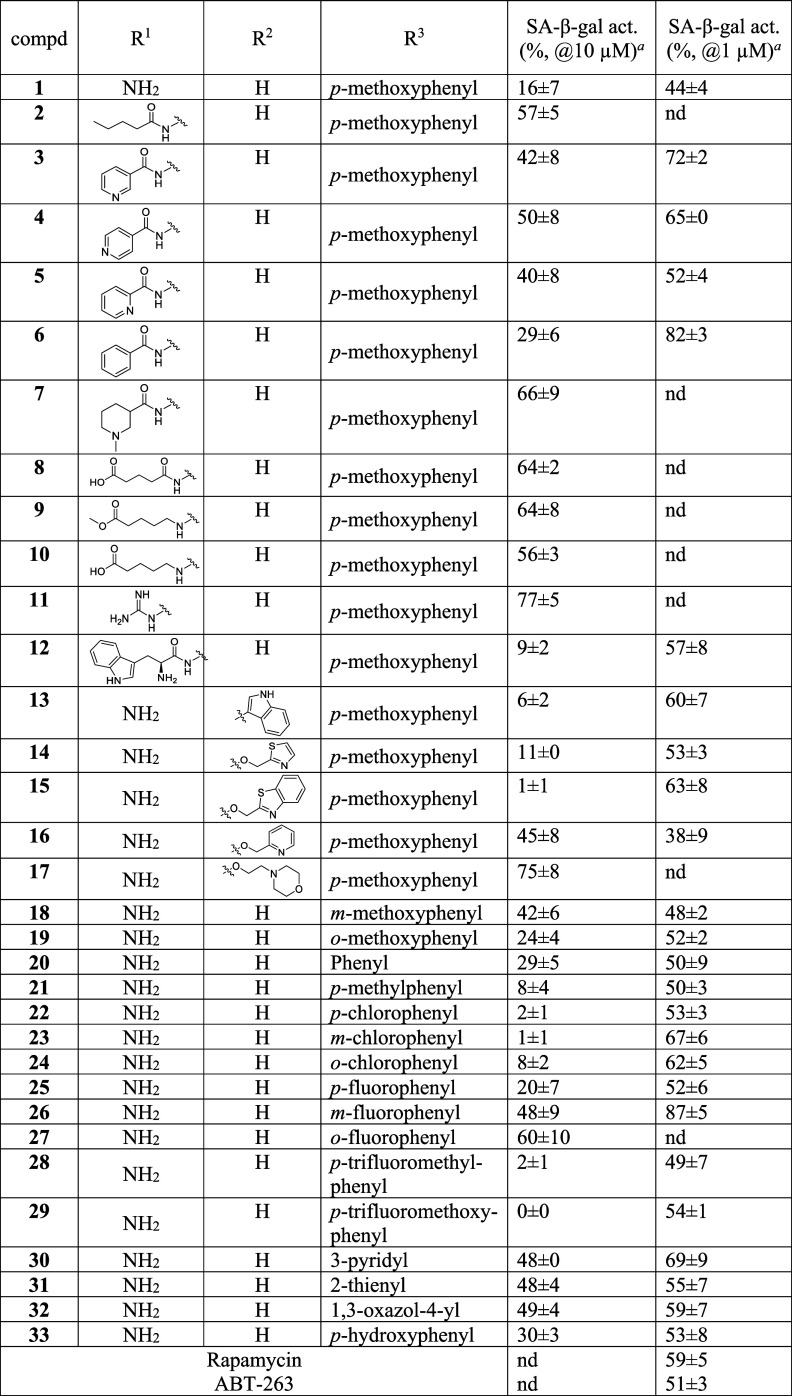
Senotherapeutic Activity of Compounds **1**–**33** in the FDG SA-β-gal Assay

a Values are the mean ± SEM of at least three independent experiments with triplicate determinations; nd = not determined.

New compounds **13**–**17**, resulting from the structural variations at R^2^ moiety, exhibited senotherapeutic activity, with SA-β-gal activity values below 55% at 10 μM except in the case of morpholine derivative **17** (Table 1). Thiazole and pyridine analogues **14** and **16** were also active at 1 μM and displayed no toxicity in nonsenescent cells (viability >97%).

The modifications in R^3^ moiety afforded the most active compounds, since very low SA-β-gal activities were observed at 10 μM, as shown in Table 1 for analogues **18**–**33**. In general, the different substituents were tolerated at all three positions of the phenyl ring, while heterocycles were less favorable (SA-β-gal activity, **30**: 48%, **31**: 48%, **32**: 49%). Compounds containing methyl, chloro, trifluoromethyl, and trifluoromethoxy substituents were able to reduce SA-β-gal activity to less than 10% (see analogues **21–24**, **28** and **29**). When tested at 1 μM, compounds **18–22**, **25**, **28**, **29** and **33** maintained activity and no toxicity was observed in nonsenescent cells (viability >93%).

### Senolytic Activity and Pharmacokinetics of Selected Compounds

Compounds **1**, **5**, **14**, **16**, **18**–**22**, **25**, **28**, **29**, **33**, able to reduce SA-β-gal activity in human senescent fibroblasts to values lower than 55% at 1 μM and devoid of toxicity in nonsenescent cells, were selected for further evaluation of senotherapeutic effects. Their potential as senolytics was assessed in human lung adenocarcinoma (A549) cells treated with bleomycin, a well-known senescence-inducing chemotherapeutic drug. Cell viability by MTT assay was measured in bleomycin-induced senescence A549 cells for the selected compounds at different concentrations (1 and 10 μM) and time points (24, 48, and 72 h) (Figure 3A). From this initial screening, we considered as potential senolytic compounds those above the threshold (the average for the whole population of senescent A549 cells plus 2 standard deviations): **22** (1 μM, 48 h), **29** (10 μM, 48 h), **25** (10 μM, 72 h) and **16** (10 μM, 72 h). Interestingly, selectivity for bleomycin-induced senescent over proliferating A549 cells was observed for compound **25**, with the best performance after 48–72 h post-treatment at both 1 and 10 μM (Figure 3B). The senolytic drug ABT-263 was used as positive control in our viability assays.

**Figure 3:**
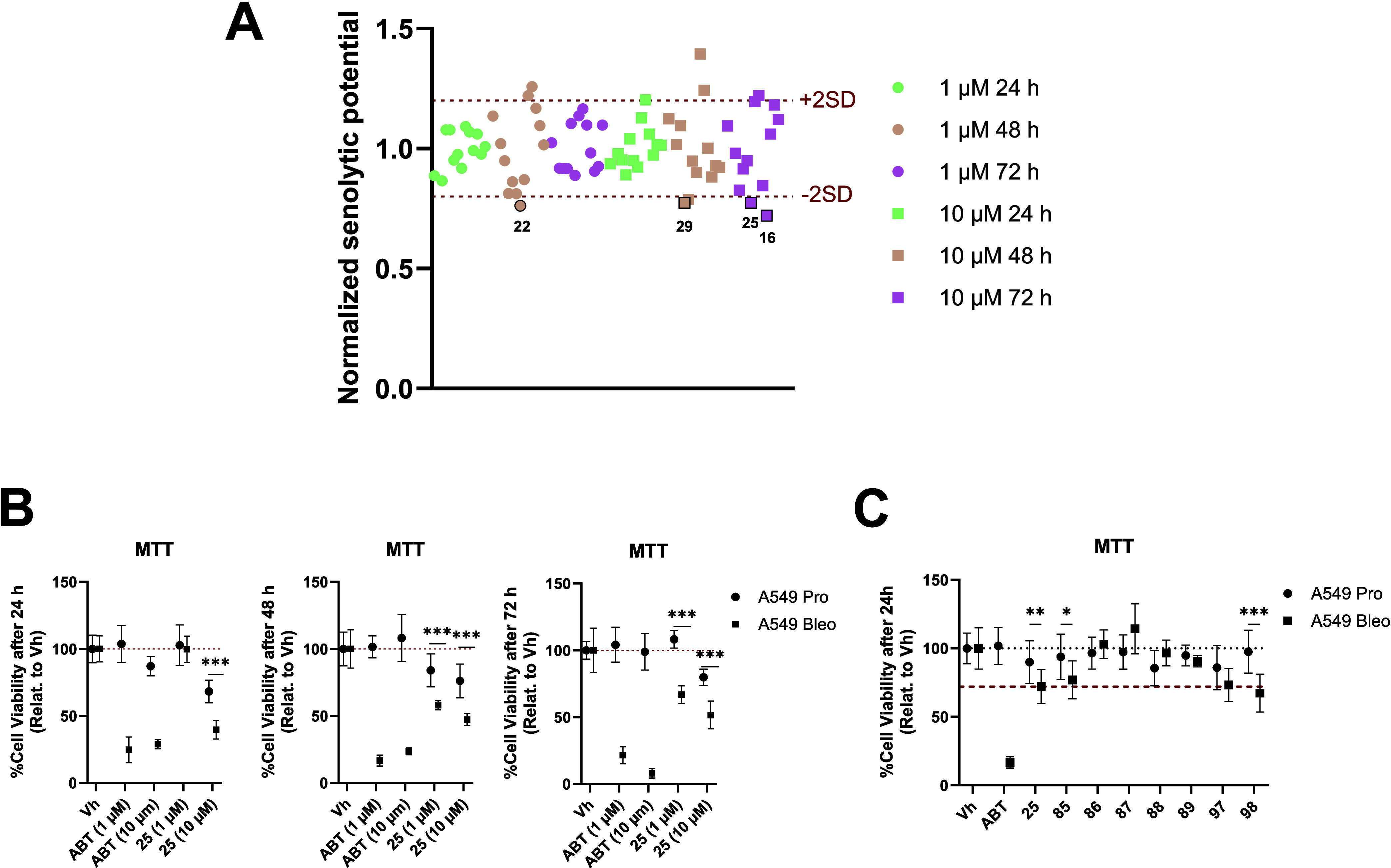
Senolytic activity of selected compounds: viability in senescent and proliferative human lung adenocarcinoma (A549) cells, using an MTT assay. (A) Scatter plot representing the normalized senolytic activity obtained with each compound tested in senescent A549 cells. (B) Relative cell viability (%) of compound **25** tested in proliferative (Pro) and bleomycin-induced senescent (Bleo) A549 cells at 24 (left), 48 (middle), or 72 h (right). (C) Relative cell viability (%) of compounds **25**, **85**–**89**, **97**, and **98**, at 10 μM for 24 h. ABT-263 (ABT) was used as a positive control of senolysis. Statistical significance was assessed by the two-tailed Student’s *t* test: *p < 0.05, **p < 0.01, ***p < 0.001.

Based on these data, we synthesized new analogues **85**–**89** related to compound **25** to explore the replacement of the (cyclopropylmethyl)­amine moiety for amino, methylamino, acetamido, hydroxy, and methoxy groups (X-R^4^), while maintaining the primary amine (R^1^), the methyl group (R^2^) and the *p*-fluorophenyl system (R^3^) (Figure 2 and Scheme 4). Amino derivative **85** was synthesized from cyclohexenecarbaldehyde **62** via reductive amination with methylamine, followed by reduction of the nitro group. For analogues **86** and **87**, intermediate **62** was treated with sodium borohydride to reduce the aldehyde to the corresponding alcohol. This transformation occurred without epimerization at the NO_2_-substituted carbon. Subsequent treatment with pyridine promoted the epimerization, affording stereoisomer **91**, which exhibits the same relative configuration as all final compounds. Next, Mitsunobu reaction between alcohol **91** and phthalimide yielded intermediate **92**, which was then converted into primary amine **93**. From this intermediate, reduction of the nitro group provided final compound **86**, while target acetamide **87** was prepared by acetylation followed by reduction. Regarding the oxygen derivatives (Scheme 4), amino alcohol **88** was obtained by reduction of the nitro group in intermediate **91**. *N*-Boc protection of **88**, *O*-methylation and final deprotection afforded methoxy derivative **89**.

**Scheme 4:**
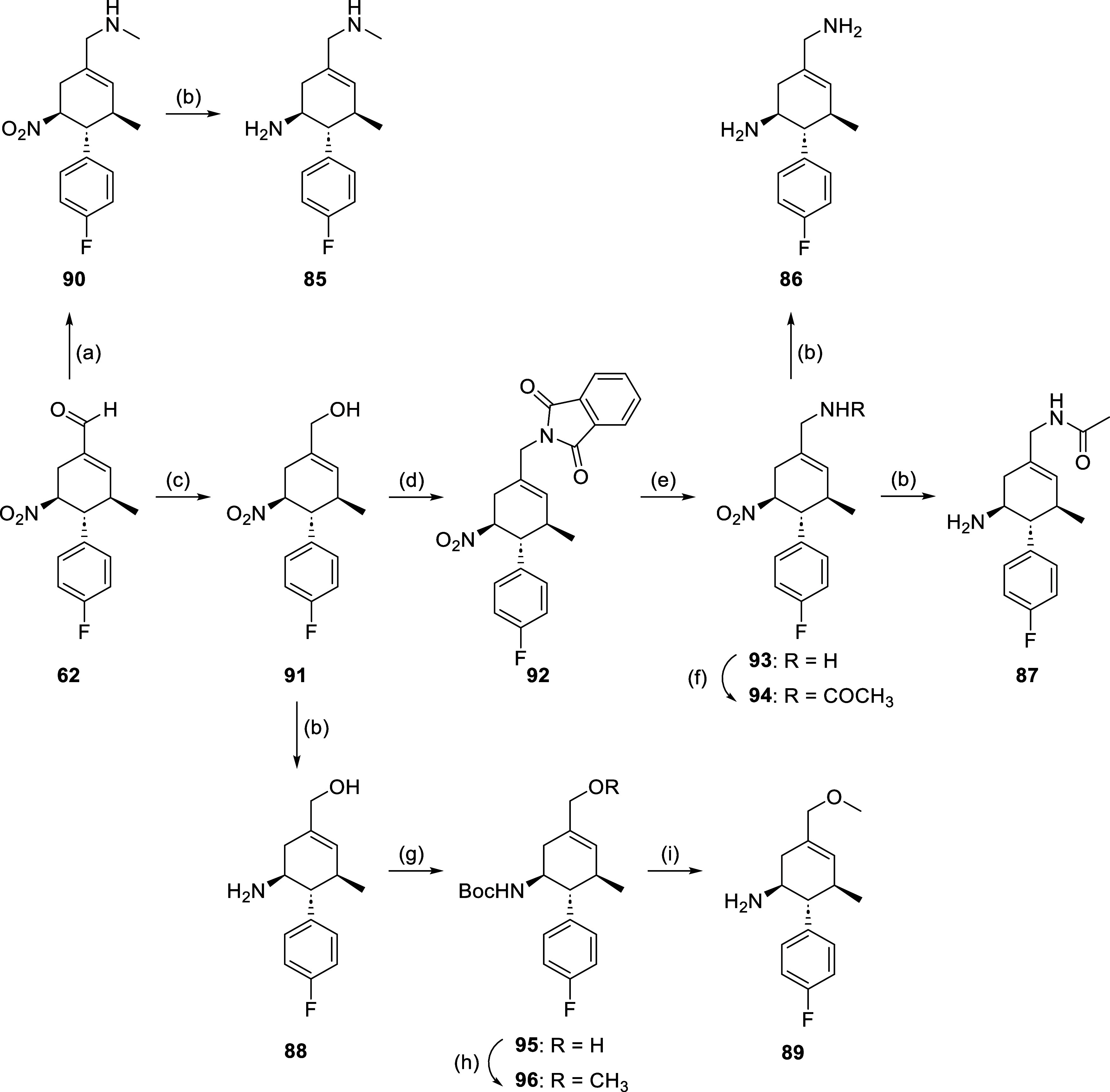
Synthesis of Compounds **85**–**89** ^a^

a Reagents and conditions: (a) i. methylamine, MeOH, rt, 3 h; ii. NaBH_4_, rt, 3 h, 58%; (b) Zn, AcOH/MeOH, 2 h, 51–91%; (c) i. NaBH_4_, MeOH, rt, 2 h; ii. pyridine, EtOAc, rt, 24 h, 75%; (d) phtalimide, PPh_3_, DEAD, THF, rt, 4 h, 64%; (e) N_2_H_4_·H_2_O, EtOH, reflux, 2 h, 72%; (f) Ac_2_O, DCM:py 3:1, 0 °C, 1 h, on, rt, 85%; (b) Boc_2_O, Et_3_N, DCM, rt, 4 h, 64%; (c) i. CsOH·H_2_O, DMF, rt, 30 min; ii. MeI, rt, 4 h, 50%; (d) HCl 4 M in dioxane, rt, on, 63%.

The new compounds **85**–**89** were assessed for inhibition of SA-β-gal activity and viability in both proliferative and senescent A549 cells, revealing that only analogue **85**, bearing a methylamino group, retained the senolytic effect (Table 2 and Figure 3C). According to these results, compounds **25** and **85**, endowed with low SA-β-gal activity (20% and 49% at 10 μM, respectively, Table 2) and senolytic activity (ca. 1.3-fold higher viability in proliferative vs senescent A549 cells, Figure 3C), were assessed for cell permeability. PAMPA revealed limited permeability values (P) of 8.4·10^–6^ and 3.4·10^–6^ cm/s, respectively. Hence, we synthesized new *N*-methyl derivatives **97** and **98** that have higher predicted lipophilicity (clogP) than parent analogues **25** and **85** (Table 2). For the synthesis of these new compounds, both amino groups present in compounds **25** and **85** were protected with the Boc group, followed by methylation with iodomethane, and final deprotection (Scheme 5).

**Scheme 5:**
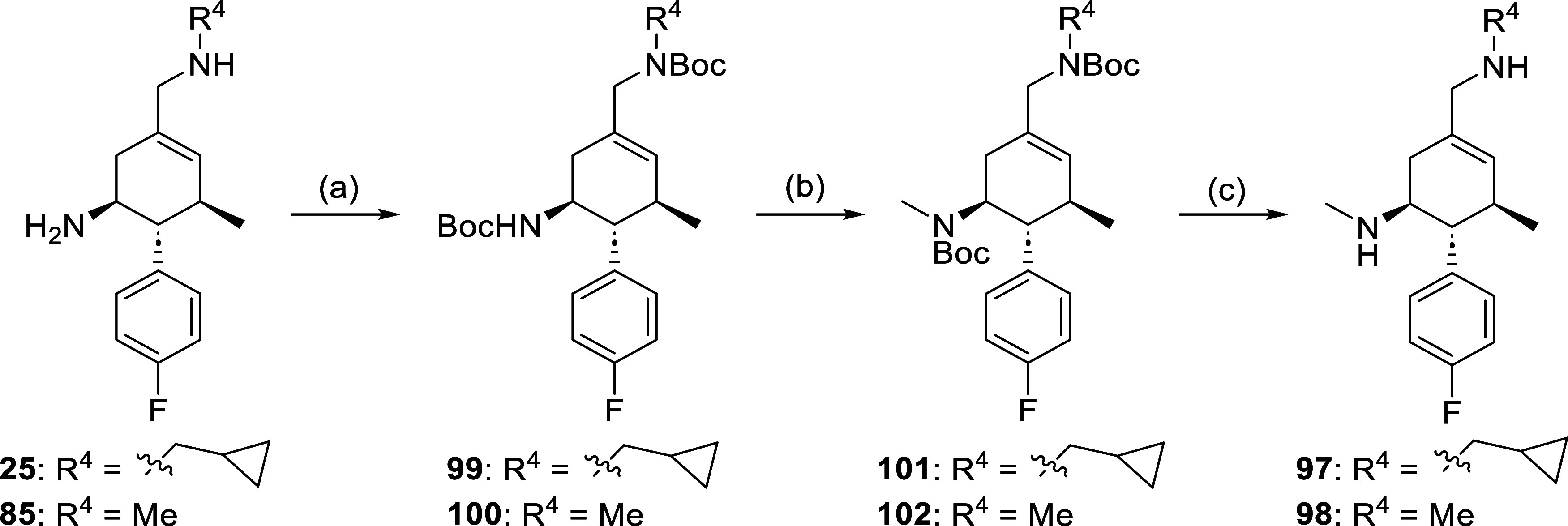
Synthesis of Compounds **97** and **98** ^a^

a Reagents and conditions: (a) Boc_2_O, Et_3_N, DCM, rt, 4 h, 66–84%; (b) i. NaH, DMF, 0 °C, 1 h; ii. MeI, rt, on, 78%; (c) HCl 4 M in dioxane, rt, on, 81–90%.

**Table 2:**
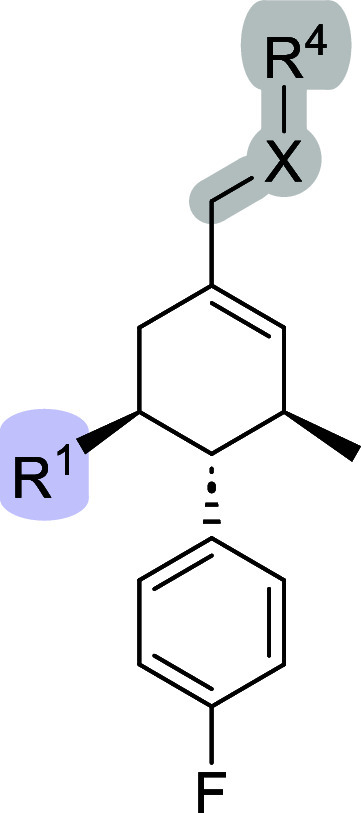

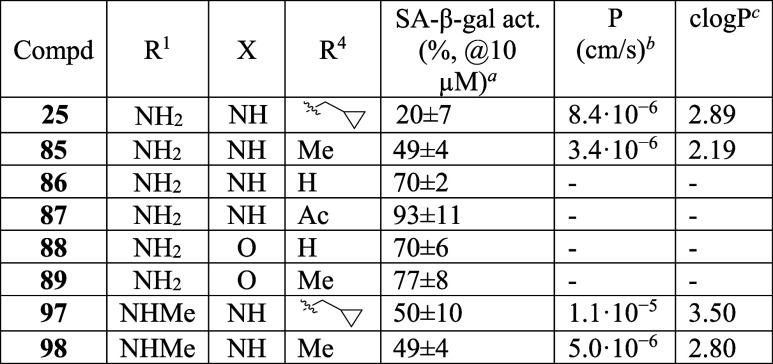
Senotherapeutic Activity of Compounds **85**–**89**, **97**, **98** in the FDG SA-β-gal Assay and Permeability Values

a Data are the mean ± SEM of at least three independent experiments performed in triplicate.

b PAMPA acceptance criteria: *P* ≤ 10^–7^ cm/s for low permeability and *P* ≥ 10^–5^ cm/s for high permeability.

c Value calculated with the ACD/Laboratories Percepta software (version 6.0).

Compound **98** did not exhibit an improved permeability but retained SA-β-gal activity and moreover it showed the highest selectivity for clearing senescent vs proliferative A549 cells (1.5 fold, Figure 3C). Taken together, the data obtained in the *in vitro* senescence models and the permeability assay (Table 2 and Figure 3C) led to the selection of compounds **25** and **98**. The IC_50_ values, determined from the concentration–response curves (0.4 ± 0.1 μM for **25** and 1.4 ± 0.5 μM for **98**, Figure S1), confirmed a potent SA-β-gal inhibition.

Next, we evaluated additional ADME properties. Metabolic stability in mouse (MLM) and human (HLM) liver microsomes was determined and high half-life times (*t*_1/2_) indicated good first-pass metabolism for both compounds (Table 3). Also, in human and mouse serum *t*_1/2_ higher than 48 h were obtained. Binding to HSA protein revealed values around 70% indicating an appropriate capacity of the compounds to be distributed from plasma to cells. In addition, none of the compounds showed relevant inhibition of the hERG channel (IC_50_ >100 μM), suggesting a low potential for cardiotoxicity.

**Table 3:**
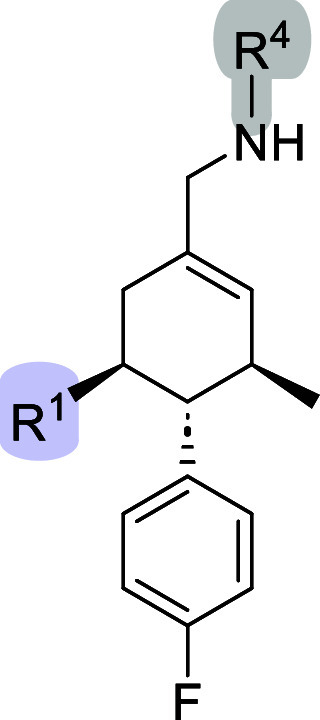

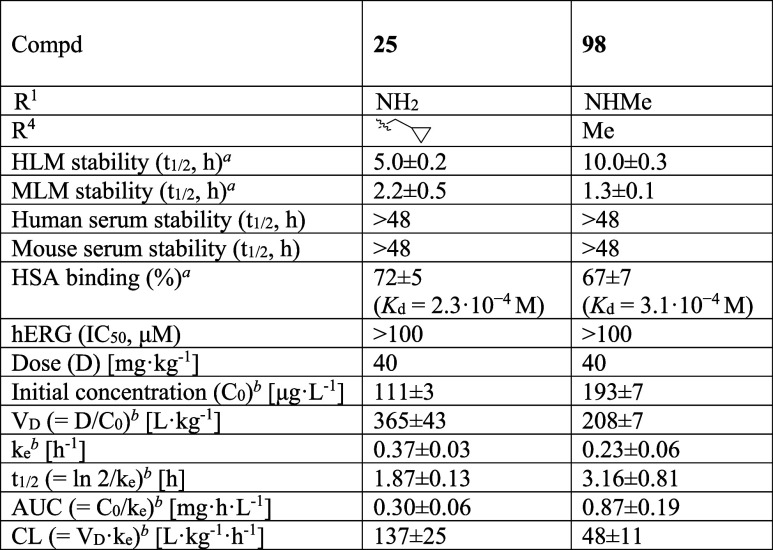
ADME Properties and *In Vivo* Pharmacokinetic Parameters Obtained for Compounds **25** and **98**

a Data are the mean ± SEM of at least two experiments performed in duplicate.

b Parameters obtained from lineal regression: ln­([compound]) = ln­(*C*_0_) – *k*_e_·*t*. *V*_D_: distribution volume; *k*_e_: elimination rate constant; AUC: area under the curve; CL: clearance.

### *In Vivo* Efficacy of the New Senolytic Compounds 25 and 98 in a Mouse Pulmonary Fibrosis Model

*In vivo* pharmacokinetics was addressed for selected compounds **25** and **98**. Following a single intraperitoneal (i.p.) administration of a 40 mg/kg dose to healthy male and female mice, plasma concentrations were quantifiable up to 6 h. The obtained *t*_1/2_ values of ca. 2 h for **25** and longer than 3 h for analogue **98** (Table 3) allowed for the assessment of efficacy *in vivo*. The effect of compounds **25** (UCM-17017) and **98** (UCM-7221) was tested in a bleomycin-triggered mouse model of pulmonary fibrosis. After 2 weeks of intratracheal administration of bleomycin, animals (*n* = 8) were treated intraperitoneally with each tested compound (40 mg/kg) or vehicle for 7 days. The reference senolytic ABT-263 was administered orally (50 mg/kg) to the control group. Lungs were removed at the end of the experiment and were processed for fibrotic and senescence markers. The lung damage caused by bleomycin is linked to weight loss in mice, which was not affected by either compound **25**, **98**, or ABT-263 (Figure 4A). Masson trichrome staining in the lungs confirmed that bleomycin treatment causes the accumulation of collagen, leading to fibrotic tissue formation (Figure 4B, upper panels). Treatment with compound **25** significantly reduced collagen deposition in the lung to levels similar to those seen with the reference senolytic ABT-263. However, no effect was observed for compound **98**. SA-β-gal activity was also quantified via histochemical staining of the lungs and a moderate reduction in the senescence marker was observed for compound **98**, whereas **25** and ABT-263 showed a nonsignificant trend (Figure 4B, bottom panels). This observation is consistent with previous reports highlighting the cell-type specificity of ABT-263 senolytic activity.^21,27^ Likewise, compound **98** may preferentially target a subset of senescent cells that do not play a central role in driving fibrosis in this model. Additionally, we measured transcriptional levels of markers for senescence (*Cdkn1a*), fibrosis (*Col1a1*) and inflammation (*Ccl2*), as shown in Figure 4C. Compound **25** was able to reduce the expression levels of *Ccl2*, a key marker of inflammation and extracellular matrix remodeling, which plays a pivotal role in senescence and fibrosis.^56,57^ Importantly, hematoxylin-eosin (H&E) staining and the absence of cleaved caspase-3 expression in liver and kidney tissues collected from treated mice indicated a lack of treatment-induced structural damage, apoptosis or toxicity in both key metabolic organs (Figure S2). These preliminary data support the safety profile of compound **25** at a dose of 40 mg/kg *in vivo*.

**Figure 4:**
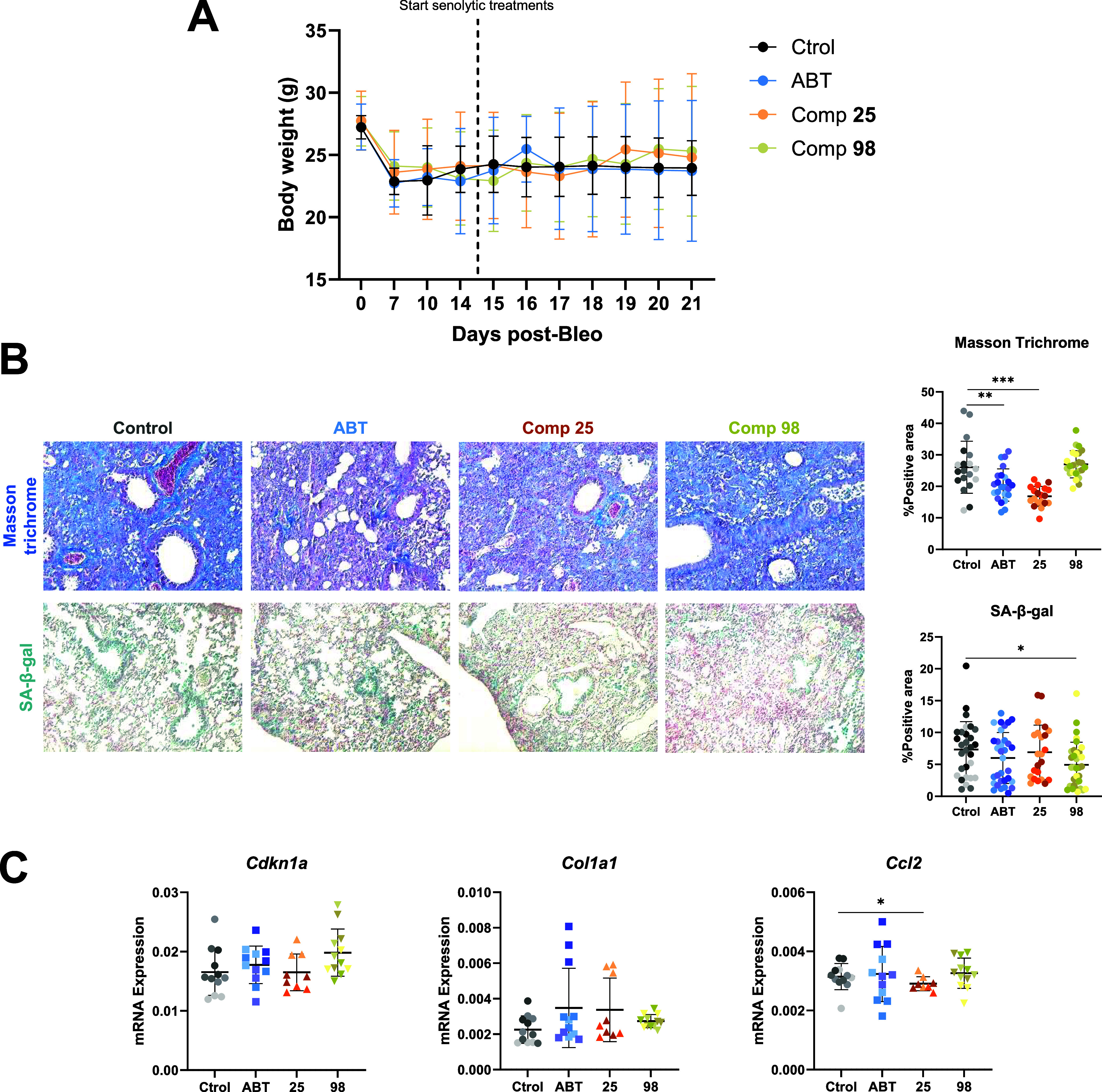
*In vivo* characterization of compounds **25** and **98** in a pulmonary fibrosis mouse model. (A) Body weight measurement of mice throughout the experiment (Ctrol: control group; ABT: ABT-263 treated group; Comp **25**: compound **25** treated group; Comp **98**: compound **98** treated group). (B) Immunohistochemical images of lungs stained for collagen deposition by Masson trichrome (top left) and SA-β-gal (bottom left) staining, and corresponding quantifications (right). (C) mRNA expression (relative to *Gapdh*) of the senescence marker *Cdkn1a* (left), the fibrosis marker *Col1a1* (middle), and the inflammation marker *Ccl2* (right). Statistical significance was assessed by the Mann–Whitney test: *p < 0.05, **p < 0.01, ***p < 0.001.

Taken together, compound **25** shows cellular senolytic activity, and its *in vivo* effect in the bleomycin-induced model primarily involves the modulation of the pro-inflammatory and pro-fibrotic environment. These results show the efficacy of the new senolytic agent in targeting pulmonary fibrosis.

## Conclusion

In the screening of our in-house chemical library in relevant cellular senescence phenotypes, we identified tetrasubstituted cyclohexene scaffold as a new bioactive chemotype. Following the synthesis and evaluation of related analogues, we have discovered compound **25** (UCM-17017) that decreases SA-β-gal activity in senescent human fibroblasts, reduces the viability of senescent human lung adenocarcinoma (A549) cells over proliferative cells, and exhibits a moderate *in vitro* ADME profile, which translated into favorable pharmacokinetics *in vivo*. In a mouse model of pulmonary fibrosis, the new senolytic compound UCM-17017 induces a significant reduction in collagen deposition and in the expression levels of the inflammation marker *Ccl2*. Our results reinforce the targeting of cellular senescence as a promising strategy to provide new therapies against pulmonary fibrosis, one of the most common diseases directly related to the senescence process and with unmet medical needs.

## Experimental Section

### Synthesis

The starting materials, reagents, and solvents were purchased as high-grade commercial products from Sigma-Aldrich (Merck), Fisher, Acros, ABCR, Fluorochem, or Scharlab. Dichloromethane (DCM), tetrahydrofuran (THF) and diethyl ether were dried using a Pure Solv Micro 100 Liter solvent purification system. Ethanol-free chloroform was obtained by washing with water and subsequent distillation over P_2_O_5_.

Analytical thin-layer chromatography (TLC) was run on Merck silica gel plates (Kieselgel 60 F-254), with detection by UV light (λ = 254 nm), 5% ninhydrin solution in ethanol, or 10% phosphomolybdic acid solution in ethanol. Unless otherwise stated, products were purified in a Biotage Selekt system using silica gel cartridges (Biotage Sfär, size particle 60 μM).

All compounds were obtained as oils, except those whose melting points (mp) are indicated, which were solids. Mp were determined on a Stuart Scientific electrothermal apparatus. Infrared (IR) spectra were measured on a Bruker Tensor 27 instrument equipped with a Specac attenuated total reflection (ATR) accessory of 5200–650 cm^–1^ transmission range; frequencies (ν) are expressed in cm^–1^. ^1^H- and ^13^C NMR spectra were recorded on a Bruker Avance III 700 MHz (^1^H, 700 MHz; ^13^C, 175 MHz), Bruker Avance 500 MHz (^1^H, 500 MHz; ^13^C, 125 MHz) or Bruker DPX 300 MHz (^1^H, 300 MHz; ^13^C, 75 MHz) instrument at rt at the Universidad Complutense de Madrid (UCM) NMR facility. Bruker DPX 300 MHz spectrometer was used unless otherwise stated. Chemical shifts (δ) are expressed in parts per million relative to the residual solvent peak for ^1^H and ^13^C nuclei (CDCl_3_: δ_H_ = 7.26, δ_C_ = 77.16; MeOH-*d*4: δ_H_ = 3.31, δ_C_ = 49.00) and to internal (trifluoromethyl)­benzene for ^19^F nucleus; coupling constants (*J*) are in hertz (Hz). The following abbreviations are used to describe peak patterns when appropriate: s (singlet), d (doublet), t (triplet), q (quadruplet), quint (quintuplet), m (multiplet), and br (broad). 2D NMR experiments—homonuclear correlation spectroscopy (H,H–COSY), heteronuclear multiple quantum correlation (HMQC), and heteronuclear multiple bond correlation (HMBC)—of representative compounds were acquired to assign protons and carbons of new structures. The following abbreviations have been used for the peak assignment: cpr (cyclopropane), ind (indole), py (pyridine), thz (thiazole), morp (morpholine), and ox (oxazole). The relative configuration of the compounds was confirmed by 1D ^1^H NMR NOE experiments, in which the signal of interest was irradiated with a selective pulse and NOE interactions were observed. Numbered chemical structures for NMR assignation of compounds **1**, **25**, **34**, **35**, **62**, **77**, **98**, **100**, and **102** described in this section are shown in Figure S3. High-resolution mass spectrometry (HRMS) was carried out on Bruker Impact II QTOF mass spectrometer in electrospray ionization (ESI) mode at UCM’s mass spectrometry facilities.

For all final compounds, a purity of at least 95% was determined by HPLC-MS using an Agilent 1200LC-MSD VL instrument. LC separation was achieved with a Zorbax SB-C3 column (5 μm, 2.1 mm × 50 mm) or an Eclipse XDB-C18 column (5 μm, 4.6 mm × 150 mm), together with a guard column (5 μm, 4.6 mm × 12.5 mm). The mobile phase consisted of water and acetonitrile (ACN) with 0.1% formic acid as solvent modifier, and the gradients are indicated in Table S2. MS analysis was performed using an electrospray irradiation source. The capillary voltage was set to 3.0 kV and the fragmentor voltage to 72 eV. The drying gas temperature was 350 °C, the drying gas flow was 10 L/min, and the nebulizer pressure was 20 psi. Spectra were acquired in positive or negative ionization mode from 80 to 800 *m*/*z* and in UV-mode at four different wavelengths (210, 230, 254, and 280 nm).

Optical rotation [α] was measured on an Anton Paar MCP 100 modular circular polarimeter using a sodium lamp (λ = 589 nm) and a 0.1 dm path length; concentrations (c) are given in g/100 mL. The enantiomeric ratio (er) of final compounds **25** and **98** was determined by chiral HPLC using Chiralpak IA and IC columns. HPLC traces were compared with those obtained from the corresponding racemic samples. The enantiomers of **25** and **98** were prepared following the same synthetic route, employing the *S*-enantiomer of the Jørgensen–Hayashi catalyst in the initial asymmetric cascade forming the cyclohexene scaffold.

Final compounds **1**–**33**, **85**–**89**, **97** and **98** were characterized (α, R_f_, IR, NMR, HPLC-MS) and subsequently transformed into the corresponding hydrochloride salts. Thus, a commercial solution of 2 M HCl (g) in diethyl ether (3 mL/mmol) was added to a solution of the compound in anhydrous DCM or methanol (MeOH) (6 mL/mmol). The resulting salt was isolated by filtration or evaporation of the solvent, washed with anhydrous diethyl ether, and dried under vacuum. A purity of at least for 95% for the salts was determined by HPLC-MS and elemental chemical analysis (C, H, N, S) using a LECO CHNS-932 instrument at the UCM Elemental Analysis facility.

IUPAC rules have been followed to name all organic compounds, except for 1-methoxy-4-[(*E*)-2-nitroethenyl]­benzene and pyridine-2-carboxylic, pyridine-3-carboxylic, pyridine-4-carboxylic and pentanoic acids, whose common names *trans*-*p*-methoxy-β-nitrostyrene and picolinic, nicotinic, isonicotinic and valeric acids, respectively, have been employed for simplicity.

No unexpected or unusually high safety hazards were encountered during the course of the experimental work reported.

### General Procedure A: Three-Component Cascade Reaction

To a solution of the corresponding nitroalkene (1.00 equiv) and (*R*)-2-{diphenyl­[(trimethylsilyl)­oxy]­methyl}­pyrrolidine (0.20 equiv) in anhydrous toluene (0.8 mL/mmol) at 0 °C, propionaldehyde (1.20 equiv) and acrolein (1.05 equiv) were added successively. After stirring at 0 °C for 1 h, the reaction was warmed up to rt and stirred overnight. Then, the solvent was removed under reduced pressure and the residue was purified by flash chromatography to afford the corresponding cyclohexenecarbaldehyde **34**, **55**–**69**.

#### (1*R*,2*R*,6*R*)-4′-Methoxy-6-methyl-2-nitro-1,2,3,6-tetrahydro­[1,1′-biphenyl]-4-carbaldehyde, **34**

Following general procedure A using *trans*-*p*-methoxy-β-nitrostyrene (550 mg, 3.07 mmol), compound **34** was obtained as a yellow solid (423 mg, 50%). Chromatography: hexane to hexane/EtOAc 7:3. Mp: 126–128 °C. R_f_: 0.33 (hexane/EtOAc 7:3). [α]_20_^D^ = −12.9 (c = 1.10, CHCl_3_). IR (ATR): ν 1681 (C=O), 1546 (NO_2_), 1251 (COC). ^1^H NMR (CDCl_3_, 700 MHz): δ 1.22 (d, *J* = 7.2, 3H, CH_3_), 2.80 (dt, *J* = 5.4, 1.7, 2H, 2H_3_), 3.12 (dd, *J* = 6.9, 3.8, 1H, H_1_), 3.16–3.21 (m, 1H, H_6_), 3.79 (s, 3H, OCH_3_), 4.90 (td, *J* = 5.6, 3.8, 1H, H_2_), 6.84 (d, *J* = 8.7, 2H, H_3′_, H_5′_), 6.91 (dt, *J* = 3.2, 1.7, 1H, H_5_), 7.00 (d, *J* = 8.7, 2H, H_2′_, H_6′_), 9.57 (s, 1H, CHO). ^13^C NMR (CDCl_3_, 175 MHz): δ 19.9 (CH_3_), 24.6 (C_3_), 34.4 (C_6_), 48.9 (C_1_), 55.4 (OCH_3_), 83.9 (C_2_), 114.5 (C_3′_, C_5′_), 128.8 (C_2′_, C_6′_), 129.5 (C_1′_), 136.0 (C_4_), 154.2 (C_5_), 159.5 (C_4′_), 192.6 (CHO). 1D ^1^H NMR NOE: irradiation of the signal at δ 3.16–3.21 ppm (m, H_6_) yielded NOE on 7.00 (d, H_2′_, H_6_); and irradiation of the signal at δ 4.90 ppm (td, H_2_) yielded NOE on 3.12 (dd, H_1_). HPLC (method A, t_R_, min): 12.9. MS (ESI, *m*/*z*, %): 274.1 ([M-H]^−^, 100).

#### (1*R*,2*R*,6*R*)-4′-Fluoro-6-methyl-2-nitro-1,2,3,6-tetrahydro­[1,1′-biphenyl]-4-carbaldehyde, **62**

Following general procedure A using 1-fluoro-4-[(*E*)-2-nitroethenyl]­benzene (400 mg, 2.40 mmol), compound **62** was obtained as a yellow oil (198 mg, 31%). Chromatography: hexane to hexane/EtOAc 6:4. R_f_: 0.43 (hexane/EtOAc 7:3). [α]_20_^D^ = −73.0 (c = 1.0, CHCl_3_). IR (ATR): ν 1682 (C=O), 1510 (NO_2_). ^1^H-RMN (CDCl_3_): δ 1.22 (d, *J* = 6.9, 3H, CH_3_), 2.81 (dt, *J* = 6.4, 1.7, 2H, 2H_3_), 3.13–3.22 (m, 2H, H_1_, H_6_), 4.91 (td, *J* = 5.5, 3.4, 1H, H_2_), 6.89–6.91 (m, 1H, H_5_), 7.01–7.07 (m, 4H, H_2′_, H_3′_, H_5′_, H_6’_), 9.57 (s, 1H, CHO). ^13^C-RMN (CDCl_3_): δ 19.8 (CH_3_), 24.6 (C_3_), 34.2 (C_6_), 48.9 (C_1_), 83.7 (C_2_), 116.1 (d, *J* = 21.4, C_3′_, C_5′_), 129.4 (d, *J* = 8.1, C_2′_, C_6′_), 133.4 (d, *J* = 3.4, C_1′_), 135.9 (C_4_), 153.7 (C_5_), 162.6 (d, *J* = 247.3, C_4′_), 192.5 (CHO). HPLC (method B, t_R_, min): 10.50.

### General Procedure B: Four-Component Cascade Reaction

To a solution of (*R*)-2-{diphenyl­[(trimethylsilyl)­oxy]­methyl}­pyrrolidine (0.20 equiv), the corresponding nitroalkene (1.00 equiv) and nucleophile (1.20 equiv), and benzoic acid (0.25 equiv) in ethanol-free chloroform (1 mL/mmol), a 1 M solution of acrolein in ethanol-free chloroform (3.00 equiv) was added via a syringe pump (0.9 mL/h). After addition was complete, the reaction mixture was stirred at rt overnight. Then, the solvent was removed under reduced pressure and the residue was purified by flash chromatography to afford the corresponding cyclohexenecarbaldehyde **45**–**49**.

### General Procedure C: One-Pot Reductive Amination

To a solution of the corresponding aldehyde (1.00–1.50 equiv) in anhydrous methanol (5 mL/mmol) and DCM (2 mL/mmol; only in those cases where the aldehyde is not soluble in methanol) under nitrogen atmosphere, the appropriate amine (1.00–2.00 equiv) was added and the reaction mixture was stirred at rt for 2–4 h to form the corresponding imine (confirmed by ^1^H NMR analysis of an aliquot). Then, NaBH_4_ (2.00 equiv) was added at 0 °C and the mixture was allowed to react at rt for 3 h. The reaction was quenched with a sat. NaHCO_3_ solution and the solvent was evaporated under reduced pressure. The residue was suspended in water and extracted with EtOAc (×2). The organic layers were washed with brine, dried over Na_2_SO_4_, filtered, and evaporated under reduced pressure to afford the corresponding amine **35**, **50**–**54**, **70**–**84**, **90**, which was purified by flash chromatography or used in the next step without further purification.

#### 1-Cyclopropyl-*N*-{[(1*R*,2*S*,6*R*)-4′-methoxy-6-methyl-2-nitro-1,2,3,6-tetrahydro­[1,1′-biphenyl]-4-yl]­methyl}­methanamine, **35**

Following general procedure C using **34** (450 mg, 1.64 mmol) and (cyclopropylmethyl)­amine (0.28 mL, 3.28 mmol), compound **35** was obtained as a yellow oil (257 mg, 57%). Chromatography: DCM to DCM/MeOH/NH_3_ 9:1:0.1. R_f_: 0.48 (DCM/MeOH/NH_3_ 9:1:0.1). [α]_20_^D^ = −4.9 (c = 0.62, CHCl_3_). IR (ATR): ν 3311 (NH), 1550 (NO_2_), 1251 (COC). ^1^H NMR (CDCl_3_, 700 MHz): δ 0.12–0.14 (m, 2H, CH_2cpr_), 0.49–0.52 (m, 2H, CH_2cpr_), 0.90 (d, *J* = 7.0, 3H, CH_3_), 0.94–1.00 (m, 1H, CH_cpr_), 1.73 (br s, 1H, NH), 2.44–2.48 (m, 3H, H_6_, NHCH_2_CH), 2.72–2.84 (m, 2H, 2H_3_), 2.82 (t, *J* = 11.1, 1H, H_1_), 3.24 (AB system, *J* = 13.8, 2H, NHCH_2_), 3.78 (s, 3H, OCH_3_), 4.96 (td, *J* = 11.1, 5.5, 1H, H_2_), 5.56 (s, 1H, H_5_), 6.83 (d, *J* = 8.2, 2H, H_3′_, H_5′_), 7.10 (d, *J* = 8.3, 2H, H_2′_, H_6′_). ^13^C NMR (CDCl_3_, 175 MHz): δ 3.6 (2CH_2cpr_), 11.3 (CH_cpr_), 19.7 (CH_3_), 33.4 (C_3_), 37.5 (C_6_), 51.8 (C_1_), 54.6 (NHCH_2_CH), 54.8 (NHCH_2_), 55.3 (OCH_3_), 88.8 (C_2_), 114.2 (C_3′_, C_5′_), 128.4 (C_5_), 129.1 (C_2′_, C_6′_), 130.6 (C_1′_), 131.8 (C_4_), 159.0 (C_4′_). 1D ^1^H NMR NOE: irradiation of the signal at δ 0.90 ppm (d, CH_3_) yielded NOE on 2.82 (t, H_1_); irradiation of the signal at δ 2.44–2.48 ppm (m, H_6_) yielded NOE on 7.10 (d, H_2′_, H_6′_); and irradiation of the signal at δ 4.96 ppm (td, H_2_) yielded NOE on 7.10 (d, H_2′_, H_6′_). HPLC (method A, t_R_, min): 15.25. MS (ESI, *m*/*z*, %): 331.1 ([M + H]^+^, 100).

#### 1-Cyclopropyl-*N*-{[(1*R*,2*S*,6*R*)-4′-fluoro-6-methyl-2-nitro-1,2,3,6-tetrahydro­[1,1′-biphenyl]-4-yl} methanamine, **77**

Following general procedure C using **62** (188 mg, 0.71 mmol) and (cyclopropylmethyl)­amine (0.12 mL, 1.42 mmol), compound **77** was obtained as a yellow oil (102 mg, 45%). Chromatography: DCM to DCM/EtOH/NH_3_ 9:1:0.1. R_f_: 0.69 (DCM/MeOH/NH_3_ 9:1:0.1). [α]_20_^D^ = −3.0 (c = 1.0, CHCl_3_). IR (ATR): ν 3311 (NH_2_), 1513 (NH), 1549 (NO_2_). ^1^H-RMN (CDCl_3_): δ 0.01–0.05 (m, 2H, CH_2cpr_), 0.37–0.43 (m, 2H, CH_2cpr_), 0.79 (d, *J* = 7.0, 3H, CH_3_), 0.84–0.92 (m, 1H, CH_cpr_), 1.75 (br s, 1H, NH), 2.40–2.54 (m, 3H, H_6_, NHCH_2_CH), 2.78 (dd, *J* = 8.6, 1.8, 2H, 2H_3_), 2.77 (dd, *J* = 11.5, 10.5, 1H, H_1_), 3.15 (s, 2H, NHCH_2_), 4.86 (ddd, *J* = 11.6, 9.1, 7.2, 1H, H_2_), 5.56 (d, *J* = 1.1, 1H, H_5_), 6.87–6.92 (m, 2H, H_3′_, H_5′_), 7.05 (dd, *J* = 8.7, 5.3, 2H, H_2′_, H_6′_). ^13^C-RMN (CDCl_3_): δ 3.56 (CH_2cpr_), 3.57 (CH_2cpr_), 11.3 (CH_cpr_), 19.6 (CH_3_), 33.3 (C_3_), 37.5 (C_6_), 51.8 (C_1_), 54.5 (NHCH_2_CH), 54.7 (NHCH_2_), 88.6 (C_2_), 115.8 (d, *J* = 21.4, C_3′_, C_5′_), 128.2 (C_5_), 129.6 (C_2′_, C_6′_), 131.9 (C_4_)_,_ 134.4 (C_1′_), 162.3 (d, *J* = 246.0, C_4′_). HPLC (method A, t_R_, min): 14.90. MS (ESI, *m*/*z*, %): 318.9 ([M + H]^+^, 100).

### General Procedure D: Nitro Group Reduction with Zn/Acetic Acid

To a solution of the corresponding nitro derivative (1.00 equiv) in a 1:1 mixture of glacial acetic acid and anhydrous methanol (4 mL/mmol), Zn powder (10.0 equiv) was added and the reaction was stirred until complete conversion of starting material (1–3 h). Then, the mixture was filtered and washed with methanol, and the filtrate was evaporated. The residue was suspended in a sat. NaHCO_3_ solution and extracted with DCM (×3). The combined organic layers were washed with brine, dried over Na_2_SO_4_, filtered, and concentrated under reduced pressure. The residue was purified by flash chromatography to afford the corresponding final compound **1**, **13**–**17**, **18**–**32**, **85**–**87**, or intermediate **37**, **88**.

#### (1*R*,2*S*,6*R*)-4-{[(Cyclopropylmethyl)­amino]­methyl}-4′-methoxy-6-methyl-1,2,3,6-tetrahydro [1,1′-biphenyl]-2-amine, **1**

Following general procedure D using **35** (216 mg, 0.65 mmol), compound **1** was obtained as a yellow oil (176 mg, 90%, er >95:5). Chromatography: DCM to DCM/MeOH/NH_3_ 8:2:0.1. R_f_: 0.04 (DCM/MeOH/NH_3_ 9:1:0.1). [α]_20_^D^ = −12.2 (c = 0.99, CHCl_3_). ^1^H NMR signal in the diastereomer from chiral derivatization reaction: δ 6.01 ppm. IR (ATR): ν 3560 (NH_2_), 1513 (C–N), 1250 (COC). ^1^H NMR (CDCl_3_, 700 MHz): δ 0.11–0.13 (m, 2H, CH_2cpr_), 0.48–0.50 (m, 2H, CH_2cpr_), 0.81 (d, *J* = 7.0, 3H, CH_3_), 0.96–1.01 (m, 1H, CH_cpr_), 1.93–1.97 (m, 1H, H_1_), 2.00 (t, *J* = 10.4, 1H, H_3_), 2.35–2.38 (m, 2H, H_3_, H_6_), 2.46 (dd, *J* = 7.3, 2.5, 2H, NHCH_2_CH), 3.16 (td, *J* = 10.5, 5.2, 1H, H_2_), 3.21 (AB system, *J* = 13.8, 2H, NHCH_2_), 3.80 (s, 3H, OCH_3_), 5.47 (s, 1H, H_5_), 6.87 (d, *J* = 8.3, 2H, H_3′_, H_5′_), 7.11 (d, *J* = 8.3, 2H, H_2′_, H_6′_).^13^C NMR (CDCl_3_, 175 MHz): δ 3.5 (CH_2cpr_), 3.6 (CH_2cpr_), 11.4 (CH_cpr_), 20.2 (CH_3_), 37.0 (C_3_), 38.1 (C_6_), 51.9 (C_2_), 54.5 (NHCH_2_CH), 55.3 (NHCH_2_), 55.4 (OCH_3_), 57.4 (C_1_), 114.1 (C_3′_, C_5′_), 128.5 (C_5_), 129.4 (C_2′_, C_6′_), 133.7 (C_1′_), 134.7 (C_4_), 158.4 (C_4′_). 1D ^1^H NMR NOE: irradiation of the signal at δ 3.16 ppm (td, H_2_) yielded NOE on 7.11 (d, H_2′_, H_6′_). HPLC for HCl salt (method C, t_R_, min): 4.00 MS (ESI, *m*/*z*, %): 301.1 ([M + H]^+^, 100). Elemental analysis calculated for C_19_H_28_N_2_O·2HCl·2H_2_O: %C 55.74, %H 8.37, %N 6.84; experimental: %C 56.13, %H 7.99, %N 6.49.

#### (1*R*,2*S*,6*R*)-4-{[(Cyclopropylmethyl)­amino]­methyl}-4′-fluoro-6-methyl-1,2,3,6-tetrahydro­[1,1′-biphenyl]-2-amine, **25** (UCM-17017)

Following general procedure D using **77** (86 mg, 0.27 mmol), compound **25** was obtained as a yellow oil (55 mg, 71%, er >95:5). Chromatography: DCM to DCM/EtOH/NH_3_ 8:2:0.1. R_f_: 0.37 (DCM/MeOH/NH_3_ 9:1:0.1). [α]_20_^D^ = −11.0 (c = 1.0, CHCl_3_). IR (ATR): ν 3300 (NH_2_). ^1^H NMR (CDCl_3_, 500 MHz): δ 0.11–0.12 (m, 2H, CH_2cpr_), 0.46–0.48 (m, 2H, CH_2cpr_), 0.77 (d, *J* = 7.0, 3H, CH_3_), 0.94–0.98 (m, 1H, CH_cpr_), 1.84–1.98 (m, 4H, H_3_, NH, NH_2_), 2.04 (t, *J* = 10.4, 1H, H_1_), 2.31–2.40 (m, 2H, H_3_, H_6_), 2.45 (d, *J* = 6.8, 2H, NHCH_2_CH), 3.15 (td, *J* = 10.5, 5.2, 1H, H_2_), 3.20 (d, *J* = 5.1, 2H, NHCH_2_), 5.45 (s, 1H, H_5_), 6.99 (t, *J* = 8.4, 2H, H_3′_, H_5′_), 7.13 (dd, *J* = 8.4, 5.4, 2H, H_2′_, H_6′_). ^13^C NMR (CDCl_3_, 125 MHz): δ 3.5, 3.6 (2CH_2cpr_), 11.1 (CH_cpr_), 20.0 (CH_3_), 36.8 (C_3_), 38.1 (C_6_), 51.8 (C_2_), 54.3 (NHCH_2_CH), 55.0 (NHCH_2_), 57.3 (C_1_), 115.5 (d, *J* = 21.0, C_3′_, C_5′_), 128.6 (C_5_), 129.8 (d, *J* = 7.6, C_2′_, C_6′_), 133.4 (C_4_), 138.3 (d, *J* = 3.3, C_1′_), 161.7 (d, *J* = 244.4, C_4′_). HPLC for HCl salt (method C, t_R_, min): 4.77. MS (ESI, *m*/*z*, %): 289.2 ([M + H]^+^, 100). HRMS (ESI, *m*/*z*): calculated for C_18_H_26_FN_2_ [M + H]^+^: 289.2075, found: 289.2069. Elemental analysis calculated for C_18_H_25_FN_2_·2HCl·3H_2_O: %C 52.05, %H 8.01, %N 6.55; experimental: %C 52.39, %H 7.83, %N: 6.55.

### General Procedure E: Amidation Reaction

A solution of the appropriate carboxylic acid (1.00 equiv), HOBt (1.10 equiv), EDC (1.10 equiv) and DIPEA (1.10 equiv) in anhydrous DCM (5 mL/mmol) under nitrogen atmosphere was stirred at rt until complete consumption of starting material (40 min–3 h). Then, a solution of the corresponding amine (1.00 equiv) in anhydrous DCM (5 mL/mmol) was added at 0 °C and the reaction was stirred at this temperature for 3 h. Next, the mixture was diluted with EtOAc, and successively washed with water, a 1 M K_2_CO_3_ solution, and brine. The organic layer was dried over Na_2_SO_4_, filtered and concentrated under reduced pressure. The residue was purified by flash chromatography to afford the corresponding *N*-Fmoc amide derivative, intermediate **44**, or final compound **2**, **3**.

### General Procedure F: *N*-Fmoc Deprotection

To a solution of the corresponding *N*-Fmoc-protected amine (1.00 equiv) in anhydrous DCM (10 mL/mmol), piperidine (10.0 equiv) was added and the reaction was stirred at rt until complete consumption of starting material (8–16 h). The reaction was diluted with DCM and washed with water (×2). The organic layer was washed with brine, dried over Na_2_SO_4_, filtered and concentrated under reduced pressure. The residue was purified by flash chromatography to afford the corresponding final compound **4**–**7**.

### General Procedure G: *N*-Boc Protection

To a solution of the corresponding aminoderivative (1.00 equiv) and triethylamine in anhydrous DCM (7 mL/mmol) at 0 °C, a solution of di-*tert*-butyl dicarbonate (2 or 4 equiv) in DCM (3 mL/mmol) was added dropwise and the reaction mixture was stirred at 0 °C for 30 min and at rt until complete conversion of starting material (1–4 h). Then, the solvent was removed under reduced pressure and the residue was purified by flash chromatography to afford the corresponding *N*-Boc intermediate **38**, **95**, **99**, **100**.

#### *tert*-Butyl ({(1*R*,2*S*,6*R*)-2-[(*tert*-Butoxycarbonyl)­amino]-4′-fluoro-6-methyl-1,2,3,6-tetrahydro­[1,1′-biphenyl]-4-yl}­methyl)­methylcarbamate, **100**

Following general procedure G using **85** (130 mg, 0.52 mmol), triethylamine (0.2 mL, 1.36 mmol) and di-*tert*-butyl dicarbonate (457 mg, 2.08 mmol), compound **100** was obtained as a colorless oil (196 mg, 84%). Chromatography: hexane to hexane/EtOAc 7:3. R_f_: 0.70 (hexane/EtOAc 7:3). [α]_20_^D^ = −19.0 (c = 1.0, CHCl_3_). IR (ATR): ν 3362 (NH), 1696 (C=O), 1511 (C–N). ^1^H NMR (CDCl_3_, mixture of rotamers): δ 0.82 (d, *J* = 7.0, 3H, CH_3_), 1.24 (s, 9H, 3CH_3_), 1.46 (s, 9H, 3CH_3_), 1.85–1.97 (m, 1H, H_3_), 2.21 (t, *J* = 8.0, 1H, H_1_), 2.44 (br s, 2H, H_3_, H_6_), 2.81 (br s, 3H, NCH_3_), 3.75 and 3.96 (br s, 2H, NCH_2_), 4.13 (br s, 1H, H_2_), 5.37 (s, 1H, H_5_), 6.98 (t, *J* = 8.7, 2H, H_3′_, H_5′_), 7.14 (dd, *J* = 8.5, 5.5, 2H, H_2′_, H_6′_). ^13^C NMR (CDCl_3_): δ 20.2 (CH_3_), 28.3 (3CH_3_), 28.6 (3CH_3_), 33.7 (NCH_3_), 34.7 (br, C_3_), 38.6 (C_6_), 51.1 (br, C_2_), 53.3 and 54.3 (br, NCH_2_), 54.6 (br, C_1_), 79.2 (C­(CH_3_)_3_), 79.7 (C­(CH_3_)_3_), 115.2 (d, *J* = 21.4, C_3′_, C_5′_), 129.0 (br, C_5_), 130.1 (d, *J* = 7.8, C_2′_, C_6′_), 131.5 (C_4_), 137.2 (br, C_1′_), 155.2 (CO), 156.2 (CO), 161.9 (d, *J* = 244.1, C_4′_). HPLC (method B, t_R_, min): 12.36. MS (ESI, *m*/*z*, %): 349.2 ([M-Boc]^+^, 100).

### General Procedure H: *N*-Methylation

To a solution of the corresponding amine (1.00 equiv) in anhydrous DMF (7 mL/mmol) at 0 °C, NaH (4.00 equiv) was added and the reaction was stirred for 1 h. Then, iodomethane (10.0 equiv) was added and the mixture was stirred at rt overnight. The reaction was quenched with a sat. NaHCO_3_ solution and extracted with EtOAc (×2). The organic layers were washed with brine, dried over Na_2_SO_4_, filtered and evaporated under reduced pressure. The residue was purified by flash chromatography to afford the corresponding *N*-methyl derivative **101**, **102**.

#### *tert*-Butyl [(1*R*,2*S*,6*R*)-4-{[(*tert*-Butoxycarbonyl)­(cyclopropylmethyl)­amino]­methyl}-4′-fluoro-6-{[(pyridin-2-yl)­methoxy]­methyl}-1,2,3,6-tetrahydro­[1,1′-biphenyl]-2-yl]­methylcarbamate, **102**

Following general procedure H, using **100** (196 mg, 0.44 mmol), compound **102** was obtained as a colorless oil (157 mg, 78%). Chromatography: hexane to hexane/EtOAc 7:3. R_f_: 0.30 (hexane/EtOAc 7:3). [α]_20_^D^ = −19.0 (c = 1.0, CHCl_3_). IR (ATR): ν 1691 (C=O), 1511 (C–N). ^1^H NMR (CDCl_3_, mixture of rotamers): δ 0.81 (d, *J* = 6.9, 3H, CH_3_), 1.26 (s, 9H, 3CH_3_), 1.46 (s, 9H, 3CH_3_), 1.98–2.09 (m, 1H, H_3_), 2.10–2.23 (m, 1H, H_3_), 2.25–2.40 (m, 2H, H_1_, H_6_), 2.43 and 2.51 (s, 3H, CHNCH_3_), 2.82 (br s, 3H, CH_2_NCH_3_), 3.61–4.00 (br m, 2H, NCH_2_), 4.49–4.58 and 4.74–4.84 (m, 1H, H_2_), 5.34 (s, 1H, H_5_), 6.93–6.97 (m, 2H, H_3′_, H_5′_), 7.06–7.11 and 7.14–7.17 (m, 2H, H_2′_, H_6′_). ^13^C NMR (CDCl_3_): δ 20.1 (CH_3_), 27.3 and 27.9 (CHNCH_3_), 28.4 (3CH_3_), 28.6 (3CH_3_), 29.8 and 30.1 (C_3_), 33.7 and 33.8 (CH_2_NCH_3_), 39.26 and 39.33 (C_6_), 51.7 (C_1_), 53.5 and 54.4 (NCH_2_), 53.7 and 55.0 (C_2_), 79.6 (C­(CH_3_)_3_), 79.7 (C­(CH_3_)_3_), 114.9 and 115.2 (d, *J* = 21.2, C_3′_, C_5′_), 129.0 (br, C_5_), 129.6 and 130.0 (C_2′_, C_6′_), 131.8 (br, C_4_), 137.1 (br, C_1′_), 155.4 and 155.9 (CO), 156.2 (br, CO), 161.7 (d, *J* = 243.6, C_4′_). HPLC (method B, t_R_, min): 16.90. MS (ESI, *m*/*z*, %): 363.3 ([M-Boc]^+^, 100).

### General Procedure I: *N*-Boc Deprotection

A solution of the corresponding *N*-Boc-protected amine (1.00 equiv) in HCl (4 M in dioxane, 15.0 equiv) was stirred at rt until complete consumption of starting material (4–16 h). Solvent was then evaporated under reduced pressure and the corresponding final compound **8**–**12**, **89**, **97**, **98** was purified using a SiliCycle SCX-2 cartridge and elution with 2 M NH_3_ in methanol according to the manufacturer procedure.

#### (1*R*,2*S*,6*R*)-4′-Fluoro-*N*,6-dimethyl-4-[(methylamino)­methyl]-1,2,3,6-tetrahydro­[1,1′-biphenyl]-2-amine, **98** (UCM-17221)

Following general procedure I using **102** (160 mg, 0.32 mmol), compound **98** was obtained as a colorless oil (82 mg, 90%, er >95:5). R_f_: 0.30 (DCM/MeOH/NH_3_ 8:2:0.1). [α]_20_^D^ = 6.0 (c = 1.00, MeOH). IR (ATR): ν 3328 (NH), 1508 (C–N). ^1^H NMR (CDCl_3_): δ 0.78 (d, *J* = 6.9, 3H, CH_3_), 1.85–2.00 (m, 1H, H_3_), 2.17–2.29 (m, 6H, CHNHCH_3_, H_1_, 2NH), 2.30–2.39 (m, 1H, H_6_), 2.44 (s, 3H, CH_2_NHCH_3_), 2.51 (dd, *J* = 16.5, 5.0, 1H, H_3_), 2.86 (td, *J* = 10.3, 5.2, 1H, H_2_), 3.20 (s, 2H, NHCH_2_), 5.50 (s, 1H, H_5_), 7.00 (t, *J* = 8.7, 2H, H_3′_, H_5′_), 7.16 (dd, *J* = 8.7, 5.4, 2H, H_2′_, H_6′_). ^13^C NMR (CDCl_3_): δ 20.0 (CH_3_), 33.6 (C_3_), 33.9 (CHNHCH_3_), 35.5 (CH_2_NHCH_3_), 38.1 (C_6_), 54.7­(C_1_), 57.3 (NHCH_2_), 59.5 (C_2_), 115.7 (d, *J* = 22.0, C_3′_, C_5′_), 129.5 (C_5_), 129.8 (d, *J* = 7.7, C_2′_, C_6′_), 132.4 (C_4_), 137.9 (d, *J* = 3.3, C_1′_), 161.9 (d, *J* = 244.7, C_4′_). HPLC for HCl salt (method C, t_R_, min): 4.77. MS (ESI, *m*/*z*, %): 263.1 ([M + H]^+^, 100). HRMS (ESI, *m*/*z*): calculated for C_16_H_24_FN_2_ [M + H]^+^: 263.1918; found: 263.1913. Elemental analysis calculated for C_16_H_23_FN_2_·2HCl·2H_2_O: %C 51.76, %H 7.87, %N 7.54; experimental: %C 51.92, %H 7.51, %N 7.32.

### *In Vitro* Assays

#### Cell Lines and Culture

Fibroblast cell line (IMR-90) was obtained from American Type Culture Collection (ATCC; reference number CCL-186) and maintained in Dulbecco’s modified Eagle medium (DMEM, Gibco) supplemented with 15% heat-inactivated fetal bovine serum (FBS), 10 U/mL penicillin, and 10 μg/mL streptomycin. A549 cells were cultured in high-glucose DMEM (4,500 mg·L^–1^; Sigma-Aldrich), supplemented with 10% FBS (Corning), 1% penicillin/streptomycin (Sigma-Aldrich, P4333), and 1% glutamine (Sigma-Aldrich, G8540), and were routinely tested for mycoplasma contamination. Cells were incubated in a humidified atmosphere at 37 °C in the presence of 5% CO_2_.

#### FDG SA-β-gal Senescence Assay

IMR-90 cells (2 million) were seeded in a P-100 Petri dish with DMEM and left to settle for 24 h. Then, DMEM was replaced by DMEM containing 300 μM H_2_O_2_. After 2 h, the medium was replaced by fresh DMEM without H_2_O_2_ and the cells were incubated for 4 days. Next, the cells were split in a 1:2 ratio and incubated for 24 h, followed by a second treatment with DMEM containing 300 μM H_2_O_2_ for 2 h. Finally, the cells were incubated in fresh DMEM without H_2_O_2_ for 2–4 days. The senescence phenotype was confirmed both by monitoring the characteristic morphological changes as enlarged and flattered cells with bigger nuclei, and in the FDG SA-β-gal assay.

Senescent IMR-90 cells (6·10^3^ cells per well) were cultured in DMEM in a 96-well plate overnight for attachment. Then, the medium was replaced by fresh DMEM containing tested compound or the equivalent volume of DMSO as vehicle. After 48 h, the cells were fixed for 5 min in 2% formaldehyde and 0.2% glutaraldehyde buffered with PBS, and then incubated with 95 μL of staining solution (0.2 M citric acid, 0.4 M Na_2_HPO_4_, 100 mM potassium ferrocyanide, 100 mM potassium ferricyanide, 5 M NaCl, and 0.2 M MgCl_2_ in water) and 5 μL of 2 mM FDG (Sigma-Aldrich) for 24 h at 37 °C without CO_2_. Then, an equal volume of the supernatant of each well was transferred to a new 96-well plate for fluorescence measurement. Fluorescein fluorescence was registered at 520 nm using a FluoStar Optima instrument (BMG Labtech), with an excitation wavelength of 485 nm. One well containing the reaction mixture without cells was used as blank for subtracting the background fluorescence. For vehicle-treated cells, the higher fluorescence compared to cells not treated with H_2_O_2_ confirmed the senescence phenotype and was assigned a value of 100% SA-β-gal activity. For compound-treated cells, the SA-β-gal activity was expressed as a fluorescence percentage relative to the vehicle, obtained from at least three experiments performed in triplicate.

For the concentration–response curves, senescent IMR-90 cells were treated with increasing concentrations of the tested compound. IC_50_ values (*n* = 3) were determined by nonlinear regression analysis using Prism Software (GraphPad, v.10.4.2).

#### MTT Viability Assay

IMR-90 cells were seeded in 96-well plates (10·10^3^ cells per well) in DMEM with 15% FBS for 24 h prior to treatments. The medium was then replaced by fresh medium containing tested compound or the equivalent volume of DMSO as vehicle. After 48 h, the medium was replaced by fresh DMEM with 5 mg/mL of MTT (Sigma-Aldrich), and the cells were incubated for 4 h at 37 °C in the dark. The supernatants were removed, formazan crystals were dissolved in DMSO (100 μL/well), and the absorbance was measured at 570 nm (OD570–630) using an Asys UVM 340 microplate reader (Biochrom Ltd., Cambridge, U.K.). The background absorbance of blank wells containing only medium with compound or vehicle were subtracted from each test well. The results were reported as cell viability percentage for tested compound relative to the vehicle, obtained from at least two experiments performed in triplicate.

Proliferative A549 cells were treated with 20 μM bleomycin (Mylan Pharmaceuticals) for 5 days as a chemotherapeutic agent to induce senescence. Proliferative and senescent A549 cells were treated with the tested compounds for 24, 48, or 72 h and cell viability was determined by MTT assay as above. ABT-263 (Abbvie) was used as a positive control of senolytic compound.

#### HSA Binding Assay

The compounds were incubated with different concentrations of immobilized HSA, using the TRANSILXL HSA Binding Kit (TMP-0210–2096, Sovicell). An 8-well unit of the TRANSIL assay plate was used for each tested compound; six wells contained increasing concentrations of HSA immobilized on silica beads suspended in PBS at pH 7.4, and two wells contained buffer only to account for nonspecific binding. The TRANSIL assay plate was thawed for 3 h at rt and centrifuged at 750*g* for 5 s. Then, 15 μL of an 80 μM stock solution of the compound in PBS (for a final concentration of 5 μM) was added to each well, and the plate was incubated on a shaker at 1000 rpm and rt for 12 min. Then the plate was centrifuged at 750*g* for 10 min, and 50 μL of the supernatants were transferred for analytical quantification by HPLC-MS using selected ion monitoring (SIM) as acquisition method. The binding percentage was calculated from the remaining free compound concentration in the supernatant of each well, using the spreadsheet and algorithms supplied with the kit.

#### Stability in Human and Mouse Sera

An aliquot of 625 μL of a 250 μM solution of tested compound in PBS (pH 7.4) was added to 1.875 mL of mouse (Europa Bioproducts, EQSM-0100) or human serum (Sigma-Aldrich) prewarmed at 37 °C. Next, the solution was incubated at 37 °C for 4 h, taking aliquots of 250 μL at different times (0, 1, 2, 4, 8, 24, and 48 h). Each aliquot was quenched in 375 μL of cold ACN, vortexed, incubated for 10 min in ice and centrifuged at 39,000*g* for 10 min. Supernatants were then analyzed by HPLC-MS using SIM mode, and quantification was estimated by using the peak area integration normalized with an internal standard.

#### Stability Assays in Mouse and Human Liver Microsomes

Tested compound was incubated at 37 °C in 1 μM PBS with NADPH (final concentration of 2 mM) and MgCl_2_ (final concentration of 5 mM). Metabolic reactions were initiated by the addition of a suspension of MLMs (male CD-1 mice pooled, Sigma-Aldrich) or HLMs (male human pooled, Sigma-Aldrich), at a final protein concentration of 1 mg/mL. The solutions were shaken in a vortex and kept at 37 °C in a water bath open to the air. Aliquots of 100 μL were quenched at time zero and at seven points ranging to 2 h (MLM) or 8 h (HLM) by pouring into 100 μL of ice-cold ACN. Quenched samples were centrifuged at 10,000*g* for 5 min, and the supernatants were filtered through a poly­(tetrafluoroethylene) membrane syringe filter (pore size of 0.2 μm, Albet Labscience). The relative loss of parent compound over the course of the incubation was monitored by HPLC-MS using SIM mode. Concentrations were quantified by measuring the area under the peak ([M + H]^+^) and converted to the percentage of remaining compound, using the time zero peak area value as 100%. The natural logarithm of the percentage remaining versus time data for each compound was fitted to linear regression, and the slope was used to calculate the degradation half-life time (*t*_1/2_).

#### Parallel Artificial Membrane Permeability Assay (PAMPA)

Prior to use, the commercially available 96-well Corning Gentest precoated PAMPA plate system (Cultek S.L.U., Spain) was warmed to rt for 30 min. Then, 300 μL of 200 μM solution of tested compound in 2% DMSO in PBS were added into wells in the donor plate, and 200 μL of PBS were added into wells in the acceptor plate. The acceptor plate was placed on the donor plate by lowering the precoated PAMPA plate. The assembly was incubated at rt for 5 h, and then buffer samples were collected from each plate. The final concentrations of compound in both donor and acceptor wells were analyzed by HPLC-MS using SIM mode and quantification was estimated by using the peak area integration normalized with an internal standard. Permeability values of tested compounds and propranolol and metoprolol as reference compounds were calculated using the following formula: *P* (cm/s) = {−ln­[1 – *C*_A_(*t*)/*C*_eq_]}/[*A* × (1/*V*_D_ + 1/*V*_A_) × *t*], where *A* = filter area (0.3 cm^2^), *V*_D_ = donor well volume (0.3 mL), *V*_A_ = acceptor well volume (0.2 mL), *t* = incubation time (s), *C*_A_(*t*) = compound concentration (μM) in acceptor well at time *t*, *C*_D_(*t*) = compound concentration (μM) in donor well at time *t*, and *C*_eq_ = [*C*_D_(*t*) × *V*_D_ + *C*_A_(*t*) × *V*_A_]/(*V*_D_ + *V*_A_).

#### hERG Assay

Interaction of the compounds with the hERG potassium channel was evaluated by Fundación Medina using the FluxOR potassium channel assay, using hERG-HEK293 cell line that stably expresses at passage 18. The assay was performed as outlined in the Invitrogen information sheet and performed on the FLIPR TETRA (Molecular Devices). Powerload concentrate and water-soluble probenecid were added in the first step, followed by FluxOR. Loading buffer was 165 mM NaCl, 4.5 mM KCl, 2 mM CaCl_2_, 1 mM MgCl, 10 mM Hepes and 10 mM glucose, adjusted to pH 7.2. Media were removed from the cell plates and 50 μL of loading buffer containing the FluxOR dye mix was applied to each for 60 min at rt, then removed manually. The cell plates were subsequently washed once with assay buffer, before adding the samples at 100 μM a final volume of 50 μL of assay buffer. Plates were incubated at rt for 30 min to allow equilibration of the test compounds. Stimulation buffer (Tl_2_SO_4_ + K_2_SO_4_) was prepared following the manufacturer instruction. The injection of stimulation buffer into the plates was performed on the FLIPR TETRA, and the kinetic data obtained for 8 points from time 0 to 120 s were analyzed using *Genedata Screener*. Positive control (20 μM astemizole) and negative control (1% DMSO) were introduced.

### *In Vivo* Experiments

Animal procedures were approved by the Bioethics Committee of Santiago de Compostela University in compliance with the Principles of Laboratory Animal Care of national laws (license number 15012/2022/020).

#### Pharmacokinetics

Tested compound was administered (40 mg/kg, i.p.) and blood was collected by cardiac puncture at the selected time points postdose (*n* = 2 per time point). Blood was allowed to clot at rt for 20 min and centrifuged at 4 °C for 10 min at 10,000*g*. The supernatant was transferred to a clean polypropylene tube and stored at −80 °C until analysis. For analysis, a volume of cold ACN was added to the serum. The sample was incubated in an ice bath for 10 min and centrifuged at 4 °C for 10 min at 18,000*g*. The resulting organic layer was filtered through a poly­(tetrafluoroethylene) filter (0.2 μM, 13 mm diameter, Fisher Scientific) and 20 μL of the sample were analyzed by LC-MS/MS (UCM’s mass spectrometry facilities). Separation was performed using an Agilent Zorbax Rx-SIL column (C18, 5 μm 80 Å 250 × 4.6 mm), with a run time of 8 min and a flow of 0.4 mL/min (gradient: 3 min 5% to 35% B; 5 min 35% to 100% B; 8 min 5% B; phase A: water with formic acid 0.1%; phase B: ACN). The entire eluent was directly introduced to an electrospray ionization source operating in the positive ion mode in a Shimadzu LCMS8030 triple quadrupole mass spectrometer coupled to UHPLC with an oven temperature of 31.5 °C. The mass spectrometer ion optics were set in the multiple reaction monitoring mode and the transition selected for quantification was set in the molecular weight range. Concentrations were quantified by measuring the area under the peak (AUC) registered for the compound ([M + H]^+^) in each sample. The natural logarithm of the concentration versus time data for each compound was fitted to linear regression, and the slope was used to calculate the elimination rate constant (*k*_e_).

#### Pulmonary Fibrosis Model

Male C57BL/6J 10 weeks old mice were intratracheally instilled with bleomycin (1 IU/kg, Mylan Pharmaceuticals) under anesthesia. After 2 weeks, animals were treated with the tested compound (i.p. at 40 mg/kg) or ABT-263 (AbbVie, p.o. at 50 mg/kg) daily for 1 week. At the end of the experiment, animals were sacrificed and lungs were removed for analysis.

#### Tissue Staining

SA**-**β**-**gal staining was performed following well-established methods.^44^ Briefly, whole mount lungs were fixed at rt in 2% formaldehyde/0.2% glutaraldehyde, washed, and incubated overnight at 37 °C with fresh SA-β-gal staining solution: 1 mg of 5-bromo-4-chloro-3-indolyl β-d-galactoside (X-gal) per mL (Fisher Scientific), 40 mM citric acid/sodium phosphate (pH 5.6), 5 mM K_3_Fe­[CN]_6_, 5 mM K4Fe­[CN]_6_, 150 mM NaCl, and 2 mM MgCl_2_. Tissues were then embedded in paraffin, sectioned, and counterstained with Nuclear Fast Red.

Masson trichrome staining was performed by the Histopathology Unit at the Clinical University Hospital of Santiago de Compostela following standard protocols.^58^

Stained tissue sections were visualized, and pictures were taken with AxioVert.A1Microscope (Zeiss). The images were analyzed with Zen Blue Edition software (Zeiss) and ImageJ (FIJI) for the quantification of the positive area for each marker.

#### Q-RT-PCR

To assess gene expression, total RNA was isolated from lung tissues using the NucleoSpin RNA Kit (Macherey-Nagel) as per the manufacturer’s guidelines and converted to cDNA through High-Capacity cDNA Reverse Transcription (Applied Biosystems). For quantification, we utilized the NZYSpeedy qPCR Green Master Mix reagent (2X), ROX (NZYTech), and the AriaMx Real-Time PCR systems thermocycler (Agilent Technologies). Each reaction included 33 ng of cDNA, oligonucleotides at a final concentration of 0.25 μM, 5 μL SYBR, and nuclease-free water, resulting in a final volume of 10 μL per reaction. GAPDH served as the housekeeping gene, and the gene expression levels were normalized to its expression. Triplicate analyses were conducted for each sample. The results were evaluated with the AriaMx 1.0 software (Agilent Technologies), and the primers (Table 4) were sourced from Eurofins Genomics.

**Table 4:** Primers for Q-RT-PCR

*Gapdh*	5′-TCCATGACAACTTTGGCATCGTGG-3′
	5′-GTTGCTGTTGAAGTCACAGGAGAC-3′
*Cdkn1a*	5′-GTGGGTCTGACTCCAGCCC-3′
	5′-CCTTCTCGTGAGACGCTTAC-3′
*Col1a1*	5′- TTCTCCTGGCAAAGACGGACTCAA-3′
	5′- AGGAAGCTGAAGTCATAACCGCCA-3′
*Ccl2*	5′- CATCCACGTGTTGGCTCA-3′
	5′- GATCATCTTGCTGGTGAATGAGT-3′

#### Statistics

Statistical analysis was conducted using Prism Software (GraphPad, v.10.4.2). Data are presented as mean ± SD unless indicated otherwise. Group allocation occurred randomly. For *in vitro* data that are normally distributed with equal variance, we assessed statistical significance using a two-tailed unpaired Student’s *t* test. For *in vivo* data, the nonparametric Mann–Whitney test was used.

## Supplementary Material





## Notes

Tables S1 and S2, Figures S1–S3, synthesis and characterization data of final compounds **2**–**24**, **26**–**33**, **85**–**89**, and **97**, and intermediates **36**–**61**, **63**–**76**, **78**–**84**, **90**–**96**, **99**, **101**, NMR, HPLC-MS, and HRMS spectra of final compounds **25** and **98** (PDF)
Molecular formula strings (CSV)

## Abbreviations Used

ACN: acetonitrile
ATR: attenuated total reflection
br: broad
CL: clearance
cpr: cyclopropane
DIPEA: *N*,*N*-diisopropylethylamine
DMEM: Dulbecco’s modified Eagle’s medium
dr: diastereoisomeric ratio
EDC: *N*-(3-(dimethylamino)­propyl)-*N*′-ethylcarbodiimide
FBS: fetal bovine serum
FDG: fluorescein-di-β-galactopyranoside
HLM: human liver microsomes
ind: indole
*k*_e_: elimination rate constant
MLM: mouse liver microsomes
morp: morpholine
MTT: 3-(4,5-dimethylthiazol-2-yl)-2,5-diphenyl-2*H*-tetrazolium bromide
nd: not determined
ox: oxazole
*P*: permeability value
py: pyridine
quint: quintuplet
SA-β-gal: senescence-associated β-galactosidase
SASP: senescence-associated secretory phenotype
SEM: standard error of the mean
SIM: selected ion monitoring
thz: thiazole
UCM: Universidad Complutense de Madrid
*V*_D_: distribution volume
