# Supplementary material for: Discovery of Novel Synthetic Cyclohexene-Based Small Molecules Targeting Senescence against Age-Related Pulmonary Fibrosis
Source: J Med Chem. 2026 Jul 10;69(14):16765–83. doi: 10.1021/acs.jmedchem.6c00412 (PMC13403236; doi:10.1021/acs.jmedchem.6c00412)
Supplement: Supplementary file 1 [file jm6c00412_si_001.pdf]

# Supporting Information

# Discovery of novel synthetic cyclohexene-based small molecules targeting senescence against age-related pulmonary fibrosis

*Iván Arribas-Álvarez,[a] Sergio Algar,[a] Pilar Picallos-Rabina,[b] Anabel Sánchez-Merino,[a] Beatriz Marcos-Ramiro,[a] Manuel Collado,[b,c] Henar Vázquez-Villa,[a] María L. López-Rodríguez*,[a] and Bellinda Benhamú*,[a]*

[a] Department of Organic Chemistry, Faculty of Chemistry, Universidad Complutense de Madrid, E-28040 Madrid, Spain

[b] Laboratory of Cell Senescence, Cancer and Aging, Center for Research in Molecular Medicine and Chronic Diseases (CiMUS), University of Santiago de Compostela, Health Research Institute of Santiago de Compostela (IDIS), E-15706 Santiago de Compostela, Spain.

[c] Department of Immunology and Oncology, National Centre for Biotechnology (CNB-CSIC), E-28049 Madrid, Spain.

bellinda.benhamu@quim.ucm.es, mluzlr@ucm.es

**Table of Contents**

1. Table S1 and Figures S1 and S2 — S2
2. Experimental methods — S5
   2.1. Table S2 and Figure S3 — S5
   2.2. Synthesis of final compounds **2** and **3** — S6
   2.3. Synthesis of final compounds **4-7** — S7
   2.4. Synthesis of final compounds **8-12** — S13
   2.5. Synthesis of final compounds **13-17** — S24
   2.6. Synthesis of final compounds **18-24, 26-33** — S35
   2.7. Synthesis of final compounds **85-89** — S64
   2.8. Synthesis of final compound **97** — S72
3. NMR, HPLC-MS and HRMS spectra of final compounds **25** and **98** — S75

## 1. Table S1 and Figures S1 and S2

**Table S1.** Senotherapeutic activity of compounds **S1-S9** in the FDG SA-β-gal assay.

| compd | Structure | SA-β-gal act. (%, @10 μM)[a] | SA-β-gal act. (%, @1 μM)[a] |
|---|---|---|---|
| **S1** | 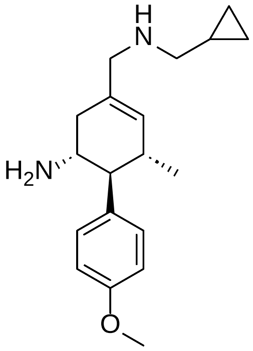 | 45±3 | 48±4 |
| **S2** | 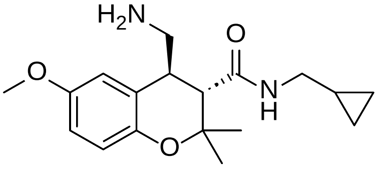 | 43±3 | 55±2 |
| **S3** | 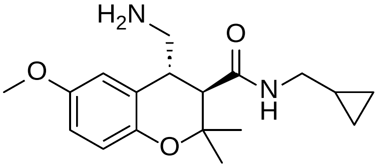 | 25±11 | 54±2 |
| **S4** | 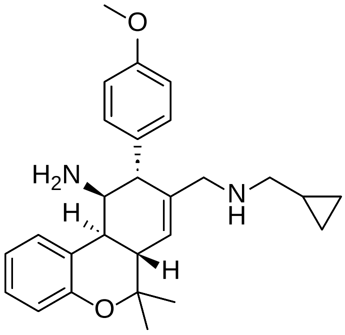 | 0 | 52±5 |
| **S5** | 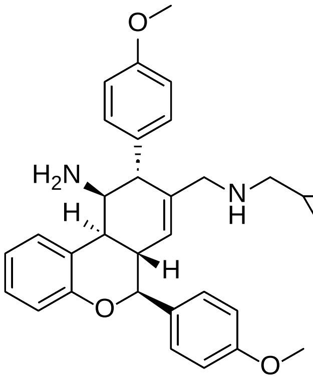 | 0 | 55±1 |

| | | | |
|---|---|---|---|
| **S6** | 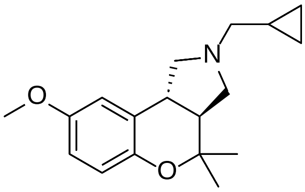 | 42±15 | 49±0 |
| **S7** | 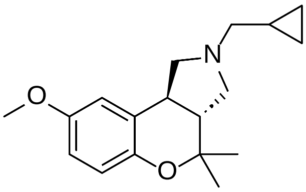 | 41±5 | 53±9 |
| **S8** | 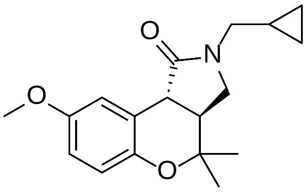 | 10±5 | 54±13 |
| **S9** | 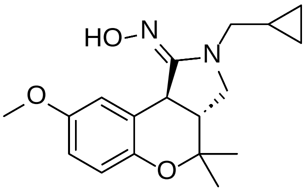 | 31±3 | 51±7 |

[a] Values are the mean±SEM of two independent experiments with triplicate determinations.

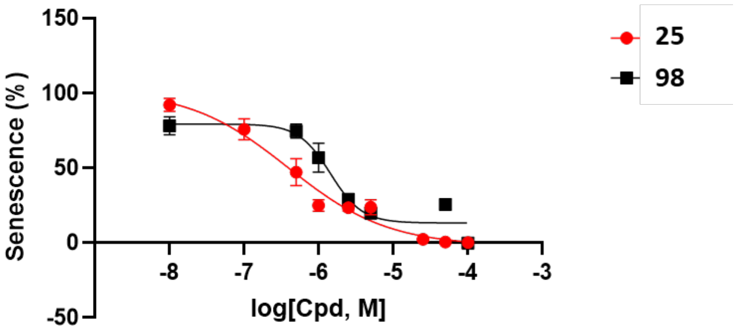


**Figure S1**. Concentration-response curves of compounds **25** and **98** for SA-β-gal inhibition in senescent fibroblasts IMR90. Data obtained from three separated experiments performed in triplicate.

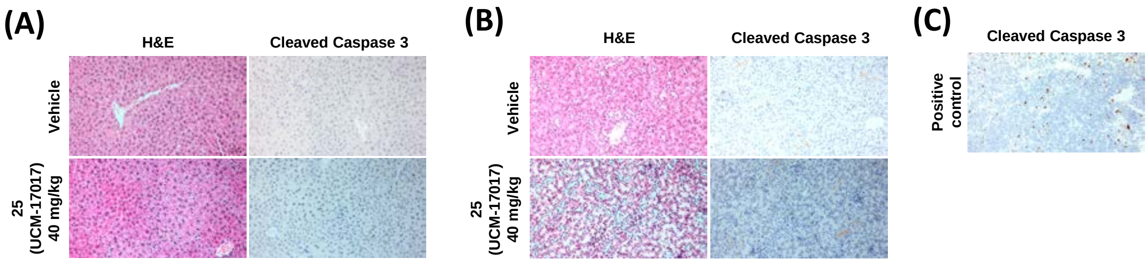


**Figure S2**. IHC analysis of apoptotic cells in a representative sample of (A) liver and (B) kidney from bleomycin-instilled mice treated with DMSO (vehicle, top) vs compound **25** (40 mg/kg, bottom): H&E (left) and cleaved caspase-3 (right). (C) Mouse thymus was used as positive control for cleaved caspase-3 staining. Scale bar: 50 µm.

## 2. Experimental methods

For the general considerations regarding the synthesis of the compounds, see the Experimental Section of the paper.

The general synthetic procedures A-I mentioned along this section are described in the Experimental Section of the manuscript.

### 2.1. Table S2 and Figure S3.

**Table S2.** Gradients of the mobile phases used in the HPLC-MS analysis of the synthetized compounds.

| **Method A** (Column XDB-C18) | | **Method B** (Column SB-C3) | | **Method C** (Column XDB-C18) | |
|---|---|---|---|---|---|
| **t (min)** | **% ACN** | **t (min)** | **% ACN** | **t (min)** | **% ACN** |
| 0 | 0 | 0 | 0 | 0 | 0 |
| 2 | 0 | 2 | 0 | 1 | 0 |
| 8 | 50 | 8 | 80 | 4 | 50 |
| 14 | 100 | 10 | 100 | 7 | 100 |
| 18 | 100 | 18 | 100 | 9 | 100 |
| 22 | 0 | 22 | 0 | 13 | 0 |
| 25 | 0 | 25 | 0 | 15 | 0 |

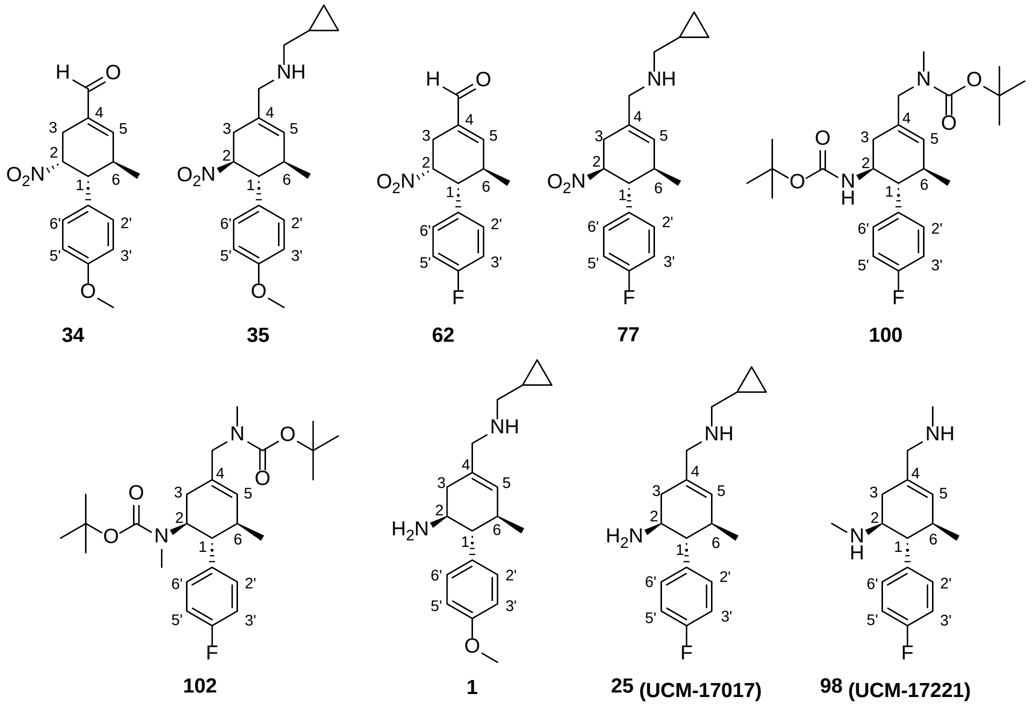


**Figure S3.** Numbered chemical structures for NMR assignation of compounds **34**, **35**, **62**, **77**, **100**, **102**, **1**, **25 (UCM-17017)**, and **98 (UCM-17221)**.

## 2.2. Synthesis of final compounds 2 and 3 (Scheme 1)

***N*-[(1*R*,2*S*,6*R*)-4-{[(Cyclopropylmethyl)amino]methyl}-4'-methoxy-6-methyl-1,2,3,6-tetrahydro[1,1'-biphenyl]-2-yl]pentanamide, 2.** Following general procedure E using **1** (225 mg, 0.75 mmol) and valeric acid (81 µL, 0.75 mmol), compound **2** was obtained as a yellow oil (42 mg, 18%). Chromatography: DCM to DCM/MeOH/$NH_3$ 9.5:0.5:0.1.

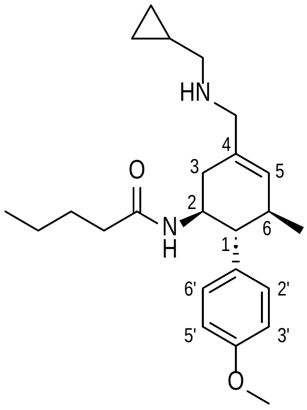


$R_f$: 0.34 (DCM/MeOH/$NH_3$ 9:1:0.1). $[\alpha]^D_{20}$ = -26.5 (c = 0.77, $CHCl_3$). IR (ATR): ν 3282 (NH), 1640 (C=O), 1513 (C-N), 1248 (COC). $^1$H-NMR ($CDCl_3$): δ 0.10-0.15 (m, 2H, $CH_{2cpr}$), 0.46-0.52 (m, 2H, $CH_{2cpr}$), 0.73 (t, *J* = 7.2, 3H, C<u>H</u>$_3$CH$_2$), 0.83 (d, *J* = 6.9, 3H, $CH_3$), 0.89-1.04 (m, 3H, $CH_{cpr}$, CH$_3$C<u>H</u>$_2$), 1.19-1.30 (m, 2H, CH$_2$C<u>H</u>$_2$CH$_2$), 1.76-1.98 (m, 2H, NHCOC<u>H</u>$_2$), 1.96-2.03 (m, 1H, $H_3$), 2.15-2.23 (m, 1H, $H_1$), 2.37-2.47 (m, 1H, $H_6$), 2.45 (d, *J* = 6.8, 2H, NHC<u>H</u>$_2$CH), 2.59 (dd, *J* = 16.9, 5.4, 1H, $H_3$), 3.19 (s, 2H, NHC<u>H</u>$_2$), 3.78 (s, 3H, $OCH_3$), 4.27-4.39 (m, 1H, $H_2$), 4.99 (d, *J* = 8.2, 1H, NHCO), 5.47 (s, 1H, $H_5$), 6.83 (d, *J* = 8.6, 2H, $H_{3'}$, $H_{5'}$), 7.07 (d, *J* = 8.6, 2H, $H_{2'}$, $H_{6'}$). $^{13}$C-NMR ($CDCl_3$): δ 3.5 ($CH_{2cpr}$), 3.6 ($CH_{2cpr}$), 11.4 ($CH_{cpr}$), 13.9 (<u>C</u>H$_3$CH$_2$), 20.2 ($CH_3$), 22.1 (CH$_3$<u>C</u>H$_2$), 27.9 (CH$_2$<u>C</u>H$_2$CH$_2$), 35.4 ($C_3$), 36.8 (NHCO<u>C</u>H$_2$), 38.9 ($C_6$), 49.8 ($C_2$), 53.9 ($C_1$), 54.6 (NH<u>C</u>H$_2$CH), 55.2 ($NHCH_2$), 55.4 ($OCH_3$), 113.9 ($C_{3'}$, $C_{5'}$), 128.2 ($C_5$), 129.3 ($C_{2'}$, $C_{6'}$), 133.6 ($C_{1'}$), 133.7 ($C_4$), 158.5 ($C_{4'}$), 172.6 (NHCO). HPLC (method A, $t_R$, min): 15.54. MS (ESI, *m/z*, %): 385.2 ($[M+H]^+$, 100). Elemental analysis calculated for $C_{24}H_{36}N_2O_2$·HCl·$H_2O$: %C 65.66, %H 8.95, %N 6.38; experimental: %C 65.28, %H 8.59, %N 6.25.

***N*-[(1*R*,2*S*,6*R*)-4-{[(Cyclopropylmethyl)amino]methyl}-4'-methoxy-6-methyl-1,2,3,6-tetrahydro[1,1'-biphenyl]-2-yl]pyridine-3-carboxamide, 3.** Following general procedure E using **1** (100 mg, 0.33 mmol) and nicotinic acid (41 mg, 0.33 mmol),

compound **3** was obtained as a yellow oil (49 mg, 36%). Chromatography: DCM to DCM/MeOH/$NH_3$ 9.5:0.5:0.1.

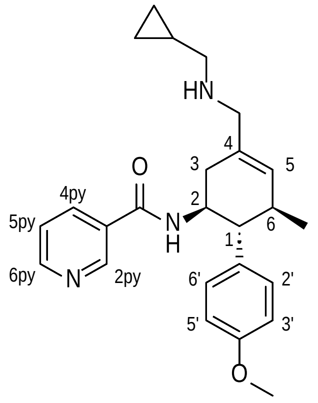


$R_f$: 0.29 (DCM/MeOH/$NH_3$ 9:1:0.1). $[\alpha]^D_{20}$ = +13.5 (c = 0.92, $CHCl_3$). IR (ATR): ν 3277 (NH), 1637 (C=O), 1513 (C-N), 1248 (COC). $^1$H-NMR ($CDCl_3$): δ 0.11-0.16 (m, 2H, $CH_{2cpr}$), 0.47-0.53 (m, 2H, $CH_{2cpr}$), 0.88 (d, *J* = 6.9, 3H, $CH_3$), 0.92-1.05 (m, 1H, $CH_{cpr}$), 1.80 (br s, 1H, NH), 2.08-2.18 (m, 1H, $H_3$), 2.33-2.40 (m, 1H, $H_1$), 2.45-2.53 (m, 1H, $H_6$), 2.48 (d, *J* = 6.9, 2H, NHC$\underline{H}_2$CH), 2.77 (dd, *J* = 16.8, 5.1, 1H, $H_3$), 3.24 (s, 2H, NHC$\underline{H}_2$), 3.75 (s, 3H, $OCH_3$), 4.46 (tdd, *J* = 10.7, 7.6, 5.1, 1H, $H_2$), 5.53 (s, 1H, $H_5$), 5.79 (d, *J* = 7.6, 1H, NHCO), 6.84 (d, *J* = 8.6, 2H, $H_{3'}$, $H_{5'}$), 7.14 (d, *J* = 8.6, 2H, $H_{2'}$, $H_{6'}$), 7.23-7.27 (m, 1H, $H_{5py}$), 7.74 (dt, d, *J* = 7.9, 1.9, 1H, $H_{4py}$), 8.46 (d, *J* = 1.9, 1H, $H_{2py}$), 8.60 (dd, *J* = 4.8, 1.6, 1H, $H_{6py}$). $^{13}$C-NMR ($CDCl_3$): δ 3.58 ($CH_{2cpr}$), 3.61 ($CH_{2cpr}$), 11.3 ($CH_{cpr}$), 20.2 ($CH_3$), 35.2 ($C_3$), 38.7 ($C_6$), 51.0 ($C_2$), 53.8 ($C_1$), 54.6 (NH$\underline{C}H_2$CH), 55.1 ($NHCH_2$), 55.3 ($OCH_3$), 114.2 ($C_{3'}$, $C_{5'}$), 123.5 ($C_{5py}$), 128.4 ($C_5$), 129.2 ($C_{2'}$, $C_{6'}$), 130.8 ($C_{3py}$), 133.1 ($C_{1'}$), 133.4 ($C_4$), 135.0 ($C_{4py}$), 147.5 ($C_{2py}$), 152.1 ($C_{6py}$), 158.7 ($C_{4'}$), 165.4 (NHCO). HPLC (method A, $t_R$, min): 13.57. MS (ESI, *m/z*, %): 406.1 ($[M+H]^+$, 100). Elemental analysis calculated for $C_{25}H_{31}N_3O_2$·2HCl·2$H_2O$: %C 58.36, %H 7.25, %N 8.17; experimental: %C 58.76, %H 6.97, %N, 8.02.

### 2.3. Synthesis of final compounds 4-7 (Scheme 1)

**(9*H*-Fluoren-9-yl)methyl (cyclopropylmethyl){[(1*R*,2*S*,6*R*)-4'-methoxy-6-methyl-2-nitro-1,2,3,6-tetrahydro[1,1'-biphenyl]-4-yl]methyl}carbamate, 36.** To a solution of **35** (459 mg, 1.36 mmol) in anhydrous DCM (13.6 mL) at 0 ºC, *N*-[(9*H*-fluoren-9-yl)methoxycarbonyloxy]succinimide (450 mg, 1.36 mmol) was added and the reaction was stirred at rt for 3 h. After this time, the mixture was washed with water (x2) and brine. The organic layer was dried over $Na_2SO_4$, filtered, and the solvent was evaporated under

reduced pressure. The crude was purified by flash chromatography (hexane to hexane/EtOAc 7:3) to afford compound **36** as a yellow oil (480 mg, 64%).

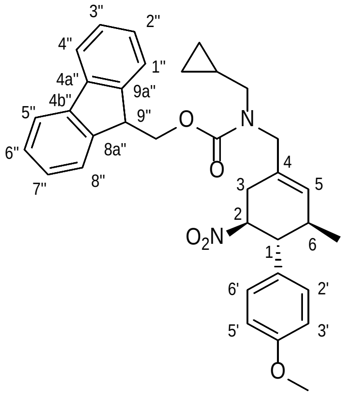

$R_f$: 0.50 (hexane/EtOAc 7:3). $[\alpha]^D_{20}$ = -3.0 (c = 1.00, $CHCl_3$). IR (ATR): ν 1696 (C=O), 1550 (C-N), 1513 ($NO_2$), 1248 (COC). $^1$H-NMR ($CDCl_3$, mixture of rotamers): δ -0.02 and 0.17 (s, 2H, $CH_{2cpr}$), 0.39 and 0.48 (s, 2H, $CH_{2cpr}$), 0.73-0.92 (m, 1H, $CH_{cpr}$), 0.74 (d, *J* = 8.5) and 0.79 (d, *J* = 6.9, 3H, $CH_3$), 2.12 (dd, *J* = 16.4, 5.3, 1/2H, 1/2$H_3$), 2.29-2.58 (m, 3/2H, 1/2$H_3$, $H_6$), 2.69 (t, *J* = 10.7) and 2.78 (t, *J* = 10.6, 1H, $H_1$), 2.88 (d, *J* = 6.9) and 3.08 (br s, 2H, NC$\underline{H}_2$CH), 3.60 (s) and 3.95 (AB system, *J* = 16.5, 2H, $NCH_2$), 3.78 (s, 3H, $OCH_3$), 4.18-4.27 (m, 1H, $H_{9''}$), 4.55-4.66 (m, 2H, $OCH_2$), 4.77 (td, *J* = 11.3, 5.7) and 4.90 (td, *J* = 11.2, 5.4, 1H, $H_2$), 5.26 and 5.42 (s, 1H, $H_5$), 6.82-6.88 (d, *J* = 8.1, 2H, $H_{3'}$, $H_{5'}$), 7.08 (d, *J* = 8.3, 2H, $H_{2'}$, $H_{6'}$), 7.29-7.36 (m, 2H, $H_{2''}$, $H_{7''}$), 7.37-7.45 (m, 2H, $H_{3''}$, $H_{6''}$), 7.55-7.61 (m, 2H, $H_{1''}$, $H_{8''}$), 7.73-7.82 (m, 2H, $H_{4''}$, $H_{5''}$). $^{13}$C-NMR ($CDCl_3$, mixture of rotamers): δ 3.8 ($2CH_{2cpr}$), 9.86 and 9.89 ($CH_{cpr}$), 19.5 and 19.6 ($CH_3$), 31.0 and 32.2 ($C_3$), 37.2 and 37.4 ($C_6$), 47.6 ($C_{9''}$), 50.7 and 51.2 (NC$\underline{H}_2$CH), 51.3 and 52.0 ($NCH_2$), 51.6 ($C_1$), 55.3 ($OCH_3$), 66.6 and 67.0 ($OCH_2$), 88.4 and 88.5 ($C_2$), 114.2 ($C_{3'}$, $C_{5'}$), 120.1 ($C_{4''}$, $C_{5''}$), 124.8 ($C_{1''}$, $C_{8''}$), 127.2 ($C_{2''}$, $C_{7''}$), 127.5 ($C_{1'}$), 127.8 ($C_{3''}$, $C_{6''}$), 128.3 ($C_5$), 129.0 ($C_{2'}$, $C_{6'}$), 130.3 ($C_4$), 141.6 ($C_{4a''}$, $C_{4b''}$), 144.1 ($C_{8a''}$, $C_{9a''}$), 156.2 (CO), 159.1 ($C_{4'}$).

**(9*H*-Fluoren-9-yl)methyl {[(1*R*,2*S*,6*R*)-2-amino-4'-methoxy-6-methyl-1,2,3,6-tetrahydro[1,1'-biphenyl]-4-yl]methyl}(cyclopropylmethyl)carbamate, 37.** Following general procedure D using **36** (332 mg, 0.60 mmol), compound **37** was obtained as a colorless oil (250 mg, 80%). Chromatography: EtOAc to EtOAc/MeOH 9:1.

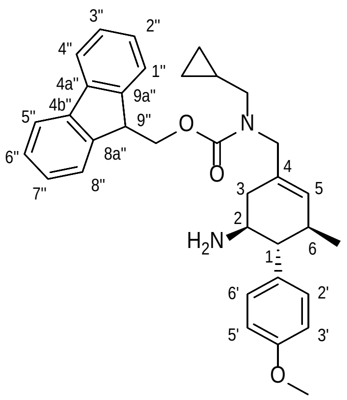


$R_f$: 0.40 (EtOAc /MeOH 8:2). $[\alpha]^D_{20}$ = -5.0 (c = 1.00, $CHCl_3$). IR (ATR): ν 1696 (C=O), 1512 (C-N), 1247 (COC). $^1$H-NMR ($CDCl_3$, mixture of rotamers): δ -0.02-0.08 and 0.17-29 (m, 2H, $CH_{2cpr}$), 0.36-0.44 and 0.45-0.53 (m, 2H, $CH_{2cpr}$), 0.72-0.99 (m, 4H, $CH_{cpr}$, $CH_3$), 1.73-1.85 and 1.86-1.95 (m, 1H, $H_3$), 1.97-2.39 (m, 5H, $H_1$, $H_3$, $H_6$, $NH_2$), 2.96 and 3.15 (br s, 3H, $H_2$, NC$\underline{H}_2$CH), 3.79 (s, 3H, $OCH_3$), 3.86-3.92 and 3.95-4.05 (m, 2H, $NCH_2$), 4.20-4.30 (m, 1H, $H_{9''}$), 4.42-4.56 (m, 2H, $OCH_2$), 5.29-5.40 (m, 1H, $H_5$), 6.82-6.92 (m, 2H, $H_{3'}$, $H_{5'}$), 7.11 (d, $J$ = 8.2, 2H, $H_{2'}$, $H_{6'}$), 7.28-7.35 (m, 2H, $H_{2''}$, $H_{7''}$), 7.35-7.43 (m, 2H, $H_{3''}$, $H_{6''}$), 7.61 (d, $J$ = 7.4, 2H, $H_{1''}$, $H_{8''}$), 7.72-7.80 (m, 2H, $H_{4''}$, $H_{5''}$). $^{13}$C-NMR ($CDCl_3$, mixture of rotamers): δ 3.6 ($CH_{2cpr}$), 3.7 ($CH_{2cpr}$), 9.86 and 9.93 ($CH_{cpr}$), 20.0 ($CH_3$), 35.6 and 35.7 ($C_3$), 38.0 ($C_6$), 47.6 ($C_{9''}$), 50.3 and 51.2 (NC$\underline{H}_2$CH), 51.6 and 52.2 ($NCH_2$), 51.9 ($C_2$), 55.4 ($OCH_3$), 56.6 and 56.8 ($C_1$), 67.2 ($OCH_2$), 114.2 ($C_{3'}$, $C_{5'}$), 120.1 ($C_{4''}$, $C_{5''}$), 125.0 ($C_{1''}$, $C_{8''}$), 127.2 ($C_{2''}$, $C_{7''}$), 127.7 ($C_{3''}$, $C_{6''}$), 129.4 ($C_{2'}$, $C_{6'}$), 129.7 ($C_5$), 130.7 ($C_4$), 134.1 ($C_{1'}$), 141.6 ($C_{4a''}$, $C_{4b''}$), 144.1 ($C_{8a''}$, $C_{9a''}$), 156.6 (CO), 159.1($C_{4'}$). HPLC (method A, $t_R$, min): 14.37. MS (ESI, *m/z*, %): 523.4 ($[M+H]^+$, 100).

***N*-[(1*R*,2*S*,6*R*)-4-{[(Cyclopropylmethyl)amino]methyl}-4'-methoxy-6-methyl-1,2,3,6-tetrahydro[1,1'-biphenyl]-2-yl]pyridine-4-carboxamide, 4.** Following general procedures E and F using **37** (60 mg, 0.11 mmol) and isonicotinic acid (14 mg, 0.11 mmol), compound **4** was obtained as a colorless oil (7 mg, 16%). Chromatography: EtOAc to EtOAc/MeOH/$NH_3$ 8:2:0.1.

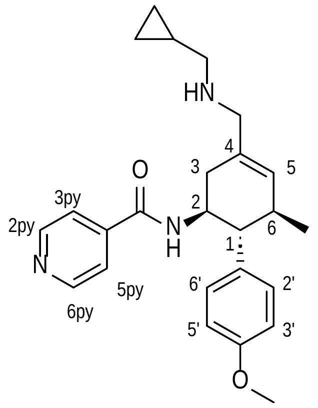


$R_f$: 0.30 (EtOAc/MeOH/$NH_3$ 8:2:0.1). $[\alpha]^D_{20}$ = +36.0 (c = 1.00, $CHCl_3$). IR (ATR): ν 3288 (NH), 1644 (C=O), 1513 (C-N), 1248 (COC). $^1$H-NMR ($CDCl_3$, 500 MHz): δ 0.11-0.18 (m, 2H, $CH_{2cpr}$), 0.46-0.53 (m, 2H, $CH_{2cpr}$), 0.88 (d, *J* = 6.9, 3H, $CH_3$), 0.95-1.03 (m, 1H, $CH_{cpr}$), 2.07-2.21 (m, 2H, $H_3$, NH), 2.37 (t, *J* = 10.4, 1H, $H_1$), 2.46-2.51 (m, 3H, $H_6$, NHC$\underline{H}_2$CH), 2.74 (dd, *J* = 17.0, 6.2, 1H, $H_3$), 3.25 (s, 2H, NHC$\underline{H}_2$), 3.76 (s, 3H, $OCH_3$), 4.40-4.50 (m, 1H, $H_2$), 5.55 (s, 1H, $H_5$), 5.89 (br s, 1H, NHCO), 6.84 (d, *J* = 8.6, 2H, $H_{3'}$, $H_{5'}$), 7.13 (d, *J* = 8.6, 2H, $H_{2'}$, $H_{6'}$), 7.15 (d, *J* = 6.1, 2H, $H_{3py}$, $H_{5py}$), 8.57 (d, *J* = 6.0, 2H, $H_{2py}$, $H_{6py}$). $^{13}$C-NMR ($CDCl_3$, 125 MHz): δ 3.6 ($CH_{2cpr}$), 3.7 ($CH_{2cpr}$), 11.1 ($CH_{cpr}$), 20.1 ($CH_3$), 35.0 ($C_3$), 38.7 ($C_6$), 51.1 ($C_2$), 53.7 ($C_1$), 54.4 (NH$\underline{C}H_2$CH), 54.9 (NH$CH_2$), 55.4 ($OCH_3$), 114.2 ($C_{3'}$, $C_{5'}$), 120.7 ($C_{3py}$, $C_{5py}$), 129.0 ($C_5$), 129.2 ($C_{2'}$, $C_{6'}$), 133.0 ($C_4$, $C_{1'}$), 142.2 ($C_{4py}$), 150.5 ($C_{2py}$, $C_{6py}$), 158.7 ($C_{4'}$), 165.4 (NHCO). HPLC (method A, $t_R$, min): 12.21. MS (ESI, *m/z*, %): 406.3 ($[M+H]^+$, 100). Elemental analysis calculated for $C_{25}H_{31}N_3O_2 \cdot 2HCl \cdot 3H_2O$: %C 56.39, %H 7.38, %N 7.89; experimental: %C 56.24, %H 7.10, %N 7.79.

***N*-[(1*R*,2*S*,6*R*)-4-{[(Cyclopropylmethyl)amino]methyl}-4'-methoxy-6-methyl-1,2,3,6-tetrahydro[1,1'-biphenyl]-2-yl]pyridine-2-carboxamide, 5.** Following general procedures E and F using **37** (60 mg, 0.11 mmol) and picolinic acid (14 mg, 0.11 mmol), compound **5** was obtained as a colorless oil (8 mg, 18%). Chromatography: EtOAc to EtOAc/MeOH/$NH_3$ 8:2:0.1.

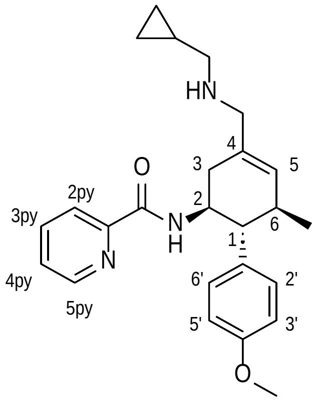


$R_f$: 0.50 (EtOAc/MeOH/$NH_3$ 8:2:0.1). $[\alpha]^{D}_{20}$ = +29.0 (c = 1.00, $CHCl_3$). IR (ATR): ν 3375 (NH), 1666 (C=O), 1512 (C-N), 1247 (COC). $^1$H-NMR ($CDCl_3$, 500 MHz): δ 0.30-0.33 (m, 2H, $CH_{2cpr}$), 0.59-0.63 (m, 2H, $CH_{2cpr}$), 0.87 (d, *J* = 6.8, 3H, $CH_3$), 1.44-1.20 (m, 1H, $CH_{cpr}$), 2.28-2.34 (m, 1H, $H_3$), 2.44 (t, *J* = 10.5, 1H, $H_1$), 2.47-2.53 (m, 1H, $H_6$), 2.70-2.79 (m, 3H, $H_3$, NHC$\underline{H}_2$CH), 3.49 (s, 2H, NHC$\underline{H}_2$), 3.68 (s, 3H, $OCH_3$), 4.48 (tdd, *J* = 10.7, 8.8, 5.2, 1H, $H_2$), 5.77 (s, 1H, $H_5$), 6.74 (d, *J* = 7.8, 2H, $H_{3'}$, $H_{5'}$), 7.11 (d, *J* = 7.7, 2H, $H_{2'}$, $H_{6'}$), 7.26-7.29 (m, 1H, $H_{5py}$), 7.67-7.73 (m, 1H, $H_{4py}$), 7.85 (d, *J* = 8.7, 1H, NHCO), 7.97 (d, *J* = 7.9, 1H, $H_{3py}$), 8.33-8.37 (m, 1H, $H_{6py}$). $^{13}$C-NMR ($CDCl_3$, 125 MHz): δ 4.36 ($CH_{2cpr}$), 4.44 ($CH_{2cpr}$), 8.3 ($CH_{cpr}$), 19.8 ($CH_3$), 35.0 ($C_3$), 38.9 ($C_6$), 49.8 ($C_2$), 52.1 (NH$\underline{C}H_2$CH), 52.4 ($NHCH_2$), 53.0 ($C_1$), 55.2 ($OCH_3$), 113.8 ($C_{3'}$, $C_{5'}$), 122.1 ($C_{3py}$), 126.0 ($C_{5py}$), 129.1 ($C_4$), 129.2 ($C_{2'}$, $C_{6'}$), 133.1 ($C_{1'}$), 134.3 ($C_5$), 137.2 ($C_{4py}$), 148.0 ($C_{6py}$), 150.0 ($C_{2py}$), 158.3 ($C_{4'}$), 165.4 (NHCO). HPLC (method A, $t_R$, min): 13.37. MS (ESI, *m/z*, %): 406.3 ($[M+H]^+$, 100). Elemental analysis calculated for $C_{25}H_{31}N_3O_2 \cdot 2HCl$: %C 62.76, %H 6.95, %N 8.78; experimental: %C 63.17, %H 7.00, %N 8.44.

***N*-[(1*R*,2*S*,6*R*)-4-{[(Cyclopropylmethyl)amino]methyl}-4'-methoxy-6-methyl-1,2,3,6-tetrahydro[1,1'-biphenyl]-2-yl]benzamide, 6.** Following general procedures E and F using **37** (60 mg, 0.11 mmol) and benzoic acid (14 mg, 0.11 mmol), compound **6** was obtained as a colorless oil (10 mg, 22%). Chromatography: EtOAc to EtOAc/MeOH/$NH_3$ 8:2:0.1.

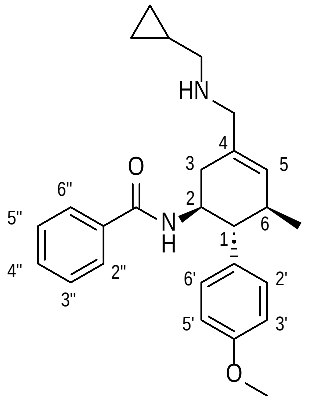

$R_f$: 0.30 (EtOAc/MeOH/$NH_3$ 8:2:0.1). $[\alpha]^D_{20}$ = +19.0 (c = 1.00, $CHCl_3$). IR (ATR): ν 3290 (NH), 1636 (C=O), 1514 (C-N), 1247 (COC). $^1$H-NMR ($CDCl_3$, 500 MHz): δ 0.23 (d, *J* = 5.0, 2H, $CH_{2cpr}$), 0.54 (d, *J* = 8.0, 2H, $CH_{2cpr}$), 0.87 (d, *J* = 6.7, 3H, $CH_3$), 1.01-1.09 (m, 1H, $CH_{cpr}$), 1.83 (br s, 1H, NH), 2.22-2.29 (m, 1H, $H_3$), 2.42 (t, *J* = 10.8, 1H, $H_1$), 2.46-2.53 (m, 1H, $H_6$), 2.56-2.66 (m, 2H, NHC$\underline{H}_2$CH), 2.73 (dd, *J* = 17.2, 5.0, 1H, $H_3$), 3.37 (s, 2H, NHC$\underline{H}_2$), 3.74 (s, 3H, $OCH_3$), 4.42-4.49 (m, 1H, $H_2$), 5.62 (s, 1H, $H_5$), 6.17 (br s, 1H, NHCO), 6.82 (d, *J* = 8.1, 2H, $H_{3'}$, $H_{5'}$), 7.14 (d, *J* = 8.3, 2H, $H_{2'}$, $H_{6'}$), 7.24-7.28 (m, 2H, $H_{3''}$, $H_{5''}$), 7.33-7.40 (m, 3H, $H_{2''}$, $H_{4''}$, $H_{6''}$). $^{13}$C-NMR ($CDCl_3$, 125 MHz): δ 3.97 ($CH_{2cpr}$), 4.07 ($CH_{2cpr}$), 9.6 ($CH_{cpr}$), 20.0 ($CH_3$), 34.9 ($C_3$), 38.8 ($C_6$), 50.6 ($C_2$), 53.3 ($C_1$, NH$\underline{C}H_2$CH), 53.9 ($NHCH_2$), 55.3 ($OCH_3$), 114.1 ($C_{3'}$, $C_{5'}$), 126.8 ($C_{2''}$, $C_{6''}$), 128.5 ($C_{3''}$, $C_{5''}$), 129.3 ($C_{2'}$, $C_{6'}$), 131.2 ($C_5$, $C_{4''}$), 133.2 ($C_4$, $C_{1'}$), 135.0 ($C_{1''}$), 158.5 ($C_{4'}$), 167.4 (NHCO). HPLC (method A, $t_R$, min): 15.86. MS (ESI, *m/z*, %): 405.2 ($[M+H]^+$, 100). Elemental analysis calculated for $C_{26}H_{32}N_2O_2 \cdot HCl \cdot H_2O$: %C 68.03, %H 7.69, %N 6.10, %S; experimental: %C 68.40, %H 7.27, %N 6.23.

***N*-[(1*R*,2*S*,6*R*)-4-{[(Cyclopropylmethyl)amino]methyl}-4'-methoxy-6-methyl-1,2,3,6-tetrahydro[1,1'-biphenyl]-2-yl]-1-methylpiperidine-3-carboxamide, 7.** Following general procedures E and F using **37** (60 mg, 0.11 mmol) and 1-methylpiperidine-3-carboxylic acid (16 mg, 0.11 mmol), compound **7** was obtained as a colorless oil (7 mg, 14%). Chromatography: DCM to DCM/MeOH/$NH_3$ 8.5:1.5:0.1.

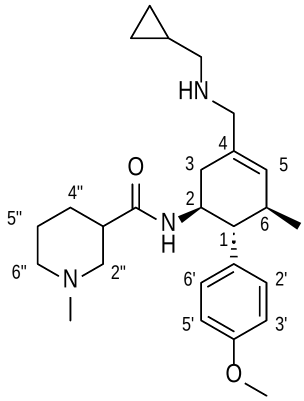


$R_f$: 0.40 (DCM/MeOH/$NH_3$ 8.5:1.5:0.1). $[\alpha]^D_{20}$ = -8.0 (c = 1.00, $CHCl_3$). IR (ATR): ν 3261 (NH), 1644 (C=O), 1514 (C-N), 1248 (COC). $^1$H-NMR ($CDCl_3$): δ 0.17-0.29 (m, 2H, $CH_{2cpr}$), 0.49-0.62 (m, 2H, $CH_{2cpr}$), 0.84 (d, *J* = 8.7, 3H, $CH_3$), 1.00-1.13 (m, 2H, $CH_{cpr}$, $H_{5''}$), 1.26-1.43 (m, 1H, $H_{4''}$), 1.46-1.57 (m, 1H, $H_{5''}$), 1.96-2.07 (m, 2H, $H_3$, $H_{4''}$), 2.14 (s, 3H, $NCH_3$), 2.16-2.22 (m, 2H, $H_{2''}$, $H_{6''}$), 2.26-2-35 (m, 3H, $H_1$, $H_{3''}$, $H_{6''}$), 2.36-2.53 (m, 2H, $H_6$, $H_{2''}$), 2.58 (dd, *J* = 7.0, 2.2, 2H, NHC$\underline{H}_2$CH), 2.69 (dd, *J* = 16.8, 5.0, 1H, $H_3$), 3.06 (br s, 2H, 2NH), 3.31 (s, 2H, NHC$\underline{H}_2$), 3.77 (s, 3H, $OCH_3$), 4.18-4.35 (m, 1H, $H_2$), 5.58 (s, 1H, $H_5$), 6.83 (d, *J* = 8.8, 2H, $H_{3'}$, $H_{5'}$), 7.10 (d, *J* = 8.7, 2H, $H_{2'}$, $H_{6'}$). $^{13}$C-NMR ($CDCl_3$): δ 3.8 ($CH_{2cpr}$), 4.0 ($CH_{2cpr}$), 10.1 ($CH_{cpr}$), 20.0 ($CH_3$), 25.8 ($C_{5''}$), 29.8 ($C_{4''}$), 35.4 ($C_3$), 38.9 ($C_6$), 41.8 ($C_{3''}$), 45.9 ($NCH_3$), 50.1 ($C_2$), 53.4 ($C_1$), 53.7 (NH$\underline{C}H_2$CH), 54.2 ($NHCH_2$), 55.5 ($OCH_3$), 55.6 ($C_{6''}$), 56.9 ($C_{2''}$), 114.0 ($C_{3'}$, $C_{5'}$), 129.5 ($C_{2'}$, $C_{6'}$), 130.8 ($C_5$), 131.6 ($C_4$), 134.2 ($C_{1'}$), 158.6 ($C_{4'}$), 176.2 (NHCO). HPLC (method A, $t_R$, min): 9.86. MS (ESI, *m/z*, %): 426.2 ($[M+H]^+$, 100). Elemental analysis calculated for $C_{26}H_{39}N_3O_2\cdot 2HCl\cdot 3H_2O$: %C 56.51, %H 8.57, %N 7.60; experimental: %C 56.89, %H 8.13, %N 7.72.

## 2.4. Synthesis of final compounds 8-12 (Scheme 1)

***tert*-Butyl (cyclopropylmethyl){[(1*R*,2*S*,6*R*)-4'-methoxy-6-methyl-2-nitro-1,2,3,6-tetrahydro[1,1'-biphenyl]-4-yl]methyl}carbamate, 38.** Following general procedure G using **35** (2.0 g, 6.05 mmol), triethylamine (1.1 mL, 7.87 mmol) and di-*tert*-butyl decarbonate (2.64 g, 12.1 mmol), compound **38** was obtained as a colorless oil (1.94 g, 75%). Chromatography: hexane to hexane/EtOAc 7:3.

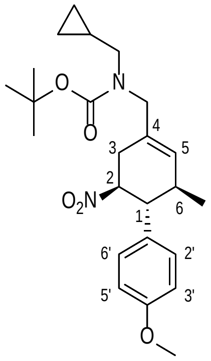


$R_f$: 0.50 (hexane/EtOAc 8:2). $[\alpha]^D_{20}$ = +12.0 (c = 1.00, $CHCl_3$). IR (ATR): ν 1690 (C=O), 1551 ($NO_2$), 1515 (C-N), 1250 (COC). $^1$H-NMR ($CDCl_3$, mixture of rotamers): δ 0.16-0.25 (m, 2H, $CH_{2cpr}$), 0.47-0.53 (m, 2H, $CH_{2cpr}$), 0.89 (d, *J* = 6.9, 3H, $CH_3$), 0.94-0.98 (m, 1H, $CH_{cpr}$), 1.46 (s, 9H, $3CH_3$), 2.42-2.52 (m, 1H, $H_6$), 2.59-2.75 (m, 2H, $2H_3$), 2.80 (t, *J* = 11.0, 1H, $H_1$), 3.06 (br s, 2H, $NC\underline{H}_2CH$), 3.77 (s, 3H, $OCH_3$), 3.94 (br s, 2H, $NC\underline{H}_2$), 4.93 (ddd, *J* = 11.6, 10.5, 5.7, 1H, $H_2$), 5.46 (s, 1H, $H_5$), 6.83 (d, *J* = 8.6, 2H, $H_{3'}$, $H_{5'}$), 7.09 (d, *J* = 8.6, 2H, $H_{2'}$, $H_{6'}$). $^{13}$C-NMR ($CDCl_3$, mixture of rotamers): δ 3.7 ($CH_{2cpr}$), 3.8 ($CH_{2cpr}$), 10.1 ($CH_{cpr}$), 19.6 ($CH_3$), 28.6 ($3CH_3$), 32.4 (br, $C_3$), 37.4 ($C_6$), 50.8 (br, $N\underline{C}H_2CH$), 51.4 (br, $NCH_2$), 51.7 ($C_1$), 55.3 ($OCH_3$), 79.9 ($\underline{C}(CH_3)_3$), 88.6 ($C_2$), 114.3 ($C_{3'}$, $C_{5'}$), 129.0 (br, $C_5$), 129.1 ($C_{2'}$, $C_{6'}$), 130.4 ($C_4$, $C_{1'}$), 155.9 (NCOO), 159.1 ($C_{4'}$).

***tert*-Butyl {[(1*R*,2*S*,6*R*)-2-amino-4'-methoxy-6-methyl-1,2,3,6-tetrahydro[1,1'-biphenyl]-4-yl]methyl}(cyclopropylmethyl)carbamate, 39.** To a solution of compound **38** (261 mg, 0.61 mmol) and DIPEA (0.74 mL, 4.24 mmol) in anhydrous DCM (6.1 mL) at 0 ºC, trichlorosilane (0.31 mL, 3.03 mmol) was added dropwise and the mixture was stirred overnight at rt. After this time, the reaction was quenched with a sat. $NaHCO_3$ solution. The solvent was evaporated and the residue was suspended in water and extracted with EtOAc (x2). The organic layers were washed with brine, dried over $Na_2SO_4$, filtered and evaporated under reduced pressure. The crude was purified by flash chromatography (DCM to DCM/MeOH/$NH_3$ 9:1:0.1) to afford compound **39** as a colorless oil (180 mg, 74%).

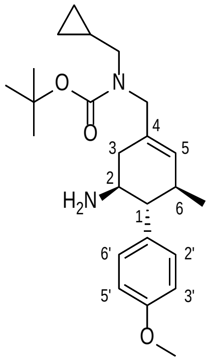

$R_f$: 0.50 (DCM/MeOH/$NH_3$ 9:1:0.1). $[\alpha]^D_{20}$ = +17.0 (c = 1.00, $CHCl_3$). IR (ATR): ν 1689 (C=O), 1512 (C-N), 1248 (COC). $^1$H-NMR ($CDCl_3$, mixture of rotamers): δ 0.15-0.25 (m, 2H, $CH_{2cpr}$), 0.42-0.52 (m, 2H, $CH_{2cpr}$), 0.79 (d, $J$ = 7.0, 3H, $CH_3$), 0.94-1.03 (m, 1H, $CH_{cpr}$), 1.46 (s, 9H, 3$CH_3$), 1.83-1.96 (m, 1H, $H_3$), 2.02 (t, $J$ = 10.4, $H_1$), 2.25-2.39 (m, 2H, $H_3$, $H_6$), 2.95-3.11 (br m, 2H, NC$\underline{H}_2$CH), 3.15 (td, $J$ = 10.5, 5.3, 1H, $H_2$), 3.79 (s, 3H, $OCH_3$), 3.81-3.99 (br m, 2H, $NCH_2$), 5.36 (s, 1H, $H_5$), 6.87 (d, $J$ = 8.5, 2H, $H_{3'}$, $H_{5'}$), 7.10 (d, $J$ = 8.5, 2H, $H_{2'}$, $H_{6'}$). $^{13}$C-NMR ($CDCl_3$, mixture of rotamers): δ 3.6 ($CH_{2cpr}$), 3.7 ($CH_{2cpr}$), 10.1 ($CH_{cpr}$), 20.1 ($CH_3$), 28.6 (3$CH_3$), 35.6 (br, $C_3$), 38.0 ($C_6$), 50.5 (br, N$\underline{C}H_2$CH), 51.9 ($C_2$), 52.1 (br, $NCH_2$), 55.4 ($OCH_3$), 56.9 ($C_1$), 79.5 ($\underline{C}(CH_3)_3$), 114.2 ($C_{3'}$, $C_{5'}$), 129.1 (br, $C_5$), 129.4 ($C_{2'}$, $C_{6'}$), 131.8 ($C_4$), 134.2 ($C_{1'}$), 156.0 (CO), 158.5 ($C_{4'}$). HPLC (method A, $t_R$, min): 8.30. MS (ESI, *m/z*, %): 401.4 ([M+H]$^+$, 100).

**5-{[(1*R*,2*S*,6*R*)-4-{[(*tert*-Butoxycarbonyl)(cyclopropylmethyl)amino] methyl}-4'-methoxy-6-methyl-1,2,3,6-tetrahydro[1,1'-biphenyl]-2-yl]amino}-5-oxopentanoic acid, 40.** A solution of compound **39** (60 mg, 0.15 mmol) and glutaric anhydride (19 mg, 0.17 mmol) in anhydrous DMF (1.5 mL) was stirred overnight at rt. Then, 1 M HCl solution was added until acid pH and the organic layer was washed with brine, dried over $Na_2SO_4$, filtered and evaporated under reduced pressure. The crude was purified by flash chromatography (DCM to DCM/EtOH 9:1) to afford compound **40** as a brown oil (27 mg, 35%).

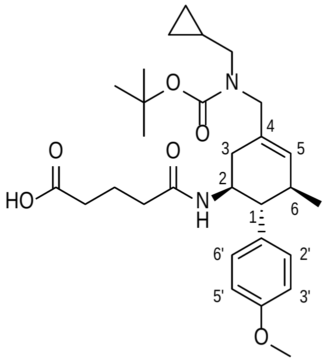

$R_f$: 0.50 (DCM/EtOH 8:2). $[\alpha]^D_{20}$ = -5.0 (c = 1.00, $CHCl_3$). IR (ATR): ν 3336 (OH), 1688 (C=O), 1667 (C=O), 1513 (C-N), 1249 (COC). $^1$H-NMR ($CDCl_3$, mixture of rotamers): δ 0.15-0.21 (m, 2H, $CH_{2cpr}$), 0.42-0.55 (m, 2H, $CH_{2cpr}$), 0.81 (d, *J* = 6.9, 3H, $CH_3$), 0.92-1.03 (m, 1H, $CH_{cpr}$), 1.46 (s, 9H, $3CH_3$), 1.50-1.69 (m, 2H, $COCH_2C\underline{H}_2$), 1.86-2.10 (m, 5H, $HOCOC\underline{H}_2$, $C\underline{H}_2CONH$, $H_3$), 2.18 (t, *J* = 10.6, 1H, $H_1$), 2.34-2.49 (m, 2H, $H_3$, $H_6$), 2.99-3.18 (br m, 2H, $NC\underline{H}_2CH$), 3.76 (s, 3H, $OCH_3$), 3.78-4.03 (br m, 2H, $NCH_2$), 4.27-4.42 (m, 1H, $H_2$), 5.37 (s, 1H, $H_5$), 5.39-5.47 (br s, 1H, CONH), 6.81 (d, *J* = 8.8, 2H, $H_{3'}$, $H_{5'}$), 7.06 (d, *J* = 8.8, 2H, $H_{2'}$, $H_{6'}$). $^{13}$C-NMR ($CDCl_3$, mixture of rotamers): δ 3.8 ($2CH_{2cpr}$), 10.1 ($CH_{cpr}$), 20.1 ($CH_3$), 20.9 ($COCH_2\underline{C}H_2$), 28.6 ($3CH_3$), 32.6 ($C_3$), 33.4 ($\underline{C}H_2CONH$), 35.4 ($HOCO\underline{C}H_2$), 38.7 ($C_6$), 49.8 ($C_2$), 50.7 ($N\underline{C}H_2CH$, $NCH_2$), 53.9 ($C_1$), 55.3 ($OCH_3$), 79.7 ($\underline{C}(CH_3)_3$), 114.0 ($C_{3'}$, $C_{5'}$), 129.3 ($C_5$, $C_{2'}$, $C_{6'}$), 131.6 ($C_4$), 133.3 ($C_{1'}$), 158.6 ($C_{4'}$), 172.0 (CONH), 177.1 (COOH), 177.3 ($\underline{C}OOC(CH_3)_3$). HPLC (method A, $t_R$, min): 19.70. MS (ESI, *m/z*, %): 415.3 ($[M\text{-}Boc]^+$, 100).

**Methyl 5-({(1*R*,2*S*,6*R*)-4-[3-*tert*-butoxy-2-(cyclopropylmethyl)-3-oxopropyl]-4'-methoxy-6-methyl-1,2,3,6-tetrahydro[1,1'-biphenyl]-2-yl}amino)pentanoate, 41.** A solution of compound **39** (150 mg, 0.37 mmol), methyl 5-bromovalerate (64 µL, 0.45 mmol), potassium carbonate (104 mg, 0.75 mmol) and potassium iodide (19 mg, 0.11 mmol) in anhydrous ACN (3.7 mL) was stirred for 4 h at rt. The solvent was evaporated and the residue was suspended in water and extracted with EtOAc (x2). The organic layers were washed with brine, dried over $Na_2SO_4$, filtered and evaporated under reduced pressure. The crude was purified by flash chromatography (DCM to DCM/MeOH/$NH_3$ 9.5:0.5:0.1) to afford compound **41** as a yellow oil (52 mg, 35%).

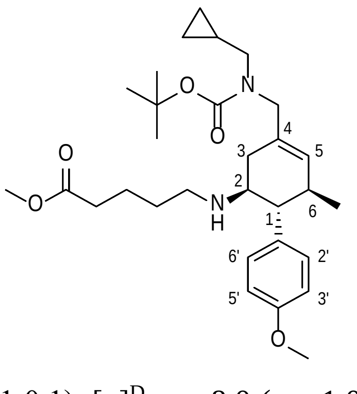

$R_f$: 0.60 (DCM/MeOH/$NH_3$ 9:1:0.1). $[\alpha]^D_{20}$ = -8.0 (c = 1.00, $CHCl_3$). IR (ATR): ν 1739 (C=O), 1691 (C=O), 1512 (C-N), 1248 (COC). $^1$H-NMR ($CDCl_3$, mixture of rotamers): δ 0.10-0.26 (m, 2H, $CH_{2cpr}$), 0.39-0.54 (m, 2H, $CH_{2cpr}$), 0.78 (d, *J* = 6.8, 3H, $CH_3$), 0.90-1.05 (m, 1H, $CH_{cpr}$), 1.23-1.33 (m, 2H, $C\underline{H}_2CH_2NH$), 1.36-1.55 (m, 11H, $3CH_3$,

$COCH_2C\underline{H}_2$), 1.80-1.99 (m, 1H, $H_3$), 2.10-2.25 (m, 3H, $COCH_2$, $H_1$), 2.26-2.42 (m, 3H, $H_3$, $H_6$, 1/2$C\underline{H}_2NH$), 2.53-2.67 (m, 1H, 1/2$C\underline{H}_2NH$), 2.86-3.17 (br m, 3H, $NC\underline{H}_2CH$, $H_2$), 3.64 (s, 3H, $COOCH_3$), 3.79 (s, 3H, $OCH_3$), 3.85 (br s, $NCH_2$), 5.36 (s, 1H, $H_5$), 6.86 (d, $J$ = 8.6, 2H, $H_{3'}$, $H_{5'}$), 7.11 (d, $J$ = 8.6, 2H, $H_{2'}$, $H_{6'}$). $^{13}$C-NMR ($CDCl_3$, mixture of rotamers): δ 3.7 ($2CH_{2cpr}$), 10.1 ($CH_{cpr}$), 20.1 ($CH_3$), 22.4 ($COCH_2\underline{C}H_2$), 28.6 ($3CH_3$), 29.0 ($\underline{C}H_2CH_2NH$), 32.8 (br, $C_3$), 33.7 ($CO\underline{C}H_2$), 38.0 ($C_6$), 46.4 (br, $CH_2NH$), 50.5 ($N\underline{C}H_2CH$), 51.6 ($COO\underline{C}H_3$, $NCH_2$), 54.1 (br, $C_1$), 55.3 ($OCH_3$), 57.7 ($C_2$), 79.4 ($\underline{C}(CH_3)_3$, 114.3 ($C_{3'}$, $C_{5'}$), 128.8 ($C_5$), 129.4 ($C_{2'}$, $C_{6'}$), 131.4 ($C_4$), 133.5 ($C_{1'}$), 156.0 ($\underline{C}OOC(CH_3)_3$), 158.6 ($C_{4'}$), 174.0 ($\underline{C}OOCH_3$). HPLC (method A, $t_R$, min): 13.47. MS (ESI, *m/z*, %): 515.2 ($[M+H]^+$, 100).

**5-{[(1*R*,2*S*,6*R*)-4-{[(*tert*-Butoxycarbonyl)(cyclopropylmethyl)amino] methyl}-4'-methoxy-6-methyl-1,2,3,6-tetrahydro[1,1'-biphenyl]-2-yl]amino} pentanoic acid, 42.** A solution of compound **41** (21 mg, 0.04 mmol) and lithium hydroxide monohydrate (9 mg, 0.21 mmol) in a 3:1 mixture of THF:$H_2O$ (0.5 mL) was stirred 48 h at rt. After this time, 1 M HCl solution was added until acid pH. The organic layer was washed with brine, dried over $Na_2SO_4$, filtered and evaporated under reduced pressure, to afford compound **42** as a colorless oil (13 mg, 62%).

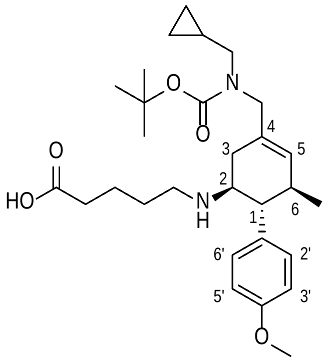


$R_f$: 0.50 (DCM/MeOH 9:1). $[\alpha]^D{}_{20}$ = +8.0 (c = 1.00, $CHCl_3$). IR (ATR): ν 3399 (OH), 1688 (C=O), 1514 (C-N), 1252 (COC). $^1$H-NMR ($CDCl_3$, mixture of rotamers): δ 0.15-0.25 (m, 2H, $CH_{2cpr}$), 0.44-0.53 (m, 2H, $CH_{2cpr}$), 0.80 (d, $J$ = 6.8, 3H, $CH_3$), 0.91-1.02 (m, 1H, $CH_{cpr}$), 1.21-1.33 (m, 2H, $COCH_2C\underline{H}_2$), 1.39-1.55 (m, 11H, $3CH_3$, $C\underline{H}_2CH_2NH$), 2.09 (t, $J$ = 6.3, 2H, $COCH_2$), 2.33-2.63 (m, 4H, $2H_3$, $H_6$, 1/2$C\underline{H}_2NH$), 2.70-2.84 (m, 2H, $H_1$, 1/2$C\underline{H}_2NH$), 2.86-3.17 (br m, 2H, $NC\underline{H}_2CH$), 3.36-3.48 (m, 1H, $H_2$), 3.76 (s, 3H, $OCH_3$), 3.82-3.89 (br m, 2H, $NCH_2$), 5.40 (s, 1H, $H_5$), 6.85 (d, $J$ = 8.5, 2H, $H_{3'}$, $H_{5'}$), 7.23 (d, $J$ = 8.5, 2H, $H_{2'}$, $H_{6'}$), 8.09 (br s, 1H, COOH). $^{13}$C-NMR ($CDCl_3$, mixture of rotamers): δ 3.6 ($CH_{2cpr}$), 3.7 ($CH_{2cpr}$), 10.1 ($CH_{cpr}$), 19.4 ($CH_3$), 21.3 ($COCH_2\underline{C}H_2$), 24.6 ($\underline{C}H_2CH_2NH$),

28.6 (3CH3), 29.6 (C3), 32.7 (COCH2), 38.4 (C6), 44.4 (CH2NH), 50.5 (C1), 51.1 (NCH2CH) 51.5 (NCH2), 55.4 (OCH3), 58.2 (C2), 80.0 (C(CH3)3, 114.9 (C3', C5'), 128.5 (C5), 130.0 (C2', C6', C4, C1'), 156.0 (COOC(CH3)3), 159.4 (C4'), 175.5 (COOH). HPLC (method A, $t_R$, min): 14.53. MS (ESI, *m/z*, %): 501.3 ($[M+H]^+$, 100).

***tert*-Butyl ({(1*R*,2*S*,6*R*)-2-[*N*',*N*''-bis(*tert*-butoxycarbonyl) carbamimidamido]-4'-methoxy-6-methyl-1,2,3,6-tetrahydro[1,1'-biphenyl]-4-yl}methyl) (cyclopropylmethyl)carbamate, 43.** A solution of compound **39** (120 mg, 0.30 mmol), *N*,*N'*-di-Boc-1*H*-pyrazole-1-carboxamidine (93 mg, 0.30 mmol) and triethylamine (0.13 mL, 0.90 mmol) in anhydrous ACN (1.8 mL) was stirred overnight at rt. Then, the solvent was evaporated under reduced pressure and the crude was purified by flash chromatography (hexane to hexane/EtOAc 7:3) to afford compound **43** as a colorless oil (57 mg, 30%).

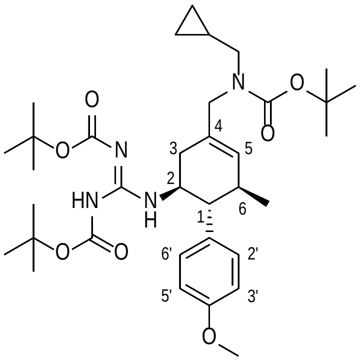

$R_f$: 0.50 (hexane/EtOAc 7:3). $[\alpha]^D_{20}$ = +1.0 (c = 1.00, $CHCl_3$). IR (ATR): ν 1721 (C=O), 1635 (C=N), 1513 (C-N), 1247 (COC). $^1$H-NMR ($CDCl_3$, mixture of rotamers): δ 0.14-0.29 (m, 2H, $CH_{2cpr}$), 0.41-0.54 (m, 2H, $CH_{2cpr}$), 0.82 (d, *J* = 7.0, 3H, $CH_3$), 0.89-1.05 (m, 1H, $CH_{cpr}$), 1.39 (s, 9H, 3$CH_3$), 1.46 (s, 18H, 6$CH_3$), 1.70-2.00 (m, 1H, $H_3$), 2.23 (t, *J* = 11.4, 1H, $H_1$), 2.38-2.54 (m, 1H, $H_6$), 2.54-2.74 (m, 1H, $H_3$), 2.97-3.24 (br m, 2H, N$C\underline{H}_2$CH), 3.76 (s, 3H, $OCH_3$), 3.78-4.01 (br m, 2H, $NCH_2$), 4.48 (br s, 1H, $H_2$), 5.35 (s, 1H, $H_5$), 6.77 (d, *J* = 8.7, 2H, $H_{3'}$, $H_{5'}$), 7.12 (d, *J* = 8.9, 2H, $H_{2'}$, $H_{6'}$), 8.12 (d, *J* = 8.0 , 1H, NH), 11.23 (s, 1H, NHCO). $^{13}$C-NMR ($CDCl_3$, mixture of rotamers): δ 3.6 (2$CH_{2cpr}$), 10.1 ($CH_{cpr}$), 20.2 ($CH_3$), 28.1, 28.5, 28.6 (3x3$CH_3$), 34.1 (br, $C_3$), 37.9 ($C_6$), 50.4 (N$\underline{C}H_2$CH), 51.3 (br, $NCH_2$, $C_2$), 53.9 ($C_1$), 55.3 ($OCH_3$), 79.5 (2$\underline{C}(CH_3)_3$), 82.8 ($\underline{C}(CH_3)_3$), 113.6 ($C_{3'}$, $C_{5'}$) 129.6 (br, $C_5$), 131.7 ($C_4$, $C_{2'}$, $C_{6'}$), 133.1 ($C_{1'}$), 153.0, 155.8, 155.9 (3CO), 158.4 ($C_{4'}$), 163.8 (C=N). HPLC (method B, $t_R$, min): 10.94. MS (ESI, *m/z*, %): 543.4 ($[M-Boc]^+$, 100).

***N*$_{\alpha}$-(*tert*-Butoxycarbonyl)-*N*-[(1*R*,2*S*,6*R*)-4-{[(*tert*-butoxycarbonyl) (cyclopropylmethyl)amino]methyl}-4'-methoxy-6-methyl-1,2,3,6-tetrahydro 1,1'-biphenyl]-2-yl]-L-tryptophanamide, 44.** Following general procedure E using **39** (80 mg, 0.20 mmol) and *N*$_{\alpha}$-(*tert*-butoxycarbonyl)-L-tryptophan (61 mg, 0.20 mmol), compound **44** was obtained as a colorless oil (47 mg, 34%). Chromatography: DCM to DCM/MeOH/$NH_3$ 9.5:0.5:0.1.

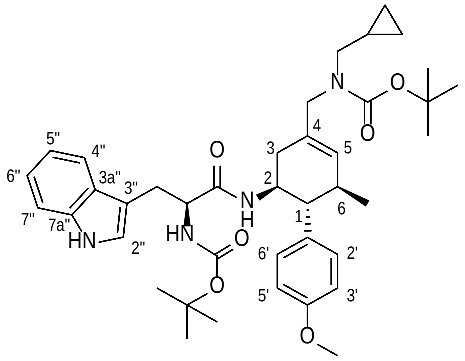


$R_f$: 0.80 (DCM/MeOH/$NH_3$ 9:1:0.1). $[\alpha]^D_{20}$ = +10.0 (c = 1.00, $CHCl_3$). IR (ATR): ν 3290 (NH), 1668 (C=O), 1513 (C-N), 1250 (COC). $^1$H-NMR ($CDCl_3$, mixture of rotamers): δ 0.21-0.28 (m, 2H, $CH_{2cpr}$), 0.48-0.57 (m, 2H, $CH_{2cpr}$), 0.85 (d, *J* = 6.8, 3H, $CH_3$), 0.95-1.10 (m, 1H, $CH_{cpr}$), 1.29 (s, 9H, 3$CH_3$), 1.47 (s, 9H, 3$CH_3$), 1.98-2.10 (m, 1H, $H_3$), 2.30-2.48 (m, 5H, $H_1$, $H_3$, $H_6$, C$\underline{H}_2$CHNH), 3.11 (d, *J* = 6.9, 2H, NC$\underline{H}_2$CH), 3.64 (s, 3H, $OCH_3$), 3.84-4.06 (br m, 3H, $NCH_2$, $CH_2$C$\underline{H}$NH), 4.26-4.39 (m, 1H, $H_2$), 5.47 (s, 1H, $H_5$), 6.80-6.88 (m, 3H, $H_{3'}$, $H_{5'}$, $H_{2''}$), 6.98 (t, *J* = 7.2, 1H, $H_{6''}$) 7.06 (t, *J* = 7.4, 1H, $H_{5''}$), 7.16 (d, *J* = 8.1, 2H, $H_{2'}$, $H_{6'}$), 7.28 (d, *J* = 8.0, 1H, $H_{7''}$), 7.38 (d, *J* = 8.5, 1H, $H_{4''}$). $^{13}$C-NMR ($CDCl_3$, mixture of rotamers): δ 4.1 (2$CH_{2cpr}$), 11.0 ($CH_{cpr}$), 20.4 ($CH_3$), 28.6 (3$CH_3$), 28.8 (3$CH_3$), 29.1 ($\underline{C}H_2$CHNH), 34.8 ($C_3$), 39.8 ($C_6$), 50.9 ($C_2$), 51.9 (N$\underline{C}H_2$CH), 53.3 ($NCH_2$), 54.8 ($C_1$), 55.7 ($OCH_3$), 56.5 ($\underline{C}H_2$CHNH), 80.3 ($\underline{C}(CH_3)_3$), 81.1 ($\underline{C}(CH_3)_3$), 111.3 ($C_{3''}$), 112.1 ($C_{7''}$), 115.0 ($C_{3'}$, $C_{5'}$), 119.4 ($C_{4''}$), 119.5 ($C_{6''}$), 122.5 ($C_{5''}$), 123.9 ($C_{2''}$), 128.8 ($C_{7a''}$), 130.2 ($C_5$), 130.5 ($C_4$, $C_{2'}$, $C_{6'}$), 135.0 ($C_{1'}$), 137.9 ($C_{3a''}$), 157.5, 157.6 (2CO), 160.0 ($C_{4'}$), 174.2 (CH$\underline{C}$ONH). HPLC (method B, $t_R$, min): 15.82. MS (ESI, *m/z*, %): 587.4 ([M-Boc]$^+$, 100).

**5-{[(1*R*,2*S*,6*R*)-4-{[(Cyclopropylmethyl)amino]methyl}-4'-methoxy-6-methyl-1,2,3,6-tetrahydro[1,1'-biphenyl]-2-yl]amino}-5-oxopentanoic acid, 8.** Following general procedure I using **40** (25 mg, 0.05 mmol), compound **8** was obtained as a brown oil (13 mg, 67%).

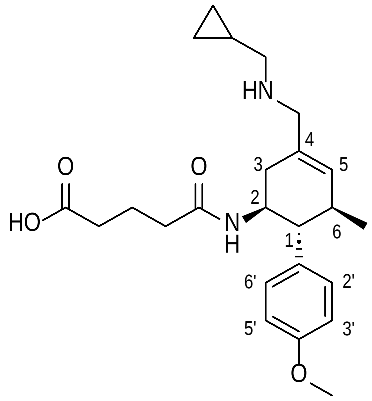


$R_f$: 0.30 (DCM/MeOH 8:2). $[\alpha]^D_{20}$ = -18.0 (c = 1.00, $CHCl_3$). IR (ATR): ν 3253 (OH), 1730 (C=O), 1639 (C=O), 1513 (C-N), 1248 (COC). $^1$H-NMR (MeOH-*d4*, 500 MHz): δ 0.34-0.52 (m, 2H, $CH_{2cpr}$), 0.64-0.80 (m, 2H, $CH_{2cpr}$), 0.88 (d, *J* = 7.0, 3H, $CH_3$), 1.07-1.18 (m, 1H, $CH_{cpr}$), 1.45-1.60 (m, 2H, $COCH_2C\underline{H}_2$), 1.83 (t, *J* = 7.5, 2H, $HOCOC\underline{H}_2$) 1.87-2.00 (m, 2H, $C\underline{H}_2CONH$), 2.14-2.24 (m, 1H, $H_3$), 2.34 (t, *J* = 10.8, 1H, $H_1$), 2.42 (dd, *J* = 16.7, 5.4, 1H, $H_3$), 2.46-2.55 (m, 1H, $H_6$), 2.92 (d, *J* = 7.4, 2H, $NHC\underline{H}_2CH$), 3.63 (s, 2H, $NHC\underline{H}_2$), 3.76 (s, 3H, $OCH_3$), 4.31 (td, *J* = 11.0, 5.2, 1H, $H_2$), 5.86 (s, 1H, $H_5$), 6.84 (d, *J* = 8.7, 2H, $H_{3'}$, $H_{5'}$), 7.11 (d, *J* = 8.7, 2H, $H_{2'}$, $H_{6'}$). $^{13}$C-NMR (MeOH-*d4*, 125 MHz): δ 4.7 ($2CH_{2cpr}$), 8.0 ($CH_{cpr}$), 19.8 ($CH_3$), 22.2 ($COCH_2\underline{C}H_2$), 33.3 ($HOCO\underline{C}H_2$), 35.2 ($C_3$), 36.1 ($\underline{C}H_2CONH$), 40.2 ($C_6$), 50.4 ($C_2$), 53.1 ($NH\underline{C}H_2$), 53.4 ($NH\underline{C}H_2CH$), 53.8 ($C_1$), 55.6 ($OCH_3$), 114.8 ($C_{3'}$, $C_{5'}$), 127.7 ($C_4$), 130.4 ($C_{2'}$, $C_{6'}$), 134.4 ($C_{1'}$), 137.4 ($C_5$), 160.1 ($C_{4'}$), 174.3 (CONH), 175.2 (COOH). HPLC (method A, $t_R$, min): 19.70. MS (ESI, *m/z*, %): 415.2 ($[M+H]^+$, 100). Elemental analysis calculated for $C_{24}H_{34}N_2O_4\cdot HCl\cdot 7/2H_2O$: %C 56.08, %H 8.24, %N 5.45; experimental: %C 56.20, %H 7.86, %N 5.57.

**Methyl 5-{[(1*R*,2*S*,6*R*)-4-{[(cyclopropylmethyl)amino]methyl}-4'-methoxy-6-methyl-1,2,3,6-tetrahydro[1,1'-biphenyl]-2-yl]amino}pentanoate, 9.** Following general procedure I using **41** (30 mg, 0.06 mmol), compound **9** was obtained as a brown oil (19 mg, 75%).

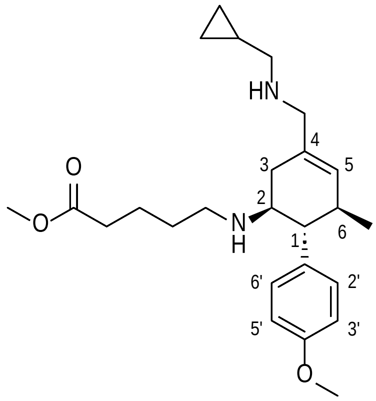

$R_f$: 0.40 (DCM/MeOH/$NH_3$ 9:1:0.1). $[\alpha]^D{}_{20}$ = -3.0 (c = 1.00, $CHCl_3$). IR (ATR): ν 1738 (C=O), 1513 (C-N), 1249 (COC). $^1$H-NMR ($CDCl_3$): δ 0.12-0.19 (m, 2H, $CH_{2cpr}$), 0.47-0.57 (m, 2H, $CH_{2cpr}$), 0.80 (d, *J* = 6.8, 3H, $CH_3$), 0.95-1.07 (m, 1H, $CH_{cpr}$), 1.24-1.38 (m, 2H, $C\underline{H}_2CH_2NH$), 1.44 (quint, 2H, $COCH_2C\underline{H}_2$), 1.89-2.07 (br m, 3H, $H_1$, 2NH), 2.15-2.25 (m, 3H, $H_3$, $COCH_2$), 2.27-2.46 (m, 3H, $H_3$, $H_6$, 1/2$CH_2C\underline{H}_2NH$), 2.49 (d, *J* = 6.9, $NHC\underline{H}_2CH$), 2.55-2.68 (m, 1H, 1/2$CH_2C\underline{H}_2NH$), 2.96 (td, *J* = 10.3, 5.2, 1H, $H_2$), 3.25 (s, 2H, $NHC\underline{H}_2$), 3.63 (s, 3H, $COOCH_3$), 3.81 (s, 3H, $OCH_3$), 5.50 (s, 1H, $H_5$), 6.87 (d, *J* = 8.6, 2H, $H_{3'}$, $H_{5'}$), 7.12 (d, *J* = 8.6, 2H, $H_{2'}$, $H_{6'}$). $^{13}$C-NMR ($CDCl_3$): δ 3.66 ($CH_{2cpr}$), 3.67 ($CH_{2cpr}$), 11.0 ($CH_{cpr}$), 20.1 ($CH_3$), 22.6 ($COCH_2\underline{C}H_2$), 29.4 ($\underline{C}H_2CH_2NH$), 33.9 ($CO\underline{C}H_2CH_2$), 34.1 ($C_3$), 38.2 ($C_6$), 46.6 ($CH_2\underline{C}H_2NH$), 51.6 ($COO\underline{C}H_3$), 54.2 ($NH\underline{C}H_2CH$, $CO\underline{C}H_2$, $C_1$), 55.2 ($NHCH_2$), 55.4 ($OCH_3$), 57.8 ($C_2$), 114.3 ($C_{3'}$, $C_{5'}$), 129.1 ($C_5$), 129.4 ($C_{2'}$, $C_{6'}$), 133.9 ($C_{1'}$, $C_4$), 158.6 ($C_{4'}$), 174.0 ($\underline{C}OOCH_3$). HPLC (method A, $t_R$, min): 11.13. MS (ESI, *m/z*, %): 415.3 ($[M+H]^+$, 100). Elemental analysis calculated for $C_{25}H_{38}N_2O_3 \cdot 2HCl \cdot 2H_2O$: %C 57.36, %H 8.47, %N 5.35; experimental: %C 57.02, %H 8.06, %N 4.97.

**5-{[(1*R*,2*S*,6*R*)-4-{[(Cyclopropylmethyl)amino]methyl}-4'-methoxy-6-methyl-1,2,3,6-tetrahydro[1,1'-biphenyl]-2-yl]amino}pentanoic acid, 10.** Following general procedure I using **42** (13 mg, 0.03 mmol), compound **10** was obtained as a colorless oil (6 mg, 59%).

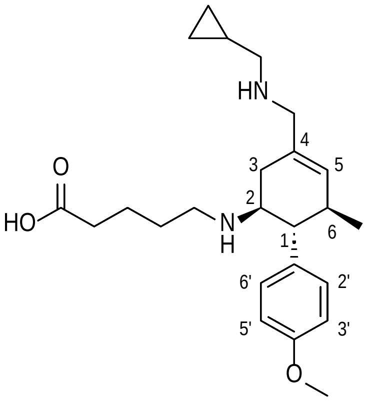


$R_f$: 0.40 (DCM/MeOH 8:2). $[\alpha]^D_{20}$ = +5.0 (c = 1.00, $CHCl_3$). IR (ATR): ν 3402 (OH, NH), 1716 (C=O), 1514 (C-N), 1253 (COC). $^1$H-NMR ($CDCl_3$): δ 0.42-0.53 (m, 2H, $CH_{2cpr}$), 0.69-0.78 (m, 2H, $CH_{2cpr}$), 0.87 (d, *J* = 6.4, 3H, $CH_3$), 1.14-1.31 (m, 1H, $CH_{cpr}$), 1.48-1.71 (m, 4H, $COCH_2C\underline{H}_2$, $C\underline{H}_2CH_2NH$), 2.28 (t, *J* = 6.8, 2H, $COCH_2$), 2.45-2.62 (m, 3H, $H_1$, $H_3$, $H_6$), 2.79-2.93 (m, 3H, $H_3$, $CH_2C\underline{H}_2NH$), 2.97(d, *J* = 7.4, 2H, $NHC\underline{H}_2CH$), 3.71 (s, 2H, $NHCH_2$), 3.82 (s, 4H, $OCH_3$, $H_2$), 5.95 (s, 1H, $H_5$), 7.01 (d, *J* = 8.6, 2H, $H_{3'}$, $H_{5'}$), 7.32 (d, *J* = 8.6, 2H, $H_{2'}$, $H_{6'}$). $^{13}$C-NMR ($CDCl_3$): δ 4.8 ($2CH_{2cpr}$), 8.1 ($CH_{cpr}$), 19.1 ($CH_3$), 22.7 ($COCH_2\underline{C}H_2$), 26.0 ($\underline{C}H_2CH_2NH$), 31.4 ($C_3$), 33.9 ($CO\underline{C}H_2$), 39.6 ($C_6$), 46.5 ($CH_2\underline{C}H_2NH$), 51.3 ($C_1$), 52.8 ($NHCH_2$), 53.5 ($NH\underline{C}H_2CH$), 55.8 ($OCH_3$), 58.1 ($C_2$), 116.1 ($C_{3'}$, $C_{5'}$), 125.8 ($C_4$), 130.8 ($C_{1'}$), 130.9 ($C_{2'}$, $C_{6'}$), 137.5 ($C_5$), 161.3 ($C_{4'}$), 176.8 (COOH). HPLC (method A, $t_R$, min): 10.87. MS (ESI, *m/z*, %): 401.3 ($[M+H]^+$, 100). Elemental analysis calculated for $C_{24}H_{36}N_2O_3{\cdot}2HCl{\cdot}9/2H_2O$: %C 51.98, %H 8.54, %N 5.05; experimental: %C 51.87, %H 8.15, %N 4.88.

***N*-[(1*R*,2*S*,6*R*)-4-{[(Cyclopropylmethyl)amino]methyl}-4'-methoxy-6-methyl-1,2,3,6-tetrahydro[1,1'-biphenyl]-2-yl]guanidine, 11.** Following general procedure I using **43** (57 mg, 0.09 mmol), compound **11** was obtained as a beige solid (15 mg, 49%).

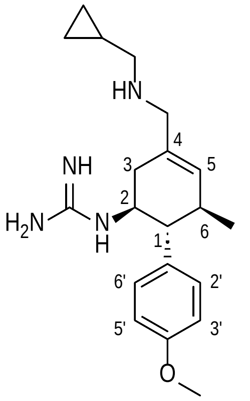

$R_f$: 0.40 (DCM/MeOH/$NH_3$ 8:2:0.1). $[\alpha]^D{}_{20}$ = -22.0 (c = 1.00, $CHCl_3$). IR (ATR): ν 3375 (NH), 1669 (C=N), 1515 (C-N). $^1$H-NMR ($CDCl_3$): δ 0.43-0.50 (m, 2H, $CH_{2cpr}$), 0.69-0.77 (m, 2H, $CH_{2cpr}$), 0.91 (d, *J* = 6.9, 3H, $CH_3$), 1.10-1.22 (m, 1H, $CH_{cpr}$), 2.23-2.35 (m, 1H, $H_3$), 2.40 (t, *J* = 10.5, 1H, $H_1$), 2.54-2.69 (m, 2H, $H_3$, $H_6$), 2.95 (dd, *J* = 7.5, 1.8, 2H, NHC$\underline{H}_2$CH), 3.66 (s, 2H, NHC$\underline{H}_2$), 3.77 (s, 3H, $OCH_3$), 4.00-4.12 (m, 1H, $H_2$), 5.90 (s, 1H, $H_5$), 6.89 (d, *J* = 6.9, 2H, $H_{3'}$, $H_{5'}$), 7.20 (d, *J* = 7.2, 2H, $H_{2'}$, $H_{6'}$). $^{13}$C-NMR ($CDCl_3$): δ 4.73 ($CH_{2cpr}$), 4.75 ($CH_{2cpr}$), 8.1 ($CH_{cpr}$), 19.6 ($CH_3$), 35.5 ($C_3$), 39.5 ($C_6$), 53.1 (NH$\underline{C}H_2$CH), 53.4 ($NHCH_2$), 53.6 ($C_1$), 54.0 ($C_2$), 55.7 ($OCH_3$), 115.1 ($C_{3'}$, $C_{5'}$), 127.0 ($C_4$), 130.4 ($C_{2'}$, $C_{6'}$), 133.4 ($C_{1'}$), 137.4 ($C_5$), 157.8 ($C_{4'}$), 160.3 (C=NH). HPLC (method A, $t_R$, min): 12.18. MS (ESI, *m/z*, %): 343.2 ($[M+H]^+$, 100). Elemental analysis calculated for $C_{20}H_{30}N_4O{\cdot}4HCl{\cdot}H_2O$: %C 47.44, %H 7.17, %N 11.07; experimental: %C 47.29, %H 6.78, %N 10.68.

***N*-[(1*R*,2*S*,6*R*)-4-{[(Cyclopropylmethyl)amino]methyl}-4'-methoxy-6-methyl-1,2,3,6-tetrahydro[1,1'-biphenyl]-2-yl]-L-tryptophanamide, 12.** Following general procedure I using **44** (15 mg, 0.02 mmol), compound **12** was obtained as a white solid (7 mg, 66%).

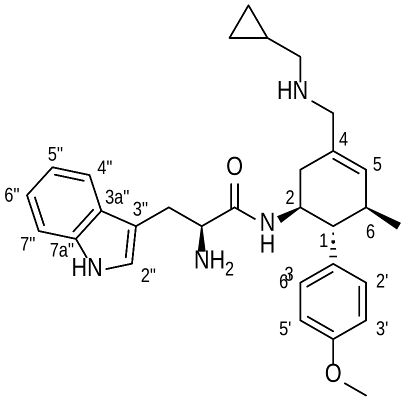


$R_f$: 0.40 (DCM/MeOH/$NH_3$ 8:2:0.1). $[\alpha]^D{}_{20}$ = -60.0 (c = 1.00, $CHCl_3$). IR (ATR): ν 3191 ($NH_2$), 1664 (C=O), 1512 (C-N), 1248 (COC). $^1$H-NMR (MeOH-*d4*): δ 0.41-0.51 (m, 2H, $CH_{2cpr}$), 0.69-0.80 (m, 2H, $CH_{2cpr}$), 0.93 (d, *J* = 6.9, 3H, $CH_3$), 1.10-1.23 (m, 1H, $CH_{cpr}$), 2.25-2.68 (m, 6H, $H_1$, 2$H_3$, $H_6$, C$\underline{H}_2$CHNH$_2$), 2.96 (d, *J* = 7.4, 2H, NHC$\underline{H}_2$CH), 3.62 (s, 3H, $OCH_3$), 3.68 (s, 2H, NHC$\underline{H}_2$), 3.91 (dd, *J* = 10.8, 3.9, 1H, CH$_2$C$\underline{H}$NH$_2$), 4.45 (td, *J* = 10.9, 5.4, 1H, $H_2$), 5.91 (s, 1H, $H_5$), 6.87 (d, *J* = 8.7, 2H, $H_{3'}$, $H_{5'}$), 6.96 (s, 1H, $H_{2''}$), 7.06 (t, *J* = 8.1, 1H, $H_{6''}$), 7.13 (t, *J* = 7.5, 1H, $H_{5''}$), 7.23 (d, *J* = 8.7, 2H, $H_{2'}$, $H_{6'}$), 7.36 (d, *J* = 8.0, 1H, $H_{7''}$), 7.55 (d, *J* = 7.7, 1H, $H_{4''}$). $^{13}$C-NMR (MeOH-*d4*): δ 4.7 (2$CH_{2cpr}$), 8.1 ($CH_{cpr}$), 19.8 ($CH_3$), 28.6 ($\underline{C}H_2$CHNH$_2$), 35.5 ($C_3$), 39.9 ($C_6$), 50.9 ($C_2$), 53.1 ($NHCH_2$),

53.4 (NH$\underline{C}H_2$CH), 54.3 ($C_1$), 54.6 ($CH_2\underline{C}HNH_2$), 55.7 ($OCH_3$), 108.0 ($C_{3''}$), 112.5 ($C_{7''}$), 115.1 ($C_{3'}$, $C_{5'}$), 119.1 ($C_{4''}$), 120.2 ($C_{6''}$), 122.9 ($C_{5''}$), 125.1 ($C_{2''}$), 127.8 ($C_4$), 128.0 ($C_{7a''}$) 130.6 ($C_{2'}$, $C_{6'}$), 134.4 ($C_{1'}$), 137.3 ($C_5$), 138.3 ($C_{3a''}$), 160.3 ($C_{4'}$), 169.4 (CO). HPLC (method A, $t_R$, min): 10.83. MS (ESI, *m/z*, %): 487.2 ($[M+H]^+$, 100). Elemental analysis calculated for $C_{30}H_{38}N_4O_2{\cdot}2HCl{\cdot}5/2H_2O$: %C 59.60, %H 7.50, %N 9.27; experimental: %C 59.51, %H 7.10, %N 8.86.

## 2.5. Synthesis of final compounds 13-17 (Scheme 2)

**(1*R*,2*R*,6*R*)-6-[(1*H*-Indol-3-yl)methyl]-4'-methoxy-2-nitro-1,2,3,6-tetrahydro[1,1'-biphenyl]-4-carbaldehyde, 45.** Following general procedure B using *trans*-*p*-methoxy-*β*-nitrostyrene (330 mg, 1.84 mmol) and indole (259 mg, 2.21 mmol), compound **45** was obtained as a yellow solid (355 mg, 49%). Chromatography: hexane to hexane/EtOAc 7:3.

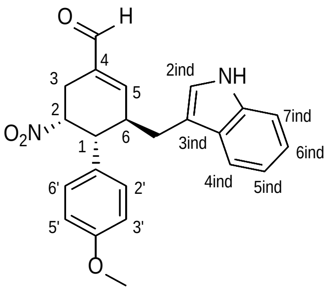


Mp: 132-133 ºC. $R_f$: 0.29 (hexane/EtOAc 6:4). $[\alpha]^D_{20}$ = -57.8 (c = 1.00, $CHCl_3$). $^1$H-NMR ($CDCl_3$): δ 2.78 (d, *J* = 5.8, 2H, $2H_3$), 2.86 (dd, *J* = 14.5, 8.6, 1H, 1/2$CH_2$), 3.12 (dd, *J* = 14.5, 5.2, 1H, 1/2$CH_2$), 3.38-3.41 (m, 1H, $H_1$), 3.41-3.51 (m, 1H, $H_6$), 3.80 (s, 3H, $CH_3$), 4.87-4.92 (m, 1H, $H_2$), 6.87 (d, *J* = 8.7, 2H, $H_{3'}$, $H_{5'}$), 7.02-7.07 (m, 2H, $H_5$, $H_{2ind}$), 7.05 (d, *J* = 8.8, 2H, $H_{2'}$, $H_{6'}$), 7.08-7.14 (m, $H_{5ind}$), 7.19-7.24 (m, 1H, $H_{6ind}$), 7.39 (d, *J* = 8.1, 1H, $H_{7ind}$), 7.45 (d, *J* = 8.1, 1H, $H_{4ind}$), 8.11 (br s, 1H, NH), 9.49 (s, 1H, CHO). $^{13}$C-NMR ($CDCl_3$): δ 24.4 ($C_3$), 29.7 ($CH_2$), 40.8 ($C_6$), 46.9 ($C_1$), 55.4 ($CH_3$), 84.0 ($C_2$), 111.5 ($C_{7ind}$), 112.2 ($C_{3ind}$), 114.6 ($C_{3'}$, $C_{5'}$), 118.8 ($C_{4ind}$), 119.8 ($C_{5ind}$), 122.6, 122.8 ($C_{2ind}$, $C_{6ind}$), 127.4 ($C_4$), 129.0 ($C_{2'}$, $C_{6'}$), 129.5 ($C_{1'}$), 136.4 ($C_{ind}$), 136.5 ($C_{ind}$), 153.0 ($C_5$), 159.6 ($C_{4'}$), 192.6 (CHO).

**(1*R*,2*R*,6*R*)-4'-Methoxy-2-nitro-6-{[(1,3-thiazol-2-yl)methoxy]methyl}-1,2,3,6-tetrahydro[1,1'-biphenyl]-4-carbaldehyde, 46.** Following general procedure B using *trans*-*p*-methoxy-*β*-nitrostyrene (418 mg, 2.30 mmol) and (1,3-thiazol-2-yl)methanol

(463 mg, 2.76 mmol), compound **46** was obtained as a yellow solid (487 mg, 54%). Chromatography: DCM to DCM/EtOH 9.5:0.5.

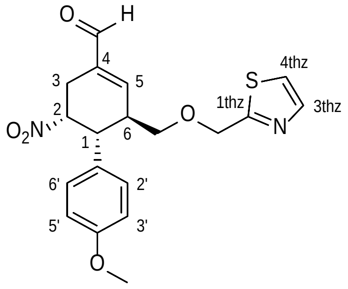


$R_f$: 0.40 (DCM/EtOH 9.5:0.5). $[\alpha]^D_{20}$ = -61.2 (c = 0.92, $CHCl_3$). IR (ATR): ν 1683 (C=O), 1548 ($NO_2$), 1253 (COC). $^1$H-NMR ($CDCl_3$): δ 2.80-2.84 (m, 2H, $2H_3$), 3.28-3.35 (m, 1H, $H_6$), 3.52 (dd, *J* = 7.0, 4.1, 1H, $H_1$), 3.63 (dd, *J* = 9.2, 5.0, 1H, 1/2O$CH_2$), 3.76-3.81 (m, 1H, 1/2O$CH_2$), 3.77 (s, 3H, $CH_3$), 4.77 (AB system, *J* = 13.6, 2H, $CH_2C_{thz}$), 4.98-5.03 (m, 1H, $H_2$), 6.83 (d, *J* = 8.8, 2H, $H_{3'}$, $H_{5'}$), 6.98 (d, *J* = 8.8, 2H, $H_{2'}$, $H_{6'}$), 7.02-7.03 (m, 1H, $H_5$), 7.35 (d, *J* = 3.3, 1H, $H_{4thz}$), 7.73 (d, *J* = 3.3, 1H, $H_{3thz}$), 9.60 (s, 1H, CHO). $^{13}$C-NMR ($CDCl_3$): δ 24.2 ($C_3$), 40.8 ($C_6$), 43.8 ($C_1$), 55.3 ($CH_3$), 70.1 ($\underline{C}H_2C_{thz}$), 71.9 (O$CH_2$), 84.2 ($C_2$), 114.5 ($C_{3'}$, $C_{5'}$), 120.0 ($C_{4thz}$), 128.8 ($C_{2'}$, $C_{6'}$), 129.0 ($C_{1'}$), 137.8 ($C_4$), 142.6 ($C_{3thz}$), 150.2 ($C_5$), 159.4 ($C_{4'}$), 167.8 ($C_{1thz}$), 192.4 (CHO). 1D $^1$H-NMR NOE: irradiation of the signal at 4.98-5.03 ppm (m, $H_2$) yielded NOE on 2.80-2.84 (m, $2H_3$), and 3.52 (dd, $H_1$). HPLC (method A, $t_R$, min): 22.85. MS (ESI, *m/z*, %): 389.1 ([M+H]$^+$, 100).

**(1*R*,2*R*,6*R*)-6-{[(1,3-Benzothiazol-2-yl)methoxy]methyl}-4'-methoxy-2-nitro-1,2,3,6-tetrahydro[1,1'-biphenyl]-4-carbaldehyde, 47.** Following general procedure B using *trans*-*p*-methoxy-*β*-nitrostyrene (100 mg, 0.56 mmol) and (1,3-benzothiazol-2-yl)methanol (110 mg, 0.67 mmol), compound **47** was obtained as a yellow oil (125 mg, 51%). Chromatography: hexane to hexane/EtOAc 6:4.

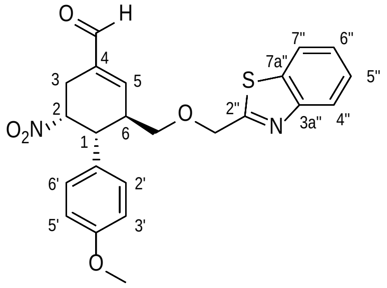


$R_f$: 0.40 (hexane/EtOAc 1:1). $[\alpha]^D_{20}$ = -68.0 (c = 1.0, $CHCl_3$). IR (ATR): ν 1684 (C=O), 1550 ($NO_2$), 1513 (C-N), 1248 (COC). $^1$H-NMR ($CDCl_3$): δ 2.82-2.87 (m, 2H, $2H_3$), 3.32-3.39 (m, 1H, $H_6$), 3.56 (dd, *J* = 7.2, 3.9, 1H, $H_1$), 3.68 (dd, *J* = 9.2, 4.9, 1H, 1/2OC$\underline{H}_2$CH), 3.76 (s, 3H, $CH_3$), 3.84 (dd, *J* = 9.1, 4.0, 1H, 1/2OC$\underline{H}_2$CH), 4.87 (AB system, *J* = 13.8,

2H, $OCH_2$), 4.97-5.09 (m, 1H, $H_2$), 6.79 (d, *J* = 8.8 , 2H, $H_{3'}$, $H_{5'}$), 6.99 (d, *J* = 8.7, 2H, $H_{2'}$, $H_{6'}$), 7.04-7.09 (m, 1H, $H_5$), 7.41 (ddd, *J* = 8.4, 7.3, 1.3, 1H, $H_{6''}$), 7.49 (ddd, *J* = 8.2, 7.2, 1.4, 1H, $H_{5''}$), 7.88-7.94 (m, 1H, $H_{7''}$), 7.95-8.00 (m, 1H, $H_{4''}$), 9.61 (s, 1H, CHO). $^{13}$C-NMR ($CDCl_3$): δ 24.5 ($C_3$), 40.8 ($C_6$), 43.8 ($C_1$), 55.3 ($CH_3$), 70.8 ($OCH_2$), 72.2 (O$\underline{C}H_2$CH), 84.3 ($C_2$), 114.2 ($C_{3'}$, $C_{5'}$), 122.0 ($C_{7''}$), 123.2 ($C_{4''}$), 125.5 ($C_{6''}$), 126.4 ($C_{5''}$), 128.9 ($C_{2'}$, $C_{6'}$), 129.0 ($C_{1'}$), 135.0 ($C_{7a''}$), 138.0 ($C_4$), 150.2 ($C_5$), 152.9 ($C_{3a''}$), 159.5 ($C_{4'}$), 169.1 ($C_{2''}$), 192.4 (CHO). HPLC (method A, $t_R$, min): 16.93. MS (ESI, *m/z*, %): 439.0 ($[M+H]^+$, 100).

**(1*R*,2*R*,6*R*)-4'-Methoxy-2-nitro-6-{[(pyridin-2-yl)methoxy]methyl}-1,2,3,6-tetrahydro[1,1'-biphenyl]-4-carbaldehyde, 48.** Following general procedure B using *trans-p*-methoxy-*β*-nitrostyrene (150 mg, 0.84 mmol) and (pyridin-2-yl)methanol (97 µL, 1.01 mmol), compound **48** was obtained as a brown oil (120 mg, 38%). Chromatography: DCM to DCM/MeOH 9.5:0.5.

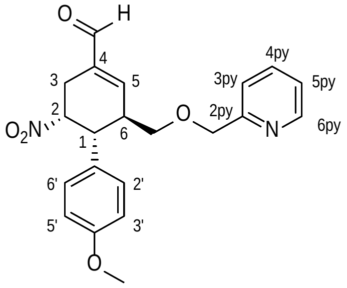


$R_f$: 0.40 (DCM/MeOH 9.5:0.5). $[\alpha]^D_{20}$ = -44.0 (c = 1.0, $CHCl_3$). IR (ATR): ν 1685 (C=O), 1550 ($NO_2$), 1514 (C-N), 1250 (COC). $^1$H-NMR ($CDCl_3$): δ 2.72-2.87 (m, 2H, 2$H_3$), 2.87-3.00 (m, 1H, $H_6$), 3.39 (dd, *J* = 9.2, 6.2, 1H, $H_1$), 3.54 (dd, *J* = 6.8, 4.0, 1H, 1/2OC$\underline{H}_2$CH), 3.72-3.77 (m, 4H, 1/2OC$\underline{H}_2$CH, $CH_3$), 4.55 (AB system, *J* = 13.7, 2H, $OCH_2$), 4.96-5.04 (m, 1H, $H_2$), 6.83 (d, *J* = 8.7, 2H, $H_{3'}$, $H_{5'}$), 6.94-7.01 (m, 1H, $H_5$), 7.08 (d, *J* = 8.7, 2H, $H_{2'}$, $H_{6'}$), 7.18-7.25 (m, 1H, $H_{5py}$), 7.34 (d, *J* = 7.9, 1H, $H_{3py}$), 7.70 (td, *J* = 7.7, 1.8, 1H, $H_{4py}$), 8.52 (d, *J* = 4.8, 1H, $H_{6py}$), 9.56 (d, *J* = 1.1, 1H, CHO). $^{13}$C-NMR ($CDCl_3$): δ 23.9 ($C_3$), 43.9 ($C_6$), 45.6 ($C_1$), 55.3 ($CH_3$), 71.9 (O$\underline{C}H_2$CH), 74.1 ($OCH_2$), 87.4 ($C_2$), 114.6 ($C_{3'}$, $C_{5'}$), 121.6 ($C_{3py}$), 122.9 ($C_{5py}$), 128.9 ($C_{2'}$, $C_{6'}$), 129.0 ($C_{1'}$), 137.2 ($C_{4py}$), 137.8 ($C_4$), 149.0 ($C_{6py}$), 150.2 ($C_5$), 157.6 ($C_{2py}$), 159.5 ($C_{4'}$), 192.4 (CHO). HPLC (method A, $t_R$, min): 13.64. MS (ESI, *m/z*, %): 439.0 ($[M+H]^+$, 100).

**(1*R*,2*R*,6*R*)-4'-Methoxy-6-{[2-(morpholin-4-yl)ethoxy]methyl}-2-nitro-1,2,3,6-tetrahydro[1,1'-biphenyl]-4-carbaldehyde, 49.** Following general procedure B using

*trans-p*-methoxy-*β*-nitrostyrene (150 mg, 0.84 mmol) and 2-(morpholin-4-yl)ethan-1-ol (0.12 mL, 1.01 mmol), compound **49** was obtained as a brown oil (95 mg, 28%). Chromatography: DCM to DCM/EtOH 9:1.

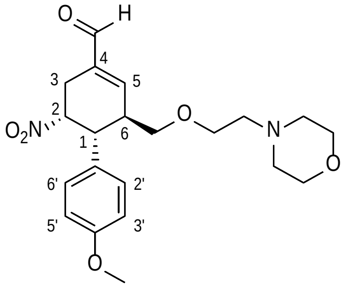


$R_f$: 0.30 (DCM/EtOH 9:1). $[\alpha]^D_{20}$ = 4.0 (c = 1.0, $CHCl_3$). IR (ATR): ν 1683 (C=O), 1550 ($NO_2$), 1514 (C-N), 1253 (COC). $^1$H-NMR ($CDCl_3$): δ 2.49-2.56 (m, 6H, 2$NCH_{2morp}$, $OCH_2C\underline{H}_2N$), 2.71-2.84 (m, 2H, 2$H_3$), 2.84-2.93 (m, 1H, $H_6$), 3.20-3.30 (m, 2H, $H_1$, 1/2$OC\underline{H}_2CH$), 3.42-3.99 (m, 3H, 1/2$OC\underline{H}_2CH$, $OC\underline{H}_2CH_2N$), 3.72 (t, *J* = 4.7, 4H, 2$OCH_{2morp}$), 3.78 (s, 3H, $CH_3$), 4.87-4.96 (m, 1H, $H_2$), 6.86 (d, *J* = 8.7, 2H, $H_{3'}$, $H_{5'}$), 6.92-6.98 (m, 1H, $H_5$), 7.11 (d, *J* = 8.7, 2H, $H_{2'}$, $H_{6'}$), 9.56 (s, 1H, CHO). $^{13}$C-NMR ($CDCl_3$): δ 27.8 ($C_3$), 44.4 ($C_6$), 45.7 ($C_1$), 54.1 (2$NCH_{2morp}$), 55.4 ($CH_3$), 58.0 ($OCH_2\underline{C}H_2N$), 66.7 (2$OCH_{2morp}$), 68.8 ($O\underline{C}H_2CH_2N$), 70.7 ($O\underline{C}H_2CH$), 87.4 ($C_2$), 114.6 ($C_{3'}$, $C_{5'}$), 128.9 ($C_{1'}$), 129.0 ($C_{2'}$, $C_{6'}$), 137.5 ($C_4$), 150.2 ($C_5$), 159.5 ($C_{4'}$), 191.9 (CHO). HPLC (method A, $t_R$, min): 11.69. MS (ESI, *m/z*, %): 405.1 ($[M+H]^+$, 100).

**1-Cyclopropyl-*N*-({(1*R*,2*S*,6*R*)-6-[(1*H*-indol-3-yl)methyl]-4'-methoxy-2-nitro-1,2,3,6-tetrahydro[1,1'-biphenyl]-4-yl}methyl)methanamine, 50.** Following general procedure C using **45** (450 mg, 1.15 mmol) and (cyclopropylmethyl)amine (0.20 mL, 2.30 mmol), compound **51** was obtained as a yellow oil (420 mg, 82%), which was used in the next step without further purification.

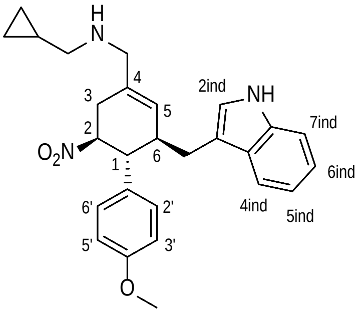


$R_f$: 0.33 (DCM/MeOH 9:1). $[\alpha]^D_{20}$ = -21.4 (c = 0.54, $CHCl_3$). $^1$H-NMR ($CDCl_3$): δ 0.04-0.09 (m, 2H, $CH_{2cpr}$), 0.43-0.49 (m, 2H, $CH_{2cpr}$), 0.83-0.96 (m, 1H, $CH_{cpr}$), 2.35 (d, *J* = 6.8, 2H, $NHC\underline{H}_2CH$), 2.42 (dd, *J* = 13.9, 9.9, 1H, 1/2$CH_2C_{ind}$), 2.71-2.84 (m, 3H, $H_6$,

$2H_3$), 2.90 (dd, *J* = 14.1, 2.6, 1H, 1/2$CH_2C_{ind}$), 3.07 (dd, *J* = 11.3, 10.3, 1H, $H_1$), 3.18 (s, 2H, NHC$\underline{H}_2$), 3.82 (s, 3H, $CH_3$), 4.96 (ddd, *J* = 11.4, 9.8, 6.3, 1H, $H_2$), 5.67 (s, 1H, $H_5$), 6.92 (d, *J* = 8.7, 2H, $H_{3'}$, $H_{5'}$), 6.91-6.95 (m, 1H, $H_{2ind}$), 7.01-7.06 (m, 1H, $H_{6ind}$), 7.13-7.24 (m, 2H, $H_{5ind}$, $H_{7ind}$), 7.24 (d, *J* = 8.7, 2H, $H_{2'}$, $H_{6'}$), 7.33 (d, *J* = 8.1, 2H, $H_{4ind}$), 8.01 ($NH_{ind}$). $^{13}$C-NMR ($CDCl_3$): δ 3.5 ($2CH_{2cpr}$), 11.3 ($CH_{cpr}$), 28.8 ($\underline{C}H_2C_{ind}$), 33.2 ($C_3$), 43.1 ($C_6$), 50.1 ($C_1$), 54.5 (NH$\underline{C}H_2$CH), 55.0 ($NHCH_2$), 55.4 ($CH_3$), 89.11 ($C_2$), 111.2 ($C_{4ind}$), 113.5 ($C_{3ind}$), 114.4 ($C_{3'}$, $C_{5'}$), 119.0 ($C_{7ind}$), 119.4 ($C_{6ind}$), 122.14 ($C_{5ind}$), 122.5 ($C_{2ind}$), 126.2 ($C_5$), 127.6 ($C_{7a\ ind}$), 129.4 ($C_{2'}$, $C_{6'}$), 130.7 ($C_{1'}$), 132.2 ($C_4$), 136.4 ($C_{3a\ ind}$), 159.2 ($C_{4'}$). HPLC (method A, $t_R$, min): 19.50. MS (ESI, *m/z*, %): 446.3 ($[M+H]^+$, 100).

**1-Cyclopropyl-*N*-{[(1*R*,2*S*,6*R*)-4'-methoxy-2-nitro-6-{[(1,3-thiazol-2-yl)methoxy] methyl}-1,2,3,6-tetrahydro[1,1'-biphenyl]-4-yl]methyl}methanamine, 51.** Following general procedure C using **46** (237 mg, 0.61 mmol) and (cyclopropylmethyl)amine (0.11 mL, 1.22 mmol), compound **51** was obtained as a yellow oil (206 mg, 76%), which was used in the next step without further purification.

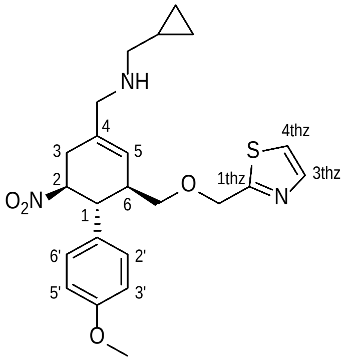


$R_f$: 0.11 (DCM/EtOH 9.5:0.5). $[\alpha]^D_{20}$ = -29.2 (c = 1.03, $CHCl_3$). IR (ATR): ν 1550 ($NO_2$), 1513 (C-N), 1251 (COC). $^1$H-NMR ($CDCl_3$): δ 0.21-0.26 (m, 2H, $CH_{2cpr}$), 0.54-0.60 (m, 2H, $CH_{2cpr}$), 1.03-1.11 (m, 1H, $CH_{cpr}$), 2.61 (d, *J* = 7.0, 2H, NHC$\underline{H}_2$CH), 2.65-2.73 (m, 1H, $H_6$), 2.82-2.89 (m, 2H, $2H_3$), 3.25 (t, *J* = 11.3, 1H, $H_1$), 3.30 (dd, *J* = 8.9, 5.8, 1H, 1/2OC$\underline{H}_2$CH), 3.42 (s, 2H, NHC$\underline{H}_2$), 3.48 (dd, *J* = 8.9, 3.2, 1H, 1/2OC$\underline{H}_2$CH), 3.76 (s, 3H, $CH_3$), 4.69 (AB system, *J* = 13.5, 2H, $CH_2C_{thz}$), 5.02 (ddd, *J* = 11.5, 10.1, 6.2, 1H, $H_2$), 5.89 (s, 1H, $H_5$), 6.81 (d, *J* = 8.6, 2H, $H_{3'}$, $H_{5'}$), 7.09 (d, *J* = 8.6, 2H, $H_{2'}$, $H_{6'}$), 7.33 (d, *J* = 3.2, 1H, $H_{4thz}$), 7.72 (d, *J* = 3.2, 1H, $H_{3thz}$). $^{13}$C-NMR ($CDCl_3$): δ 4.0 ($2CH_{2cpr}$), 9.8 ($CH_{cpr}$), 33.1 ($C_3$), 43.5 ($C_6$), 45.5 ($C_1$), 53.3 (NH$\underline{C}H_2$CH), 53.5 ($NHCH_2$), 55.3 ($CH_3$), 70.2 ($\underline{C}H_2C_{thz}$), 71.6 ($OCH_2$), 88.3 ($C_2$), 114.4 ($C_{3'}$, $C_{5'}$), 119.7 ($C_{4thz}$), 127.8 ($C_5$), 129.1 ($C_{2'}$, $C_{6'}$), 129.6 ($C_{1'}$), 133.2 ($C_4$), 142.6 ($C_{3thz}$), 159.3 ($C_{4'}$), 168.4 ($C_{1thz}$). HPLC (method A, $t_R$, min): 17.91. MS (ESI, *m/z*, %): 444.2 ($[M+H]^+$, 100).

**1-[(1*R*,2*S*,6*R*)-6-{[(1,3-Benzothiazol-2-yl)methoxy]methyl}-4'-methoxy-2-nitro-1,2,3,6-tetrahydro[1,1'-biphenyl]-4-yl]-*N*-(cyclopropylmethyl) methanamine, 52.** Following general procedure C using **47** (125 mg, 0.29 mmol) and (cyclopropylmethyl)amine (49 µL, 0.57 mmol), compound **52** was obtained as a yellow oil (41 mg, 30%). Chromatography: DCM to DCM/EtOH/$NH_3$ 9.5:0.5:0.1.

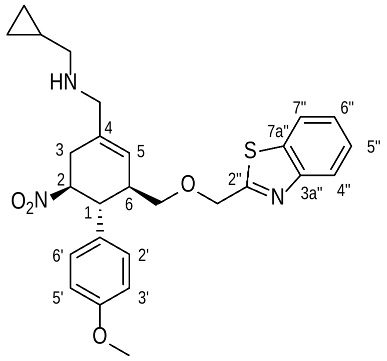


$R_f$: 0.30 (DCM/EtOH/$NH_3$ 9.5:0.5:0.1). $[\alpha]^D_{20}$ = -58.0 (c = 1.0, $CHCl_3$). IR (ATR): ν 1550 ($NO_2$), 1513 (C-N), 1249 (COC). $^1$H-NMR ($CDCl_3$): δ 0.09-0.18 (m, 2H, $CH_{2cpr}$), 0.45-0.55 (m, 2H, $CH_{2cpr}$), 0.93-1.03 (m, 1H,$CH_{cpr}$), 2.50 (dd, *J* = 6.9, 1.5, 2H, NHC$\underline{H}_2$CH), 2.63-2.75 (m, 1H, $H_6$), 2.81 (d, *J* = 7.1, 2H, $2H_3$), 3.21-3.39 (m, 4H, $NHCH_2$, 1/2OC$\underline{H}_2$CH, $H_1$), 3.52 (dd, *J* = 8.9, 3.2, 1H, 1/2OC$\underline{H}_2$CH), 3.74 (s, 3H, $CH_3$), 4.78 (AB system, *J* = 13.8, 2H, $OCH_2$), 4.91-5.06 (m, 1H, $H_2$), 5.84 (s, 1H, $H_5$), 6.77 (d, *J* = 8.7, 2H, $H_{3'}$, $H_{5'}$), 7.09 (d, *J* = 8.7, 2H, $H_{2'}$, $H_{6'}$), 7.39 (ddd, *J* = 7.8, 7.3, 1.2, 1H, $H_{6''}$), 7.47 (ddd, *J* = 8.3, 7.7, 1.3, 1H, $H_{5''}$), 7.90 (d, *J* = 7.8, 1H, $H_{7''}$), 7.96 (d, *J* = 7.5, 1H, $H_{4''}$). $^{13}$C-NMR ($CDCl_3$): δ 3.6 ($2CH_{2cpr}$), 11.1 ($CH_{cpr}$), 33.2 ($C_3$), 43.5 ($C_6$), 45.7 ($C_1$), 54.2 (NH$\underline{C}H_2$CH), 54.6 ($NHCH_2$) 55.3 ($CH_3$), 70.8 ($OCH_2$), 72.2 (O$\underline{C}H_2$CH), 88.5 ($C_2$), 114.3 ($C_{3'}$, $C_{5'}$), 121.9 ($C_{7''}$), 123.1 ($C_{4''}$), 125.0 ($C_5$), 125.3 ($C_{6''}$), 126.2 ($C_{5''}$), 129.1 ($C_{2'}$, $C_{6'}$), 129.7 ($C_{1'}$), 133.5 ($C_4$), 135.1 ($C_{7a''}$), 153.1 ($C_{3a''}$), 159.2 (C4’), 169.8 ($C_{2''}$). HPLC (method A, $t_R$, min): 12.76. MS (ESI, *m/z*, %): 494.1 ($[M+H]^+$, 100).

**1-Cyclopropyl-*N*-{[(1*R*,2*S*,6*R*)-4'-methoxy-2-nitro-6-{[(pyridin-2-yl)methoxy] methyl}-1,2,3,6-tetrahydro[1,1'-biphenyl]-4-yl]methyl} methanamine, 53.** Following general procedure C using **48** (120 mg, 0.31 mmol) and (cyclopropylmethyl)amine (54 µL, 0.63 mmol), compound **53** was obtained as a yellow oil (59 mg, 43%). Chromatography: DCM to DCM/EtOH/$NH_3$ 9:1:0.1.

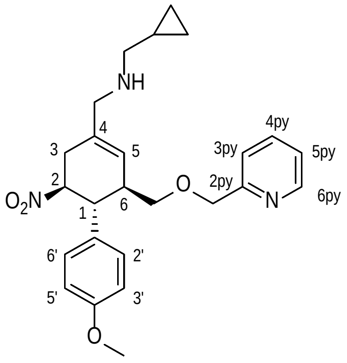

$R_f$: 0.40 (DCM/EtOH/$NH_3$ 9:1:0.1). $[\alpha]^D_{20}$ = -20.0 (c = 1.0, $CHCl_3$). IR (ATR): ν 1549 ($NO_2$), 1513 (C-N), 1249 (COC). $^1$H-NMR ($CDCl_3$): δ 0.14-0.21 (m, 2H, $CH_{2cpr}$), 0.48-0.57 (m, 2H, $CH_{2cpr}$), 0.97-1.07 (m, 1H,$CH_{cpr}$), 2.56 (d, *J* = 7.1, 1.5, 2H, NHC$\underline{H}_2$CH), 2.65-2.74 (m, 1H, $H_6$), 2.80-2.89 (m, 2H, 2$H_3$), 3.18-3.31 (m, 2H, 1/2OC$\underline{H}_2$CH, $H_1$), 3.37 (s, 2H, NHC$\underline{H}_2$), 3.44 (dd, *J* = 9.2, 3.4, 1H, 1/2OC$\underline{H}_2$CH), 3.75 (s, 3H, $CH_3$), 4.49 (AB system, *J* = 13.2, 2H, $OCH_2$), 5.01 (ddd, *J* = 11.8, 9.1, 7.1, 1H, $H_2$), 5.89 (s, 1H, $H_5$), 6.80 (d, *J* = 8.7, 2H, $H_{3'}$, $H_{5'}$), 7.08 (d, *J* = 8.7, 2H, $H_{2'}$, $H_{6'}$), 7.12-7.20 (m, 1H, $H_{5py}$), 7.32 (d, *J* = 7.8, 1H, $H_{3py}$), 7.66 (td, *J* = 7.7, 1.8, 1H, $H_{4py}$), 8.49 (dd, *J* = 4.9, 0.9, 1H, $H_{6py}$). $^{13}$C-NMR ($CDCl_3$): δ 3.9 (2$CH_{2cpr}$), 10.2 ($CH_{cpr}$), 33.1 ($C_3$), 43.5 ($C_6$), 45.8 ($C_1$), 53.6 (NHC$\underline{H}_2$CH), 53.9 ($NHCH_2$) 55.3 ($CH_3$), 71.7 (O$\underline{C}H_2$CH), 74.2 ($OCH_2$), 88.5 ($C_2$), 114.4 ($C_{3'}$, $C_{5'}$), 121.5 ($C_{3py}$), 122.5 ($C_{5py}$), 127.1 ($C_5$), 129.1 ($C_{2'}$, $C_{6'}$), 129.9 ($C_{1'}$), 131.7 ($C_4$), 136.7 ($C_{4py}$), 149.1 ($C_{6py}$), 158.3 ($C_{2py}$), 159.2 ($C_{4'}$). HPLC (method A, $t_R$, min): 11.45. MS (ESI, *m/z*, %): 438.1 ([M+H]$^+$, 100).

**1-Cyclopropyl-*N*-{[(1*R*,2*S*,6*R*)-4'-methoxy-6-{[2-(morpholin-4-yl)ethoxy] methyl}-2-nitro-1,2,3,6-tetrahydro[1,1'-biphenyl]-4-yl]methyl}methanamine, 54.** Following general procedure C using **49** (95 mg, 0.24 mmol) and (cyclopropylmethyl)amine (41 µL, 0.47 mmol), compound **54** was obtained as a yellow oil (47 mg, 44%). Chromatography: DCM to DCM/EtOH/$NH_3$ 9:1:0.1.

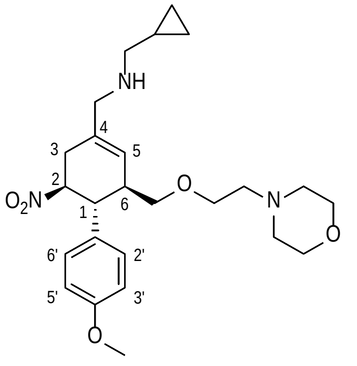

$R_f$: 0.20 (DCM/EtOH/$NH_3$ 9:1:0.1). $[\alpha]^{D}_{20}$ = -6.0 (c = 1.0, $CHCl_3$). IR (ATR): ν 1549 ($NO_2$), 1514 (C-N), 1249 (COC). $^{1}$H-NMR ($CDCl_3$): δ 0.08-0.17 (m, 2H, $CH_{2cpr}$), 0.44-0.55 (m, 2H, $CH_{2cpr}$), 0.92-1.02 (m, 1H, $CH_{cpr}$), 2.35-2.66 (m, 9H, $2NCH_{2morp}$, $OCH_2C\underline{H}_2N$, $NHC\underline{H}_2CH$, $H_6$), 2.77 (d, $J$ = 8.0, 2H, $2H_3$), 3.07-3.17 (m, 2H, $H_1$, $1/2OC\underline{H}_2CH$), 3.23-3.34 (m, 3H, $1/2OC\underline{H}_2CH$, $NHC\underline{H}_2$), 3.34-3.50 (m, 2H, $OC\underline{H}_2CH_2N$), 3.62-3.76 (m, 4H, $2OCH_{2morp}$), 3.77 (s, 3H, $CH_3$), 4.87-5.04 (m, 1H, $H_2$), 5.77 (s, 1H, $H_5$), 6.82 (d, $J$ = 8.7, 2H, $H_{3'}$, $H_{5'}$), 7.10 (d, $J$ = 8.7, 2H, $H_{2'}$, $H_{6'}$). $^{13}$C-NMR ($CDCl_3$): δ 3.6 ($2CH_{2cpr}$), 11.1 ($CH_{cpr}$), 33.1 ($C_3$), 43.2 ($C_6$), 46.0 ($C_1$), 54.2 ($2NCH_{2morp}$), 54.4 ($NH\underline{C}H_2CH$), 54.7 ($NHCH_2$), 55.3 ($CH_3$), 58.2 ($OCH_2\underline{C}H_2N$), 67.0 ($2OCH_{2morp}$), 69.0 ($O\underline{C}H_2CH_2N$), 72.1 ($O\underline{C}H_2CH$), 88.7 ($C_2$), 114.3 ($C_{3'}$, $C_{5'}$), 125.2 ($C_5$), 129.1 ($C_{2'}$, $C_{6'}$), 130.1 ($C_{1'}$), 133.0 ($C_4$), 159.1 ($C_{4'}$). HPLC (method A, $t_R$, min): 11.24. MS (ESI, *m/z*, %): 460.3 ($[M+H]^+$, 100).

**(1*R*,2*S*,6*R*)-4-{[(Cyclopropylmethyl)amino]methyl}-6-[(1*H*-indol-3-yl)methyl]-4'-methoxy-1,2,3,6-tetrahydro[1,1'-biphenyl]-2-amine, 13.** Following general procedure D using **50** (190 mg, 0.43 mmol), compound **13** was obtained as a white solid (172 mg, 95%). Chromatography: DCM to DCM/MeOH/$NH_3$ 9:1:0.1.

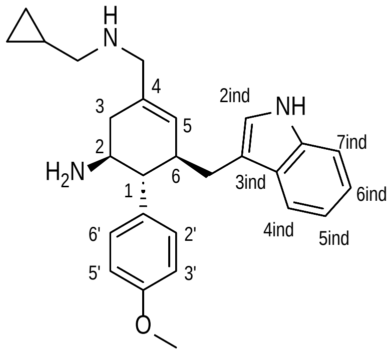


Mp: 144-145 ºC. $R_f$: 0.20 (DCM/MeOH/$NH_3$ 9:1:0.1). $[\alpha]^{D}_{20}$ = -20.8 (c = 1.06, $CHCl_3$). $^{1}$H-NMR ($CDCl_3$): δ 0.04-0.09 (m, 2H, $CH_{2cpr}$), 0.42-0.48 (m, 2H, $CH_{2cpr}$), 0.83-0.97 (m, 1H, $CH_{cpr}$), 1.92-2.01 (m, 1H, $H_3$), 2.25 (t, $J$ = 10.5, 1H, $H_1$), 2.28-2.41 (m, 2H, $1/2CH_2C_{ind}$, $H_3$), 2.37 (d, $J$ = 7.0, 2H, $NHC\underline{H}_2CH$), 2.66-2.74 (m, 1H, $H_6$), 2.81 (dd, $J$ = 14.2, 2.6, 1H, $1/2CH_2C_{ind}$), 3.16 (s, 2H, $NHC\underline{H}_2$), 3.16-3.23 (m, 1H, $H_2$), 3.84 (s, 3H, $CH_3$), 5.59 (s, 1H, $H_5$), 6.92-6.94 (m, 1H, $H_{2ind}$), 6.95 (d, $J$ = 8.6, 2H, $H_{3'}$, $H_{5'}$), 6.98-7.04 (m, 1H, $H_{6ind}$), 7.10-7.16 (m, 1H, $H_{5ind}$), 7.21-7.26 (m, 1H, $H_{7ind}$), 7.25 (d, $J$ = 8.8, 2H, $H_{2'}$, $H_{6'}$), 7.31 (d, $J$ = 8.1, 1H, $H_{4ind}$), 8.08 (s, 1H, $NH_{ind}$). $^{13}$C-NMR ($CDCl_3$): δ 3.6 ($2CH_{2cpr}$), 11.1 ($CH_{cpr}$), 29.2 ($\underline{C}H_2C_{ind}$), 36.9 ($C_3$), 43.7 ($C_6$), 52.2 ($C_2$), 54.2 ($NH\underline{C}H_2CH$), 55.3 ($NHCH_2$), 55.4 ($CH_3$), 55.5 ($C_1$), 112.1 ($C_{4ind}$), 114.36 ($C_{3'}$, $C_{5'}$), 114.42 ($C_{3ind}$),

119.07, 119.14 ($C_{6ind}$, $C_{7ind}$), 121.8 ($C_{4ind}$), 122.3 ($C_{2ind}$), 126.6 ($C_5$), 127.9 ($C_{7a\ ind}$), 129.8 ($C_{2'}$, $C_{6'}$), 133.6 ($C_4$), 134.6 ($C_{1'}$), 136.4 ($C_{3a\ ind}$), 158.63 ($C_{4'}$). HPLC (method A, $t_R$, min): 13.34. MS (ESI, *m/z*, %): 416.3 ($[M+H]^+$, 100). Elemental analysis calculated for $C_{27}H_{33}N_3O{\cdot}2HCl{\cdot}2H_2O$: %C 61.83, %H 7.49, %N 8.01; experimental: %C 62.23, %H 7.40, %N 7.68.

**(1*R*,2*S*,6*R*)-4-{[(Cyclopropylmethyl)amino]methyl}-4'-methoxy-6-{[(1,3-thiazol-2-yl)methoxy]methyl}-1,2,3,6-tetrahydro[1,1'-biphenyl]-2-amine, 14.** Following general procedure D using **51** (128 mg, 0.29 mmol), compound **14** was obtained as a yellow oil (111 mg, 93%). Chromatography: DCM to DCM/EtOH/$NH_3$ 9:1:0.1.

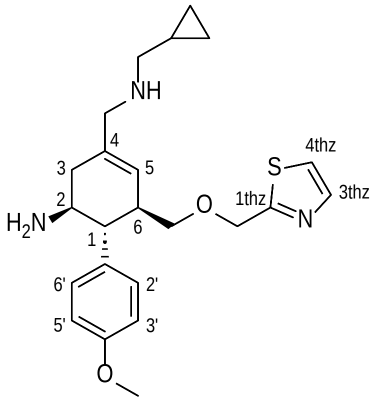


$R_f$: 0.12 (DCM/EtOH/$NH_3$ 9:1:0.1). $[\alpha]^D_{20}$ = -7.2 (c = 0.67, $CHCl_3$). IR (ATR): ν 1512 (C-N), 1249 (COC). $^1$H-NMR ($CDCl_3$): δ 0.09-0.14 (m, 2H, $CH_{2cpr}$), 0.45-0.51 (m, 2H, $CH_{2cpr}$), 0.93-1.03 (m, 1H, $CH_{cpr}$), 1.92-2.03 (m, 1H, $H_3$), 2.32 (t, *J* = 10.7, 1H, $H_1$), 2.36-2.49 (m, 1H, $H_3$), 2.46 (dd, *J* = 6.9, 1.2, 2H, NC$\underline{H}_2$CH), 2.56-2.62 (m, 1H, $H_6$), 3.15-3.29 (m, 2H, $H_2$, 1/2$OCH_2$), 3.25 (s, 2H, NHC$\underline{H}_2$), 3.42 (dd, *J* = 8.9, 3.3, 1H, 1/2$OCH_2$), 3.80 (s, 3H, $CH_3$), 4.68 (AB system, *J* = 13.6, 2H, $CH_2C_{thz}$), 5.74 (s, 1H, $H_5$), 6.86 (d, *J* = 8.6, 2H, $H_{3'}$, $H_{5'}$), 7.11 (d, *J* = 8.6, 2H, $H_{2'}$, $H_{6'}$), 7.30 (d, *J* = 3.2, 1H, $H_{4thz}$), 7.70 (d, *J* = 3.2, 1H, $H_{3thz}$). $^{13}$C-NMR ($CDCl_3$): δ 3.50 ($CH_{2cpr}$), 3.53 ($CH_{2cpr}$), 11.4 ($CH_{cpr}$), 36.9 ($C_3$), 44.1 ($C_6$), 51.2 ($C_1$), 51.9 ($C_2$), 54.4 (NH$\underline{C}H_2$CH), 55.4 ($NHCH_2$), 55.4 ($CH_3$), 70.2 ($\underline{C}H_2C_{thz}$), 73.4 ($OCH_2$), 114.3 ($C_{3'}$, $C_{5'}$), 119.4 ($C_{4thz}$), 124.3 ($C_5$), 129.4 ($C_{2'}$, $C_{6'}$), 133.8 ($C_{1'}$), 135.8 ($C_4$), 142.4 ($C_{3thz}$), 158.6 ($C_{4'}$), 169.2 ($C_{1thz}$). HPLC (method A, $t_R$, min): 14.85. MS (ESI, *m/z*, %): 413.7 ($[M]^+$, 100). Elemental analysis calculated for $C_{23}H_{31}N_3O_2S{\cdot}3HCl{\cdot}H_2O$: %C 51.06, %H 6.71, %N 7.77, %S 5.93; experimental: %C 51.47, %H 6.60, %N 7.80, %S 5.71.

**(1*R*,2*S*,6*R*)-6-{[(1,3-Benzothiazol-2-yl)methoxy]methyl}-4-{[(cyclopropylmethyl) amino]methyl}-4'-methoxy-1,2,3,6-tetrahydro[1,1'-biphenyl]-2-amine, 15.** Following general procedure D using **52** (35 mg, 0.07 mmol), compound **15** was obtained as a yellow oil (12 mg, 37%). Chromatography: DCM to DCM/EtOH/$NH_3$ 8:2:0.1.

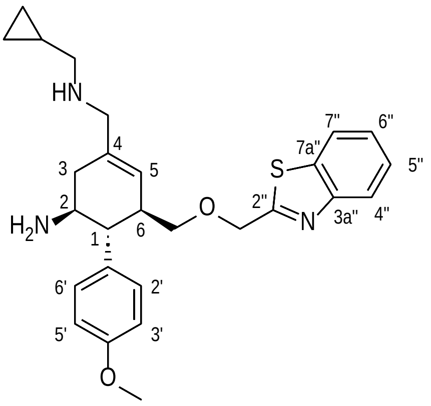


$R_f$: 0.20 (DCM/EtOH/$NH_3$ 8:2:0.1). $[\alpha]^D_{20}$ = 35.0 (c = 1.0, $CHCl_3$). IR (ATR): ν 1513 (C-N), 1248 (COC). $^1$H-NMR ($CDCl_3$): δ 0.14-0.33 (m, 2H, $CH_{2cpr}$), 0.46-0.63 (m, 2H, $CH_{2cpr}$), 1.06-1.17 (m, 1H,$CH_{cpr}$), 2.09-2.30 (m, 2H, $2H_3$), 2.51 (t, *J* = 10.3, 1H, $H_1$), 2.57-2.72 (m, 3H, NHC$\underline{H}_2$CH, $H_6$), 3.22-3.34 (m, 2H, 1/2OC$\underline{H}_2$CH, $H_2$), 3.40-3.52 (m, 3H, 1/2OC$\underline{H}_2$CH, NHC$\underline{H}_2$), 3.75 (s, 3H, $CH_3$), 4.17 (br s, 3H, NH, $NH_2$), 4.74 (AB system, *J* = 14.1, 2H, $OCH_2$), 5.90 (s, 1H, $H_5$), 6.80 (d, *J* = 8.6, 2H, $H_{3'}$, $H_{5'}$), 7.10 (d, *J* = 8.5, 2H, $H_{2'}$, $H_{6'}$), 7.34-7.42 (m, 1H, $H_{6''}$), 7.42-7.51 (m, 1H, $H_{5''}$), 7.89 (d, 1H, *J* = 7.2, $H_{7''}$), 7.95 (d, 1H, *J* = 7.5, $H_{4''}$). $^{13}$C-NMR ($CDCl_3$): δ 4.21 ($CH_{2cpr}$), 4.23 ($CH_{2cpr}$), 9.2 ($CH_{cpr}$), 34.1 ($C_3$), 44.1 ($C_6$), 49.3 ($C_1$), 51.8 ($C_2$), 52.9 (NH$\underline{C}H_2$CH), 53.7 ($NHCH_2$) 55.4 ($CH_3$), 70.7 ($OCH_2$), 73.0 (O$\underline{C}H_2$CH), 114.5 ($C_{3'}$, $C_{5'}$), 121.9 ($C_{7''}$), 123.1 ($C_{4''}$), 125.2 ($C_{6''}$), 126.2 ($C_{5''}$), 129.5 ($C_5$, $C_{2'}$, $C_{6'}$), 130.2 ($C_4$), 132.4 ($C_{1'}$), 135.1 ($C_{7a''}$), 153.1 ($C_{3a''}$), 158.9 ($C_{4'}$), 170.2 ($C_{2''}$). HPLC (method A, $t_R$, min): 12.15. MS (ESI, *m/z*, %): 464.2 ($[M]^+$, 100). Elemental analysis calculated for $C_{27}H_{33}N_3O_2S$ ·3HCl·3/2$H_2O$: %C 52.47, %H 6.69, %N 6.80, %S 5.19; experimental: %C 52.29, %H 6.22, %N 6.30, %S 4.79.

**(1*R*,2*S*,6*R*)-4-{[(Cyclopropylmethyl)amino]methyl}-4'-methoxy-6-{[(pyridin-2-yl) methoxy]methyl}-1,2,3,6-tetrahydro[1,1'-biphenyl]-2-amine, 16.** Following general procedure D using **53** (45 mg, 0.10 mmol), compound **16** was obtained as a yellow oil (21 mg, 50%). Chromatography: DCM to DCM/EtOH/$NH_3$ 8:2:0.1.

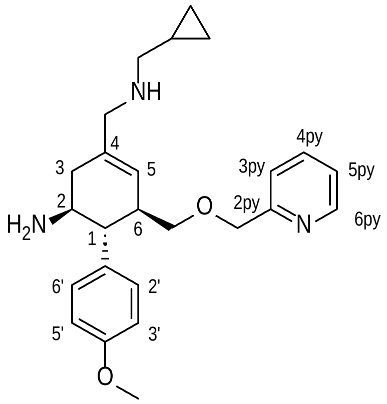

$R_f$: 0.20 (DCM/EtOH/$NH_3$ 8:2:0.1). $[\alpha]^D_{20}$ = -40.0 (c = 1.0, $CHCl_3$). IR (ATR): ν 1512 (C-N), 1248 (COC). $^1$H-NMR ($CDCl_3$): δ 0.25-0.30 (m, 2H, $CH_{2cpr}$), 0.53-0.60 (m, 2H, $CH_{2cpr}$), 1.08-1.17 (m, 1H,$CH_{cpr}$), 2.19-2.33 (m, 1H, $H_3$), 2.51-2.75 (m, 5H, $H_1$, $H_3$, $H_6$, NHC$\underline{H}_2$CH), 3.14-3.32 (m, 2H, 1/2OC$\underline{H}_2$CH, $H_2$), 3.38 (dd, $J$ = 9.1, 3.0, 1H, 1/2OC$\underline{H}_2$CH), 3.47 (s, 2H, NHC$\underline{H}_2$), 3.76 (s, 3H, $CH_3$), 4.45 (AB system, $J$ = 13.6, 2H, $OCH_2$), 4.75 (br s, 3H, $NH_2$, NH), 5.91 (s, 1H, $H_5$), 6.81 (d, $J$ = 8.4, 2H, $H_{3'}$, $H_{5'}$), 7.05-7.18 (m, 3H, $H_{5py}$, $H_{2'}$, $H_{6'}$), 7.31 (d, $J$ = 7.8, 1H, $H_{3py}$), 7.64 (td, $J$ = 7.7, 1.8, 1H, $H_{4py}$), 8.48 (dd, $J$ = 4.9, 0.8, 1H, $H_{6py}$). $^{13}$C-NMR ($CDCl_3$): δ 4.2 ($2CH_{2cpr}$), 8.7 ($CH_{cpr}$), 34.8 ($C_3$), 43.8 ($C_6$), 48.7 ($C_1$), 51.7 ($C_2$), 52.6 (NH$\underline{C}H_2$CH), 53.2 ($NHCH_2$) 55.3 ($CH_3$), 72.3 (O$\underline{C}H_2$CH), 73.9 ($OCH_2$), 114.4 ($C_{3'}$, $C_{5'}$), 121.4 ($C_{3py}$), 122.3 ($C_{5py}$), 129.4 ($C_{2'}$, $C_{6'}$), 129.5 ($C_5$), 130.8 ($C_4$), 132.4 ($C_{1'}$), 136.6 ($C_{4py}$), 148.9 ($C_{6py}$), 158.3 ($C_{2py}$), 158.7 ($C_{4'}$). HPLC (method A, $t_R$, min): 9.66. MS (ESI, *m/z*, %): 408.2 ($[M]^+$, 100). Elemental analysis calculated for $C_{25}H_{33}N_3O_2 \cdot 3HCl \cdot 5/2H_2O$: %C 53.43, %H 7.35, %N 7.48; experimental: %C 53.30, %H 6.95, %N 7.00.

**(1*R*,2*S*,6*R*)-4-{[(Cyclopropylmethyl)amino]methyl}-4'-methoxy-6-{[2-(morpholin-4-yl)ethoxy]methyl}-1,2,3,6-tetrahydro[1,1'-biphenyl]-2-amine, 17.** Following general procedure D using **54** (45 mg, 0.10 mmol), compound **17** was obtained as a yellow oil (17 mg, 40%). Chromatography: DCM to DCM/EtOH/$NH_3$ 8:2:0.1.

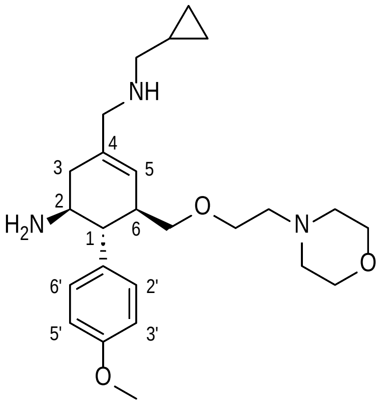


$R_f$: 0.20 (DCM/EtOH/$NH_3$ 8:2:0.1). $[\alpha]^D_{20}$ = -27.0 (c = 1.0, $CHCl_3$). IR (ATR): ν 1512 (C-N), 1248 (COC). $^1$H-NMR ($CDCl_3$): δ 0.19-0.26 (m, 2H, $CH_{2cpr}$), 0.49-0.58 (m, 2H, $CH_{2cpr}$), 1.02-1.12 (m, 1H, $CH_{cpr}$), 2.03-2.17 (m, 1H, $H_3$), 2.30-2.52 (m, 9H, $2NCH_{2morp}$, $OCH_2C\underline{H}_2N$, $H_1$, $H_3$, $H_6$), 2.57 (d, $J$ = 7.0, 2H, $NHC\underline{H}_2CH$), 3.05 (dd, $J$ = 9.1, 7.3, 1H, $1/2OC\underline{H}_2CH$), 3.14-3.26 (m, 2H, $1/2OC\underline{H}_2CH$, $H_2$), 3.30-3.46 (m, 4H, $NHC\underline{H}_2$, $OC\underline{H}_2CH_2N$), 3.62-3.70 (m, 4H, $2OCH_{2morp}$), 3.77 (s, 3H, $CH_3$), 4.01 (br s, 3H, NH, $NH_2$), 5.78 (s, 1H, $H_5$), 6.83 (d, $J$ = 8.7, 2H, $H_{3'}$, $H_{5'}$), 7.10 (d, $J$ = 8.6, 2H, $H_{2'}$, $H_{6'}$). $^{13}$C-NMR ($CDCl_3$): δ 4.0 ($2CH_{2cpr}$), 9.7 ($CH_{cpr}$), 35.6 ($C_3$), 43.6 ($C_6$), 49.8 ($C_1$), 51.8 ($C_2$), 53.3 ($NH\underline{C}H_2CH$), 54.0 ($NHCH_2$), 54.1 ($2NCH_{2morp}$), 55.3 ($CH_3$), 58.2 ($OCH_2\underline{C}H_2N$), 67.0 ($2OCH_{2morp}$), 68.6 ($O\underline{C}H_2CH_2N$), 73.0 ($O\underline{C}H_2CH$), 114.3 ($C_{3'}$, $C_{5'}$), 127.5 ($C_5$), 129.4 ($C_{2'}$, $C_{6'}$), 132.4 ($C_4$), 133.2 ($C_{1'}$), 158.7 ($C_{4'}$). HPLC (method A, $t_R$, min): 3.64. MS (ESI, *m/z*, %): 430.3 ($[M]^+$, 100). Elemental analysis calculated for $C_{25}H_{39}N_3O_3 \cdot 3HCl \cdot 7H_2O$: %C 45.15, %H 8.49, %N 6.32; experimental: %C 45.55, %H 8.10, %N 5.94.

## 2.6. Synthesis of final compounds 18-24, 26-33 (Scheme 3)

**(1*R*,2*R*,6*R*)-3'-Methoxy-6-methyl-2-nitro-1,2,3,6-tetrahydro[1,1'-biphenyl]-4-carbaldehyde**, **55.** Following general procedure A using 1-methoxy-3-[(*E*)-2-nitroethenyl]benzene (250 mg, 1.40 mmol), compound **55** was obtained as a colorless oil (205 mg, 53%). Chromatography: hexane to hexane/EtOAc 8:2.

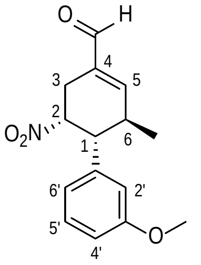

$R_f$: 0.40 (hexane/EtOAc 7:3). $[\alpha]^D_{20}$ = -68.0 (c = 1.0, $CHCl_3$). IR (ATR): ν 1683 (C=O), 1547 ($NO_2$). $^1$H-NMR ($CDCl_3$) δ 1.23 (d, 3H, *J* = 7.2, $CH_3$), 2.78-2.87 (m, 2H, $2H_3$), 3.10 (dd, 1H, *J* = 7.2, 3.8, $H_1$), 3.18-3.30 (m, 1H, $H_6$), 3.77 (s, 3H, $OCH_3$), 4.93 (td, 1H, *J* = 5.5, 3.9, $H_2$), 6.63 (t, 1H, *J* = 2.1, $H_{2'}$), 6.65-6.70 (m, 1H, $H_{4'}$), 6.84 (ddd, 1H, *J* = 8.3, 2.6, 0.9, $H_{6'}$), 6.87 (dt, 1H, *J* = 3.2, 1.7, $H_5$), 7.24 (t, 1H, *J* = 7.9, $H_{5'}$), 9.57 (s, 1H, CHO). $^{13}$C-NMR ($CDCl_3$): δ 19.8 ($CH_3$), 24.9 ($C_3$), 34.0 ($C_6$), 49.6 ($C_1$), 55.4 ($OCH_3$), 83.8 ($C_2$), 113.2 ($C_{6'}$), 114.1 ($C_{2'}$), 120.0 ($C_{4'}$), 130.2 ($C_{5'}$), 136.0 ($C_4$), 139.2 ($C_{1'}$), 154.1 ($C_5$), 160.1 ($C_{3'}$), 192.6 (CHO). HPLC (method A, $t_R$, min): 14.54. MS (ESI, *m/z*, %): 274.0 ([M-H]$^-$, 100).

**(1*R*,2*R*,6*R*)-2'-Methoxy-6-methyl-2-nitro-1,2,3,6-tetrahydro[1,1'-biphenyl]-4-carbaldehyde, 56.** Following general procedure A using 1-methoxy-2-[(*E*)-2-nitroethenyl]benzene (250 mg, 1.40 mmol), compound **56** was obtained as a colorless oil (205 mg, 53%). Chromatography: hexane to hexane/EtOAc 8:2.

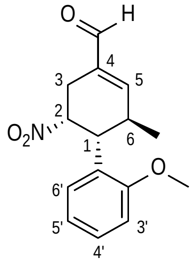


$R_f$: 0.40 (hexane/EtOAc 7:3). $[\alpha]^D_{20}$ = -175.0 (c = 1.0, $CHCl_3$). IR (ATR): ν 1682 (C=O), 1544 ($NO_2$), 1243 (COC). $^1$H-NMR ($CDCl_3$) δ 1.15 (d, *J* = 7.1, 3H, $CH_3$), 2.68-2.80 (m, 1 H, $H_3$), 2.91-3.02 (m, 1H, $H_3$), 3.22-3.36 (m, 1H, $H_6$), 3.48 (dd, *J* = 10.2, 3.4, 1H, $H_1$), 3.84 (s, 3H, $OCH_3$), 5.08 (dt, *J* = 5.8, 2.8, 1H, $H_2$), 6.86-6.98 (m, 3H, $H_5$, $H_{3'}$, $H_{4'}$), 7.07 (dd, *J* = 7.6, 1.5, 1H, $H_{6'}$) 7.26-7.32 (m, 1H, $H_{5'}$), 9.56 (s, 1H, CHO). $^{13}$C-NMR ($CDCl_3$): δ 19.1 ($CH_3$), 26.4 ($C_3$), 31.0 ($C_6$), 41.5 ($C_1$), 55.5 ($OCH_3$), 83.2 ($C_2$), 110.7 ($C_{3'}$), 121.0 ($C_{4'}$), 125.3 ($C_{1'}$), 127.0 ($C_{6'}$), 129.0 ($C_{5'}$), 135.7 ($C_4$), 155.0 ($C_5$), 157.3 ($C_{2'}$), 193.1 (CHO).

**(1*R*,2*R*,6*R*)-6-Methyl-2-nitro-1,2,3,6-tetrahydro[1,1'-biphenyl]-4-carbaldehyde, 57.** Following general procedure A using [(*E*)-2-nitroethenyl]benzene (250 mg, 1.68 mmol), compound **57** was obtained as a colorless oil (214 mg, 52%). Chromatography: hexane to hexane/EtOAc 8:2. The spectroscopic data were consistent with those previously reported for its enantiomer (Enders, D. *et al. Adv. Synth. Catal.* **2008**, *350*, 267).

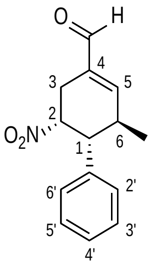


$R_f$: 0.50 (hexane/EtOAc 7:3). $[\alpha]^D_{20}$ = -72.0 (c = 1.0, $CHCl_3$). IR (ATR): ν 1683 (C=O), 1548 ($NO_2$). $^1$H-NMR ($CDCl_3$) δ 1.23 (d, *J* = 7.3, 3H, $CH_3$), 2.79-2.86 (m, 2H, $2H_3$), 3.15 (dd, *J* = 7.2, 3.7, 1H, $H_1$), 3.18-3.30 (m, 1H, $H_6$), 4.94 (td, *J* = 4.9, 3.9, 1H, $H_2$), 6.92 (dt, *J* = 3.2, 1.7, 1H, $H_5$), 7.07-7.12 (m, 2H, $H_{2'}$, $H_{6'}$), 7.28-7.35 (m, 3H, $H_{3'}$, $H_{4'}$, $H_{5'}$), 9.58 (s, 1H, CHO). $^{13}$C-NMR ($CDCl_3$): δ 19.8 ($CH_3$), 24.8 ($C_3$), 34.0 ($C_6$), 49.6 ($C_1$), 83.9 ($C_2$), 127.8 ($C_{2'}$, $C_{6'}$), 128.4 ($C_{4'}$), 129.2 ($C_{3'}$, $C_{5'}$), 136.0 ($C_4$), 137.6 ($C_{1'}$), 154.1 ($C_5$), 192.6 (CHO).

**(1*R*,2*R*,6*R*)-4',6-Dimethyl-2-nitro-1,2,3,6-tetrahydro[1,1'-biphenyl]-4-carbaldehyde, 58.** Following general procedure A using 1-methyl-4-[(*E*)-2-nitroethenyl]benzene (350 mg, 2.15 mmol), compound **58** was obtained as a yellow oil (198 mg, 31%). Chromatography: hexane to hexane/EtOAc 6:4.

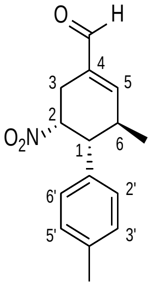


$R_f$: 0.73 (hexane/EtOAc 7:3). $[\alpha]^D_{20}$ = -72.0 (c = 1.0, $CHCl_3$). IR (ATR): ν 1682 (C=O), 1450 ($NO_2$). $^1$H-RMN ($CDCl_3$): δ 1.20 (d, *J* = 6.1, 3H, $CHCH_3$), 2.32 (s, 3H, $C_{Ar}CH_3$), 2.80-2.82 (m, 2H, $2H_3$), 3.11 (dd, *J* = 7.1, 3.8, 1H, $H_1$), 3.16-3.25 (m, 1H, $H_6$) 4.91 (td, *J* = 5.5, 3.8, 1H, $H_2$), 6.90-6.92 (m, 1H, $H_5$), 6.97 (d, *J* = 8.1, 2H, $H_{2'}$, $H_{6'}$), 7.12 (d, *J* = 8.1, 2H, $H_{3'}$, $H_{5'}$), 9.57 (s, 1H, CHO). $^{13}$C-RMN ($CDCl_3$): δ 19.8 ($CHCH_3$), 21.2 ($C_{Ar}CH_3$), 24.8 ($C_3$), 34.1 ($C_6$), 49.3 ($C_1$), 83.9 ($C_2$), 127.8 ($C_{2'}$, $C_{6'}$), 129.9 ($C_{3'}$, $C_{5'}$), 134.5 ($C_{1'}$), 136.0 ($C_4$), 138.1 ($C_{4'}$), 154.2 ($C_5$), 192.7 (CHO). HPLC (method A, $t_R$, min): 11.53.

**(1*R*,2*R*,6*R*)-4'-Chloro-6-methyl-2-nitro-1,2,3,6-tetrahydro[1,1'-biphenyl]-4-carbaldehyde, 59.** Following general procedure A using 1-chloro-4-[(*E*)-2-

nitroethenyl]benzene (400 mg, 2.18 mmol), compound **59** was obtained as a yellow oil (419 mg, 75%). Chromatography: hexane to hexane/EtOAc 6:4.

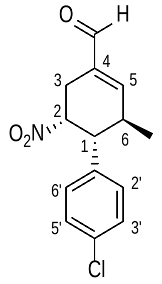


$R_f$: 0.43 (hexane/EtOAc 7:3). $[\alpha]^D_{20}$ = -20.0 (c = 1.0, $CHCl_3$). IR (ATR): ν 1684 (C=O), 1492 ($NO_2$). $^1$H-NMR ($CDCl_3$): δ 1.22 (d, *J* = 7.0, 3H, $CH_3$), 2.80-2.83 (m, 2H, $2H_3$), 3.13 (dd, *J* = 7.0, 3.5, 1H, $H_1$), 3.16-3.21 (m, 1H, $H_6$), 4.88-4.93 (m, 1H, $H_2$), 6.88-6.90 (m, 1H, $H_5$), 7.03 (d, *J* = 8.4, 2H, $H_{3'}$, $H_{5'}$), 7.14 (d, *J* = 8.4, 2H, $H_{2'}$, $H_{6'}$), 9.57 (s, 1H, CHO). $^{13}$C-NMR ($CDCl_3$): δ 19.6 ($CH_3$), 24.6 ($C_3$), 33.8 ($C_6$), 48.9 ($C_1$), 83.5 ($C_2$), 129.0 ($C_{3'}$, $C_{5'}$), 129.3 ($C_{2'}$, $C_{6'}$), 134.1 ($C_{4'}$), 136.0 ($C_4$), 136.2 ($C_{1'}$), 153.4 ($C_5$), 192.4 (CHO).

**(1*R*,2*R*,6*R*)-3'-Chloro-6-methyl-2-nitro-1,2,3,6-tetrahydro[1,1'-biphenyl]-4-carbaldehyde, 60.** Following general procedure A using 1-chloro-3-[(*E*)-2-nitroethenyl]benzene (350 mg, 1.91 mmol), compound **60** was obtained as a yellow oil (311 mg, 58%). Chromatography: hexane to hexane/EtOAc 6:4.

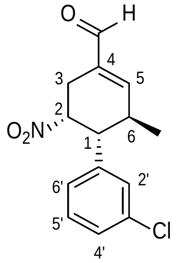


$R_f$: 0.40 (hexane/EtOAc 7:3). $[\alpha]^D_{20}$ = -68.0 (c = 1.0, $CHCl_3$). IR (ATR): ν 1683 (C=O), 1548 ($NO_2$). $^1$H-NMR ($CDCl_3$): δ 1.38 (d, *J* = 7.1, 3H, $CH_3$), 2.95-3.04 (m, 2H, $2H_3$), 3.27 (dd, *J* = 7.4, 3.7, 1H, $H_1$), 3.29-3.43 (m, 1H, $H_6$), 5.09 (td, *J* = 5.4, 3.7, 1H, $H_2$), 7.03-7.07 (m, 1H, $H_5$), 7.14 (td, *J* = 6.4, 1.8, 1H, $H_{6'}$), 7.25-7.27 (m, 1H, $H_{4'}$), 7.40-7.45 (m, 2H, $H_{2'}$, $H_{5'}$), 9.74 (s, 1H, CHO). $^{13}$C-NMR ($CDCl_3$): δ 19.7 ($CH_3$), 24.8 ($C_3$), 33.8 ($C_6$), 49.2 ($C_1$), 83.6 ($C_2$), 125.9 ($C_{6'}$), 128.1 ($C_{4'}$), 128.6 ($C_{2'}$), 130.5 ($C_{5'}$), 135.0 ($C_{3'}$), 135.9 ($C_4$), 139.7 ($C_{1'}$), 153.5 ($C_5$), 192.5 (CHO). HPLC (method B, $t_R$, min): 15.32. MS (ESI, *m/z*, %): 278.1 ($[M-H]^+$, 100).

**(1*R*,2*R*,6*R*)-2'-Chloro-6-methyl-2-nitro-1,2,3,6-tetrahydro[1,1'-biphenyl]-4-carbaldehyde, 61.** Following general procedure A using 1-chloro-2-[(*E*)-2-nitroethenyl]benzene (350 mg, 1.91 mmol), compound **61** was obtained as a yellow oil (244 mg, 46%). Chromatography: hexane to hexane/EtOAc 6:4.

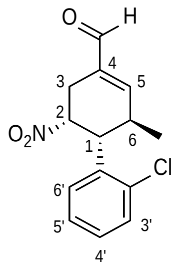


$R_f$: 0.60 (hexane/EtOAc 7:3). $[\alpha]^D_{20}$ = -285.0 (c = 1.0, $CHCl_3$). IR (ATR): ν 1684 (C=O), 1547 ($NO_2$). $^1$H-NMR ($CDCl_3$): δ 1.09 (d, *J* = 7.1, 3H, $CH_3$), 2.69-2.81 (m, 1H, $H_3$), 2.97-3.08 (m, 1H, $H_3$), 3.19-3.33 (m, 1H, $H_6$), 3.48 (dd, *J* = 10.7, 3.3, 1H, $H_1$), 5.05-5.11 (m, 1H, $H_2$), 6.80-6.88 (m, 1H, $H_5$), 7.09-7.14 (m, 1H, $H_{6'}$), 7.21-7.26 (m, 2H, $H_{4'}$, $H_{5'}$), 7.40-7.45 (m, 1H, $H_{3'}$), 9.55 (s, 1H, CHO). $^{13}$C-NMR ($CDCl_3$): δ 18.8 ($CH_3$), 26.7 ($C_3$), 31.0 ($C_6$), 45.2 ($C_1$), 83.1 ($C_2$), 127.7 ($C_{6'}$), 127.8 ($C_{5'}$), 129.3 ($C_{4'}$), 130.3 ($C_{3'}$), 134.7 ($C_{1'}$), 134.8 ($C_{2'}$), 135.6 ($C_4$), 154.0 ($C_5$), 192.8 (CHO). HPLC (method B, $t_R$, min): 15.44. MS (ESI, *m/z*, %): 278.0 ($[M-H]^+$, 100).

**(1*R*,2*R*,6*R*)-3'-Fluoro-6-methyl-2-nitro-1,2,3,6-tetrahydro[1,1'-biphenyl]-4-carbaldehyde, 63.** Following general procedure A using 1-fluoro-3-[(*E*)-2-nitroethenyl]benzene (500 mg, 2.84 mmol), compound **63** was obtained as a yellow oil (424 mg, 57%). Chromatography: hexane to hexane/EtOAc 7:3.

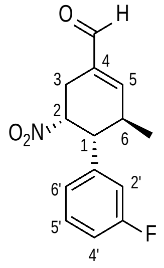


$R_f$: 0.50 (hexane/EtOAc 7:3). $[\alpha]^D_{20}$ = -100.0 (c = 1.0, $CHCl_3$). IR (ATR): ν 1683 (C=O), 1547 ($NO_2$). $^1$H-NMR ($CDCl_3$): δ 1.24 (d, *J* = 6.9, 3H, $CH_3$), 2.83 (dt, *J* = 5.5, 1.9, 2H, $2H_3$), 3.11-3.28 (m, 2H, $H_1$, $H_6$), 4.93 (td, *J* = 5.5, 3.6, 1H, $H_2$), 6.82 (dt, *J* = 9.9, 2.2, 1H, $H_{2'}$), 6.84-6.94 (m, 2H, $H_5$, $H_{6'}$), 7.00 (tdd, *J* = 8.4, 2.6, 1.0, 1H, $H_{4'}$), 7.25-7.36 (m, 1H, $H_{5'}$), 9.58 (s, 1H, CHO). $^{13}$C-NMR ($CDCl_3$): δ 19.8 ($CH_3$), 24.7 ($C_3$), 34.0 ($C_6$), 49.2 (d, *J* = 1.7, $C_1$), 83.6 ($C_2$), 114.5 (d, *J* = 22.0, $C_{2'}$), 115.4 (d, *J* = 21.0, $C_{4'}$), 123.6 (d, *J* = 3.1,

$C_{6'}$), 130.8 (d, *J* = 8.3, $C_{5'}$), 135.9 ($C_4$), 140.1 (d, *J* = 7.2, $C_{1'}$) 153.5 ($C_5$), 167.2 (d, *J* = 247.1, $C_{3'}$), 192.5 (CHO). HPLC (method B, $t_R$, min): 10.49. MS (ESI, *m/z*, %): 262.1 ($[M-H]^+$, 100).

**(1*R*,2*R*,6*R*)-2'-Fluoro-6-methyl-2-nitro-1,2,3,6-tetrahydro[1,1'-biphenyl]-4-carbaldehyde, 64.** Following general procedure A using 1-fluoro-2-[(*E*)-2-nitroethenyl]benzene (500 mg, 2.84 mmol), compound **64** was obtained as a colorless oil (253 mg, 34%). Chromatography: hexane to hexane/EtOAc 7:3.

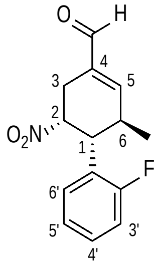


$R_f$: 0.40 (hexane/EtOAc 7:3). $[\alpha]^D_{20}$ = -156.0 (c = 1.0, $CHCl_3$). IR (ATR): ν 1685 (C=O), 1548 ($NO_2$). $^1$H-NMR ($CDCl_3$): δ 11.28 (d, *J* = 6.9, 3H, $CH_3$), 2.81-2.95 (m, 1 H, $H_3$), 3.05-3.19 (m, 1H, $H_3$), 3.35-3.45 (m, 1H, $H_6$), 3.49 (dd, *J* = 9.9, 3.3, 1H, $H_1$), 5.16 (dt, *J* = 5.9, 2.9, 1H, $H_2$), 6.99 (td, *J* = 2.3, 1.3, $H_5$), 7.20-7.27 (m, 2H, $H_{3'}$, $H_{6'}$), 7.33-7.46 (m, 2H, $H_{4'}$, $H_{5'}$), 9.68 (s, 1H, CHO). $^{13}$C-NMR ($CDCl_3$): δ 19.1 ($CH_3$), 26.2 ($C_3$), 31.3 ($C_6$), 41.4 (d, *J* = 2.9, $C_1$), 83.4 ($C_2$), 115.9 (d, *J* = 22.6, $C_{3'}$), 124.5 (d, *J* = 13.8, $C_{1'}$), 124.9 (d, *J* = 3.6, $C_{6'}$), 127.8 (d, *J* = 3.4, $C_{5'}$), 129.8 (d, *J* = 8.7, $C_{4'}$), 135.6 ($C_4$), 153.9 ($C_5$), 161.1 (d, *J* = 245.5, $C_{2'}$), 192.8 (CHO). HPLC (method B, $t_R$, min): 10.47. MS (ESI, *m/z*, %): 262.1 ($[M-H]^+$, 100).

**(1*R*,2*R*,6*R*)-6-Methyl-2-nitro-4'-(trifluoromethyl)-1,2,3,6-tetrahydro[1,1'-biphenyl]-4-carbaldehyde, 65.** Following general procedure A using (1-[(*E*)-2-nitroethenyl]-4-(trifluoromethyl)benzene (361 mg, 1.66 mmol), compound **65** was obtained as a yellow oil (100 mg, 19%). Chromatography: hexane to hexane/EtOAc 1:1.

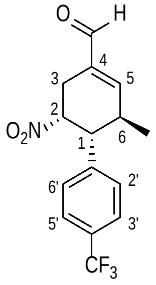

$R_f$: 0.28 (hexane/EtOAc 7:3). $[\alpha]^D{}_{20}$ = -82.0 (c = 1.0, $CHCl_3$). IR (ATR): ν 1685 (C=O), 1549 ($NO_2$). $^1$H-RMN ($CDCl_3$): δ 1.23 (d, *J* = 6.8, 3H, $CH_3$), 2.85 (d, *J* = 5.5, 2H, 2$H_3$), 3.20-3.28 (m, 2H, $H_1$, $H_6$), 4.95 (td, *J* = 5.4, 3.3, 1H, $H_2$), 6.91-6.96 (m, 1H, $H_5$), 7.23 (d, *J* = 8.1, 2H, $H_{2'}$, $H_{6'}$), 7.60 (d, *J* = 8.1, 2H, $H_{3'}$, $H_{5'}$), 9.59 (s, 1H, CHO). $^{13}$C-RMN ($CDCl_3$): δ 19.8 ($CH_3$), 24.8 ($C_3$), 33.8 ($C_6$), 49.4 ($C_1$), 83.5 ($C_2$), 121.8 (q, *J* = 273.3, $CF_3$), 126.2 (q, *J* = 3.7, $C_{3'}$, $C_{5'}$), 128.3 ($C_{2'}$, $C_{6'}$), 130.6 (q, *J* = 32.8, $C_{4'}$), 136.0 ($C_4$), 141.7 ($C_{1'}$), 155.3 ($C_5$), 192.4 (CHO).

**(1*R*,2*R*,6*R*)-6-Methyl-2-nitro-4'-(trifluoromethoxy)-1,2,3,6-tetrahydro[1,1'-biphenyl]-4-carbaldehyde, 66.** Following general procedure A using (1-[(*E*)-2-nitroethenyl]-4-(trifluoromethoxy)benzene (337 mg, 1.45 mmol), compound **66** was obtained as a yellow oil (180 mg, 38%). Chromatography: hexane to hexane/EtOAc 1:1.

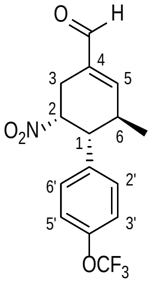

$R_f$: 0.27 (hexane/EtOAc 7:3). $[\alpha]^D{}_{20}$ = -86.0 (c = 1.0, $CHCl_3$). IR (ATR): ν 1684 (C=O), 1509 ($NO_2$). $^1$H-RMN ($CDCl_3$): δ 1.24 (d, *J* = 6.6, 3H, $CH_3$), 2.83 (d, *J* = 5.4, 2H, 2$H_3$), 3.16-3.22 (m, 2H, $H_1$, $H_6$), 4.92 (td, *J* = 5.5, 3.1, 1H, $H_2$), 6.91 (d, *J* = 2.3, 1H, $H_5$), 7.13 (d, *J* = 8.8, 2H, $H_{3'}$, $H_{5'}$), 7.18 (d, *J* = 9.1, 2H, $H_{2'}$, $H_{6'}$), 9.58 (s, 1H, CHO). $^{13}$C-RMN ($CDCl_3$): δ 19.8 ($CH_3$), 24.7 ($C_3$), 34.1 ($C_6$), 48.9 ($C_1$), 83.5 ($C_2$), 120.5 (q, *J* = 257.5, $CF_3$), 121.5 ($C_{3'}$, $C_{5'}$), 129.3 ($C_{2'}$, $C_{6'}$), 136.0 ($C_{1'}$), 136.2 ($C_4$), 149.1 (q, *J* = 2.0, $C_{4'}$), 153.4 ($C_5$), 192.5 (CHO).

**(3*R*,4*R*,5*R*)-3-Methyl-5-nitro-4-(pyridin-3-yl)cyclohex-1-ene-1-carbaldehyde, 67.** Following general procedure A using 3-[(*E*)-2-nitroethenyl]pyridine (296 mg, 1.97 mmol) at 40 ºC, compound **67** was obtained as a yellow oil (198 mg, 41%). Chromatography: hexane to hexane/EtOAc 1:9.

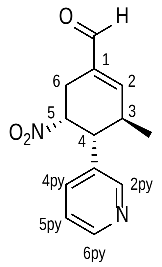


$R_f$: 0.88 (DCM/MeOH/$NH_3$ 8:2:0.1). $[\alpha]^D_{20}$ = 7.0 (c = 1.0, $CHCl_3$). IR (ATR): ν 1710 (C=O), 1549 ($NO_2$). $^1$H-RMN ($CDCl_3$): δ 1.09 (d, *J* = 7.0, 3H, $CH_3$), 2.76-2.86 (m, 2H, $2H_6$), 3.17-3.25 (m, 2H, $H_3$, $H_4$), 4.92-4.96 (m, 1H, $H_5$), 6.73 (s, 1H, $H_2$), 7.26-7.33 (m, 1H, $H_{5py}$), 7.55-7.59 (m, 1H, $H_{4py}$), 8.50 (d, *J* = 1.8, 1H, $H_{2py}$), 8.55 (dd, *J* = 4.8, 1.5, 1H, $H_{6py}$), 9.55 (s, 1H, CHO). $^{13}$C-RMN ($CDCl_3$): δ 18.3 ($CH_3$), 24.7 ($C_6$), 33.7 ($C_3$), 47.1 ($C_4$), 86.6 ($C_5$), 124.1 ($C_{5py}$), 133.4 ($C_1$), 135.4 ($C_{4py}$), 136.4 ($C_{3py}$), 149.6 ($C_{6py}$), 149.7 ($C_{2py}$), 152.3 ($C_2$), 191.8 (CHO). HPLC (method A, $t_R$, min): 13.13. MS (ESI, *m/z*, %): 247.1 ($[M+H]^+$, 100).

**((3*R*,4*S*,5*R*)-3-Methyl-5-nitro-4-(thiophen-2-yl)cyclohex-1-ene-1-carbaldehyde, 68.** Following general procedure A using 2-[(*E*)-2-nitroethenyl]thiophene (470 mg, 3.03 mmol), compound **68** was obtained as a yellow oil (260 mg, 34%). Chromatography: hexane to hexane/EtOAc 1:1.

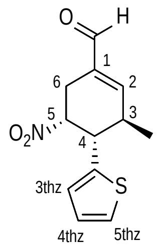


$R_f$: 0.34 (hexane/EtOAc 7:3). $[\alpha]^D_{20}$ = -38.0 (c = 1.0, $CHCl_3$). IR (ATR): ν 1684 (C=O), 1545 ($NO_2$). $^1$H-RMN ($CDCl_3$): δ 1.32 (d, *J* = 7.3, 3H, $CH_3$), 2.87 (dt, *J* = 6.3, 1.8, 2H, $2H_6$), 3.18-3.36 (m, 1H, $H_3$), 3.64 (dd, *J* = 5.6, 3.8, 1H, $H_4$), 4.91 (td, *J* = 6.3, 3.7, 1H, $H_5$), 6.81-6.83 (m, 1H, $H_{3thz}$), 6.85-6.88 (m, 1H, $H_2$), 6.95 (dd, *J* = 5.1, 3.5, 1H, $H_{4thz}$), 7.21 (dt, *J* = 5.1, 1.2, 1H, $H_{5thz}$), 9.57 (s, 1H, CHO). $^{13}$C-RMN ($CDCl_3$): δ 20.1 ($CH_3$), 24.1 ($C_6$), 36.9 ($C_3$), 44.9 ($C_4$), 82.9 ($C_5$), 125.3 ($C_{5thz}$), 126.3 ($C_{3thz}$), 127.2 ($C_{4thz}$), 136.0 ($C_1$), 139.6 ($C_{2thz}$), 152.8 ($C_2$), 192.4 (CHO). HPLC (method A, $t_R$, min): 18.13. MS (ESI, *m/z*, %): 252.1 ($[M+H]^+$, 100).

**(3*R*,4*S*,5*R*)-3-Methyl-5-nitro-4-(1,3-oxazol-4-yl)cyclohex-1-ene-1-carbaldehyde, 69.** Following general procedure A using 4-[(*E*)-2-nitroethenyl]-1,3-oxazole (463 mg, 3.36

mmol), compound **69** was obtained as a yellow oil (376 mg, 47%). Chromatography: hexane to hexane/EtOAc 6:4.

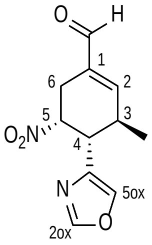


$R_f$: 0.50 (hexane/EtOAc 1:1). $[\alpha]^D_{20}$ = -20.0 (c = 1.0, $CHCl_3$). IR (ATR): ν 1682 (C=O), 1549 ($NO_2$). $^1$H-RMN ($CDCl_3$): δ 1.13 (d, *J* = 6.8, 3H, $CH_3$), 2.66-2.80 (m, 1H, $H_6$), 3.00-3.12 (m, 2H, $H_3$, $H_4$), 3.17 (dd, *J* = 17.2, 5.2, 1H, $H_6$), 4.96-5.10 (m, 1H, $H_5$), 6.72 (s, 1H, $H_2$), 7.56 (d, *J* = 0.9, 1H, $H_{5ox}$), 7.87 (s, 1H, $H_{2ox}$), 9.53 (s, 1H, CHO). $^{13}$C-RMN ($CDCl_3$): δ 18.4 ($CH_3$), 27.3 ($C_6$), 35.5 ($C_3$), 42.9 ($C_4$), 85.5 ($C_5$), 136.1 ($C_1$), 136.4 ($C_{4ox}$), 137.5 ($C_{5ox}$), 151.9 ($C_{2ox}$), 152.7 ($C_2$), 192.0 (CHO). HPLC (method A, $t_R$, min): 13.04. MS (ESI, *m/z*, %): 237.1 ($[M+H]^+$, 100).

**1-Cyclopropyl-*N*-{[(1*R*,2*S*,6*R*)-3'-methoxy-6-methyl-2-nitro-1,2,3,6-tetrahydro [1,1'-biphenyl]-4-yl]methyl}methanamine, 70.** Following general procedure C using **55** (133 mg, 0.48 mmol) and (cyclopropylmethyl)amine (83 µL, 0.97 mmol), compound **77** was obtained as a yellow oil (120 mg, 75%).

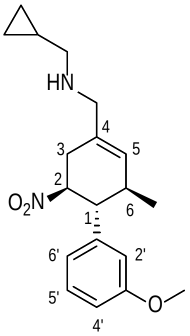


$R_f$: 0.10 (DCM/MeOH/$NH_3$ 9.5:0.5:0.1). $[\alpha]^D_{20}$ = -18.0 (c = 1.0, $CHCl_3$). IR (ATR): ν 1547 ($NO_2$). $^1$H-NMR ($CDCl_3$): δ 0.10-0.17 (m, 2H, $CH_{2cpr}$), 0.44-0.55 (m, 2H, $CH_{2cpr}$), 0.91 (d, *J* = 7.0, 3H, $CH_3$), 0.94-1.01 (m, 1H, $CH_{cpr}$), 1.96 (s, 1H, NH), 2.47 (d, *J* = 6.8, 2H, NHC$\underline{H}_2$CH) 2.49-2.59 (m, 1H, $H_6$), 2.73-2.80 (m, 2H, $2H_3$), 2.85 (t, *J* = 10.6, 1H, $H_1$), 3.25 (s, 2H, NHC$\underline{H}_2$), 3.78 (s, 3H, $OCH_3$), 4.93-5.06 (m, 1H, $H_2$), 5.56 (s, 1H, $H_5$), 6.72 (t, *J* = 2.0, 1H, $H_{2'}$), 6.77 (d, *J* = 8.0, 1H, $H_{4'}$), 6.78 (d, *J* = 8.0, 1H, $H_{6'}$), 7.21 (t, *J* = 7.9, 1H, $H_{5'}$). $^{13}$C-NMR ($CDCl_3$): δ 3.6 ($2CH_{2cpr}$), 11.2 ($CH_{cpr}$), 19.7 ($CH_3$), 33.4 ($C_3$), 37.4 ($C_6$), 52.5 ($C_1$), 54.5 (NH$\underline{C}H_2$CH), 54.6 ($NHCH_2$), 55.3 ($OCH_3$), 88.4 ($C_2$), 112.7

($C_{4'}$), 114.4 ($C_{2'}$) 120.3 ($C_{6'}$), 128.5 ($C_5$), 129.8 ($C_{5'}$), 131.7 ($C_4$), 140.4 ($C_{1'}$), 159.8 ($C_{3'}$). HPLC (method A, $t_R$, min): 13.60. MS (ESI, *m/z*, %): 331.2 ($[M+H]^+$, 100).

**1-Cyclopropyl-*N*-{[(1*R*,2*S*,6*R*)-2'-methoxy-6-methyl-2-nitro-1,2,3,6-tetrahydro [1,1'-biphenyl]-4-yl]methyl}methanamine, 71.** Following general procedure C using **56** (193 mg, 0.70 mmol) and (cyclopropylmethyl)amine (0.12 mL, 1.40 mmol), compound **71** was obtained as a yellow oil (188 mg, 81%).

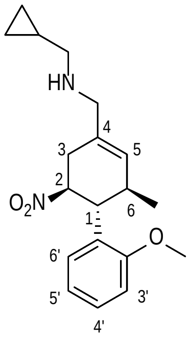


$R_f$: 0.30 (DCM/MeOH/$NH_3$ 9.5:0.5:0.1). $[\alpha]^D_{20}$ = +9.0 (c = 1.0, $CHCl_3$). IR (ATR): ν 1549 ($NO_2$), 1245 (COC). $^1$H-NMR ($CDCl_3$): δ 0.12-0.15 (m, 2H, $CH_{2cpr}$), 0.49-0.52 (m, 2H, $CH_{2cpr}$), 0.90 (d, *J* = 7.0, 3H, $CH_3$), 0.95-1.00 (m, 1H, $CH_{cpr}$), 2.48 (d, *J* = 6.7, 2H, NHC$\underline{H}_2$CH), 2.58-2.66 (m, 1H, $H_3$), 2.66-2.81 (m, 3H, $H_1$, $H_3$, $H_6$), 3.24 (AB system, *J* = 14.3, 2H, NHC$\underline{H}_2$), 3.83 (s, 3H, $OCH_3$), 5.28-5.40 (m, 1H, $H_2$), 5.55 (s, 1H, $H_5$), 6.86-6.90 (m, 2H, $H_{3'}$, $H_{4'}$), 7.09 (d, *J* = 7.4, 1H, $H_{6'}$), 7.17-7.24 (m, 1H, $H_{5'}$). $^{13}$C-NMR ($CDCl_3$): δ 3.5 ($2CH_{2cpr}$), 11.5 ($CH_{cpr}$), 20.0 ($CH_3$), 33.5 ($C_3$), 35.8 ($C_6$), 53.4 ($C_1$), 54.7 (NH$\underline{C}H_2$CH), 55.1($NHCH_2$), 55.8 ($OCH_3$), 86.7 ($C_2$), 111.8 ($C_{3'}$), 121.0 ($C_{4'}$), 128.0 (br, $C_{1'}$) 128.8 ($C_5$, $C_{5'}$), 130.7 (br, $C_{6'}$), 132.4 ($C_4$), 158.2 ($C_{2'}$). HPLC (method A, $t_R$, min): 13.63. MS (ESI, *m/z*, %): 331.2 ($[M+H]^+$, 100).

**1-Cyclopropyl-*N*-{[(1*R*,2*S*,6*R*)-6-methyl-2-nitro-1,2,3,6-tetrahydro[1,1'-biphenyl]-4-yl]methyl}methanamine, 72.** Following general procedure C using **57** (201 mg, 0.82 mmol) and (cyclopropylmethyl)amine (0.14 mL, 1.64 mmol), compound **72** was obtained as a yellow oil (232 mg, 94%).

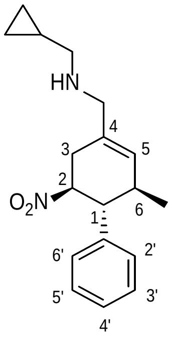


$R_f$: 0.02 (DCM/MeOH/NH$_3$ 9.5:0.5:0.1). $[\alpha]^D_{20}$ = +1.0 (c = 1.0, CHCl$_3$). IR (ATR): ν 1550 (NO$_2$). $^1$H-NMR (CDCl$_3$): δ 0.14-0.30 (m, 2H, CH$_{2cpr}$), 0.54-0.65 (m, 2H, CH$_{2cpr}$), 0.99 (d, *J* = 7.0, 3H, CH$_3$), 1.02-1.10 (m, 1H, CH$_{cpr}$), 2.56 (d, *J* = 6.8, 2H, NHC$\underline{H}_2$CH) 2.58-2.66 (m, 1H, H$_6$), 2.83-2.91 (m, 2H$_3$), 2.97 (t, *J* = 10.4, 1H, H$_1$), 3.35 (s, 2H, NHC$\underline{H}_2$), 5.11 (ddd, *J* = 11.6, 9.3, 6.9, 1H, H$_2$), 5.66 (s, 1H, H$_5$), 7.22-7.44 (m, 5H, H$_{2'-6'}$). $^{13}$C-NMR (CDCl$_3$): δ 3.6 (2CH$_{2cpr}$), 11.2 (CH$_{cpr}$), 19.6 (CH$_3$), 33.3 (C$_3$), 37.4 (C$_6$), 52.1 (C$_1$), 54.4 (NH$\underline{C}$H$_2$CH), 54.6 (NHCH$_2$), 88.5 (C$_2$), 127.7 (C$_{4'}$), 128.1 (C$_{2'}$, C$_{6'}$), 128.5 (C$_5$), 128.8 (C$_{3'}$, C$_{5'}$), 131.7 (C$_4$), 138.7 (C$_{1'}$). HPLC (method A, t$_R$, min): 13.23. MS (ESI, *m/z*, %): 301.2 ([M+H]$^+$, 100).

**1-Cyclopropyl-*N*-{[(1*R*,2*S*,6*R*)-4',6-dimethyl-2-nitro-1,2,3,6-tetrahydro[1,1'-biphenyl]-4-yl} methanamine, 73.** Following general procedure C using **58** (220 mg, 0.85 mmol) and (cyclopropylmethyl)amine (0.15 mL, 1.70 mmol), compound **73** was obtained as a yellow oil (164 mg, 62%). Chromatography: DCM to DCM/EtOH/NH$_3$ 9:1:0.1.

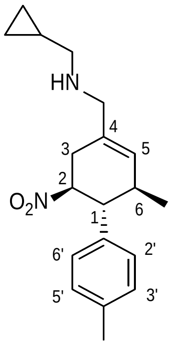


$R_f$: 0.72 (DCM/MeOH/NH$_3$ 9:1:0.1). $[\alpha]^D_{20}$ = -5.0 (c = 1.0, CHCl$_3$). IR (ATR): ν 3300 (NH), 1633 (C=O), 1416 (NO$_2$). $^1$H-NMR (CDCl$_3$): δ 0.11-0.16 (m, 2H, CH$_{2cpr}$), 0.48-0.54 (m, 2H, CH$_{2cpr}$), 0.90 (d, *J* = 7.0, 3H, CHC$\underline{H}_3$), 0.94-1.00 (m, 1H, CH$_{cpr}$), 2.30 (s, 3H, C$_{Ar}$CH$_3$), 2.43-2.57 (m, 3H, H$_6$, NHC$\underline{H}_2$CH), 2.77 (dd, *J* = 6.5, 2.5, 2H, 2H$_3$), 2.84 (dd, *J* = 11.4, 10.6, 1H, H$_1$), 3.26 (s, 2H, NHC$\underline{H}_2$), 4.99 (ddd, *J* = 11.7, 9.6, 6.7, 1H, H$_2$),

5.57 (d, $J$ = 1.9, 1H, $H_5$), 7.09-7.12 (m, 4H, $H_{2'}$, $H_{3'}$, $H_{5'}$, $H_{6'}$). $^{13}$C-NMR ($CDCl_3$): δ 3.6 ($2CH_{2cpr}$), 11.2 ($CH_{cpr}$), 19.7 (CH$\underline{C}H_3$), 21.2 ($C_{Ar}\underline{C}H_3$), 33.4 ($C_3$), 37.5 ($C_6$), 52.2 ($C_1$), 54.5 (NH$\underline{C}H_2$CH), 54.7 ($NHCH_2$), 88.7 ($C_2$), 127.9 ($C_{2'}$, $C_{6'}$), 128.6 ($C_5$), 129.5 ($C_{3'}$, $C_{5'}$), 131.7 ($C_4$), 135.6 ($C_{1'}$), 137.4 ($C_{4'}$). HPLC ($t_R$, min): 15.42. MS (method A, ESI, *m/z*, %): 315.2 ($[M+H]^+$, 100).

**1-Cyclopropyl-*N*-{[(1*R*,2*S*,6*R*)-4'-chloro-6-methyl-2-nitro-1,2,3,6-tetrahydro[1,1'-biphenyl]-4-yl} methanamine, 74.** Following general procedure C using **59** (459 mg, 1.64 mmol) and (cyclopropylmethyl)amine (0.29 mL, 3.28 mmol), compound **74** was obtained as a yellow oil (110 mg, 20%). Chromatography: DCM to DCM/EtOH/$NH_3$ 9:1:0.1.

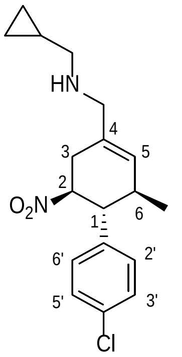


$R_f$: 0.60 (DCM/MeOH/$NH_3$ 9:1:0.1). $[\alpha]^D_{20}$ = 1.0 (c = 1.0, $CHCl_3$). IR (ATR): ν 3300 (NH), 1492 ($NO_2$). $^1$H-RMN ($CDCl_3$): δ 0.07-0.14 (m, 2H, $CH_{2cpr}$), 0.45-0.51 (m, 2H, $CH_{2cpr}$), 0.87 (d, $J$ = 6.9, 3H, $CH_3$), 0.90-0.97 (m, 1H, $CH_{cpr}$), 2.38-2.51 (m, 3H, $H_6$, NHC$\underline{H}_2$CH), 2.75 (d, $J$ = 8.2, 2H, $2H_3$), 2.85 (dd, $J$ = 11.4, 10.7, 1H, $H_1$), 3.22 (s, 2H, NHC$\underline{H}_2$), 4.95 (dt, $J$ = 11.7, 8.2, 1H, $H_2$), 5.53 (d, $J$ = 1.8, 1H, $H_5$), 7.11 (d, $J$ = 8.5, 2H, $H_{2'}$, $H_{6'}$), 7.26 (d, $J$ = 8.5, 2H, $H_{3'}$, $H_{5'}$). $^{13}$C-RMN ($CDCl_3$): δ 3.48 ($CH_{2cpr}$), 3.49 ($CH_{2cpr}$), 11.2 ($CH_{cpr}$), 19.5 ($CH_3$), 33.2 ($C_3$), 37.3 ($C_6$), 51.9 ($C_1$), 54.4 (NH$\underline{C}H_2$CH), 54.6 ($NHCH_2$), 88.3 ($C_2$), 128.0 ($C_5$), 128.9 ($C_{3'}$, $C_{5'}$), 129.4 ($C_{2'}$, $C_{6'}$), 131.9 ($C_4$), 133.4 ($C_{4'}$), 137.3 ($C_{1'}$). HPLC (method A, $t_R$, min): 15.42. MS (ESI, *m/z*, %): 335.1 ($[M+H]^+$, 100).

**1-Cyclopropyl-*N*-{[(1*R*,2*S*,6*R*)-3'-chloro-6-methyl-2-nitro-1,2,3,6-tetrahydro[1,1'-biphenyl]-4-yl} methanamine, 75.** Following general procedure C using **60** (310 mg, 1.11 mmol) and (cyclopropylmethyl)amine (0.19 mL, 2.22 mmol), compound **75** was obtained as a yellow oil (217 mg, 59%). Chromatography: DCM to DCM/EtOH/$NH_3$ 9:1:0.1.

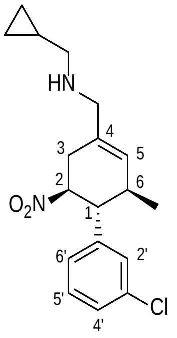

$R_f$: 0.60 (DCM/MeOH/$NH_3$ 9:1:0.1). $[\alpha]^D_{20}$ = -1.0 (c = 1.0, $CHCl_3$). IR (ATR): ν 1549 ($NO_2$). $^1$H-RMN ($CDCl_3$): δ 0.08-0.20 (m, 2H, $CH_{2cpr}$), 0.47-0.56 (m, 2H, $CH_{2cpr}$), 0.88-1.02 (m, 4H, $CH_{cpr}$, $CH_3$), 2.40-2.52 (m, 3H, NHC$\underline{H}_2$CH, $H_6$), 2.76-2.86 (m, 2H, 2$H_3$), 2.89 (t, *J* = 10.7, 1H, $H_1$), 3.20-3.31 (m, 2H, NHC$\underline{H}_2$), 5.00 (dt, *J* = 11.6, 8.1, 1H, $H_2$), 5.57 (s, 1H, $H_5$), 7.05-7.15 (m, 1H, $H_{6'}$), 7.17-7.24 (m, 1H, $H_{2'}$), 7.24-7.27 (m, 2H, $H_{4'}$, $H_{5'}$). $^{13}$C-RMN ($CDCl_3$): δ 3.5 (2$CH_{2cpr}$), 11.3 ($CH_{cpr}$), 19.6 ($CH_3$), 33.2 ($C_3$), 37.4 ($C_6$), 52.2 ($C_1$), 54.6 (NH$\underline{C}H_2$CH), 54.7 (NH$CH_2$), 88.2 ($C_2$), 126.4 ($C_{6'}$), 127.9 ($C_5$), 128.1 ($C_{2'}$), 128.3 ($C_{4'}$), 130.1 ($C_{5'}$), 132.1 ($C_4$), 134.7 ($C_{3'}$), 141.0 ($C_{1'}$). HPLC (method A, $t_R$, min): 13.53. MS (ESI, *m/z*, %): 335.1 ($[M+H]^+$, 100).

**1-Cyclopropyl-*N*-{[(1*R*,2*S*,6*R*)-2'-chloro-6-methyl-2-nitro-1,2,3,6-tetrahydro[1,1'-biphenyl]-4-yl} methanamine, 76.** Following general procedure C using **61** (222 mg, 0.79 mmol) and (cyclopropylmethyl)amine (0.14 mL, 1.58 mmol), compound **76** was obtained as a yellow oil (104 mg, 39%). Chromatography: DCM to DCM/EtOH/$NH_3$ 9:1:0.1.

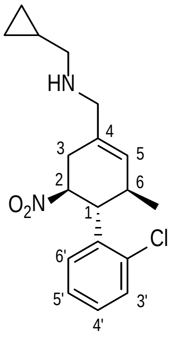

$R_f$: 0.40 (DCM/MeOH/$NH_3$ 9.5:0.5:0.1). $[\alpha]^D_{20}$ = -19.0 (c = 1.0, $CHCl_3$). IR (ATR): ν 1550 ($NO_2$). $^1$H-RMN ($CDCl_3$): δ 0.06-0.17 (m, 2H, $CH_{2cpr}$), 0.44-0.55 (m, 2H, $CH_{2cpr}$), 0.84-1.03 (m, 4H, $CH_{cpr}$, $CH_3$), 2.34-2.52 (m, 3H, NHC$\underline{H}_2$CH, $H_6$), 2.70-2.89 (m, 2H, 2$H_3$), 3.18-3.33 (m, 1H, NHC$\underline{H}_2$), 3.79 (t, *J* = 11.3, 1H, $H_1$), 4.91-5.11 (m, 1H, $H_2$), 5.56 (s, 1H, $H_5$), 7.11-7.25 (m, 3H, $H_{4'}$, $H_{5'}$, $H_{6'}$), 7.36 (d, *J* = 8.0, 1H, $H_{3'}$). $^{13}$C-RMN ($CDCl_3$):

δ 3.4 (2$CH_{2cpr}$), 11.3 ($CH_{cpr}$), 18.9 ($CH_3$), 33.3 ($C_3$), 38.9 ($C_6$), 46.5 ($C_1$), 54.5 (NH$\underline{C}H_2$CH), 54.7 (NH$CH_2$), 87.5 ($C_2$), 126.9 ($C_{5'}$), 127.3 ($C_{4'}$), 128.0 ($C_5$), 128.4 ($C_{6'}$), 129.9 ($C_{3'}$), 132.1 ($C_4$), 136.9 ($C_{2'}$), 140.9 ($C_{1'}$). HPLC (method A, $t_R$, min): 13.62. MS (ESI, *m/z*, %): 335.1 ($[M+H]^+$, 100).

**1-Cyclopropyl-*N*-{[(1*R*,2*S*,6*R*)-3'-fluoro-6-methyl-2-nitro-1,2,3,6-tetrahydro[1,1'-biphenyl]-4-yl} methanamine, 78.** Following general procedure C using **63** (485 mg, 1.84 mmol) and (cyclopropylmethyl)amine (0.32 mL, 3.68 mmol), compound **78** was obtained as a yellow oil (320 mg, 55%). Chromatography: DCM to DCM/EtOH/$NH_3$ 9:1:0.1.

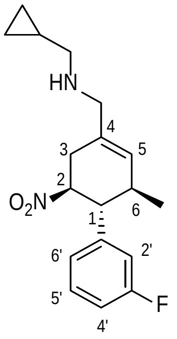


$R_f$: 0.60 (DCM/MeOH/$NH_3$ 9:1:0.1). $[\alpha]^D_{20}$ = -7.0 (c = 1.0, $CHCl_3$). IR (ATR): ν 1550 ($NO_2$). $^1$H-RMN ($CDCl_3$): δ 0.08-0.18 (m, 2H, $CH_{2cpr}$), 0.46-0.56 (m, 2H, $CH_{2cpr}$), 0.91 (d, *J* = 7.0, 3H, $CH_3$), 0.93-1.02 (m, 1H, $CH_{cpr}$), 1.30 (br s, 1H, NH), 2.40-2.58 (m, 3H, $H_6$, NHC$\underline{H}_2$CH), 2.70-2.83 (m, 2H, 2$H_3$), 2.89 (t, *J* = 11.0, 1H, $H_1$), 3.25 (s, 2H, NHC$\underline{H}_2$), 4.98 (ddd, *J* = 11.7, 8.6, 7.8, 1H, $H_2$), 5.56 (s, 1H, $H_5$), 6.80-7.03 (m, 3H, $H_{2'}$, $H_{4'}$, $H_{6'}$), 7.21-7.34 (m, 1H, $H_{5'}$). $^{13}$C-RMN ($CDCl_3$): δ 3.5 (2$CH_{2cpr}$), 11.4 ($CH_{cpr}$), 19.6 ($CH_3$), 33.3 ($C_3$), 37.4 ($C_6$), 52.3 (d, *J* = 1.8 Hz, $C_1$), 54.6 (NH$\underline{C}H_2$CH), 54.8 (NH$CH_2$), 88.3 ($C_2$), 114.8 (d, *J* = 21.1, $C_{2'}$/$C_{4'}$), 115.1 (d, *J* = 21.5, $C_{2'}$/$C_{4'}$), 123.9 (d, J = 2.6, $C_{6'}$), 127.9 ($C_5$), 130.4 (d, *J* = 8.3, $C_{5'}$), 132.2 ($C_4$), 141.4 (d, *J* = 6.9, $C_{1'}$), 163.0 (d, *J* = 246.3, $C_{3'}$). HPLC (method A, $t_R$, min): 13.68. MS (ESI, *m/z*, %): 319.2 ($[M+H]^+$, 100).

**1-Cyclopropyl-*N*-{[(1*R*,2*S*,6*R*)-2'-fluoro-6-methyl-2-nitro-1,2,3,6-tetrahydro[1,1'-biphenyl]-4-yl} methanamine, 79.** Following general procedure C using **64** (253 mg, 0.96 mmol) and (cyclopropylmethyl)amine (0.17 mL, 1.92 mmol), compound **79** was obtained as a yellow oil (124 mg, 59%). Chromatography: DCM to DCM/EtOH/$NH_3$ 9:1:0.1.

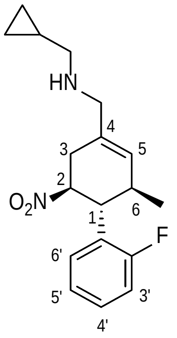


$R_f$: 0.60 (DCM/MeOH/$NH_3$ 9:1:0.1). $[\alpha]^D_{20}$ = -2.0 (c = 1.0, $CHCl_3$). IR (ATR): ν 1550 ($NO_2$). $^1$H-RMN ($CDCl_3$): δ 0.06-0.16 (m, 2H, $CH_{2cpr}$), 0.44-0.54 (m, 2H, $CH_{2cpr}$), 0.93 (d, *J* = 7.0, 3H, $CH_3$), 0.92-1.02 (m, 1H, $CH_{cpr}$), 1.33 (br s, 1H, NH), 2.46 (d, *J* = 6.8, 2H, NHC$\underline{H}_2$CH), 2.52-2.69 (m, 1H, $H_6$), 2.71-2.84 (m, 2H, $2H_3$), 3.13-3-32 (m, 3H, $H_1$, NHC$\underline{H}_2$), 5.19 (dt, *J* = 11.7, 8.1, 1H, $H_2$), 5.55 (s, 1H, $H_5$), 6.98-7.11 (m, 2H, $H_{3'}$, $H_{5'}$), 7.14-7.25 (m, 2H, $H_{4'}$, $H_{6'}$). $^{13}$C-RMN ($CDCl_3$): δ 3.51 ($CH_{2cpr}$), 3.52 ($CH_{2cpr}$), 11.4 ($CH_{cpr}$), 19.7 ($CH_3$), 33.3 ($C_3$), 36.4 ($C_6$), 47.4 (br, $C_1$), 54.6 (NH$\underline{C}$$H_2$CH), 54.8 (NH$CH_2$), 86.7 (d, *J* = 2.9, $C_2$), 116.1 (d, *J* = 22.6, $C_{3'}$) 124.5 (d, *J* = 3.6 , $C_{5'}$), 125.7 (d, *J* = 13.5, $C_{1'}$), 128.0 ($C_5$), 129.3 (d, *J* = 8.6, $C_{4'}$), 130.0 (br, $C_{6'}$), 132.1 ($C_4$), 161.3 (d, *J* = 246.4, $C_{2'}$). HPLC (method A, $t_R$, min): 13.41. MS (ESI, *m/z*, %): 319.2 ($[M+H]^+$, 100).

**1-Cyclopropyl-*N*-{[(1*R*,2*S*,6*R*)-6-methyl-2-nitro-4'-(trifluoromethyl)-1,2,3,6-tetrahydro[1,1'-biphenyl]-4-yl]methyl}methanamine, 80.** Following general procedure C using **65** (130 mg, 0.42 mmol) and (cyclopropylmethyl)amine (72 µL, 10.84 mmol), compound **80** was obtained as a yellow oil (58 mg, 38%). Chromatography: DCM to DCM/EtOH/$NH_3$ 9:1:0.1.

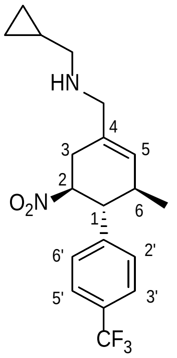


$R_f$: 0.60 (DCM/MeOH/$NH_3$ 9:1:0.1). $[\alpha]^D_{20}$ = -4.0 (c = 1.0, $CHCl_3$). IR (ATR): ν 3300 (NH), 1551 ($NO_2$). $^1$H-RMN ($CDCl_3$): δ 0.12-0.17 (m, 2H, $CH_{2cpr}$), 0.49-0.55 (m, 2H, $CH_{2cpr}$), 0.90 (d, *J* = 7.0, 3H, $CH_3$), 0.96-1.00 (m, 1H, $CH_{cpr}$), 2.44-2.58 (m, 3H, $H_6$, NHC$\underline{H}_2$CH), 2.81 (d, *J* = 8.0, 2H, $2H_3$), 2.97 (t, *J* = 10.8, 1H, $H_1$), 3.28 (s, 2H, NHC$\underline{H}_2$),

5.03 (ddd, $J$ = 11.7, 8.8, 7.5, 1H, $H_2$), 5.8 (s, 1H, $H_5$), 7.32 (d, $J$ = 8.1, 2H, $H_{2'}$, $H_{6'}$), 7.57 (d, $J$ = 8.1, 2H, $H_{3'}$, $H_{5'}$).$^{13}$C-NMR ($CDCl_3$): δ 3.6 ($2CH_{2cpr}$), 11.1 ($CH_{cpr}$), 19.6 ($CH_3$), 33.3 ($C_3$), 37.5 ($C_6$), 52.3 ($C_1$), 54.5 (NH<u>C</u>$H_2$CH), 54.7 ($NHCH_2$), 88.1 ($C_2$), 122.3 (q, $J$ = 275.3, $CF_3$), 125.9 (q, $J$ = 3.7, $C_{3'}$, $C_{5'}$), 128.3 ($C_5$), 128.6 ($C_{2'}$, $C_{6'}$), 130.1 (q, $J$ = 32.6, $C_{4'}$), 131.9 ($C_4$), 143.0 ($C_{1'}$). HPLC (method A, $t_R$, min): 14.55. MS (ESI, *m/z*, %): 368.8 ($[M+H]^+$, 100).

**1-Cyclopropyl-*N*-{[(1*R*,2*S*,6*R*)-6-methyl-2-nitro-4'-(trifluoromethoxy)-1,2,3,6-tetrahydro[1,1'-biphenyl]-4-yl]methyl}methanamine, 81.** Following general procedure C using **66** (166 mg, 0.50 mmol) and (cyclopropylmethyl)amine (87 µL, 1.01 mmol), compound **81** was obtained as a yellow oil (140 mg, 72%). Chromatography: DCM to DCM/EtOH/$NH_3$ 9:1:0.1.

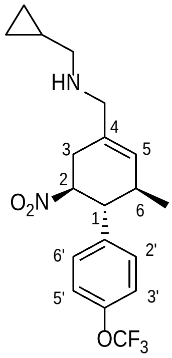


$R_f$: 0.88 (DCM/MeOH/$NH_3$ 9:1:0.1). $[\alpha]^D_{20}$ = 8.0 (c = 1.0, $CHCl_3$). IR (ATR): ν 3300 (NH), 1549 ($NO_2$). $^1$H-RMN ($CDCl_3$): δ 0.09-0.16 (m, 2H, $CH_{2cpr}$), 0.46-0.53 (m, 2H, $CH_{2cpr}$), 0.89 (d, $J$ = 7.0, 3H, $CH_3$), 0.94-0.99 (m, 1H, $CH_{cpr}$), 2.40-2.55 (m, 3H, $H_6$, NHC<u>H</u>$_2$CH), 2.78 (d, $J$ = 8.2, 2H, $2H_3$), 2.91 (dd, $J$ = 11.7, 10.4, 1H, $H_1$), 3.25 (s, 2H, NHC<u>H</u>$_2$), 4.98 (dt, $J$ = 11.7, 8.2, 1H, $H_2$), 5.55 (s, 1H, $H_5$), 7.15 (d, $J$ = 8.8, 2H, $H_{3'}$,$H_{5'}$), 7.22 (d, $J$ = 8.8, 2H, $H_{2'}$, $H_{6'}$). $^{13}$C-NMR ($CDCl_3$): δ 3.5 ($CH_{2cpr}$), 3.9 ($CH_{2cpr}$), 11.3 ($CH_{cpr}$), 19.6 ($CH_3$), 33.3 ($C_3$), 37.5 ($C_6$), 51.9 ($C_1$), 54.6 (NH<u>C</u>$H_2$CH), 54.7 ($NHCH_2$), 88.4 ($C_2$), 120.5 (q, $J$ = 257.1, $OCF_3$), 121.2 ($C_{3'}$, $C_{5'}$), 127.9 ($C_5$), 129.5 ($C_{2'}$, $C_{6'}$), 133.2 ($C_4$), 137.5 ($C_{1'}$), 148.7 (q, $J$ = 1.8, $C_{4'}$). HPLC (method A, $t_R$, min): 14.74. MS (ESI, *m/z*, %): 384.8 ($[M+H]^+$, 100).

**1-Cyclopropyl-*N*-{[(3*R*,4*R*,5*S*)-3-methyl-5-nitro-4-(pyridin-3-yl)cyclohex-1-en-1-yl]methyl}methanamine, 82.** Following general procedure C using **67** (220 mg, 0.85 mmol) and (cyclopropylmethyl)amine (0.15 mL, 1.70 mmol), compound **82** was obtained as a yellow oil (164 mg, 62%). Chromatography: DCM to DCM/EtOH/$NH_3$ 8:2:0.1.

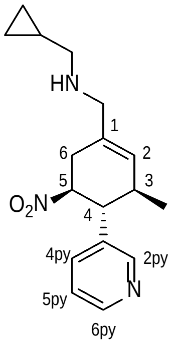


$R_f$: 0.81 (DCM/MeOH/$NH_3$ 8:2:0.1). $[\alpha]^D_{20}$ = -17.0 (c = 1.0, $CHCl_3$). IR (ATR): ν 3300 (NH), 1549 ($NO_2$). $^1$H-RMN ($CDCl_3$): δ 0.10-0.17 (m, 2H, $CH_{2cpr}$), 0.46-0.55 (m, 2H, $CH_{2cpr}$), 0.91 (d, *J* = 7.0, 3H, $CH_3$), 0.93-0.99 (m, 1H, $CH_{cpr}$), 2.47 (d, *J* = 6.8, 2H, NHC$\underline{H}_2$CH), 2.47-2.54 (m, 1H, $H_3$), 2.81 (d, *J* = 8.2, 2H, 2$H_6$), 2.92 (dd, *J* = 11.8, 10.4, 1H, $H_4$), 3.27 (s, 2H, NHC$\underline{H}_2$), 5.02 (ddd, *J* = 11.7, 9.0, 7.3, 1H, $H_5$), 5.58 (d, *J* = 1.7, 1H, $H_2$), 7.23-7.28 (m, 1H, $H_{5py}$), 7.51-7.55 (m, 1H, $H_{4py}$), 8.47 (dd, *J* = 2.4, 0.6, 1H, $H_{2py}$), 8.51 (dd, *J* = 4.8, 1.6, 1H, $H_{6py}$). $^{13}$C-NMR ($CDCl_3$): δ 3.6 (2$CH_{2cpr}$), 11.1 ($CH_{cpr}$), 19.5 ($CH_3$), 33.2 ($C_6$), 37.3 ($C_3$), 50.0 ($C_4$), 54.47 (NH$\underline{C}H_2$CH), 54.54 (NH$CH_2$), 87.9 ($C_5$), 123.8 ($C_{5py}$), 127.9 ($C_2$), 132.0 ($C_1$), 134.4 ($C_{4py}$), 135.3 ($C_{3py}$),149.3 ($C_{6py}$), 150.0 ($C_{2py}$). HPLC (method A, $t_R$, min): 8.57. MS (ESI, *m/z*, %): 302.1 ($[M+H]^+$, 100).

**1-Cyclopropyl-*N*-{[(3*R*,4*S*,5*S*)-3-methyl-5-nitro-4-(thiophen-2-yl)cyclohex-1-en-1-yl]methyl}methanamine, 83.** Following general procedure C using **68** (238 mg, 0.95 mmol) and (cyclopropylmethyl)amine (0.16 mL, 1.89 mmol), compound **83** was obtained as a yellow oil (123 mg, 44%). Chromatography: DCM to DCM/EtOH/$NH_3$ 8:2:0.1.

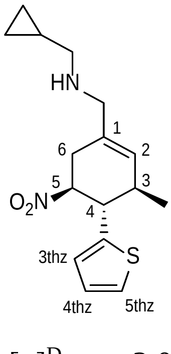


$R_f$: 0.80 (DCM/MeOH/$NH_3$ 8:2:0.1). $[\alpha]^D_{20}$ = -2.0 (c = 1.0, $CHCl_3$). IR (ATR): ν 3300 (NH), 1551 ($NO_2$). $^1$H-RMN ($CDCl_3$): δ 0.09-0.14 (m, 2H, $CH_{2cpr}$), 0.46-0.52 (m, 2H, $CH_{2cpr}$), 0.93-0.97 (m, 1H, $CH_{cpr}$), 1.00 (d, *J* = 7.0, 1H, $CH_3$), 2.45 (d, *J* = 6.8, 2H, NHC$\underline{H}_2$CH), 2.51-2.58 (m, 1H, $H_3$), 2.75-2.81 (m, 2H, 2$H_6$), 3.20-3.27 (m, 3H, NHC$\underline{H}_2$, $H_4$), 4.89 (ddd, *J* = 11.5, 9.8, 6.6, 1H, $H_5$), 5.55 (s, 1H, $H_2$), 6.87 (dd, *J* = 3.6, 1.3, 1H, $H_{3thz}$), 6.91 (dd, *J* = 5.1, 3.5, 1H, $H_{4thz}$), 7.20 (ddd, *J* = 5.0, 1.2, 0.6, 1H, $H_{5thz}$). $^{13}$C-NMR

($CDCl_3$): δ 3.5 ($2CH_{2cpr}$), 11.3 ($CH_{cpr}$), 19.9 ($CH_3$), 33.2 ($C_6$), 38.7 ($C_3$), 47.5 ($C_4$), 54.5 (NH<u>C</u>$H_2$CH), 54.7 ($NHCH_2$), 89.8 ($C_5$), 124.7 ($C_{5thz}$), 126.7 ($C_{4thz}$), 126.9 ($C_{3thz}$), 127.8 ($C_2$), 132.1 ($C_1$), 141.7 ($C_{2thz}$). HPLC (method A, $t_R$, min): 16.45. MS (ESI, *m/z*, %): 307.1 ($[M+H]^+$, 100).

**1-Cyclopropyl-*N*-{[(3*R*,4*S*,5*S*)-3-methyl-5-nitro-4-(1,3-oxazol-4-yl)cyclohex-1-en-1-yl]methyl}methanamine, 84.** Following general procedure C using **69** (170 mg, 0.72 mmol) and (cyclopropylmethyl)amine (0.13 mL, 1.44 mmol), compound **84** was obtained as a yellow oil (133 mg, 63%). Chromatography: DCM to DCM/EtOH/$NH_3$ 9:1:0.1.

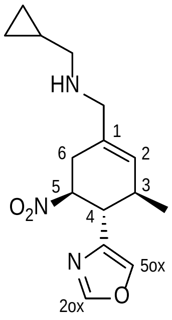


$R_f$: 0.40 (DCM/MeOH/$NH_3$ 9.5:0.5:0.1). $[\alpha]^D_{20}$ = 1.0 (c = 1.0, $CHCl_3$). IR (ATR): ν 3369 (NH), 1550 ($NO_2$). $^1$H-RMN ($CDCl_3$): δ 0.07-0.16 (m, 2H, $CH_{2cpr}$), 0.44-0.54 (m, 2H, $CH_{2cpr}$), 0.85-0.93 (m, 1H, $CH_{cpr}$), 0.95 (d, *J* = 6.8, 3H, $CH_3$), 1.49 (br s, 1H, NH), 2.43 (d, *J* = 6.8, 2H, NHC<u>H</u>$_2$CH) 2.65-2.84 (m, 3H, $H_3$, $2H_6$), 2.95 (t, *J* = 9.8, 1H, $H_4$), 3.23 (s, 2H, NHC<u>H</u>$_2$), 5.01-5.15 (m, 1H, $H_5$), 5.54 (s, 1H, $H_2$), 7.51 (d, *J* = 1.0, 1H, $H_{5ox}$), 7.83 (s, 1H, $H_{2ox}$). $^{13}$C-NMR ($CDCl_3$): δ 3.5 ($2CH_{2cpr}$), 11.3 ($CH_{cpr}$), 19.7 ($CH_3$), 32.7 ($C_6$), 34.2 ($C_3$), 43.2 ($C_4$), 54.5 (NH<u>C</u>$H_2$CH), 54.8 ($NHCH_2$), 86.5 ($C_5$), 127.7 ($C_2$), 131.8 ($C_1$), 137.1 ($C_{5ox}$), 137.4 ($C_{4ox}$), 151.6 ($C_{2ox}$). HPLC (method A, $t_R$, min): 16.59. MS (ESI, *m/z*, %): 292.2 ($[M+H]^+$, 100).

**(1*R*,2*S*,6*R*)-4-{[(Cyclopropylmethyl)amino]methyl}-3'-methoxy-6-methyl-1,2,3,6-tetrahydro[1,1'-biphenyl]-2-amine, 18.** Following general procedure D using **70** (100 mg, 0.30 mmol), compound **18** was obtained as a yellow oil (50 mg, 55%). Chromatography: DCM to DCM/EtOH/$NH_3$ 8:2:0.1.

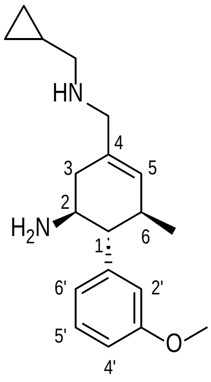

$R_f$: 0.30 (DCM/MeOH/$NH_3$ 8:2:0.1). $[\alpha]^D_{20}$ = +1.0 (c = 1.00, $CHCl_3$). IR (ATR): ν 3377 ($NH_2$), 1454 (C-N), 1264 (COC). $^1$H-NMR ($CDCl_3$): δ 0.08-0.25 (m, 2H, $CH_{2cpr}$), 0.44-0.56 (m, 2H, $CH_{2cpr}$), 0.79 (d, *J* = 7.0, 3H, $CH_3$), 0.90-1.07 (m, 1H, $CH_{cpr}$), 1.96-2.07 (m, 1H, $H_3$), 2.10 (t, *J* = 10.5, 1H, $H_1$), 2.30-2.49 (m, 2H, $H_3$, $H_6$), 2.53 (d, *J* = 7.0, 2H, $NHC\underline{H}_2CH$), 3.19 (td, *J* = 10.5, 5.1, 1H, $H_2$), 3.29 (s, 2H, $NHC\underline{H}_2$), 3.76 (s, 3H, $OCH_3$), 5.52 (s, 1H, $H_5$), 6.67-6.83 (m, 3H, $H_{2'}$, $H_{4'}$, $H_{6'}$), 7.14-7.24 (m, 1H, $H_{5'}$). $^{13}$C-NMR ($CDCl_3$): δ 3.8 ($CH_{2cpr}$), 3.9 ($CH_{2cpr}$), 9.7 ($CH_{cpr}$), 19.8 ($CH_3$), 35.6 ($C_3$), 37.8 ($C_6$), 51.7 ($C_2$), 53.2 ($NH\underline{C}H_2CH$), 53.9 ($NHCH_2$), 55.2 ($OCH_3$), 56.7 ($C_1$), 112.0 ($C_{4'}$), 114.4 ($C_{2'}$) 120.9 ($C_{6'}$), 129.8 ($C_{5'}$), 131.0 ($C_5$), 131.1 ($C_4$), 143.5 ($C_{1'}$), 159.9 ($C_{3'}$). HPLC (method A, $t_R$, min): 11.73. MS (ESI, *m/z*, %): 301.3 ($[M+H]^+$, 100). Elemental analysis calculated for $C_{19}H_{28}N_2O{\cdot}2HCl{\cdot}H_2O$: %C 58.31, %H 8.24, %N 7.16; experimental: %C 57.89, %H 7.95, %N: 6.93.

**(1*R*,2*S*,6*R*)-4-{[(Cyclopropylmethyl)amino]methyl}-2'-methoxy-6-methyl-1,2,3,6-tetrahydro[1,1'-biphenyl]-2-amine, 19.** Following general procedure D using **71** (170 mg, 0.51 mmol), compound **19** was obtained as a yellow oil (30 mg, 20%). Chromatography: DCM to DCM/EtOH/$NH_3$ 8:2:0.1.

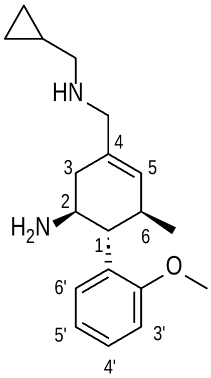

$R_f$: 0.04 (DCM/MeOH/$NH_3$ 8:2:0.1). $[\alpha]^D_{20}$ = +37.0 (c = 1.00, $CHCl_3$). IR (ATR): ν 3356 ($NH_2$), 1492 (C-N), 1241 (COC). $^1$H-NMR ($CDCl_3$, 700 MHz): δ 0.14-0.21 (m, 2H,

$CH_{2cpr}$), 0.47-0.54 (m, 2H, $CH_{2cpr}$), 0.78 (d, 3H, *J* = 7.0, $CH_3$), 1.00-1.07 (m, 1H, $CH_{cpr}$), 2.00-2.08 (m, 1H, $H_3$), 2.29-2.39 (m, 1H, $H_6$), 2.42-2.49 (m, 1H, $H_3$), 2.53 (d, *J* = 6.9, 2H, NHC$\underline{H}_2$CH), 2.70-3.17 (br m, 5H, $H_1$, $H_2$, $NH_2$, NH), 3.27 (AB system, *J* = 14.7, 2H, NHC$\underline{H}_2$), 3.76 (s, 3H, $OCH_3$), 5.51 (s, 1H, $H_5$), 6.81-6.88 (m, 1H, $H_{3'}$), 6.89-7.00 (m, 1H, $H_{4'}$), 7.09-7.16 (m, 1H, $H_{6'}$), 7.18 (t, 1H, *J* = 7.7, $H_{5'}$). $^{13}$C-NMR ($CDCl_3$, 175 MHz): δ 3.76 ($CH_{2cpr}$), 3.78 ($CH_{2cpr}$), 10.5 ($CH_{cpr}$), 19.7 ($CH_3$), 36.7 ($C_3$), 38.4 ($C_6$), 46.9 ($C_1$), 52.0 ($C_2$), 54.0 (NH$\underline{C}H_2$CH), 54.7 ($NHCH_2$), 55.7 ($OCH_3$), 110.8 ($C_{3'}$), 121.2 ($C_{4'}$), 126.4 ($C_{6'}$), 127.3 ($C_{5'}$), 130.3 ($C_5$), 131.0 ($C_4$), 132.3 ($C_{1'}$), 158.9 ($C_{2'}$). HPLC (method A, $t_R$, min): 11.90. MS (ESI, *m/z*, %): 301.2 ($[M+H]^+$, 100). Elemental analysis calculated for $C_{19}H_{28}N_2O·2HCl·H_2O$: %C 58.31, %H 8.24, %N 7.16; experimental: %C 57.92, %H 7.86, %N: 6.80.

**(1*R*,2*S*,6*R*)-4-{[(Cyclopropylmethyl)amino]methyl}-6-methyl-1,2,3,6-tetrahydro [1,1'-biphenyl]-2-amine, 20.** Following general procedure D using **72** (210 mg, 0.70 mmol), compound **20** was obtained as a yellow oil (31 mg, 16%). Chromatography: DCM to DCM/EtOH/$NH_3$ 8:2:0.1.

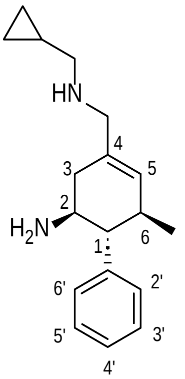


$R_f$: 0.4 (DCM/MeOH/$NH_3$ 8:2:0.1). $[\alpha]^D{}_{20}$ = -3.0 (c = 1.00, $CHCl_3$. IR (ATR): ν 3363 ($NH_2$), 1453 (C-N). $^1$H-NMR ($CDCl_3$): δ 0.08-0.17 (m, 2H, $CH_{2cpr}$), 0.43-0.54 (m, 2H, $CH_{2cpr}$), 0.80 (d, *J* = 7.0, 3H, $CH_3$), 0.92-1.05 (m, 1H, $CH_{cpr}$), 1.78 (br s, 3H, $NH_2$, NH), 1.90-2.02 (m, 1H, $H_3$), 2.05 (t, *J* = 10.4, 1H, $H_1$), 2.33-2.44 (m, 2H, $H_3$, $H_6$), 2.46 (d, *J* = 6.9, 2H, NHC$\underline{H}_2$CH), 3.13-3.28 (m, 3H, $H_2$, NHC$\underline{H}_2$), 5.47 (s, 1H, $H_5$), 7.14-7.25 (m, 3H, $H_{2'}$, $H_{4'}$, $H_{6'}$), 7.27-7.36 ($H_{3'}$, $H_{5'}$). $^{13}$C-NMR ($CDCl_3$): δ 3.5 ($CH_{2cpr}$), 3.6 ($CH_{2cpr}$), 11.2 ($CH_{cpr}$), 20.1 ($CH_3$), 36.9 ($C_3$), 38.0 ($C_6$), 51.8 ($C_2$), 55.4 (NH$\underline{C}H_2$CH), 55.2 ($NHCH_2$), 58.2 ($C_1$), 126.7 ($C_{4'}$), 128.6 ($C_5$), 128.7 ($C_{2'}$, $C_{6'}$), 128.7 ($C_{3'}$, $C_{5'}$), 133.5 ($C_4$), 142.7 ($C_{1'}$). HPLC (method A, $t_R$, min): 11.11. MS (ESI, *m/z*, %): 271.2 ($[M+H]^+$, 100). Elemental

analysis calculated for $C_{18}H_{26}N_2·2HCl·1/2H_2O$: %C 58.38, %H 8.44, %N 7.56; experimental: %C 58.76, %H 8.04, %N: 6.96.

**(1*R*,2*S*,6*R*)-4-{[(Cyclopropylmethyl)amino]methyl}-4',6-dimethyl-1,2,3,6-tetrahydro[1,1'-biphenyl]-2-amine, 21.** Following general procedure D using **73** (153 mg, 0.49 mmol), compound **21** was obtained as a yellow oil (90 mg, 65%). Chromatography: DCM to DCM/EtOH/$NH_3$ 8:2:0.1.

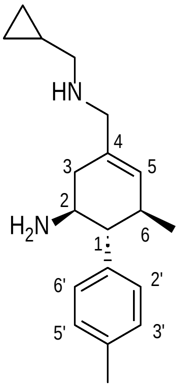


$R_f$: 0.38 (DCM/MeOH/$NH_3$ 8:2:0.1). $[\alpha]^D_{20}$ = -5.0 (c = 1.0, $CHCl_3$). IR (ATR): ν 3300 ($NH_2$). $^1$H-RMN ($CDCl_3$): δ 0.11-0.16 (m, 2H, $CH_{2cpr}$), 0.47-0.53 (m, 2H, $CH_{2cpr}$), 0.81 (d, *J* = 7.0, 3H, $CH\underline{H}_3$), 0.95-1.05 (m, 1H, $CH_{cpr}$), 1.73 (br s, 3H, $NH_2$, NH), 1.92-2.00 (m, 1H, $H_3$), 2.02 (t, *J* = 10.5, 1H, $H_1$), 2.33 (s, 1H, $C_{Ar}C\underline{H}_3$), 2.33-2.43 (m, 2H, $H_3$, $H_6$), 2.48 (d, *J* = 6.9, 2H, $NHC\underline{H}_2CH$), 3.17 (td, *J* = 10.5, 5.2, 1H, $H_2$), 3.23 (s, 2H, $NHC\underline{H}_2$), 5.48 (s, 1H, $H_5$), 7.07 (d, *J* = 8.2, 2H, $H_{3'}$, $H_{5'}$), 7.13 (d, *J* = 8.2, 2H, $H_{2'}$, $H_{6'}$).$^{13}$C-NMR ($CDCl_3$): δ 3.60 ($CH_{2cpr}$), 3.61 ($CH_{2cpr}$), 11.2 ($CH_{cpr}$), 20.2 ($CHC\underline{H}_3$), 21.2 ($C_{Ar}CH_3$), 36.9 ($C_3$), 38.1 ($C_6$), 51.8 ($C_2$), 54.4 ($NH\underline{C}H_2CH$), 55.1 ($NHCH_2$), 57.7 ($C_1$), 128.5 ($C_{2'}$, $C_{6'}$), 128.9 ($C_5$), 129.5 ($C_{3'}$, $C_{5'}$), 133.3 ($C_4$), 136.2 ($C_{4'}$), 139.5 ($C_{1'}$). HPLC (method A, $t_R$, min): 15.29. MS (ESI, *m/z*, %): 285.3 ($[M+H]^+$, 100). Elemental analysis calculated for $C_{19}H_{28}N_2·2HCl·4H_2O$: %C 53.14, %H 8.92, %N 6.52; experimental: %C 53.12, %H 8.54, %N: 6.33.

**(1*R*,2*S*,6*R*)-4-{[(Cyclopropylmethyl)amino]methyl}-4'-chloro-6-methyl-1,2,3,6-tetrahydro[1,1'-biphenyl]-2-amine, 22.** Following general procedure D using **74** (125 mg, 0.37 mmol), compound **22** was obtained as a yellow oil (50 mg, 44%). Chromatography: DCM to DCM/EtOH/$NH_3$ 8:2:0.1.

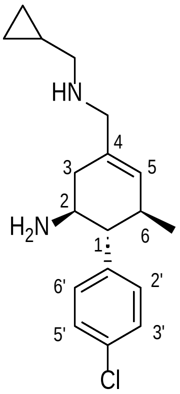


$R_f$: 0.43 (DCM/MeOH/$NH_3$ 9:1:0.1). $[\alpha]^D_{20}$ = -2.0 (c = 1.0, $CHCl_3$). IR (ATR): ν 3300 ($NH_2$). $^1$H-RMN ($CDCl_3$): δ 0.08-0.15 (m, 2H, $CH_{2cpr}$), 0.44-0.50 (m, 2H, $CH_{2cpr}$), 0.78 (d, *J* = 6.9, 3H, $CH_3$), 0.90-1.00 (m, 1H, $CH_{cpr}$), 1.41 (m, 3H, $NH_2$, NH), 1.89-2.00 (m, 1H, $H_3$), 2.03 (t, *J* = 10.5, 1H, $H_1$), 2.32-2.37 (m, 2H, $H_3$, $H_6$), 2.44 (d, *J* = 6.9, 2H, NHC$\underline{H}_2$CH), 3.15 (td, *J* = 10.6, 5.3, 1H, $H_2$), 3.19 (s, 2H, NHC$\underline{H}_2$), 5.44 (s, 1H, $H_5$), 7.12 (d, *J* = 8.4, 2H, $H_{3'}$, $H_{5'}$), 7.28 (d, *J* = 8.4, 2H, $H_{2'}$, $H_{6'}$). $^{13}$C-RMN ($CDCl_3$): δ 3.50 ($CH_{2cpr}$), 3.51 ($CH_{2cpr}$), 11.2 ($CH_{cpr}$), 20.0 ($CH_3$), 36.9 ($C_3$), 38.0 ($C_6$), 51.7 ($C_2$), 54.4 (NH$\underline{C}H_2$CH), 55.1 ($NHCH_2$), 57.6 ($C_1$), 128.1 ($C_5$), 128.8 ($C_{3'}$, $C_{5'}$), 129.8 ($C_{2'}$, $C_{6'}$), 132.2 ($C_{4'}$), 133.7 ($C_4$), 141.3 ($C_{1'}$). HPLC (method A, $t_R$, min): 11.52. MS (ESI, *m/z*, %): 305.2 ($[M+H]^+$, 100). Elemental analysis calculated for $C_{18}H_{25}ClN_2 \cdot 2HCl \cdot 2H_2O$: %C 52.25, %H 7.55, %N 6.77; experimental: %C 52.66, %H 7.16, %N: 6.38.

**(1*R*,2*S*,6*R*)-4-{[(Cyclopropylmethyl)amino]methyl}-3'-chloro-6-methyl-1,2,3,6-tetrahydro[1,1'-biphenyl]-2-amine, 23.** Following general procedure D using **75** (210 mg, 0.63 mmol), compound **23** was obtained as a colorless oil (88 mg, 46%). Chromatography: DCM to DCM/EtOH/$NH_3$ 8.5:1.5:0.1.

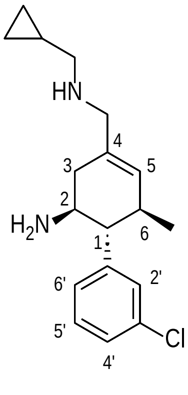


$R_f$: 0.40 (DCM/MeOH/$NH_3$ 9:1:0.1). $[\alpha]^D_{20}$ = -17.0 (c = 1.0, $CHCl_3$). IR (ATR): ν 3318 ($NH_2$), 1455 (C-N). $^1$H-RMN ($CDCl_3$): δ 0.07-0.18 (m, 2H, $CH_{2cpr}$), 0.44-0.55 (m, 2H, $CH_{2cpr}$), 0.81 (d, *J* = 6.9, 3H, $CH_3$), 0.92-1.04 (m, 1H, $CH_{cpr}$), 1.30 (br s, 3H, $NH_2$, NH),

1.87-2.03 (m, 1H, $H_3$), 2.05 (t, *J* = 10.5, 1H, $H_1$), 2.30-2.44 (m, 2H, $H_3$, $H_6$), 2.46 (d, *J* = 6.9, 2H, NHC$\underline{H}_2$CH), 3.13-3.82 (m, 3H, $H_2$, NHC$\underline{H}_2$), 5.46 (s, 1H, $H_5$), 7.09 (dt, *J* = 7.2, 1.7, 1H, $H_{6'}$), 7.20 (d, *J* = 1.6, 1H, $H_{2'}$), 7.21-7.24 (m, 2H, $H_{4'}$, $H_{5'}$). $^{13}$C-RMN ($CDCl_3$): δ 3.52 ($CH_{2cpr}$), 3.54 ($CH_{2cpr}$), 11.4 ($CH_{cpr}$), 20.1 ($CH_3$), 37.0 ($C_3$), 38.0 ($C_6$), 51.8 ($C_2$), 54.5 (NH$\underline{C}H_2$CH), 55.2 ($NHCH_2$), 58.2 ($C_1$), 127.0 ($C_{6'}$), 128.1 ($C_5$, $C_{2'}$), 128.6 ($C_{4'}$), 130.0 ($C_{5'}$), 133.9 ($C_4$), 134.6 ($C_{3'}$), 145.2 ($C_{1'}$). HPLC (method A, $t_R$, min): 12.10. MS (ESI, *m/z*, %): 305.2 ($[M+H]^+$, 100). Elemental analysis calculated for $C_{18}H_{25}ClN_2$·2HCl·$2H_2O$: %C 52.25, %H 7.55, %N 6.77; experimental: %C 52.61, %H 7.08, %N: 6.59.

**(1*R*,2*S*,6*R*)-4-{[(Cyclopropylmethyl)amino]methyl}-2'-chloro-6-methyl-1,2,3,6-tetrahydro[1,1'-biphenyl]-2-amine, 24.** Following general procedure D using **76** (100 mg, 0.30 mmol), compound **24** was obtained as a colorless oil (54 mg, 59%). Chromatography: DCM to DCM/EtOH/$NH_3$ 8.5:1.5:0.1.

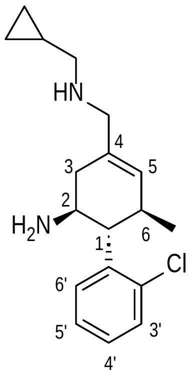


$R_f$: 0.30 (DCM/MeOH/$NH_3$ 9:1:0.1). $[\alpha]^D_{20}$ = -28.0 (c = 1.0, $CHCl_3$). IR (ATR): ν 1475 (C-N). $^1$H-RMN ($CDCl_3$): δ 0.07-0.17 (m, 2H, $CH_{2cpr}$), 0.44-0.55 (m, 2H, $CH_{2cpr}$), 0.83 (d, *J* = 7.0, 3H, $CH_3$), 0.90-1.04 (m, 1H, $CH_{cpr}$), 1.32 (br s, $NH_2$, NH), 1.93-2.08 (m, 1H, $H_3$), 2.32-2.47 (m, 2H, $H_3$, $H_6$,), 2.46 (dd, *J* = 6.8, 1.5, 1H, NHC$\underline{H}_2$CH), 2.94 (t, *J* = 10.4, 1H, $H_1$), 3.13 (td, *J* = 10.4, 5.1, 1H, $H_2$), 3.21 (s, 2H, NHC$\underline{H}_2$), 5.47 (s, 1H, $H_5$), 7.09-7.21 (m, 1H, $H_{5'}$), 7.19-7.31 (m, 2H, $H_{4'}$, $H_{6'}$), 7.39 (dd, *J* = 7.9, 1.3, 1H, $H_{3'}$).$^{13}$C-RMN ($CDCl_3$): δ 3.50 ($CH_{2cpr}$), 3.53 ($CH_{2cpr}$), 11.4 ($CH_{cpr}$), 19.7 ($CH_3$), 37.4 ($C_3$), 38.9 ($C_6$), 52.6 ($C_2$), 52.7 ($C_1$), 54.6 (NH$\underline{C}H_2$CH), 55.2 ($NHCH_2$), 127.4 ($C_{5'}$), 127.5 ($C_{4'}$), 127.6 ($C_{6'}$), 128.2 ($C_5$), 129.6 ($C_{3'}$), 134.1 ($C_4$), 136.5 ($C_{2'}$), 140.9 ($C_{1'}$). HPLC (method A, $t_R$, min): 11.77. MS (ESI, *m/z*, %): 305.1 ($[M+H]^+$, 100). Elemental analysis calculated for $C_{18}H_{25}ClN_2$·2HCl·$3H_2O$: %C 50.07, %H 7.70, %N 6.49; experimental: %C 50.43, %H 7.36, %N: 6.15.

**(1*R*,2*S*,6*R*)-4-{[(Cyclopropylmethyl)amino]methyl}-3'-fluoro-6-methyl-1,2,3,6-tetrahydro[1,1'-biphenyl]-2-amine, 26.** Following general procedure D using **78** (650 mg, 2.04 mmol), compound **26** was obtained as a colorless oil (388 mg, 66%). Chromatography: DCM to DCM/EtOH/$NH_3$ 8.5:1.5:0.1.

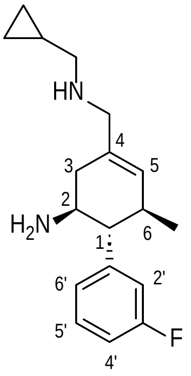


$R_f$: 0.30 (DCM/MeOH/$NH_3$ 8:2:0.1). $[\alpha]^D{}_{20}$ = -18.0 (c = 1.0, $CHCl_3$). IR (ATR): ν 3305 ($NH_2$), 1448 (C-N). $^1$H-RMN ($CDCl_3$): δ .07-0.17 (m, 2H, $CH_{2cpr}$), 0.43-0.52 (m, 2H, $CH_{2cpr}$), 0.80 (d, *J* = 6.9, 3H, $CH_3$), 0.92-1.05 (m, 1H, $CH_{cpr}$), 1.59 (br s, 3H, $NH_2$, NH), 1.90-2.02 (m, 1H, $H_3$), 2.07 (t, *J* = 10.4, 1H, $H_1$), 2.29-2.42 (m, 2H, $H_3$, $H_6$), 2.46 (d, *J* = 6.9, 2H, NHC$\underline{H}_2$CH), 3.11-3.19 (m, 1H, $H_2$) 3.21 (s, 2H, NHC$\underline{H}_2$), 5.46 (s, 1H, $H_5$), 6.86-7.02 (m, 3H, $H_{2'}$, $H_{4'}$, $H_{6'}$), 7.22-7.32 (m, 1H, $H_{5'}$). $^{13}$C-RMN ($CDCl_3$): δ 3.5 ($CH_{2cpr}$), 3.6 ($CH_{2cpr}$), 11.2 ($CH_{cpr}$), 20.1 ($CH_3$), 36.9 ($C_3$), 38.0 ($C_6$), 51.8 ($C_2$), 54.4 (NH$\underline{C}H_2$CH), 55.1 ($NHCH_2$), 58.1 (d, *J* = 1.6, $C_1$), 113.6 (d, *J* = 21.0, $C_{4'}$), 115.2 (d, *J* = 20.8, $C_{2'}$), 124.4 (d, *J* = 2.3, $C_{6'}$), 128.4 ($C_5$), 130.1 (d, *J* = 8.3, $C_{5'}$), 133.6 ($C_4$), 145.7 (d, *J* = 6.8, $C_{1'}$), 163.2 (d, *J* = 246.0, $C_{3'}$). HPLC (method A, $t_R$, min): 10.22. MS (ESI, *m/z*, %): 289.2 ($[M+H]^+$, 100). Elemental analysis calculated for $C_{18}H_{25}FN_2 \cdot 2HCl \cdot 3/2H_2O$: %C 50.94, %H 8.08, %N 6.68; experimental: %C 51.18, %H 7.72, %N: 6.68.

**(1*R*,2*S*,6*R*)-4-{[(Cyclopropylmethyl)amino]methyl}-2'-fluoro-6-methyl-1,2,3,6-tetrahydro[1,1'-biphenyl]-2-amine, 27.** Following general procedure D using **79** (325 mg, 1.02 mmol), compound **27** was obtained as a colorless oil (120 mg, 41%). Chromatography: DCM to DCM/EtOH/$NH_3$ 8.5:1.5:0.1.

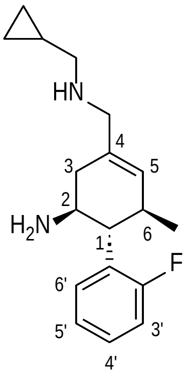


$R_f$: 0.30 (DCM/MeOH/NH$_3$ 8:2:0.1). $[\alpha]^D_{20}$ = 6.0 (c = 1.0, CHCl$_3$). IR (ATR): ν 3286 (NH$_2$), 1453 (C-N). $^1$H-RMN (CDCl$_3$): δ 0.08-0.21 (m, 2H, CH$_{2cpr}$), 0.45-0.56 (m, 2H, CH$_{2cpr}$), 0.83 (d, *J* = 6.5, 3H, CH$_3$), 0.91-1.07 (m, 1H, CH$_{cpr}$), 1.72 (br s, 3H, NH$_2$, NH), 1.89-2.05 (m, 1H, H$_3$), 2.34-2.57 (m, 5H, H$_1$, H$_3$, H$_6$,NHC$\underline{H}_2$CH), 3.15-3.32 (m, 3H, H$_2$, NHC$\underline{H}_2$), 5.48 (s, 1H, H$_5$), 6.99-7.08 (m, 1H, H$_{3'}$), 7.09-7.15 (m, 1H, H$_{5'}$), 7.15-7.24 (m, 2H, H$_{4'}$, H$_{6'}$). $^{13}$C-RMN (CDCl$_3$): δ 3.56 (CH$_{2cpr}$), 3.59 (CH$_{2cpr}$), 11.2 (CH$_{cpr}$), 20.1 (CH$_3$), 37.2 (C$_3$), 37.3 (C$_6$), 51.1 (br, C$_1$, C$_2$), 54.4 (NH$\underline{C}$H$_2$CH), 55.1 (NHCH$_2$), 115.7 (d, *J* = 23.4, C$_{3'}$) 124.6 (d, *J* = 3.4, C$_{5'}$), 128.0 (d, *J* = 8.3, C$_{4'}$, C$_{6'}$), 128.6 (C$_5$), 129.5 (d, *J* = 14.7, C$_{1'}$), 133.7 (C$_4$), 162.1 (d, *J* = 246.4, C$_{2'}$). HPLC (method A, t$_R$, min): 10.05. MS (ESI, *m/z*, %): 289.2 ([M+H]$^+$, 100). Elemental analysis calculated for $C_{18}H_{25}FN_2 \cdot 2HCl \cdot 5H_2O$: %C 47.89, %H 8.26, %N 6.21; experimental: %C 48.01, %H 7.89, %N: 5.83.

**(1*R*,2*S*,6*R*)-4-{[(Cyclopropylmethyl)amino]methyl}-6-methyl-4'-(trifluoromethyl)-1,2,3,6-tetrahydro[1,1'-biphenyl]-2-amine, 28.** Following general procedure D using **80** (51 mg, 0.14 mmol), compound **28** was obtained as a yellow oil (14 mg, 30%). Chromatography: DCM to DCM/EtOH/NH$_3$ 8:2:0.1.

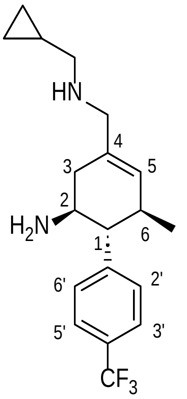


$R_f$: 0.89 (DCM/MeOH/NH$_3$ 8:2:0.1). $[\alpha]^D_{20}$ = -4.0 (c = 1.0, CHCl$_3$). IR (ATR): ν 3300 (NH$_2$). $^1$H-NMR (CDCl$_3$): δ 0.19-0.24 (m, 2H, CH$_{2cpr}$), 0.52-0.58 (m, 2H, CH$_{2cpr}$), 0.81 (d, *J* = 7.0, 3H, CH$_3$), 1.02-1.09 (m, 1H, CH$_{cpr}$), 2.03-2.11 (m, 1H, H$_3$), 2.22 (t, *J* = 10.4,

1H, $H_1$), 2.42-2.52 (m, 2H, $H_3$, $H_6$), 2.57 (d, *J* = 7.0, 2H, NHC$\underline{H}_2$CH), 3.02 (s, 3H, $NH_2$, NH), 3.26 (td, *J* = 10.3, 5.2, 1H, $H_2$), 3.33 (s, 2H, NHC$\underline{H}_2$), 5.56 (s, 1H, $H_5$), 7.33 (d, *J* = 8.0, 2H, $H_{2'}$, $H_{6'}$), 7.59 (d, *J* = 8.0, 2H, $H_{3'}$, $H_{5'}$). $^{13}$C-RMN ($CDCl_3$): δ 3.9 ($2CH_{2cpr}$), 10.2 ($CH_{cpr}$), 19.9 ($CH_3$), 36.5 ($C_3$), 38.0 ($C_6$), 51.7 ($C_2$), 53.8 (NH$\underline{C}H_2$CH), 54.3 ($NHCH_2$), 57.4 ($C_1$), 122.3 (q, *J* = 275.1, $CF_3$), 125.8 (d, *J* = 3.6, $C_{3'}$, $C_{5'}$), 129.0 ($C_{2'}$, $C_{6'}$), 129.1 (q, *J* = 31.5, $C_{4'}$), 130.4 ($C_5$), 132.0 ($C_4$), 146.9 ($C_{1'}$). HPLC (method A, $t_R$, min): 12.41. MS (ESI, *m/z*, %): 339.2 ($[M+H]^+$, 100). Elemental analysis calculated for $C_{19}H_{25}F_3N_2 \cdot 2HCl \cdot 7/2H_2O$: %C 48.11, %H 7.22, %N 5.91; experimental: %C 48.36, %H 6.88, %N: 5.54.

**(1*R*,2*S*,6*R*)-4-{[(Cyclopropylmethyl)amino]methyl}-6-methyl-4'-(trifluoromethoxy) -1,2,3,6-tetrahydro[1,1'-biphenyl]-2-amine, 29.** Following general procedure D using **81** (98 mg, 0.26 mmol), compound **29** was obtained as a yellow oil (23 mg, 26%). Chromatography: DCM to DCM/EtOH/$NH_3$ 8:2:0.1.

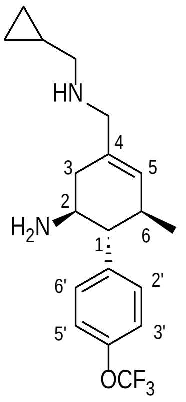


$R_f$: 0.35 (DCM/MeOH/$NH_3$ 8:2:0.1). $[\alpha]^D_{20}$ = -2.0 (c = 1.0, $CHCl_3$). IR (ATR): ν 3300 ($NH_2$). $^1$H-NMR ($CDCl_3$): δ 0.10-0.15 (m, 2H, $CH_{2cpr}$), 0.46-0.52 (m, 2H, $CH_{2cpr}$), 0.80 (d, *J* = 6.9, 3H, $CH_3$), 0.94-1.01 (m, 1H, $CH_{cpr}$), 1.59 (s, 3H, $NH_2$, NH), 1.93-2.02 (m, 1H, $H_3$), 2.09 (t, *J* = 10.5, 1H, $H_1$), 2.36-2.46 (m, 2H, $H_3$, $H_6$), 2.47 (d, *J* = 6.9, 2H, NHC$\underline{H}_2$CH), 3.16-3.27 (m, 3H, $H_2$, NHC$\underline{H}_2$), 5.48 (s, 1H, $H_5$), 7.17 (d, *J* = 8.8, 2H, $H_{3'}$, $H_{5'}$), 7.22 (d, *J* = 8.9, 2H, $H_{2'}$, $H_{6'}$). $^{13}$C-RMN ($CDCl_3$): δ 3.6 ($2CH_{2cpr}$), 11.2 ($CH_{cpr}$), 20.1 ($CH_3$), 36.9 ($C_3$), 38.1 ($C_6$), 51.7 ($C_2$), 54.4 (NH$\underline{C}H_2$CH), 55.1 ($NHCH_2$), 57.6 ($C_1$), 120.6 (q, *J* = 256.9, $OCF_3$), 121.2 ($C_{3'}$, $C_{5'}$), 128.4 ($C_5$), 129.8 ($C_{2'}$, $C_{6'}$), 133.6 ($C_4$), 141.6 ($C_{1'}$), 148.0 (q, *J* = 1.7, $C_{4'}$). HPLC (method A, $t_R$, min): 11.92. MS (ESI, *m/z*, %): 355.2 ($[M+H]^+$, 100). Elemental analysis calculated for $C_{19}H_{25}F_3N_2O$ $\cdot 2HCl \cdot 9/2H_2O$: %C 44.89, %H 7.14, %N 5.51; experimental: %C 44.86, %H6.92, %N: 5.12.

**(1*S*,5*R*,6*R*)-3-{[(Cyclopropylmethyl)amino]methyl}-5-methyl-6-(pyridin-3-yl) cyclohex-3-en-1-amine, 30.** Following general procedure D using **82** (56 mg, 0.19 mmol), compound **30** was obtained as a yellow oil (31 mg, 62%). Chromatography: DCM to DCM/EtOH/$NH_3$ 8:2:0.1.

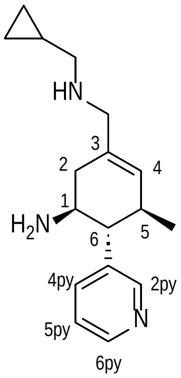


$R_f$: 0.26 (DCM/MeOH/$NH_3$ 8:2:0.1). $[\alpha]^D_{20}$ = -3.0 (c = 1.0, $CHCl_3$). IR (ATR): ν 3300 ($NH_2$). $^1$H-NMR ($CDCl_3$): δ 0.12-0.17 (m, 2H, $CH_{2cpr}$), 0.48-0.52 (m, 2H, $CH_{2cpr}$), 0.81 (d, *J* = 6.9, 3H, $CH_3$), 0.95-1.05 (m, 1H, $CH_{cpr}$), 1.90-2.20 (s, 3H, $NH_2$, NH), 1.95-2.05 (m, 1H, $H_2$), 2.12 (t, *J* = 10.5, 1H, $H_6$), 2.39-2.49 (m, 2H, $H_2$, $H_5$), 2.49 (d, *J* = 6.9, 2H, $NHC\underline{H}_2CH$), 3.20-3.30 (m, 1H, $H_1$), 3.25 (s, 2H, $NHC\underline{H}_2$) 5.50 (s, 1H, $H_4$), 7.28 (dd, *J* = 7.5, 4.9, 1H, $H_{5py}$), 7.53 (dt, *J* = 7.8, 2.0, 1H, $H_{4py}$), 8.48 (d, *J* = 1.9, 1H, $H_{2py}$), 8.50 (dd, *J* = 4.8, 1.6, 1H, $H_{6py}$). $^{13}$C-RMN ($CDCl_3$): δ 3.6 ($2CH_{2cpr}$), 11.1 ($CH_{cpr}$), 20.0 ($CH_3$), 36.9 ($C_2$), 37.9 ($C_5$), 51.6 ($C_1$), 54.3 ($NH\underline{C}H_2CH$), 55.0 ($NHCH_2$), 55.6 ($C_6$), 123.9 ($C_{5py}$), 128.6 ($C_4$), 133.5 ($C_3$), 135.7 ($C_{4py}$), 138.2 ($C_{3py}$), 148.5 ($C_{6py}$), 150.7 ($C_{2py}$). HPLC (method A, $t_R$, min): 3.32. MS (ESI, *m/z*, %): 272.2 ($[M+H]^+$, 100). Elemental analysis calculated for $C_{17}H_{25}N_3$ 3HCl·$8H_2O$: %C 38.90, %H 8.45, %N 8.01; experimental: %C 38.61, %H 8.09, %N: 6.62.

**(1*S*,5*R*,6*S*)-3-{[(Cyclopropylmethyl)amino]methyl}-5-methyl-6-(thiophen-2-yl) cyclohex-3-en-1-amine, 31.** Following general procedure D using **83** (96 mg, 0.31 mmol), compound **31** was obtained as a yellow oil (70 mg, 81%). Chromatography: DCM to DCM/EtOH/$NH_3$ 8:2:0.1.

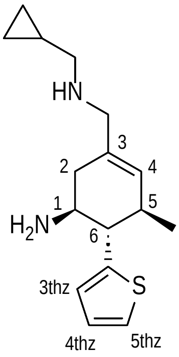


$R_f$: 0.21 (DCM/MeOH/$NH_3$ 8:2:0.1). $[\alpha]^D_{20}$ = -42.0 (c = 1.0, $CHCl_3$). IR (ATR): ν 3300 ($NH_2$). $^1$H-NMR ($CDCl_3$): δ 0.10-0.15 (m, 2H, $CH_{2cpr}$), 0.48-0.51 (m, 2H, $CH_{2cpr}$), 0.91 (d, *J* = 6.6, 3H, $CH_3$), 0.93-1.01 (m, 1H, $CH_{cpr}$), 1.67 (s, 3H, $NH_2$, NH), 1.93-2.02 (m, 1H, $H_2$), 2.36-2.43 (m, 3H, $H_2$, $H_5$, $H_6$), 2.46 (d, *J* = 6.9, 2H, NHC$\underline{H}_2$CH), 3.10 (td, *J* = 10.3, 5.3, 1H, $H_1$), 3.21 (s, 2H, NHC$\underline{H}_2$) 5.46 (s, 1H, $H_4$), 6.90 (dd, *J* = 3.4, 1.2, 1H, $H_{3thz}$), 6.96 (dd, *J* = 5.1, 3.4, 1H, $H_{4thz}$), 7.21 (dd, *J* = 5.1, 1.2, 1H, $H_{5thz}$). $^{13}$C-RMN ($CDCl_3$): δ 3.6 (2$CH_{2cpr}$), 11.2 ($CH_{cpr}$), 20.4 ($CH_3$), 36.8 ($C_2$), 39.5 ($C_5$), 53.3 ($C_1$), 53.7 ($C_6$), 54.4 (NHC$\underline{H}_2$CH), 55.1 (NH$CH_2$), 123.8 ($C_{5thz}$), 125.9 ($C_{3thz}$), 126.8 ($C_{4thz}$), 128.2 ($C_4$), 133.6 ($C_3$), 146.5 ($C_{2thz}$). HPLC (method A, $t_R$, min): 10.00. MS (ESI, *m/z*, %): 277.2 ($[M+H]^+$, 100). Elemental analysis calculated for $C_{17}H_{24}N_2S{\cdot}2HCl{\cdot}3/2H_2O$: %C 51.06, %H 7.77, %N 7.44; experimental: %C 50.44, %H 7.40, %N: 7.04.

**(1*S*,5*R*,6*S*)-3-{[(Cyclopropylmethyl)amino]methyl}-5-methyl-6-(1,3-oxazol-4-yl) cyclohex-3-en-1-amine, 32.** Following general procedure D using **84** (120 mg, 0.41 mmol), compound **32** was obtained as a brown oil (65 mg, 60%). Chromatography: DCM to DCM/EtOH/$NH_3$ 9:1:0.1.

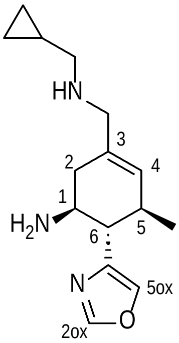


$R_f$: 0.40 (DCM/MeOH/$NH_3$ 9:1:0.1). $[\alpha]^D_{20}$ = 4.0 (c = 1.0, $CHCl_3$). IR (ATR): ν 3279 ($NH_2$), 1513 (C-N). $^1$H-NMR ($CDCl_3$): δ 0.07-0.16 (m, 2H, $CH_{2cpr}$), 0.43-0.52 (m, 2H, $CH_{2cpr}$), 0.86 (d, *J* = 7.0, 3H, $CH_3$), 0.91-1.04 (m, 1H, $CH_{cpr}$), 1.60 (br s, 3H, NH, $NH_2$), 1.85-2.00 (m, 1H, $H_2$), 2.12 (t, *J* = 10.3, 1H, $H_6$), 2.29-2.41 (m, 1H, $H_2$), 2.44 (d, *J* = 6.8,

2H, NHC$\underline{H}_2$CH) 2.49-2.59 (m, 1H, $H_5$), 3.20 (s, 2H, NHC$\underline{H}_2$), 3.29 (td, $J$ = 10.6, 5.3, $H_1$), 5.45 (s, 1H, $H_4$), 7.55 (d, $J$ = 1.0, 1H, $H_{5ox}$), 7.89 (d, $J$ = 1.0, 1H, $H_{2ox}$). $^{13}$C-RMN ($CDCl_3$): δ 3.5 ($CH_{2cpr}$), 3.6 ($CH_{2cpr}$), 11.3 ($CH_{cpr}$), 20.1 ($CH_3$), 35.1 ($C_5$), 36.5 ($C_2$), 48.7 ($C_6$), 49.9 ($C_1$), 54.4 (NH$\underline{C}H_2$CH), 55.2 ($NHCH_2$), 128.0 ($C_4$), 133.6 ($C_3$), 136.7 ($C_{5ox}$), 140.4 ($C_{4ox}$), 151.7 ($C_{2ox}$). HPLC (method A, $t_R$, min): 3.82. MS (ESI, *m/z*, %): 262.2 ([M+H]$^+$, 100). Elemental analysis calculated for $C_{15}H_{23}N_3O$ ·3HCl·1/2$H_2O$: %C 47.44, %H 7.17, %N 11.07; experimental: %C 47.71, %H 7.05, %N: 11.02.

**(1'*R*,2'*S*,6'*R*)-2'-Amino-4'-{[(cyclopropylmethyl)amino]methyl}-6'-methyl-1',2',3',6'-tetrahydro[1,1'-biphenyl]-4-ol, 33.** To a solution of **1** (68 mg, 0.23 mmol) in anhydrous DCM (10 mL/mmol), 1 M $BBr_3$ in DCM (1.15 mL, 1.15 mmol) was added at 0 ºC and the reaction mixture was stirred at this temperature for 2 h. Then, water was added and the mixture was stirred for 20 min. Next, the aqueous phase was washed with EtOAc, basified with a sat. $NaHCO_3$ solution until pH 9 and extracted with EtOAc (x2). The combined organic extracts were dried over $Na_2SO_4$ and filtered, and the solvent was evaporated under reduced pressure to afford **33** as a yellow oil (43 mg, 65%).

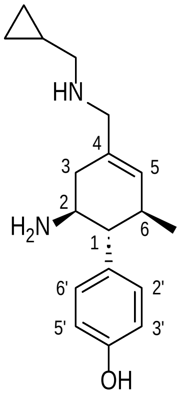


$R_f$: 0.22 (DCM/MeOH/$NH_3$ 9:1:0.1). $[\alpha]^D_{20}$ = -14.9 (c = 0.62, MeOH). $^1$H-NMR (MeOH-*d4*): δ 0.16-0.21 (m, 2H, $CH_{2cpr}$), 0.50-0.56 (m, 2H, $CH_{2cpr}$), 0.81 (d, $J$ = 7.0, 3H, $CH_3$), 0.91-1.03 (m, 1H, $CH_{cpr}$), 1.98-2.05 (m, 2H, $H_1$, $H_3$), 2.33-2.41 (m, 2H, $H_3$, $H_6$), 2.45 (d, $J$ = 6.9, 2H, NHC$\underline{H}_2$CH), 3.15 (td, $J$ = 10.6, 5.2, 1H, $H_2$), 3.22 (s, 2H, NHC$\underline{H}_2$), 5.53 (s, 1H, $H_5$), 6.79 (d, $J$ = 8.5, 2H, $H_{3'}$, $H_{5'}$), 7.06 (d, $J$ = 8.5, 2H, $H_{2'}$, $H_{6'}$).$^{13}$C-NMR (MeOH-*d4*): δ 4.05, 4.08 ($2CH_{2cpr}$), 11.2 ($CH_{cpr}$), 20.3 ($CH_3$), 36.7 ($C_3$), 39.2 ($C_6$), 52.7 ($C_2$), 54.6 (NH$\underline{C}H_2$CH), 55.4 ($NHCH_2$), 57.4 ($C_{1'}$), 116.6 ($C_3$, $C_5$), 130.4 ($C_2$, $C_6$), 131.0 ($C_5$) 133.3, 133.6 ($C_{1'}$, $C_4$), 157.5 ($C_{4'}$). HPLC (method A, $t_R$, min): 13.02. MS (ESI, *m/z*, %): 287.3 ([M+H]$^+$, 100). Elemental analysis calculated for $C_{18}H_{26}N_2O$·2HCl·2$H_2O$: %C 54.68, %H 8.16, %N 7.09; experimental: %C 55.08, %H 8.23, %N 6.69.

## 2.7. Synthesis of final compounds 85-89 (Scheme 4)

**1-[(1*R*,2*S*,6*R*)-4'-Fluoro-6-methyl-2-nitro-1,2,3,6-tetrahydro[1,1'-biphenyl]-4-yl]-*N*-methylmethanamine, 90.** Following general procedure C using **62** (750 mg, 2.85 mmol) and a 2 M solution of methylamine in methanol (2.85 mL, 5.70 mmol), compound **90** was obtained as a yellow oil (295 mg, 58%). Chromatography: DCM to DCM/MeOH/$NH_3$ 9:1:0.1.

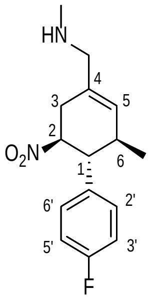


$R_f$: 0.30 (DCM/MeOH/$NH_3$ 9.5:0.5:0.1). $[\alpha]^D_{20}$ = -12.0 (c = 1.0, $CHCl_3$). IR (ATR): ν 1550 ($NO_2$), 1511 (C-N). $^1$H-RMN ($CDCl_3$): δ 0.89 (d, *J* = 7.0, 3H, $CH_3$), 2.42 (s, 3H, NHC$\underline{H}_3$), 2.44-2.56 (m, 1H, $H_6$), 2.71-2.80 (m, 2H, $2H_3$), 2.87 (t, *J* = 10.9, 1H, $H_1$), 3.18 (s, 2H, NHC$\underline{H}_2$), 4.96 (ddd, *J* = 11.6, 9.7, 6.6, 1H, $H_2$), 5.55 (s, 1H, $H_5$), 6.99 (t, *J* = 8.6, 2H, $H_{3'}$, $H_{5'}$), 7.12-7.16 (m, 2H, $H_{2'}$, $H_{6'}$). $^{13}$C-RMN ($CDCl_3$): δ 19.6 ($CH_3$), 33.2 ($C_3$), 36.1 ($NHCH_3$), 37.5 ($C_6$), 51.9 ($C_1$), 57.0 ($NHCH_2$), 88.6 ($C_2$), 115.7 (d, *J* = 21.4, $C_{3'}$, $C_{5'}$), 128.4 ($C_5$), 129.6 (d, *J* = 8.1, $C_{2'}$, $C_{6'}$), 131.8 ($C_4$), 134.4 (d, *J* = 3.3, $C_{1'}$), 162.3 (d, *J* = 246.0, $C_{4'}$). HPLC (method A, $t_R$, min): 12.75. MS (ESI, *m/z*, %): 279.1 ($[M+H]^+$, 100).

**[(1*R*,2*S*,6*R*)-4'-Fluoro-6-methyl-2-nitro-1,2,3,6-tetrahydro[1,1'-biphenyl]-4-yl] methanol, 91.** To a solution of **62** (806 mg, 3.06 mmol) in methanol (30 mL) at 0 ºC, $NaBH_4$ (232 mg, 6.12 mmol) was added portionwise and the mixture was stirred at rt for 2 h. A sat. $NH_4Cl$ solution was added until acid pH and the organic solvent was evaporated under reduced pressure. The residue was dissolved in EtOAc (30 mL), pyridine (2.2 mL, 30.6 mmol) was added and the mixture was stirred at rt for 2 days. After this time, the reaction mixture was washed with water (x2) and the organic layers were dried over $Na_2SO_4$, filtered and evaporated under reduced pressure. The crude was purified by flash chromatography (hexane to hexane/EtOAc 7:3) to afford compound **91** as a brown oil (605 mg, 75%).

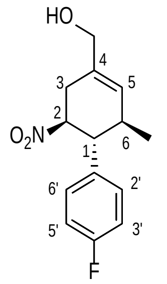


$R_f$: 0.50 (hexane/EtOAc 1:1). $[\alpha]^D_{20}$ = -15.0 (c = 1.0, MeOH). IR (ATR): ν 3390 (OH), 1547 ($NO_2$), 1510 (C-N). $^1$H-RMN ($CDCl_3$): δ 0.90 (d, *J* = 7.0, 3H, $CH_3$), 1.58 (br s, 1H, OH), 2.43-2.54 (m, 1H, $H_6$), 2.75-2.84 (m, 2H, 2$H_3$), 2.89 (t, *J* = 11.0, 1H, $H_1$), 4.12 (AB system, *J* = 13.2, 2H, C$\underline{H}_2$OH), 4.98 (ddd, *J* = 11.7, 10.0, 6.3, 1H, $H_2$), 5.66 (s, 1H, $H_5$), 6.96-7.06 (m, 2H, $H_{3'}$, $H_{5'}$), 7.13-7.21 (m, 2H, $H_{2'}$, $H_{6'}$). $^{13}$C-RMN ($CDCl_3$): δ 19.4 ($CH_3$), 31.9 ($C_3$), 37.4 ($C_6$), 51.8 ($C_1$), 66.1 ($CH_2OH$), 88.3 ($C_2$), 115.9 (d, *J* = 21.5, $C_{3'}$, $C_{5'}$), 128.4 ($C_5$), 129.6 (d, *J* = 8.1, $C_{2'}$, $C_{6'}$), 132.8 ($C_4$), 134.3 (d, *J* = 3.3, $C_{1'}$), 162.3 (d, *J* = 246.2, $C_{4'}$). HPLC (method B, $t_R$, min): 14.82. MS (ESI, *m/z*, %): 264.1 ($[M-H]^+$, 100).

**2-{[(1*R*,2*S*,6*R*)-4'-Fluoro-6-methyl-2-nitro-1,2,3,6-tetrahydro[1,1'-biphenyl]-4-yl] methyl}-1H-isoindole-1,3(2*H*)-dione, 92.** To a solution of **91** (330 mg, 1.24 mmol), phthalimide (293 mg, 1.98 mmol) and triphenylphosphine (490 mg, 1.86 mmol) in anhydrous THF (12 mL) at 0 ºC, a solution of diethyl azodicarboxylate (40% in toluene, 0.88 mL, 1.98 mmol) was added dropwise and the mixture was stirred at rt for 4 h. Then, the solvent was evaporated under reduced pressure and the crude was purified by flash chromatography (hexane to hexane/EtOAc 7:3) to afford compound **92** as a white solid (314 mg, 64%).

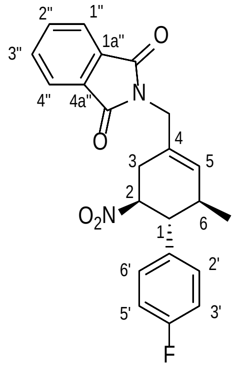


Mp: 195-202 ºC. $R_f$: 0.40 (hexane/EtOAc 7:3). $[\alpha]^D_{20}$ = 2.0 (c = 1.0, $CHCl_3$). IR (ATR): ν 1709 (CO), 1548 ($NO_2$), 1510 (C-N). $^1$H-RMN ($CDCl_3$): δ 0.87 (d, *J* = 7.0, 3H, $CH_3$), 2.39-2.54 (m, 1H, $H_6$), 2.67-2.81 (m, 2H, 2$H_3$), 2.86 (dd, *J* = 11.4, 10.7, 1H, $H_1$), 4.28 (s, 2H, $CH_2N$), 4.94 (ddd, *J* = 11.7, 10.3, 6.0, 1H, $H_2$), 5.66 (s, 1H, $H_5$), 6.91-7.03 (m, 2H,

$H_{3'}$, $H_{5'}$), 7.07-7.16 (m, 2H, $H_{2'}$, $H_{6'}$), 7.71-7.81 (m, 2H, $H_{2''}$, $H_{3''}$), 7.83-7.92 (m, 2H, $H_{1''}$, $H_{4''}$). $^{13}$C-RMN ($CDCl_3$): δ 19.2 ($CH_3$), 32.7 ($C_3$), 37.4 ($C_6$), 42.5 ($CH_2N$), 51.4 ($C_1$), 88.1 ($C_2$), 115.9 (d, $J$ = 21.5, $C_{3'}$, $C_{5'}$), 123.7 ($C_{1''}$), 123.8 ($C_{4''}$), 127.9 ($C_4$), 129.6 (d, $J$ = 8.1, $C_{2'}$, $C_{6'}$), 130.6 ($C_5$), 132.0 ($C_{1a''}$), 132.8 ($C_{4a''}$), 134.1 (d, $J$ = 3.3, $C_{1'}$), 134.4 ($C_{2''}$), 134.5 ($C_{3''}$), 162.3 (d, $J$ = 246.1, $C_{4'}$), 168.1 (CO), 168.2 (CO).

**1-[(1*R*,2*S*,6*R*)-4'-Fluoro-6-methyl-2-nitro-1,2,3,6-tetrahydro[1,1'-biphenyl]-4-yl] methanamine, 93.** A solution of **92** (295 mg, 0.75 mmol) and hydrazine monohydrate (65% solution, 0.11 mL, 1.50 mmol) in ethanol (3 mL) was stirred at reflux for 2 h. Then, the reaction mixture was filtered, the solvent was evaporated under reduced pressure and the crude was purified by flash chromatography (DCM to DCM/MeOH/$NH_3$ 9:1:0.1) to afford compound **93** as a colorless oil (141 mg, 72%).

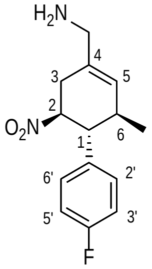


$R_f$: 0.40 (DCM/MeOH/$NH_3$ 8:2:0.1). $[\alpha]^D_{20}$ = -11.0 (c = 1.0, $CHCl_3$). IR (ATR): ν 1550 ($NO_2$), 1511 (C-N). $^1$H-RMN ($CDCl_3$): δ 0.90 (d, $J$ = 6.8, 3H, $CH_3$), 2.04 (br s, 2H, $NH_2$), 2.42-2.58 (m, 1H, $H_6$), 2.65-2.92 (m, 3H, $H_1$, 2$H_{3,}$), 3.31 (AB system, $J$ = 14.9, 2H, C$\underline{H}_2$$NH_2$), 4.91-5.06 (m, 1H, $H_2$), 5.57 (s, 1H, $H_5$), 6.94-7.06 (m, 2H, $H_{3'}$, $H_{5'}$), 7.11-7.23 (m, 2H, $H_{2'}$, $H_{6'}$). $^{13}$C-RMN ($CDCl_3$): δ 19.6 ($CH_3$), 33.0 ($C_3$), 37.5 ($C_6$), 47.0 ($CH_2NH_2$), 51.9 ($C_1$), 88.5 ($C_2$), 115.8 (d, $J$ = 21.3, $C_{3'}$, $C_{5'}$), 127.0 ($C_5$), 129.6 (d, $J$ = 8.1, $C_{2'}$, $C_{6'}$), 133.9 ($C_4$), 134.4 (d, $J$ = 3.3, $C_{1'}$), 162.3 (d, $J$ = 246.2, $C_{4'}$). HPLC (method A, $t_R$, min): 13.61. MS (ESI, *m/z*, %): 265.1 ($[M+H]^+$, 100).

***N*-{[(1*R*,2*S*,6*R*)-2-Amino-4'-fluoro-6-methyl-1,2,3,6-tetrahydro[1,1'-biphenyl]-4-yl] methyl}acetamide, 94.** To a solution of **93** (204 mg, 0.77 mmol) in a 3:1 mixture of DCM:pyridine (6 mL) at 0 ºC, acetic anhydride (87 µL, 0.93 mmol) was added dropwise and the mixture was stirred at 0 ºC for 1 h and overnight at rt. Then, the residue was suspended in a sat. $NaHCO_3$ solution and extracted with EtOAc (x3). The combined organic layers were washed with brine, dried over $Na_2SO_4$, filtered and concentrated

under reduced pressure. The crude was purified by flash chromatography (DCM to DCM/MeOH/$NH_3$ 9:1:0.1) to afford compound **94** as a colorless oil (200 mg, 85%).

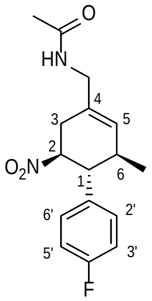


$R_f$: 0.60 (DCM/MeOH/$NH_3$ 9:1:0.1). $[\alpha]^D{}_{20}$ = -30.0 (c = 1.0, $CHCl_3$). IR (ATR): ν 3291 ($NH_2$, NH), 1655 (CO), 1509 (C-N), 1220 (COC). $^1$H-RMN ($CDCl_3$): δ 0.83 (d, *J* = 7.0 Hz, 3H, $CH_3$), 1.95 (s, 3H, $COCH_3$), 2.33-2.49 (m, 1H, $H_6$), 2.53-2.73 (m, 2H, $2H_3$), 2.81 (t, *J* = 11.1, 1H, $H_1$), 3.79 (d, *J* = 5.9, 2H, C$\underline{H}_2$NH), 4.92 (ddd, *J* = 11.6, 10.4, 6.0, 1H, $H_2$), 5.48 (s, 1H, $H_5$), 6.26 (t, *J* = 5.8, 1H, NH), 6.89-6.99 (m, 2H, $H_{3'}$, $H_{5'}$), 7.03-7.19 (m, 2H, $H_{2'}$, $H_{6'}$). $^{13}$C-RMN ($CDCl_3$): δ 19.2 ($CH_3$), 23.1 (CO$\underline{C}H_3$), 32.5 ($C_3$), 37.2 ($C_6$), 44.1 ($CH_2$NH), 51.5 ($C_1$), 88.1 ($C_2$), 115.7 (d, *J* = 21.4, $C_{3'}$, $C_{5'}$), 128.6 ($C_5$), 129.5 (d, *J* = 8.1, $C_{2'}$, $C_{6'}$), 130.0 ($C_4$), 134.1 (d, *J* = 3.2, $C_{1'}$), 162.1 (d, *J* = 246.1, $C_{4'}$), 170.5 (CO). HPLC (method A, $t_R$, min): 11.81. MS (ESI, *m/z*, %): 277.1 ($[M+H]^+$, 100).

**(1*R*,2*S*,6*R*)-4'-Fluoro-6-methyl-4-[(methylamino)methyl]-1,2,3,6-tetrahydro[1,1'-biphenyl]-2-amine, 85.** Following general procedure D using **90** (295 mg, 1.06 mmol), compound **85** was obtained as a colorless oil (151 mg, 82%). Chromatography: DCM to DCM/EtOH/$NH_3$ 8:2:0.1.

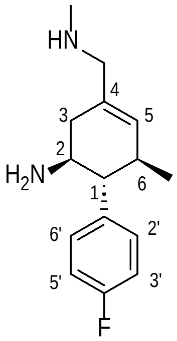


$R_f$: 0.12 (DCM/MeOH/$NH_3$ 8:2:0.1). $[\alpha]^D{}_{20}$ = -32.0 (c = 1.0, $CHCl_3$). IR (ATR): ν 3264 (NH), 1509 (C-N). $^1$H-NMR ($CDCl_3$): δ 0.78 (d, *J* = 7.0, 3H, $CH_3$), 1.91-2.18 (m, 6H, $H_1$, $2H_3$, NH, $NH_2$), 2.27-2.38 (m, 1H, $H_6$), 2.42 (s, 3H, NHC$\underline{H}_3$), 3.10-3.22 (m, 3H, $H_2$, NHC$\underline{H}_2$), 5.47 (s, 1H, $H_5$), 7.00 (t, *J* = 8.7, 2H, $H_{3'}$, $H_{5'}$), 7.15 (dd, *J* = 8.7, 5.5, 2H, $H_{2'}$, $H_{6'}$). $^{13}$C-NMR ($CDCl_3$): δ 20.0 ($CH_3$), 33.6 ($NHCH_3$), 36.7 ($C_3$), 38.1 ($C_6$), 51.8

($C_2$), 57.15 ($NHCH_2$), 57.19 ($C_1$), 115.6 (d, *J* = 21.1, $C_{3'}$, $C_{5'}$), 129.3 ($C_5$), 129.9 (d, *J* = 7.8, $C_{2'}$, $C_{6'}$), 132.8 ($C_4$), 138.2 (d, *J* = 3.2, $C_{1'}$), 161.8 (d, *J* = 246.0, $C_{4'}$). HPLC (method A, $t_R$, min): 10.48. MS (ESI, *m/z*, %): 249.2 ($[M+H]^+$, 100). Elemental analysis calculated for $C_{15}H_{21}FN_2{\cdot}2HCl{\cdot}5/2H_2O$: %C 51.73, %H 7.52, %N 8.04; experimental: %C 51.97, %H 7.16, %N 7.75.

**(1*R*,2*S*,6*R*)-4-(Aminomethyl)-4'-fluoro-6-methyl-1,2,3,6-tetrahydro[1,1'-biphenyl]-2-amine, 86.** Following general procedure D using **93** (80 mg, 0.30 mmol), compound **86** was obtained as a colorless oil (36 mg, 51%). Chromatography: DCM to DCM/MeOH/$NH_3$ 8:2:0.1.

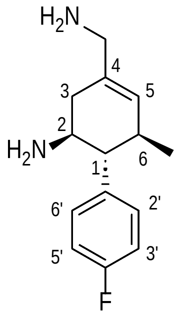


$R_f$: 0.10 (DCM/MeOH/$NH_3$ 9:1:0.1). $[\alpha]^D_{20}$ = -39.0 (c = 1.0, $CHCl_3$). IR (ATR): ν 3353 ($NH_2$), 1508 (C-N). $^1$H-NMR ($CDCl_3$): δ 0.80 (d, *J* = 6.9, 3H, $CH_3$), 1.29 (br s, 4H, 2$NH_2$), 1.90-2.00 (m, 1H, $H_3$), 2.04 (t, *J* = 10.5, 1H, $H_1$), 2.25-2.41 (m, 2H, $H_3$, $H_6$), 3.11-3.26 (m, 3H, $H_2$, $NH_2C\underline{H}_2$), 5.44 (s, 1H, $H_5$), 6.95-7.08 (m, 2H, $H_{3'}$, $H_{5'}$) 7.09-7.21 (m, 2H, $H_{2'}$, $H_{6'}$). $^{13}$C-NMR ($CDCl_3$): δ 20.1 ($CH_3$), 36.7 ($C_3$), 38.1 ($C_6$), 47.7 ($NH_2CH_2$), 51.9 ($C_2$), 57.6 ($C_1$), 115.6 (d, *J* = 21.0, $C_{3'}$, $C_{5'}$), 126.3 ($C_5$), 129.9 (d, *J* = 7.7, $C_{2'}$, $C_{6'}$), 136.7 ($C_4$), 138.4 (d, *J* = 3.3, $C_{1'}$), 161.8 (d, *J* = 244.3, $C_{4'}$). HPLC (method A, $t_R$, min): 9.35. MS (ESI, *m/z*, %): 235.1 ($[M+H]^+$, 100). Elemental analysis calculated for $C_{14}H_{19}FN_2{\cdot}2HCl$: %C 54.73, %H 6.89, %N 9.12; experimental: %C 54.39, %H 6.56, %N 8.72.

***N*-{[(1*R*,2*S*,6*R*)-2-Amino-4'-fluoro-6-methyl-1,2,3,6-tetrahydro[1,1'-biphenyl]-4-yl] methyl}acetamide, 87.** Following general procedure D using **94** (260 mg, 0.85 mmol), compound **87** was obtained as a colorless oil (214 mg, 91%). Chromatography: DCM to DCM/MeOH/$NH_3$ 9:1:0.1.

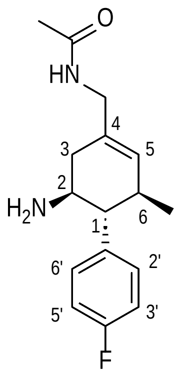


$R_f$: 0.60 (DCM/MeOH/$NH_3$ 9:1:0.1). $[\alpha]^D_{20}$ = -30.0 (c = 1.0, $CHCl_3$). IR (ATR): ν 3291 ($NH_2$, NH), 1655 (CO), 1509 (C-N), 1220 (COC). $^1$H-NMR ($CDCl_3$): δ 0.75 (d, *J* = 7.0, 3H, $CH_3$), 1.62 (br s, 2H, $NH_2$), 1.83-1.95 (m, 1H, $H_3$), 1.96-2.06 (m, 4H, $COCH_3$, $H_1$), 2.22-2.36 (m, 2H, $H_3$, $H_6$), 3.13 (td, *J* = 10.5, 5.3, 1H, $H_2$), 3.68-3.85 (m, 2H, $NHC\underline{H}_2$), 5.40 (s, 1H, $H_5$), 5.95-6.08 (m, 1H, CONH), 6.91-7.02 (m, 2H, $H_{3'}$, $H_{5'}$), 7.02-7.17 (m, 2H, $H_{2'}$, $H_{6'}$). $^{13}$C-NMR ($CDCl_3$): δ 19.8 ($CH_3$), 23.3 ($CO\underline{C}H_3$), 36.2 ($C_3$), 37.9 ($C_6$), 44.7 ($NHCH_2$), 51.6 ($C_2$), 57.0 ($C_1$), 115.6 (d, *J* = 21.0, $C_{3'}$, $C_{5'}$), 128.7 ($C_5$), 129.7 (d, *J* = 7.7, $C_{2'}$, $C_{6'}$), 132.1 ($C_4$), 137.9 (d, *J* = 3.3, $C_{1'}$), 161.7 (d, *J* = 244.5, $C_{4'}$), 170.3 (CO). HPLC (method A, $t_R$, min): 11.81. MS (ESI, *m/z*, %): 277.1 ($[M+H]^+$, 100). Elemental analysis calculated for $C_{16}H_{21}FN_2O \cdot HCl \cdot H_2O$: %C 58.09, %H 7.31, %N 8.47; experimental: %C 57.88, %H 6.92, %N 8.15.

**[(1*R*,2*S*,6*R*)-2-Amino-4'-fluoro-6-methyl-1,2,3,6-tetrahydro[1,1'-biphenyl]-4-yl] methanol, 88.** Following general procedure D using **91** (215 mg, 0.81 mmol), compound **88** was obtained as a colorless oil (130 mg, 68%). Chromatography: DCM to DCM/MeOH/$NH_3$ 9:1:0.1.

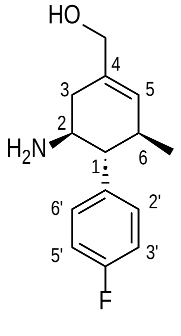


$R_f$: 0.60 (DCM/MeOH/$NH_3$ 9:1:0.1). $[\alpha]^D_{20}$ = -37.0 (c = 1.0, $CHCl_3$). IR (ATR): ν 3360 (OH), 3230 ($NH_2$), 1509 (C-N). $^1$H-NMR ($CDCl_3$): δ 0.80 (d, *J* = 7.0, 3H, $CH_3$), 1.97-2.08 (m, 1H, $H_3$), 2.12 (t, *J* = 10.5, 1H, $H_1$), 2.37 (br s, 4H, $H_6$, $NH_2$, OH), 2.46 (dd, *J* = 17.1, 5.3, 1H, $H_3$), 3.19 (td, *J* = 10.5, 5.3, 1H, $H_2$), 4.03 (s, 2H, $C\underline{H}_2OH$), 5.54 (s, 1H, $H_5$),

6.98-7.11 (m, 2H, $H_{3'}$, $H_{5'}$), 7.09-7.22 (m, 2H, $H_{2'}$, $H_{6'}$). $^{13}$C-NMR ($CDCl_3$): δ 19.9 ($CH_3$), 35.1 ($C_3$), 38.0 ($C_6$), 51.9 ($C_2$), 56.7 ($C_1$), 66.5 ($CH_2OH$), 115.7 (d, *J* = 21.1, $C_{3'}$, $C_{5'}$), 128.4 ($C_5$), 129.9 (d, *J* = 7.8, $C_{2'}$, $C_{6'}$), 135.1 ($C_4$), 137.8 (d, *J* = 3.2, $C_{1'}$), 161.9 (d, *J* = 244.8, $C_{4'}$). HPLC (method A, $t_R$, min): 12.31. MS (ESI, *m/z*, %): 236.1 ($[M+H]^+$, 100). Elemental analysis calculated for $C_{14}H_{18}FNO{\cdot}HCl{\cdot}1/2H_2O$: %C 59.89, %H 7.18, %N 4.99; experimental: %C 59.51, %H 6.78, %N 4.95.

***tert*-Butyl [(1*R*,2*S*,6*R*)-4'-fluoro-4-(hydroxymethyl)-6-methyl-1,2,3,6-tetrahydro [1,1'-biphenyl]-2-yl]carbamate, 95.** Following general procedure G using **88** (120 mg, 0.51 mmol), triethylamine (92 µL, 0.66 mmol) and di-*tert*-butyl dicarbonate (223 mg, 1.02 mmol), compound **95** was obtained as a colorless oil (110 mg, 64%). Chromatography: hexane to hexane/EtOAc 7:3.

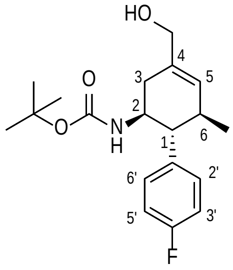

$R_f$: 0.40 (hexane/EtOAc 7:3). $[\alpha]^D_{20}$ = -71.0 (c = 1.0, $CHCl_3$). IR (ATR): ν 3354 (OH), 1686 (CO), 1510 (C-N), 1223 (COC). $^1$H-NMR ($CDCl_3$): δ 0.84 (d, *J* = 7.0, 3H, $CH_3$), 1.25 (s, 9H, $3CH_3$), 1.64 (br s, 1H, OH), 2.01-2.10 (m, 1H, $H_3$), 2.25 (t, *J* = 10.7, 1H, $H_1$), 2.40-2.50 (m, 1H, $H_6$), 2.56 (dd, *J* = 16.3, 5.3, 1H, $H_3$), 3.93-4.02 (m, 1H, $H_2$), 4.06 (AB system, *J* = 13.3, 2H, C$\underline{H}_2$OH), 4.15-4.25 (m, 1H, NH), 5.57 (s, 1H, $H_5$), 6.98-7.02 (m, 2H, $H_{3'}$, $H_{5'}$), 7.11-7.18 (m, 2H, $H_{2'}$, $H_{6'}$). $^{13}$C-NMR ($CDCl_3$): δ 20.0 ($CH_3$), 28.3 ($3CH_3$), 34.1 ($C_3$), 38.6 ($C_6$), 51.1 ($C_2$), 54.5 ($C_1$), 66.6 ($CH_2OH$), 79.3 ($\underline{C}(CH_3)_3$), 115.2 (d, *J* = 21.1, $C_{3'}$, $C_{5'}$), 128.2 ($C_5$), 130.0 (d, *J* = 7.8, $C_{2'}$, $C_{6'}$), 135.0 ($C_4$), 137.2 (d, *J* = 3.2, $C_{1'}$), 155.3 (CO), 161.9 (d, *J* = 244.8, $C_{4'}$).

***tert*-Butyl [(1*R*,2*S*,6*R*)-4'-fluoro-4-(methoxymethyl)-6-methyl-1,2,3,6-tetrahydro [1,1'-biphenyl]-2-yl]methylcarbamate, 96.** To a solution of cesium hydroxide monohydrate (46 mg, 0.28 mmol) in anhydrous DMF (2.2 mL/mmol) with activated 4 Å molecular sieves at rt, **95** was added (77 mg, 0.23 mmol) and the mixture was stirred for 30 min. Then, iodomethane (43 µL, 0.69 mmol) was added and the reaction was stirred for 4 h at rt. After this time, the mixture was filtered through celite, water was added to

the filtrate, and it was extracted with EtOAc (x2). The organic layers were washed with brine, dried over $Na_2SO_4$, filtered and evaporated under reduced pressure. The crude was purified by flash chromatography (hexane to hexane/EtOAc 7:3) to afford compound **96** as a colorless oil (40 mg, 50%).

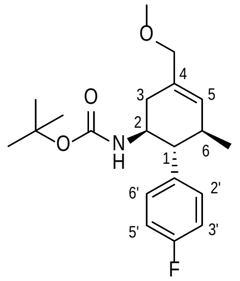


$R_f$: 0.50 (hexane/EtOAc 7:3). $[\alpha]^D_{20}$ = -22.0 (c = 1.0, $CHCl_3$). IR (ATR): ν 3344 (NH), 1689 (CO), 1510 (C-N), 1223 (COC). $^1$H-NMR ($CDCl_3$): δ 0.83 (d, *J* = 7.0, 3H, $CH_3$), 1.24 (s, 9H, $3CH_3$), 1.95-2.09 (m, 1H, $H_3$), 2.24 (t, *J* = 10.6, 1H, $H_1$), 2.38-2.48 (m, 1H, $H_6$), 2.53 (dd, *J* = 17.1, 5.6, 1H, $H_3$), 3.32 (s, 3H, $OCH_3$), 3.82 (s, 2H, C$\underline{H}_2$$OCH_3$), 3.90-4.09 (m, 1H, $H_2$), 4.12-4.24 (m, 1H, NH), 5.56 (s, 1H, $H_5$), 6.91-7.03 (m, 2H, $H_{3'}$, $H_{5'}$), 7.08-7.13 (m, 2H, $H_{2'}$, $H_{6'}$). $^{13}$C-NMR ($CDCl_3$): δ 20.0 ($CH_3$), 28.3 ($3CH_3$), 34.4 ($C_3$), 38.6 ($C_6$), 51.1 ($C_2$), 54.5 ($C_1$), 58.0 ($OCH_3$), 76.3 ($\underline{C}H_2OCH_3$), 79.1 ($\underline{C}(CH_3)_3$), 115.1 (d, *J* = 21.2, $C_{3'}$, $C_{5'}$), 130.0 (d, *J* = 7.8, $C_{2'}$, $C_{6'}$), 130.1 ($C_5$), 132.4 ($C_4$), 137.3 (d, *J* = 3.2, $C_{1'}$), 155.2 (CO), 161.8 (d, *J* = 244.0, $C_{4'}$). HPLC (method B, $t_R$, min): 10.67. MS (ESI, *m/z*, %): 250.1 ($[M\text{-}Boc]^+$, 100).

**(1*R*,2*S*,6*R*)-4'-Fluoro-4-(methoxymethyl)-6-methyl-1,2,3,6-tetrahydro[1,1'-biphenyl]-2-amine, 89.** Following general procedure I using **96** (29 mg, 0.08 mmol), compound **89** was obtained as a colorless oil (13 mg, 63%).

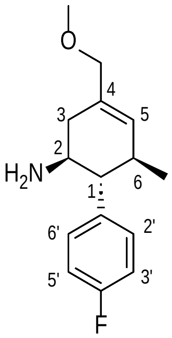


$R_f$: 0.54 (DCM/MeOH/$NH_3$ 9:1:0.1). $[\alpha]^D_{20}$ = -77.0 (c = 1.00, $CHCl_3$). IR (ATR): ν 1509 (C-N), 1221 (COC). $^1$H-NMR ($CDCl_3$): δ 0.81 (d, *J* = 7.0, 3H, $CH_3$), 1.70 (br s, 2H, $NH_2$), 1.86-2.03 (m, 1H, $H_3$), 2.11 (t, *J* = 10.4, 1H, $H_1$), 2.28-2.52 (m, 2H, $H_3$, $H_6$), 3.20 (td, *J* = 10.5, 5.2, 1H, $H_2$), 3.33 (s, 3H, $OCH_3$), 3.84 (s, 2H, C$\underline{H}_2$$OCH_3$), 5.57 (s, 1H, $H_5$), 6.88-

7.09 (m, 2H, $H_{3'}$, $H_{5'}$), 7.09-7.21 (m, 2H, $H_{2'}$, $H_{6'}$). $^{13}$C-NMR ($CDCl_3$): δ 19.9 ($CH_3$), 35.6 ($C_3$), 38.1 ($C_6$), 51.8 ($C_2$), 57.0 ($C_1$), 58.0 ($OCH_3$), 76.4 (<u>C</u>$H_2OCH_3$), 115.7 (d, *J* = 21.0, $C_{3'}$, $C_{5'}$), 129.9 (d, *J* = 7.7, $C_{2'}$, $C_{6'}$), 130.4 ($C_5$), 132.3 ($C_4$), 138.1 (d, *J* = 2.9, $C_{1'}$), 161.9 (d, *J* = 244.5, $C_{4'}$). HPLC (method A, $t_R$, min): 11.56. MS (ESI, *m/z*, %): 250.1 ($[M+H]^+$, 100). Elemental analysis calculated for $C_{15}H_{20}FNO·HCl·H_2O$: %C 59.30, %H 7.63, %N 4.61; experimental: %C 58.96, %H 7.25, %N 4.69.

## 2.8. Synthesis of final compound 97 (Scheme 5)

***tert*-Butyl ({(1*R*,2*S*,6*R*)-2-[(*tert*-butoxycarbonyl)amino]-4'-fluoro-6-methyl-1,2,3,6-tetrahydro[1,1'-biphenyl]-4-yl}methyl)(cyclopropylmethyl)carbamate, 99.** Following general procedure G using **25** (137 mg, 0.32 mmol), triethylamine (0.2 mL, 0.83 mmol) and di-*tert*-butyl dicarbonate (280 mg, 1.28 mmol), compound **99** was obtained as a colorless oil (152 mg, 66%). Chromatography: hexane to hexane/EtOAc 7:3.

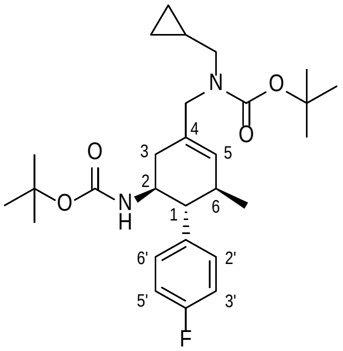


$R_f$: 0.70 (hexane/EtOAc 7:3). $[\alpha]^D{}_{20}$ = -9.0 (c = 1.0, $CHCl_3$). IR (ATR): ν 3356 (NH), 1690 (C=O), 1511 (C-N). $^1$H-NMR ($CDCl_3$, mixture of rotamers): δ 0.12-0.26 (m, 2H, $CH_{2cpr}$), 0.40-0.53 (m, 2H, $CH_{2cpr}$), 0.81 (d, *J* = 6.9, 3H, $CH_3$), 0.91-1.04 (m, 1H, $CH_{cpr}$), 1.23 (s, 9H, $3CH_3$), 1.46 (s, 9H, $3CH_3$), 1.84-2.00 (m, 1H, $H_3$), 2.20 (t, *J* = 10.8, 1H, $H_1$), 2.35-2.52 (m, 2H, $H_3$, $H_6$), 3.06 (br s, 2H, NC<u>H</u>$_2$CH), 3.73-4.06 (m, 3H, $H_2$, $NCH_2$), 4.06-4.19 (m, 1H, NH), 5.35 (s, 1H, $H_5$), 6.97 (t, *J* = 8.7, 2H, $H_{3'}$, $H_{5'}$), 7.13 (dd, *J* = 8.6, 5.5, 2H, $H_{2'}$, $H_{6'}$). $^{13}$C-NMR ($CDCl_3$): δ 3.7 ($2CH_{2cpr}$), 10.1 ($CH_{cpr}$). 20.1 ($CH_3$), 28.3 ($3CH_3$), 28.6 ($3CH_3$), 34.7 (br, $C_3$), 38.6 ($C_6$), 50.6 (N<u>C</u>$H_2$CH), 51.4 ($NCH_2$), 51.9 (br, $C_2$), 54.7 ($C_1$), 79.2 (<u>C</u>$(CH_3)_3$), 79.6 (<u>C</u>$(CH_3)_3$), 115.1 (d, *J* = 21.0, $C_{3'}$, $C_{5'}$), 128.6 ($C_5$), 130.0 (d, *J* = 7.8, $C_{2'}$, $C_{6'}$), 132.0 ($C_4$), 137.3 ($C_{1'}$), 155.2 (CO), 155.9 (CO), 161.8 (d, *J* = 244.1, $C_{4'}$). HPLC (method B, $t_R$, min): 14.51. MS (ESI, *m/z*, %): 389.2 ([M-Boc]$^+$, 100).

***tert*-Butyl [(1*R*,2*S*,6*R*)-4-{[(*tert*-butoxycarbonyl)(cyclopropylmethyl) amino]methyl} -4'-fluoro-6-methyl-1,2,3,6-tetrahydro[1,1'-biphenyl]-2-yl] methylcarbamate, 101.** Following general procedure H using **99** (125 mg, 0.26 mmol), compound **101** was obtained as a colorless oil (100 mg, 78%). Chromatography: hexane to hexane/EtOAc 7:3.

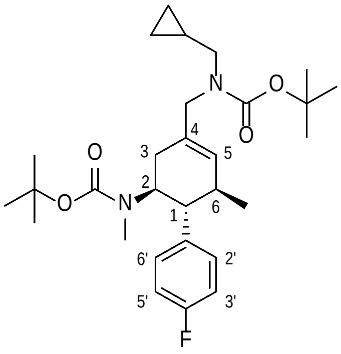


$R_f$: 0.60 (hexane/EtOAc 7:3). $[\alpha]^D_{20}$ = -25.0 (c = 1.0, $CHCl_3$). IR (ATR): ν 1690 (C=O), 1511 (C-N). $^1$H-NMR ($CDCl_3$, mixture of rotamers): δ 0.14-0.27 (m, 2H, $CH_{2cpr}$), 0.43-0.53 (m, 2H, $CH_{2cpr}$), 0.75-0.85 (m, 3H, $CH_3$), 0.94-1.03 (m, 1H, $CH_{cpr}$), 1.25 (s, 9H, $3CH_3$), 1.47 (s, 9H, $3CH_3$), 2.02-2.11 (m, 1H, $H_3$), 2.11-2.22 (m, 1H, $H_3$), 2.23-2.40 (m, 2H, $H_1$, $H_6$), 2.43 and 2.51 (s, 3H, $NCH_3$), 3.07 (br s, 2H, $NC\underline{H}_2CH$), 3.85, 4.00 (br m, 2H, $NCH_2$), 4.48-4.58 and 4.72-4.84 (m, 1H, $H_2$), 5.33 (s, 1H, $H_5$), 6.93-6.97 (m, 2H, $H_{3'}$, $H_{5'}$), 7.09-7.11 and 7.14-7.17 (m, 2H, $H_{2'}$, $H_{6'}$). $^{13}$C-NMR ($CDCl_3$): δ 3.7 and 3.8 ($2CH_{2cpr}$), 10.1 ($CH_{cpr}$). 20.1 ($CH_3$), 27.3 and 27.9 ($NCH_3$), 28.4 ($3CH_3$), 28.6 ($3CH_3$), 30.0 (br, $C_3$), 39.3 ($C_6$), 50.5 and 51.0 ($N\underline{C}H_2CH$), 51.8 ($C_1$), 52.1 (br, $NCH_2$), 53.7 and 55.1 ($C_2$), 79.2 ($\underline{C}(CH_3)_3$), 79.6 ($\underline{C}(CH_3)_3$), 114.9 (d, *J* = 21.3) and 115.2 (d, *J* = 21.1, $C_{3'}$, $C_{5'}$), 128.5 (br, $C_5$), 129.6 and 129.9 (br, $C_{2'}$, $C_{6'}$), 132.4 ($C_4$), 137.2 (br, $C_{1'}$), 155.4 (CO), 156.0 (br, CO), 161.7 (d, *J* = 243.7, $C_{4'}$). HPLC (method B, $t_R$, min): 20.56. MS (ESI, *m/z*, %): 403.3 ($[M\text{-}Boc]^+$, 100).

**(1*R*,2*S*,6*R*)-4-{[(Cyclopropylmethyl)amino]methyl}-4'-fluoro-*N*,6-dimethyl-1,2,3,6-tetrahydro[1,1'-biphenyl]-2-amine, 97.** Following general procedure I using **101** (100 mg, 0.18 mmol), compound **97** was obtained as a colorless oil (51 mg, 81%).

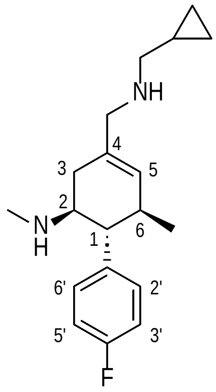

$R_f$: 0.50 (DCM/MeOH/$NH_3$ 8:2:0.1). $[\alpha]^D_{20}$ = -19.0 (c = 1.00, $CHCl_3$). IR (ATR): ν 1510 (C-N). $^1$H-NMR ($CDCl_3$): δ 0.06-0.16 (m, 2H, $CH_{2cpr}$), 0.44-0.52 (m, 2H, $CH_{2cpr}$), 0.78 (d, *J* = 6.9, 3H, $CH_3$), 0.92-1.04 (m, 1H, $CH_{cpr}$), 1.24-1.45 (br s, 2H, 2NH), 1.84-1.96 (m, 1H, $H_3$), 2.19 (t, *J* = 10.0, $H_1$), 2.26 (s, 3H, NHC$\underline{H}_3$), 2.28-2.39 (m, 1H, $H_6$), 2.42-2.52 (m, 3H, $H_3$, NHC$\underline{H}_2$CH), 2.85 (td, *J* = 10.2, 5.3, 1H, $H_2$), 3.15-3.29 (m, 2H, NHC$\underline{H}_2$), 5.47 (s, 1H, $H_5$), 7.01 (t, *J* = 8.7, 2H, $H_{3'}$, $H_{5'}$), 7.16 (dd, *J* = 8.7, 5.5, 2H, $H_{2'}$, $H_{6'}$). $^{13}$C-NMR ($CDCl_3$): δ 3.52 ($CH_{2cpr}$), 3.54 ($CH_{2cpr}$), 11.4 ($CH_{cpr}$). 20.1 ($CH_3$), 33.7 ($C_3$), 33.9 ($NHCH_3$), 38.1 ($C_6$), 54.5 (NH$\underline{C}H_2$CH), 54.9 ($C_1$), 55.5 ($NHCH_2$), 59.6 ($C_2$), 115.7 (d, *J* = 21.0, $C_{3'}$, $C_{5'}$), 128.1 ($C_5$), 129.9 (d, *J* = 7.7, $C_{2'}$, $C_{6'}$), 133.6 ($C_4$), 138.1 (d, *J* = 3.3, $C_{1'}$), 161.8 (d, *J* = 244.6, $C_{4'}$). HPLC (method A, $t_R$, min): 11.40. MS (ESI, *m/z*, %): 303.2 ($[M+H]^+$, 100). Elemental analysis calculated for $C_{19}H_{27}FN_2 \cdot 2HCl \cdot 5/2H_2O$: %C 54.28, %H 8.15, %N 6.66; experimental: %C 54.57, %H 7.76, %N 6.59.

## 3. NMR, HPLC-MS and HRMS spectra of final compounds 25 and 98

### $^1$H- and $^{13}$C-NMR ($CDCl_3$, 500 MHz) of 25 (UCM-17017):

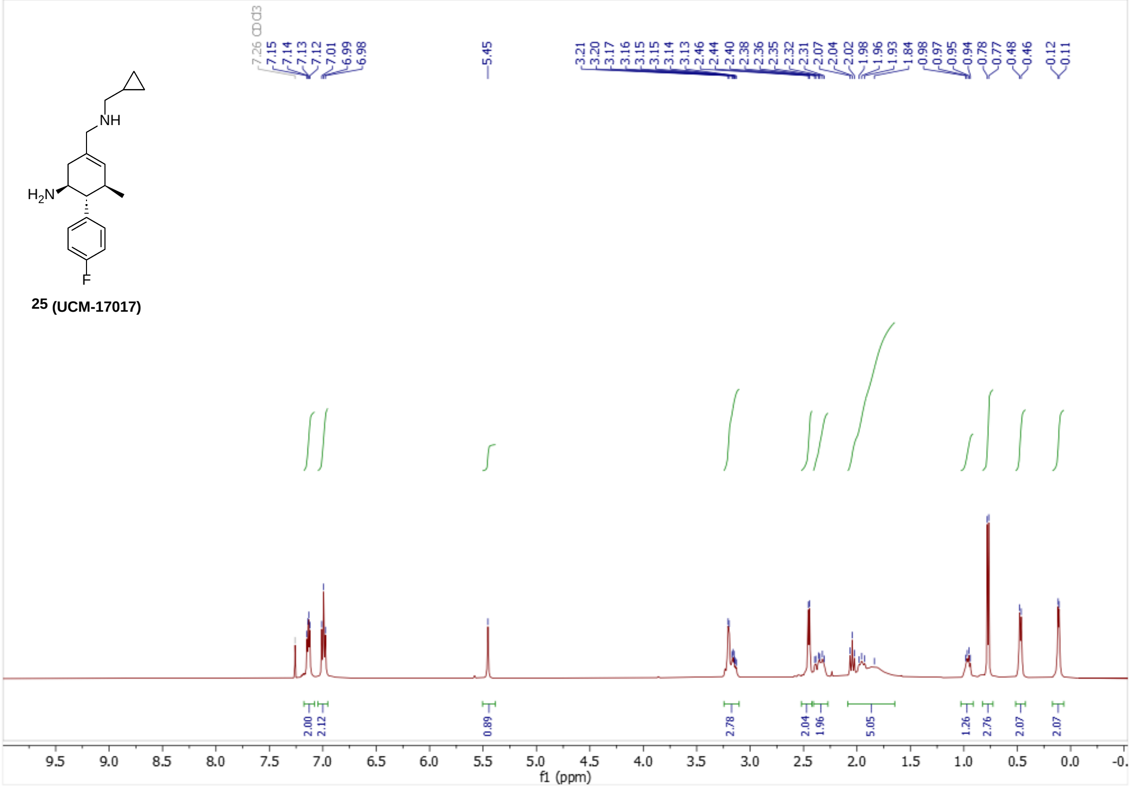


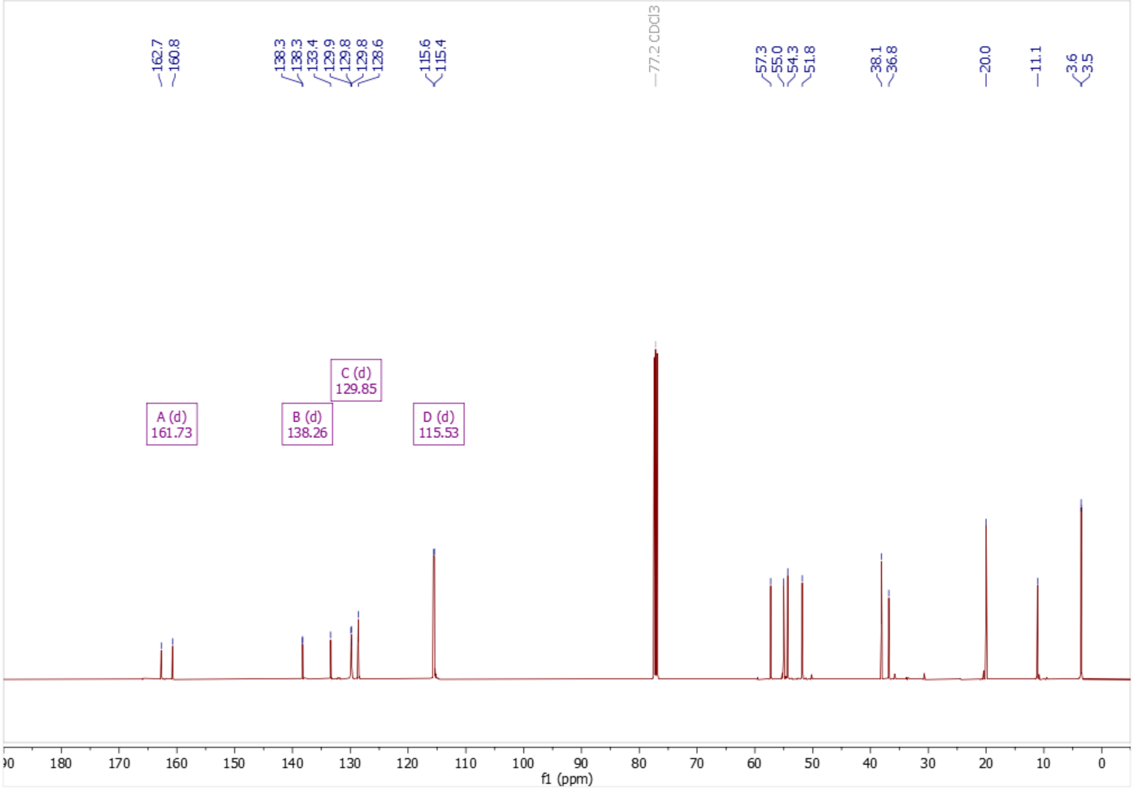

**HPLC-MS spectra of 25 (UCM-17017)**:

The HCl salt of compound **25** elutes as an early injection-front peak (3.014 min) and a retained peak (4.769 min), both exhibiting the expected molecular mass.

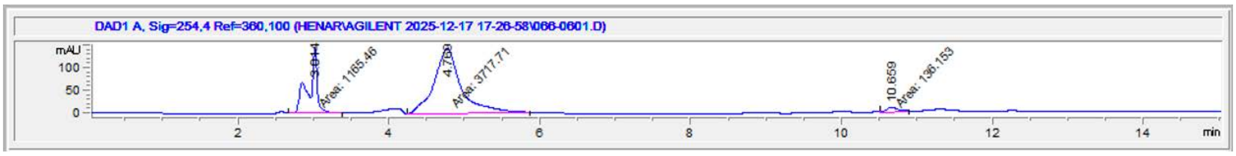


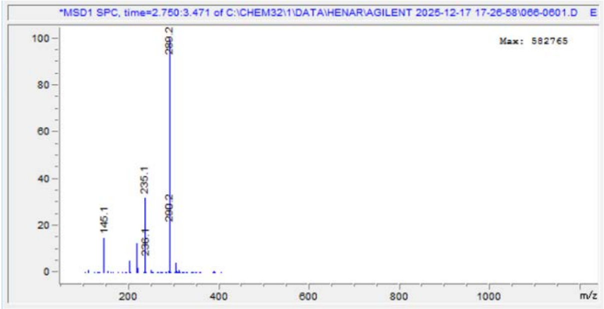


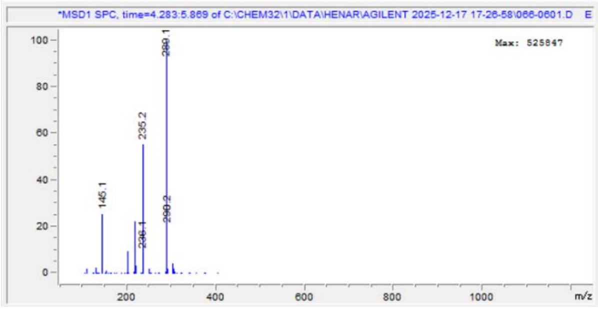


**HRMS spectra of 25 (UCM-17017)**:

**Acquisition Parameter**

| | | | | | |
|---|---|---|---|---|---|
| Source Type | ESI | Ion Polarity | Positive | Set Nebulizer | 3.0 Bar |
| Focus | Active | Set Capillary | 4500 V | Set Dry Heater | 200 °C |
| Scan Begin | 40 m/z | Set End Plate Offset | -500 V | Set Dry Gas | 10.0 l/min |
| Scan End | 1000 m/z | Set Collision Cell RF | 1000.0 Vpp | Set Divert Valve | Waste |

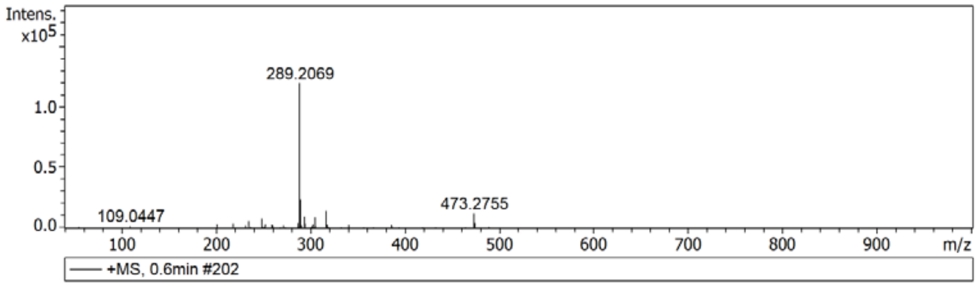


| # | m/z | Res. | S/N | I | I % | FWHM |
|---|---|---|---|---|---|---|
| 1 | 201.1079 | 12016245 | 193.4 | 3482 | 2.9 | 0.0000 |
| 2 | 218.1344 | 11687058 | 220.7 | 3972 | 3.3 | 0.0000 |
| 3 | 232.1132 | 13081037 | 130.1 | 2342 | 2.0 | 0.0000 |
| 4 | 235.1601 | 11267000 | 330.9 | 5956 | 5.0 | 0.0000 |
| 5 | 249.1395 | 12020219 | 458.9 | 8260 | 6.9 | 0.0000 |
| 6 | 252.6617 | 10117150 | 194.4 | 3500 | 2.9 | 0.0000 |
| 7 | 259.6525 | 12463248 | 172.7 | 3108 | 2.6 | 0.0000 |
| 8 | 287.1911 | 12179905 | 230.8 | 4154 | 3.5 | 0.0000 |
| 9 | 289.2069 | 12814264 | 6625.7 | 119262 | 100.0 | 0.0000 |
| 10 | 290.2102 | 12330454 | 1322.1 | 23798 | 20.0 | 0.0000 |
| 11 | 294.1995 | 12433872 | 537.1 | 9668 | 8.1 | 0.0000 |
| 12 | 294.7011 | 11945558 | 215.3 | 3876 | 3.2 | 0.0000 |
| 13 | 303.1862 | 11125337 | 193.7 | 3486 | 2.9 | 0.0000 |
| 14 | 305.2011 | 12088720 | 499.1 | 8984 | 7.5 | 0.0000 |
| 15 | 317.2020 | 12172409 | 799.0 | 14382 | 12.1 | 0.0000 |
| 16 | 318.2046 | 10761888 | 178.4 | 3212 | 2.7 | 0.0000 |
| 17 | 341.2377 | 10867029 | 149.6 | 2692 | 2.3 | 0.0000 |
| 18 | 386.2341 | 9805799 | 139.3 | 2508 | 2.1 | 0.0000 |
| 19 | 473.2755 | 9485927 | 684.2 | 12316 | 10.3 | 0.0000 |
| 20 | 474.2766 | 8524738 | 242.2 | 4360 | 3.7 | 0.0001 |

$^{1}$H- and $^{13}$C-NMR ($CDCl_3$, 300 MHz) of 98 (UCM-17221):

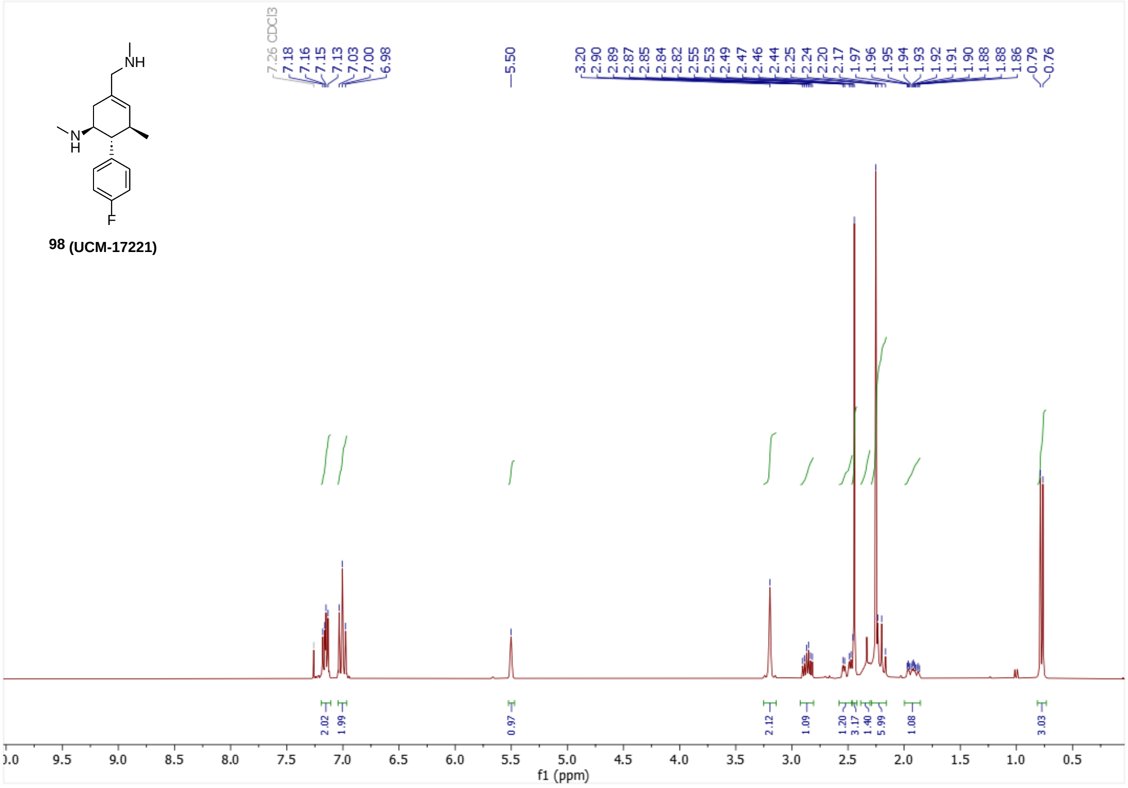

98 (UCM-17221)

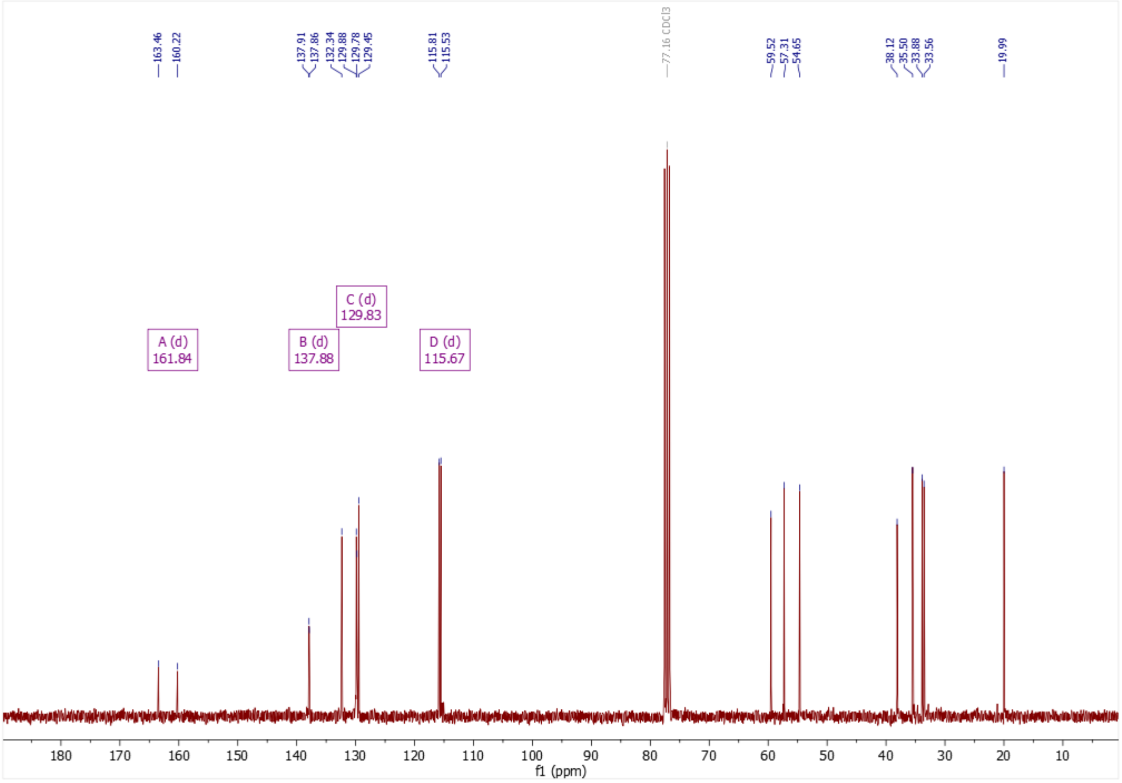

**HPLC-MS spectra of 98 (UCM-17221)**:

The HCl salt of compound **98** elutes as an early injection-front peak (3.063 min) and a retained peak (4.146 min), both exhibiting the expected molecular mass.

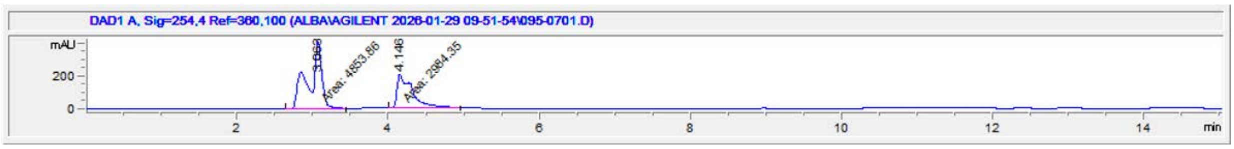


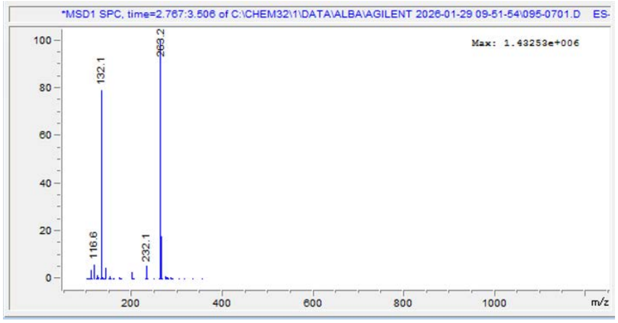


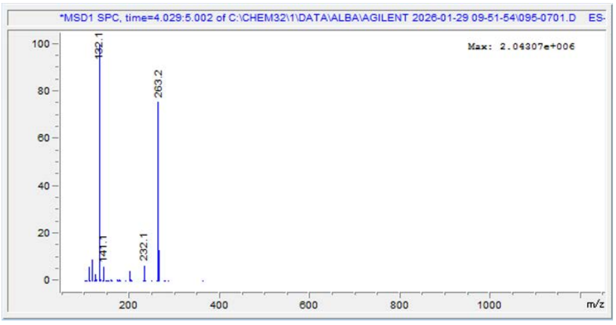


**HRMS spectra of 98 (UCM-17221)**:

**Acquisition Parameter**

| | | | | | |
|---|---|---|---|---|---|
| Source Type | ESI | Ion Polarity | Positive | Set Nebulizer | 3.0 Bar |
| Focus | Active | Set Capillary | 4500 V | Set Dry Heater | 200 °C |
| Scan Begin | 40 m/z | Set End Plate Offset | -500 V | Set Dry Gas | 10.0 l/min |
| Scan End | 1000 m/z | Set Collision Cell RF | 1000.0 Vpp | Set Divert Valve | Waste |

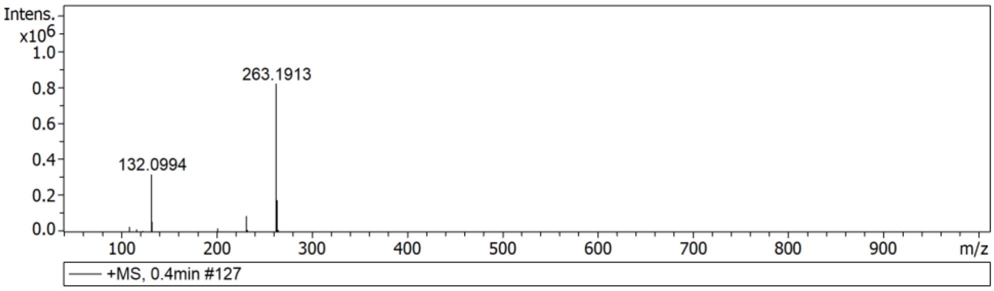


| # | m/z | Res. | S/N | I | I % | FWHM |
|---|---|---|---|---|---|---|
| 1 | 109.0450 | 12313690 | 1600.4 | 28808 | 3.5 | 0.0000 |
| 2 | 116.5786 | 11991336 | 834.0 | 15012 | 1.8 | 0.0000 |
| 3 | 123.0606 | 11070269 | 439.8 | 7916 | 1.0 | 0.0000 |
| 4 | 132.0994 | 13358334 | 17677.2 | 318190 | 38.9 | 0.0000 |
| 5 | 132.6011 | 12120359 | 3147.4 | 56654 | 6.9 | 0.0000 |
| 6 | 133.1033 | 9679393 | 265.4 | 4778 | 0.6 | 0.0000 |
| 7 | 173.0757 | 10802261 | 282.6 | 5086 | 0.6 | 0.0000 |
| 8 | 201.1071 | 12107756 | 1247.6 | 22456 | 2.7 | 0.0000 |
| 9 | 232.1493 | 13272933 | 4961.7 | 89310 | 10.9 | 0.0000 |
| 10 | 233.1525 | 11491905 | 753.4 | 13562 | 1.7 | 0.0000 |
| 11 | 249.1754 | 11584919 | 257.3 | 4632 | 0.6 | 0.0000 |
| 12 | 263.1913 | 13244727 | 45425.4 | 817658 | 100.0 | 0.0000 |
| 13 | 263.3117 | 11299350 | 316.6 | 5698 | 0.7 | 0.0000 |
| 14 | 263.3951 | 7053905 | 304.4 | 5480 | 0.7 | 0.0000 |
| 15 | 264.1947 | 12944951 | 9751.9 | 175534 | 21.5 | 0.0000 |
| 16 | 265.1980 | 10281049 | 704.0 | 12672 | 1.5 | 0.0000 |
| 17 | 276.1813 | 11791783 | 277.6 | 4996 | 0.6 | 0.0000 |
| 18 | 279.1862 | 10291694 | 261.3 | 4704 | 0.6 | 0.0000 |
| 19 | 285.1731 | 11018358 | 285.8 | 5144 | 0.6 | 0.0000 |
| 20 | 323.3176 | 10125673 | 283.3 | 5100 | 0.6 | 0.0000 |
